# Supplementary material for: New Benzofuranoids and Phenylpropanoids from the Mangrove Endophytic Fungus, Aspergillus sp. ZJ-68
Source: Mar Drugs. 2019 Aug 18;17(8):478. doi: 10.3390/md17080478 (PMC6723808; doi:10.3390/md17080478)
Supplement: Supplementary file 1 [file marinedrugs-17-00478-s001.zip › Supporting Information-revision with yollow mark.pdf]

# Supporting Information

# New Benzofuranoids and Phenylpropanoids from the Mangrove Endophytic Fungus, *Aspergillus* sp. ZJ-68

**Runlin Cai [1], Hongming Jiang [1], Zhenming Zang [1], Chunyuan Li [2,*] and Zhigang She [1,3,*]**

[1] School of Chemistry, Sun Yat-sen University, Guangzhou 510275, People's Republic of China; cairlin@mail2.sysu.edu.cn (R.C.); jianghm7@mail2.sysu.edu.cn (H.J.); zangzhm@mail2.sysu.edu.cn (Z.Z.); cesshzhg@mail.sysu.edu.cn (Z. S.)

[2] College of Materials and Energy, South China Agricultural University, Guangzhou 510642, People's Republic of China; chunyuanli@scau.edu.cn (C.L.)

[3] South China Sea Bio-Resource Exploitation and Utilization Collaborative Innovation Center, Guangzhou 510006, People's Republic of China

* Correspondence: chunyuanli@scau.edu.cn (C.L); cesshzhg@mail.sysu.edu.cn (Z. S.); Tel./Fax: +86-020-84113356 (Z. S.)

**The List of Contents**


| No. | Content | page |
|---|---|---|
| **1** | Figure S1. HRESIMS spectrum of **1** | 1 |
| **2** | Figure S2. $^{1}$H NMR spectrum of **1** in $CDCl_3$ | 1 |
| **3** | Figure S3. $^{13}$C NMR spectrum of **1** in $CDCl_3$ | 2 |
| **4** | Figure S4. DEPT spectrum of **1** in $CDCl_3$ | 2 |
| **5** | Figure S5. $^{1}$H-$^{1}$H COSY spectrum of **1** in $CDCl_3$ | 3 |
| **6** | Figure S6. HSQC spectrum of **1** in $CDCl_3$ | 3 |
| **7** | Figure S7. HMBC spectrum of **1** in $CDCl_3$ | 4 |
| **8** | Figure S8. IR spectrum of **1** | 4 |
| **9** | Figure S9. UV data of **1** in MeOH | 5 |
| **10** | Figure S10. HRESIMS spectrum of **2** | 5 |
| **11** | Figure S11. $^{1}$H NMR spectrum of **2** in $CDCl_3$ | 6 |
| **12** | Figure S12. $^{13}$C NMR spectrum of **2** in $CDCl_3$ | 6 |
| **13** | Figure S13. DEPT spectrum of **2** in $CDCl_3$ | 7 |
| **14** | Figure S14. $^{1}$H-$^{1}$H COSY spectrum of **2** in $CDCl_3$ | 7 |
| **15** | Figure S15. HSQC spectrum of **2** in $CDCl_3$ | 8 |
| **16** | Figure S16. HMBC spectrum of **2** in $CDCl_3$ | 8 |
| **17** | Figure S17. IR spectrum of **2** | 9 |
| **18** | Figure S18. UV data of **2** in MeOH | 9 |
| **19** | Figure S19. HRESIMS spectrum of **3** | 10 |
| **20** | Figure S20. $^{1}$H NMR spectrum of **3** in $CDCl_3$ | 10 |
| **21** | Figure S21. $^{13}$C NMR spectrum of **3** in $CDCl_3$ | 11 |
| **22** | Figure S22. DEPT spectrum of **3** in $CDCl_3$ | 11 |
| **23** | Figure S23. $^{1}$H-$^{1}$H COSY spectrum of **3** in $CDCl_3$ | 12 |
| **24** | Figure S24. HSQC spectrum of **3** in $CDCl_3$ | 12 |
| **25** | Figure S25. HMBC spectrum of **3** in $CDCl_3$ | 13 |
| **26** | Figure S26. IR spectrum of **3** | 13 |
| **27** | Figure S27. UV data of **3** in MeOH | 14 |

| | | |
|---|---|---|
| **28** | Figure S28. HRESIMS spectrum of **4** | 14 |
| **29** | Figure S29. $^{1}$H NMR spectrum of **4** in $CDCl_3$ | 15 |
| **30** | Figure S30. $^{13}$C NMR spectrum of **4** in $CDCl_3$ | 15 |
| **31** | Figure S31. $^{1}$H-$^{1}$H COSY spectrum of **4** in $CDCl_3$ | 16 |
| **32** | Figure S32. HSQC spectrum of **4** in $CDCl_3$ | 16 |
| **33** | Figure S33. HMBC spectrum of **4** in $CDCl_3$ | 17 |
| **34** | Figure S34. IR spectrum of **4** | 17 |
| **35** | Figure S35. UV data of **4** in MeOH | 18 |
| **36** | Figure S36. HRESIMS spectrum of **5** | 18 |
| **37** | Figure S37. $^{1}$H NMR spectrum of **5** in $CDCl_3$ | 19 |
| **38** | Figure S38. $^{13}$C NMR spectrum of **5** in $CDCl_3$ | 19 |
| **39** | Figure S39. $^{1}$H-$^{1}$H COSY spectrum of **5** in $CDCl_3$ | 20 |
| **40** | Figure S40. HSQC spectrum of **5** in $CDCl_3$ | 20 |
| **41** | Figure S41. HMBC spectrum of **5** in $CDCl_3$ | 21 |
| **42** | Figure S42. IR spectrum of **5** | 21 |
| **43** | Figure S43. UV data of **5** in MeOH | 22 |
| **44** | Figure S44. HRESIMS spectrum of **6** | 22 |
| **45** | Figure S45. $^{1}$H NMR spectrum of **6** in acetone-$d_6$ | 23 |
| **46** | Figure S46. $^{13}$C NMR spectrum of **6** in acetone-$d_6$ | 23 |
| **47** | Figure S47. DEPT spectrum of **6** in acetone-$d_6$ | 24 |
| **48** | Figure S48. $^{1}$H-$^{1}$H COSY spectrum of **6** in acetone-$d_6$ | 24 |
| **49** | Figure S49. HSQC spectrum of **6** in acetone-$d_6$ | 25 |
| **50** | Figure S50. HMBC spectrum of **6** in acetone-$d_6$ | 25 |
| **51** | Figure S51. IR spectrum of **6** | 26 |
| **52** | Figure S52. UV data of **6** in MeOH | 26 |
| **53** | Figure S53. HRESIMS spectrum of **7** | 27 |
| **54** | Figure S54. $^{1}$H NMR spectrum of **7** in methanol-$d_4$ | 27 |
| **55** | Figure S55. $^{13}$C NMR spectrum of **7** in methanol-$d_4$ | 28 |
| **56** | Figure S56. DEPT spectrum of **7** in methanol-$d_4$ | 28 |

| | | |
|---|---|---|
| **57** | Figure S57. $^{1}H$-$^{1}H$ COSY spectrum of **7** in methanol-$d_4$ | 29 |
| **58** | Figure S58. HSQC spectrum of **7** in methanol-$d_4$ | 29 |
| **59** | Figure S59. HMBC spectrum of **7** in methanol-$d_4$ | 30 |
| **60** | Figure S60. NOESY spectrum of **7** in methanol-$d_4$ | 30 |
| **61** | Figure S61. IR spectrum of **7** | 31 |
| **62** | Figure S62. UV data of **7** in MeOH | 31 |
| **63** | Preparation of MTPA esters of **1** by the modified Mosher's method | 32 |
| **64** | Figure S63. ESIMS spectrum of *(S)*-MTPA ester (**1a)** | 32 |
| **65** | Figure S64. ESIMS spectrum of *(R)*-MTPA ester (**1b)** | 33 |
| **66** | Figure S65. $^{1}H$ NMR spectrum of *(S)*-MTPA ester (**1a)** in $CDCl_3$ | 33 |
| **68** | Figure S66. $^{1}H$ NMR spectrum of *(R)*-MTPA ester (**1b)** in $CDCl_3$ | 34 |
| **69** | The $^{1}H$ and $^{13}C$ NMR data and HRMS data of compounds **8–14** | 35 |
| **70** | The rDNA sequencing data of *Aspergillus* sp. ZJ-68 | 37 |
| **71** | Figure S67. Simple picture and microscopic picture of *Aspergillus* sp. ZJ-68 | 37 |
| **72** | Figure S68. The chiral HPLC separation of (±)-penicisochroman A | 38 |
| **73** | ECD calculation details (Methods and Results) | 39 |

**Figure S1**. HRESIMS spectrum of **1**

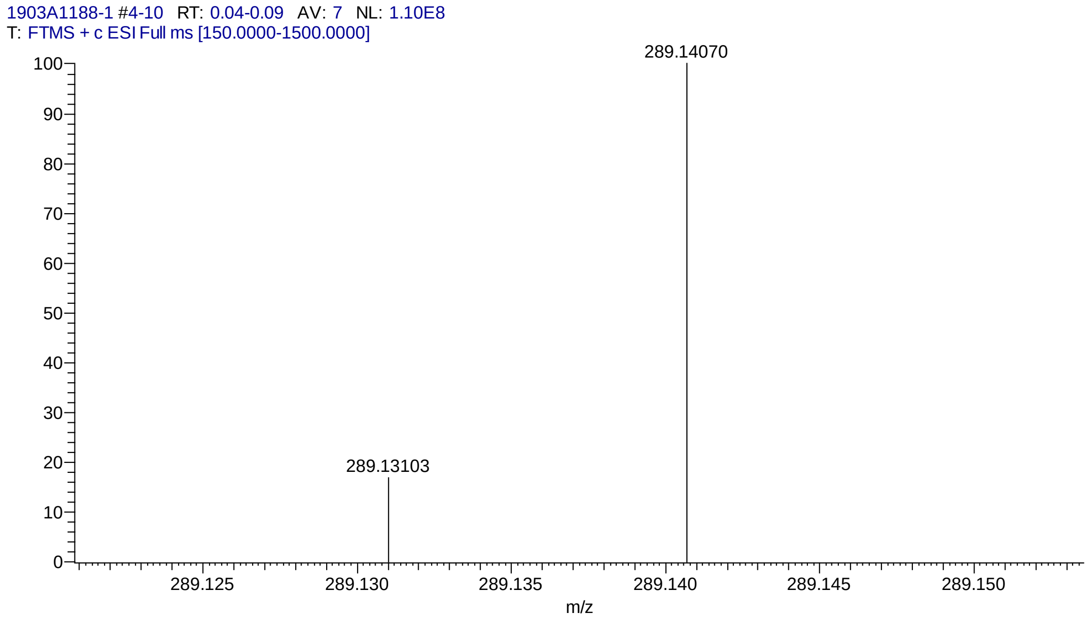


| m/z | Theo. Mass | Delta (mmu) | RDB equiv. | Composition |
|---|---|---|---|---|
| 289.14070 | 289.14103 | -0.33 | 4.5 | $C_{15}H_{22}O_4Na$ |

**Figure S2**. $^1$H NMR spectrum of **1** in $CDCl_3$

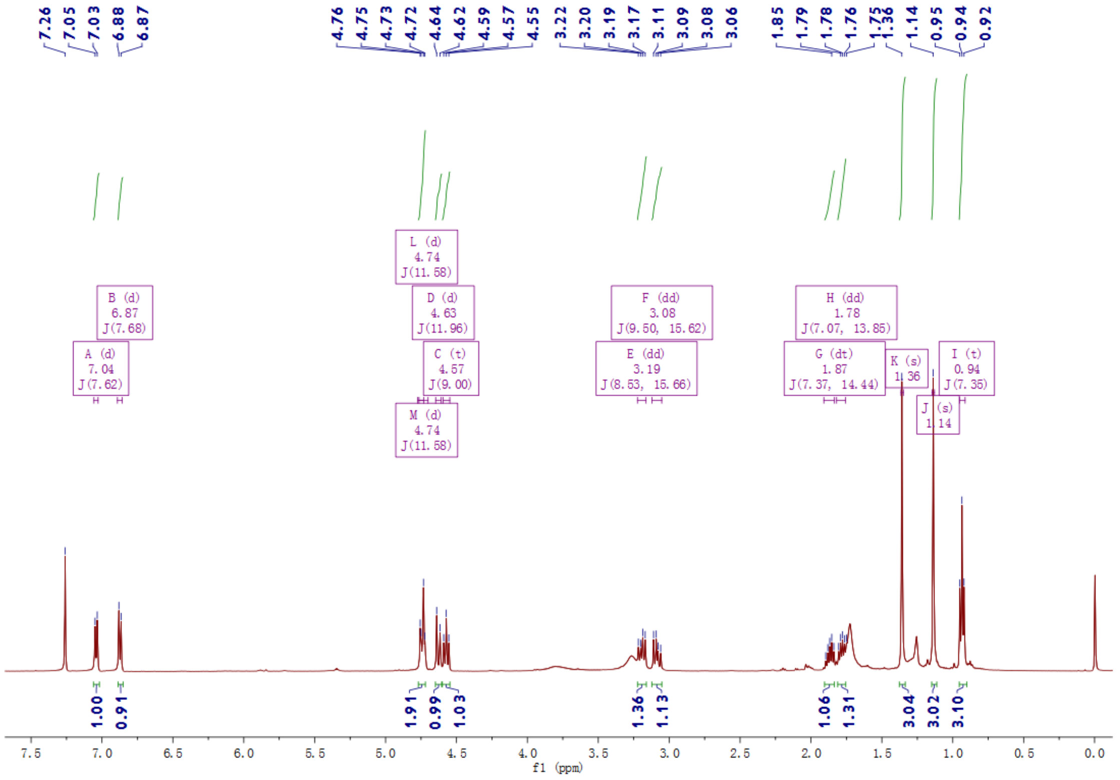

**Figure S3**. $^{13}$C NMR spectrum of **1** in $CDCl_3$

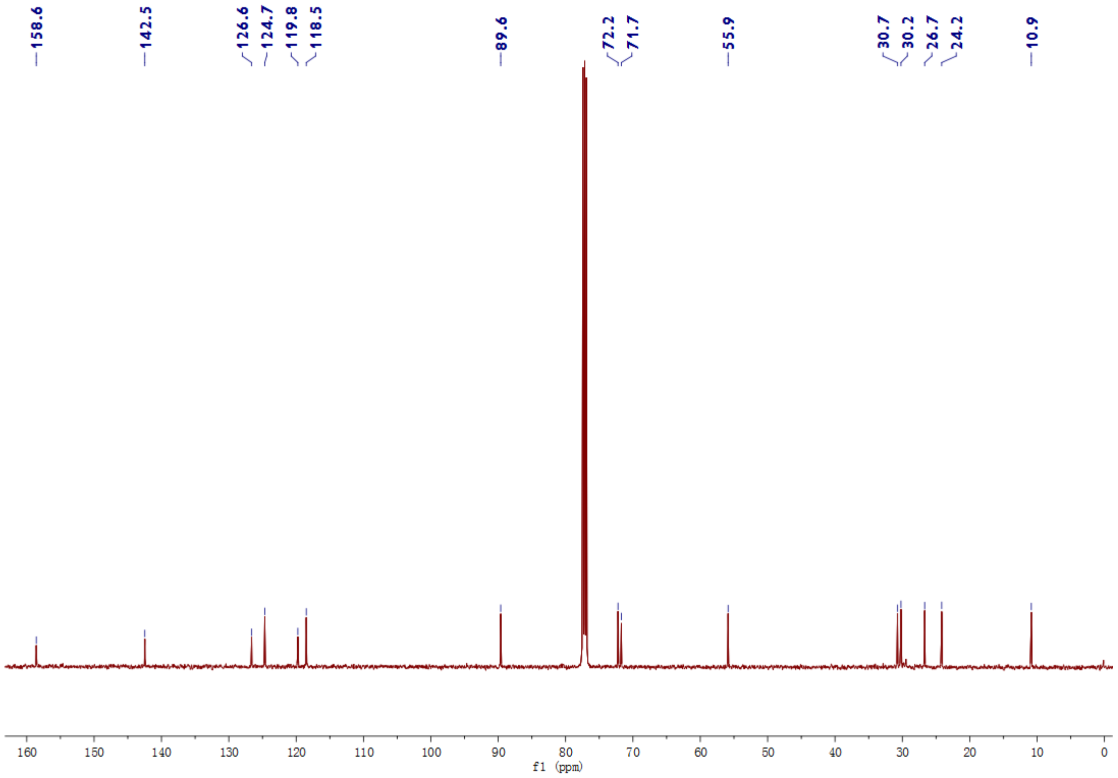


**Figure S4**. DEPT spectrum of **1** in $CDCl_3$

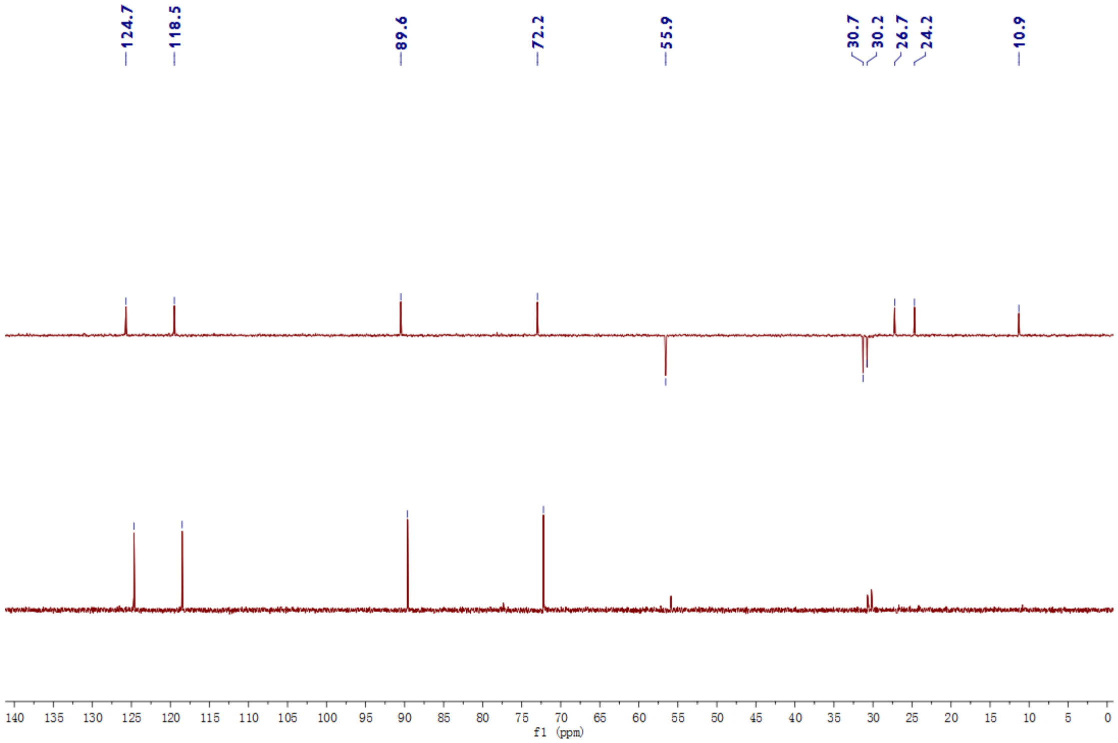

**Figure S5**. $^1H$-$^1H$ COSY spectrum of **1** in $CDCl_3$

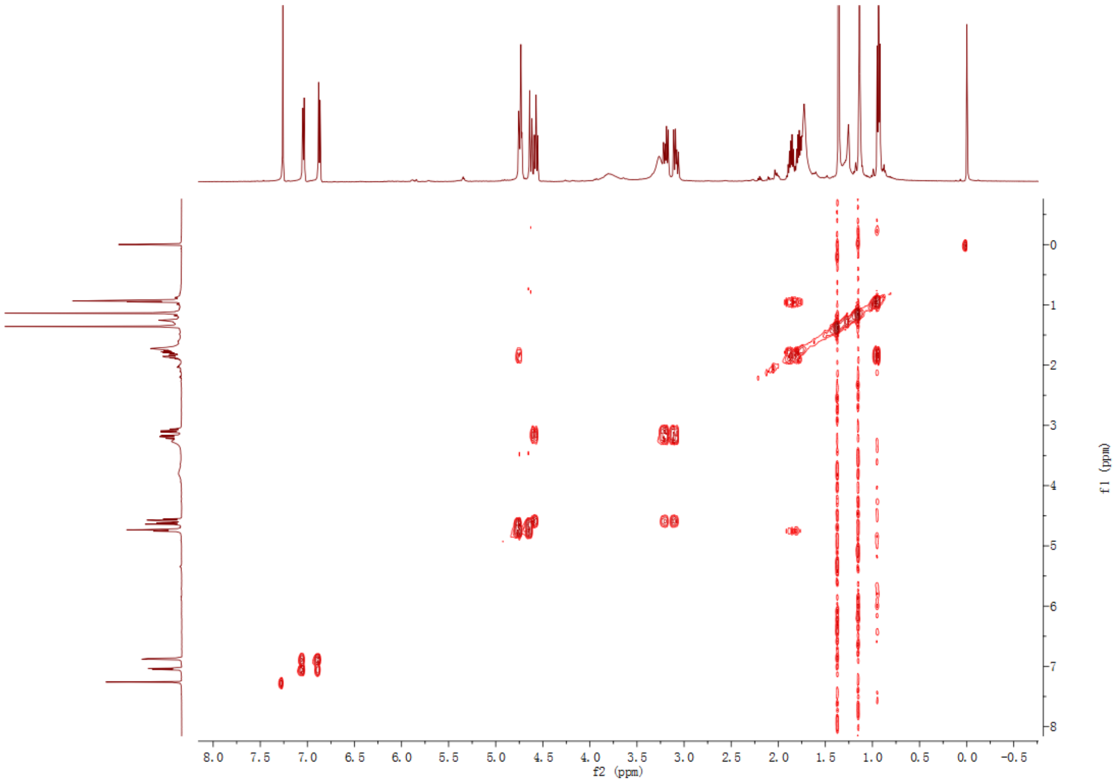


**Figure S6**. HSQC spectrum of **1** in $CDCl_3$

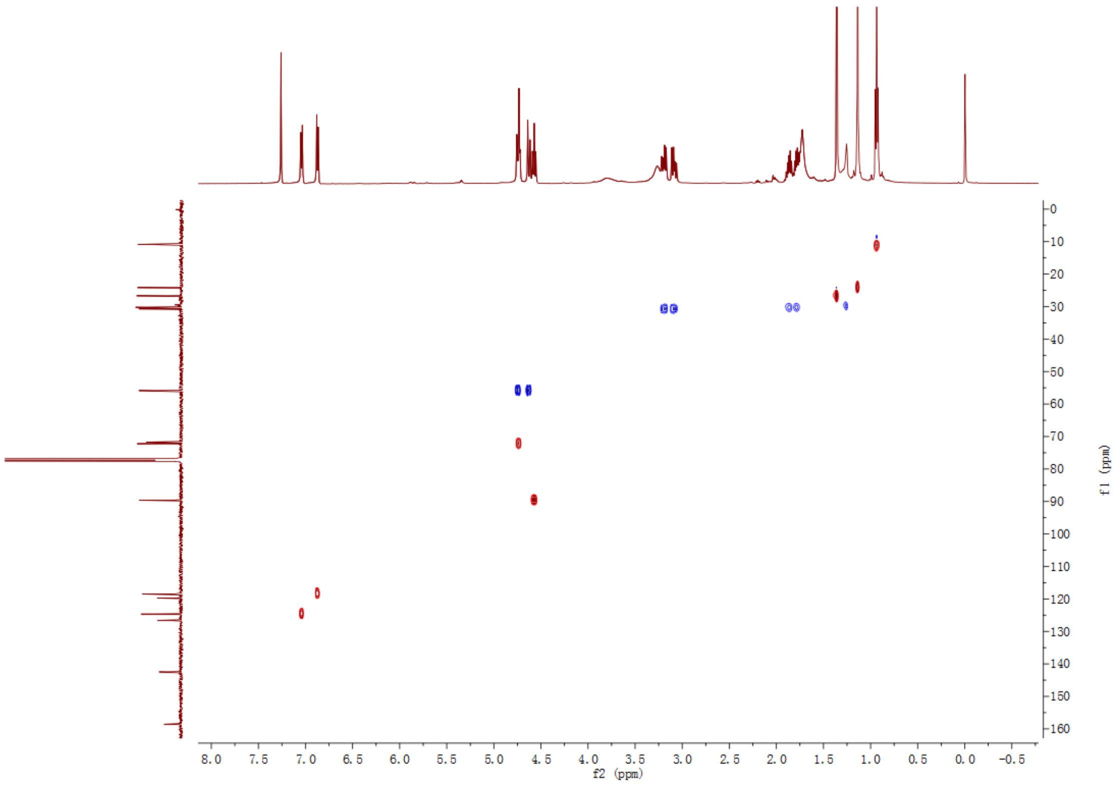

**Figure S7**. HMBC spectrum of **1** in $CDCl_3$

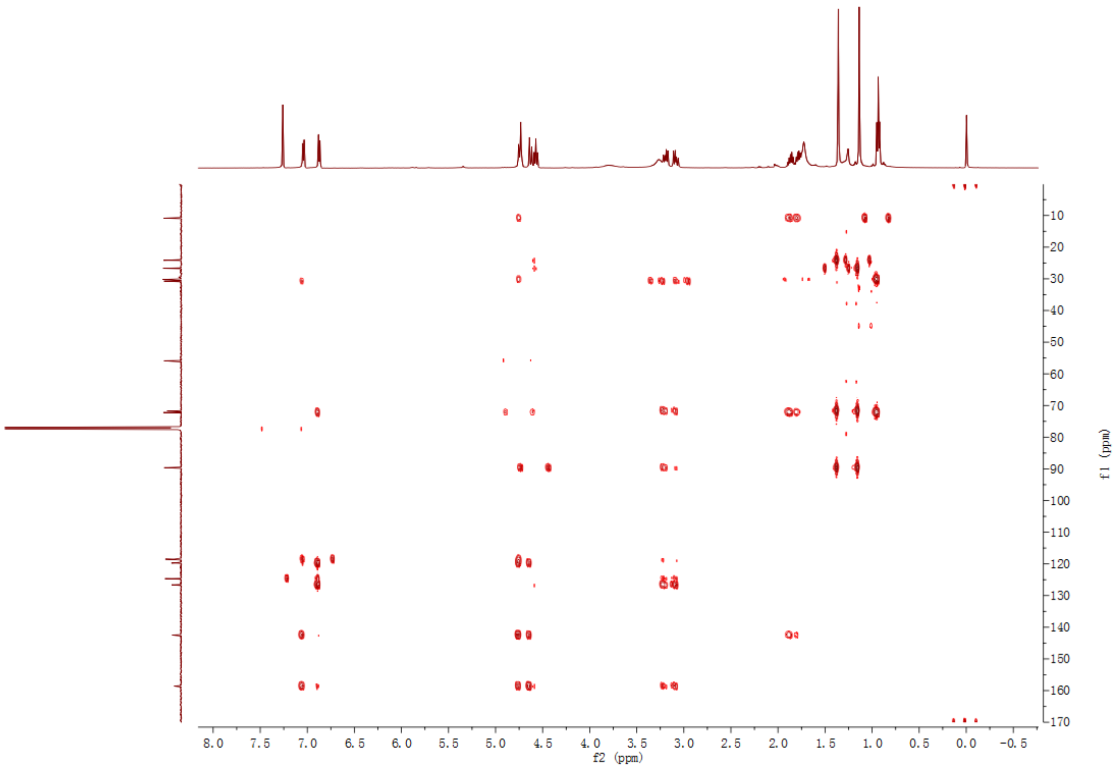


**Figure S8**. IR spectrum of **1**

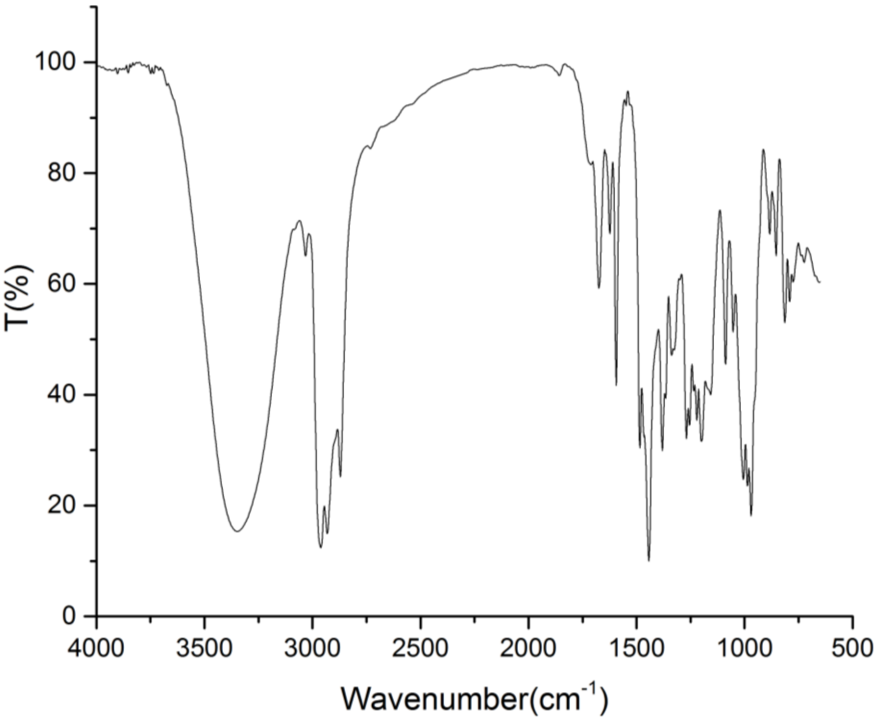

**Figure S9**. UV data of **1** in MeOH

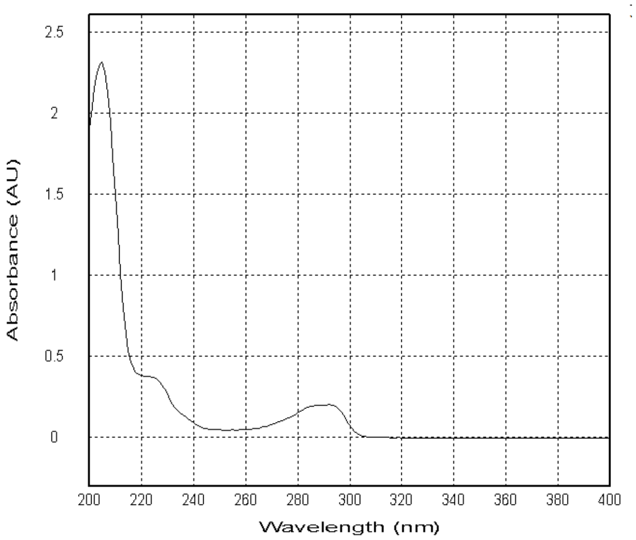


**Figure S10**. HRESIMS spectrum of **2**

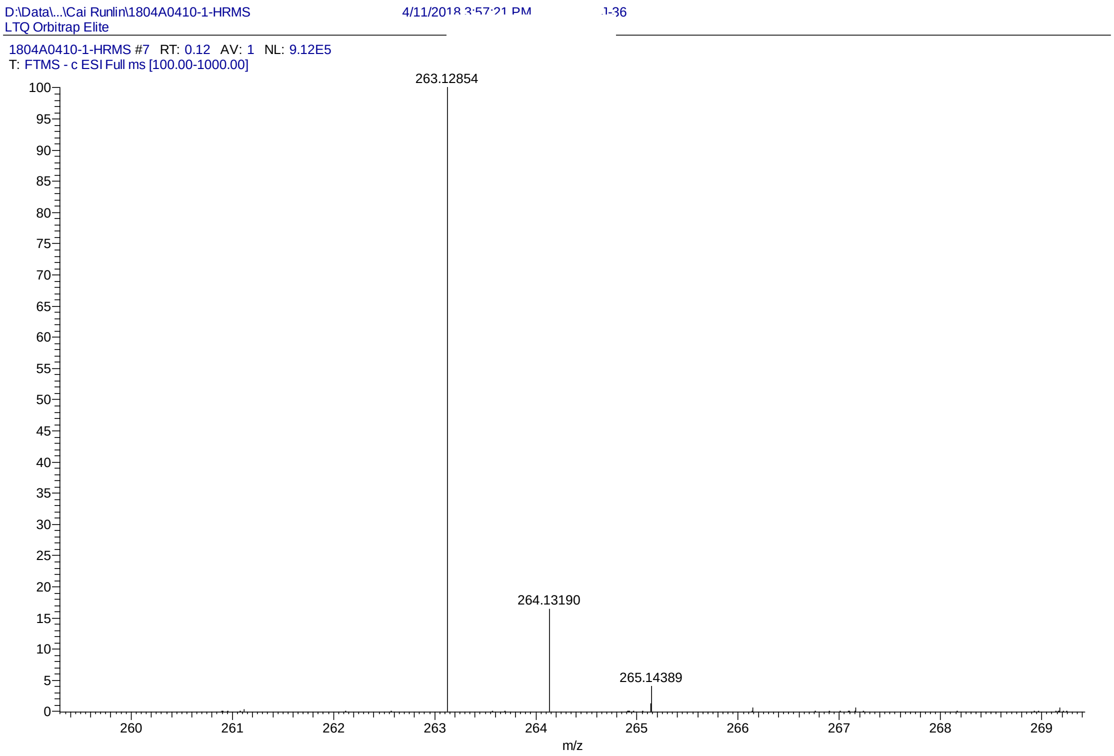


| SPECTRUM - simulation : | | | | |
|---|---|---|---|---|
| m/z | Theo. Mass | Delta (ppm) | RDB equiv. | Composition |
| 263.12854 | 263.12888 | -1.3 | 6.5 | $C_{15}H_{19}O_4$ |

**Figure S11**. $^{1}$H NMR spectrum of **2** in $CDCl_3$

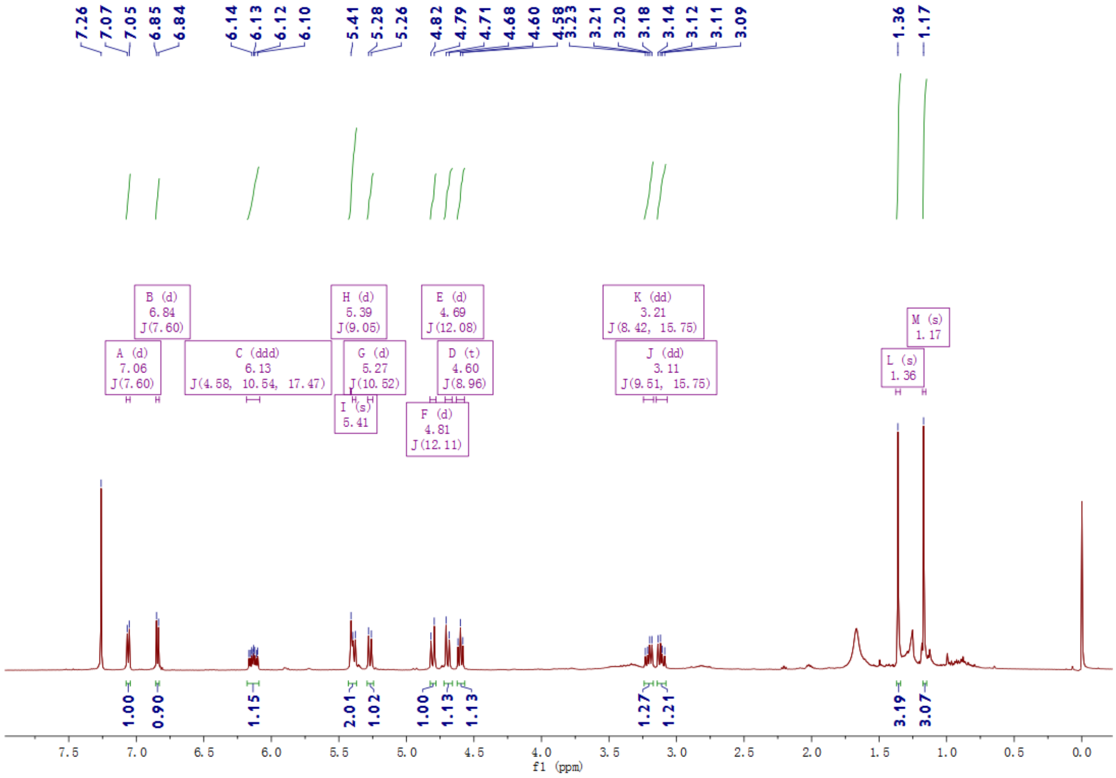

**Figure S12**. $^{13}$C NMR spectrum of **2** in $CDCl_3$

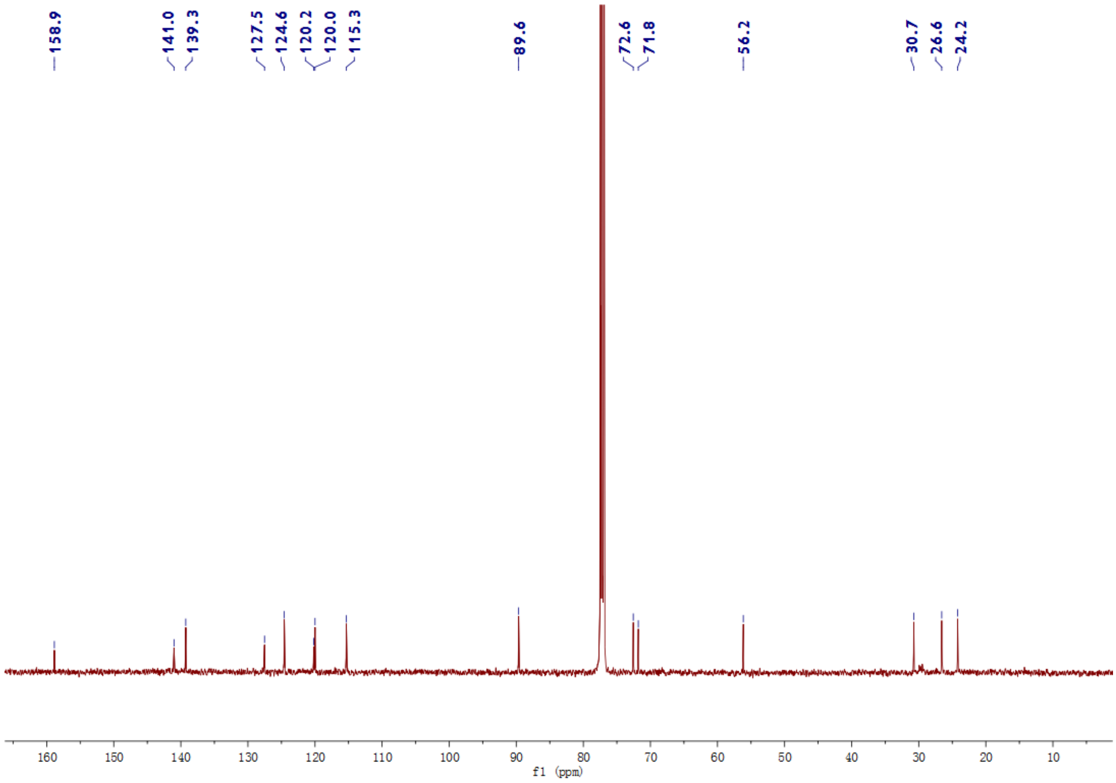

**Figure S13**. DEPT spectrum of **2** in $CDCl_3$

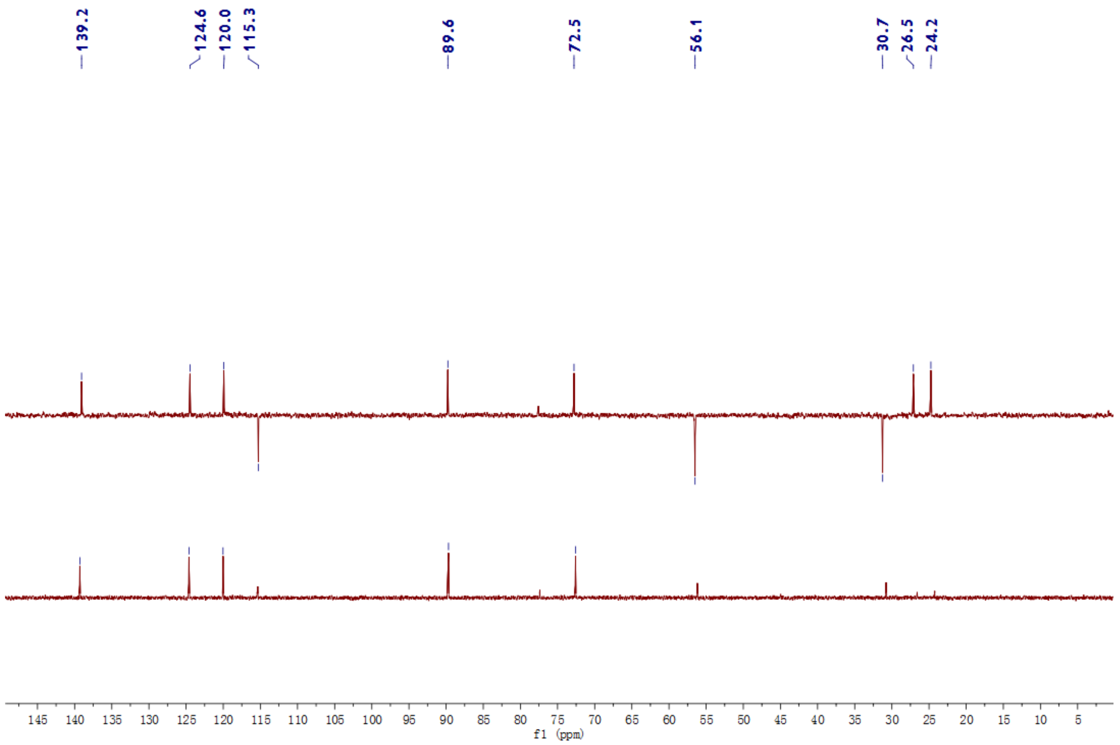


**Figure S14**. $^{1}$H-$^{1}$H COSY spectrum of **2** in $CDCl_3$

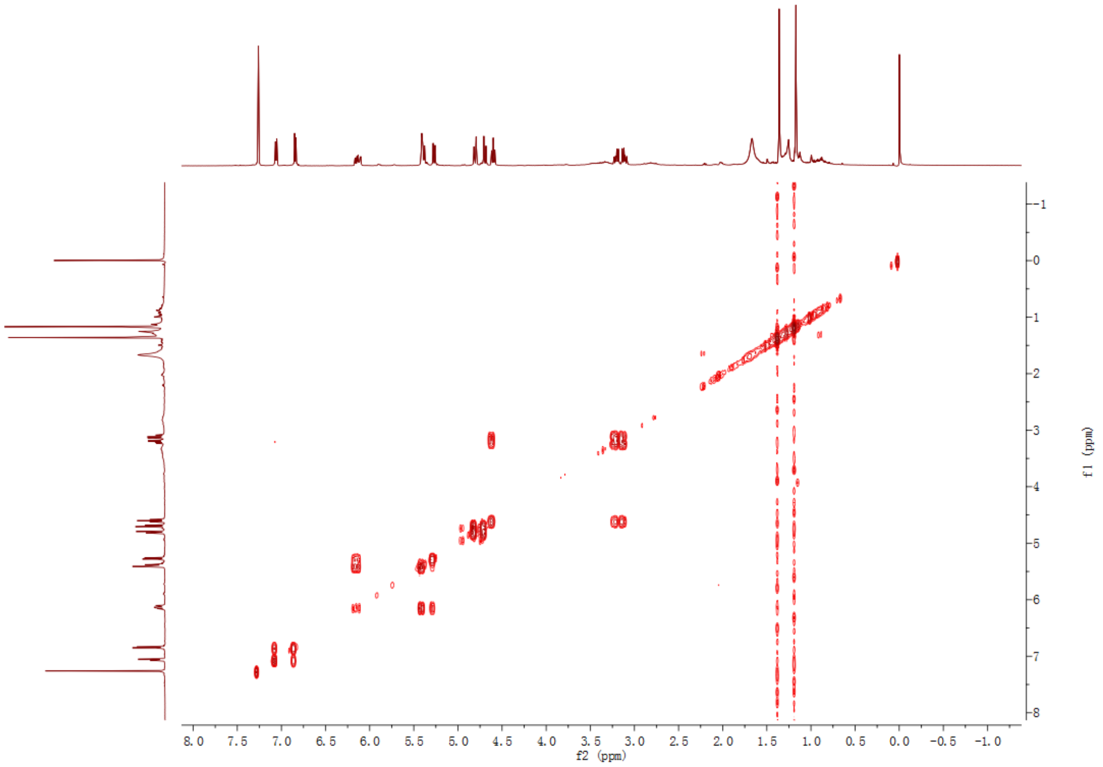

**Figure S15**. HSQC spectrum of **2** in $CDCl_3$

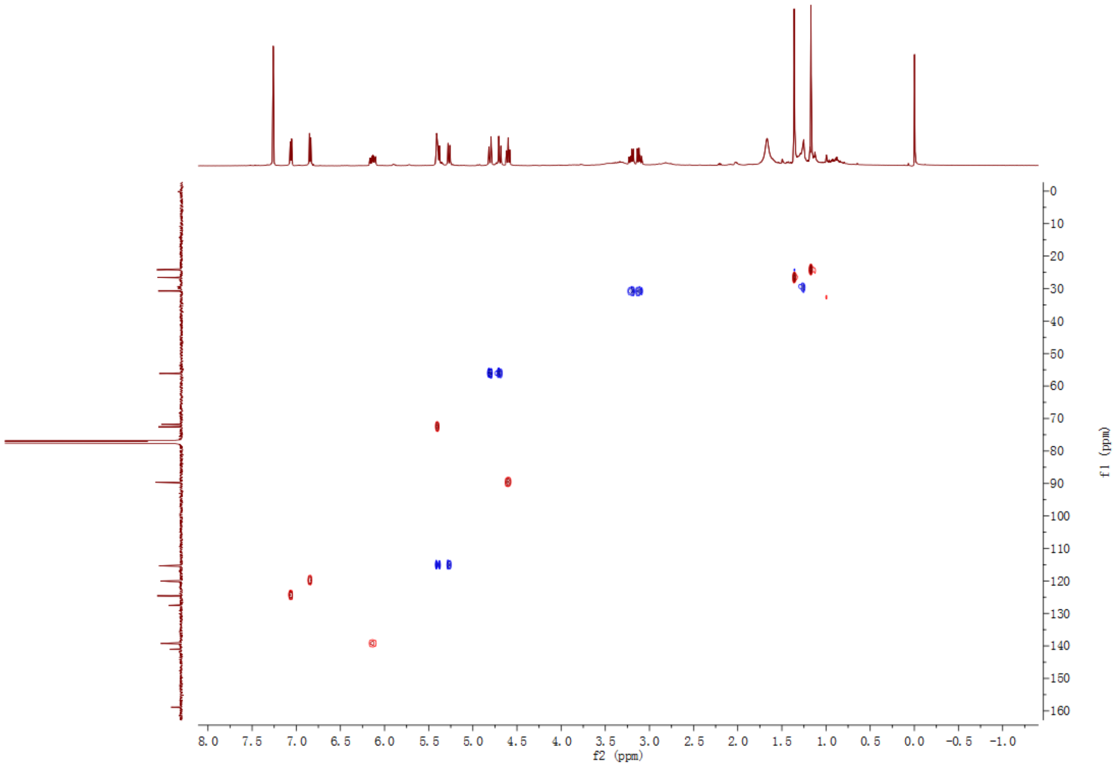


**Figure S16**. HMBC spectrum of **2** in $CDCl_3$

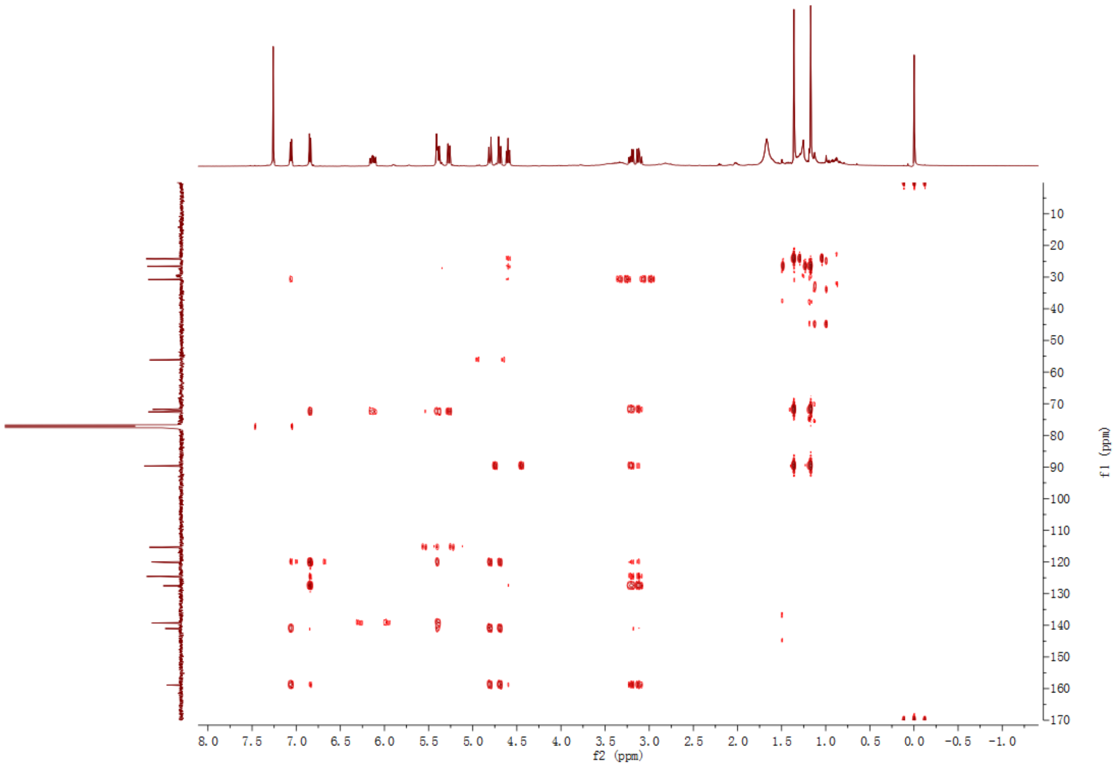

**Figure S17**. IR spectrum of **2**

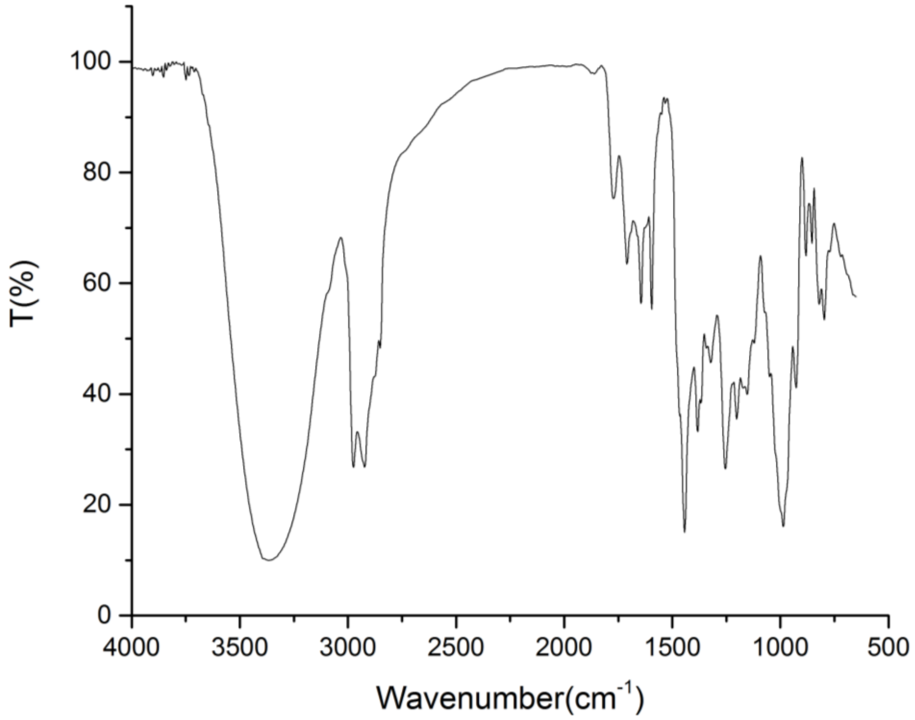


**Figure S18**. UV data of **2** in MeOH

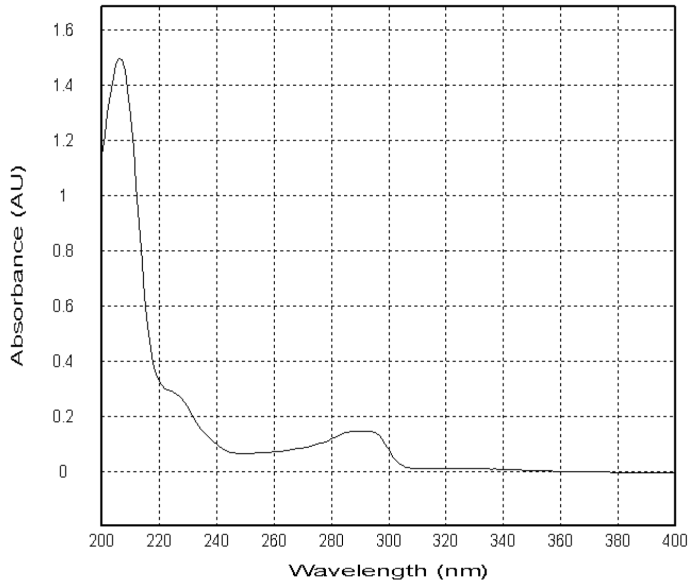

**Figure S19**. HRESIMS spectrum of **3**

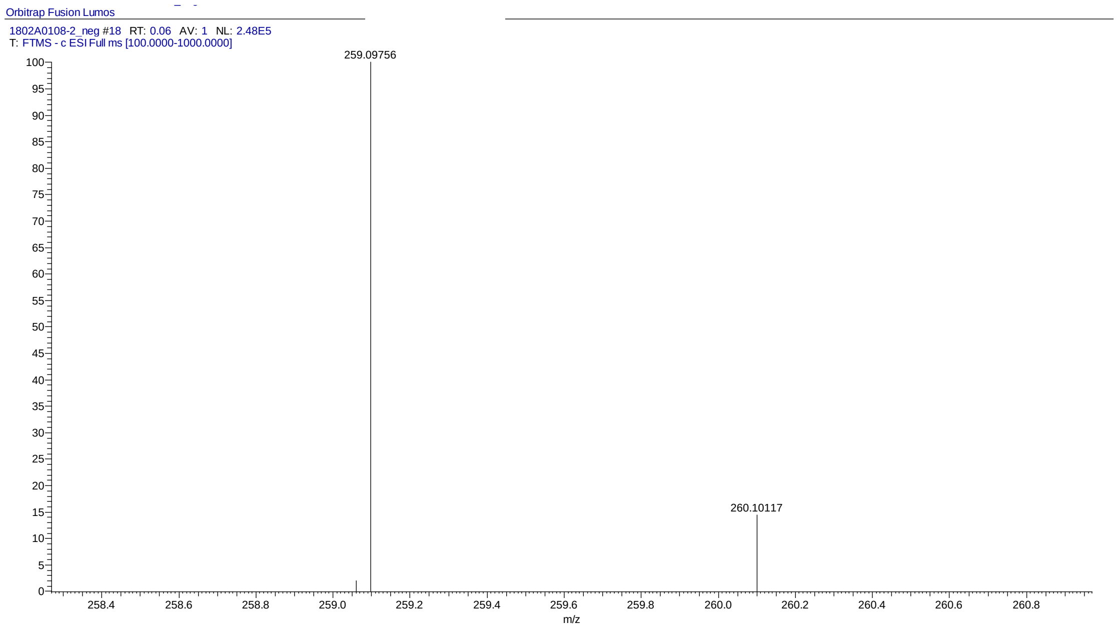


SPECTRUM - simulation :

| m/z | Theo. Mass | Delta (ppm) | RDB equiv. | Composition |
|---|---|---|---|---|
| 259.09756 | 259.09758 | -0.09 | 8.5 | $C_{15}H_{15}O_4$ |

**Figure S20**. $^1$H NMR spectrum of **3** in $CDCl_3$

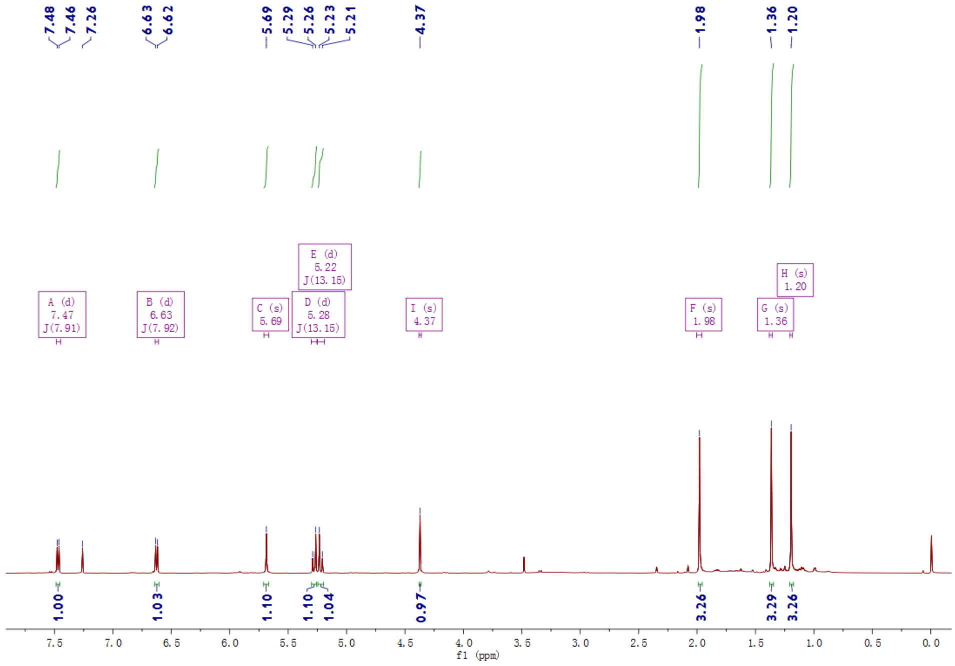

**Figure S21**. $^{13}$C NMR spectrum of **3** in $CDCl_3$

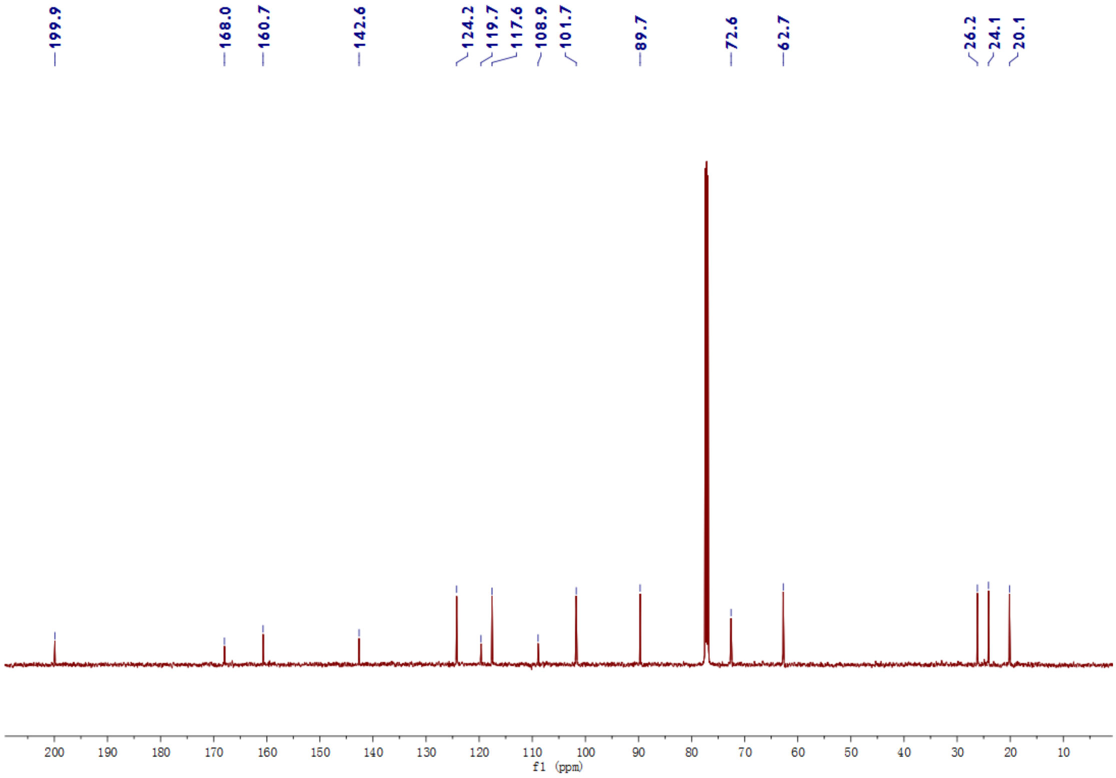


**Figure S22**. DEPT spectrum of **3** in $CDCl_3$

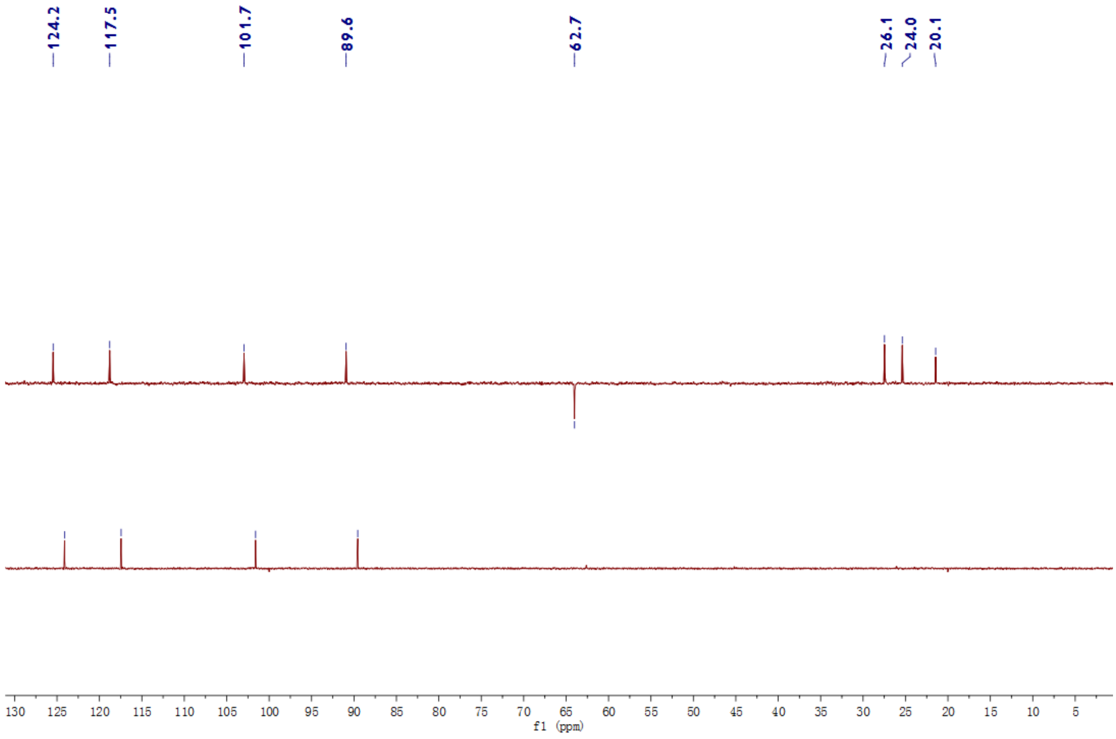

**Figure S23**. $^{1}H$-$^{1}H$ COSY spectrum of **3** in $CDCl_3$

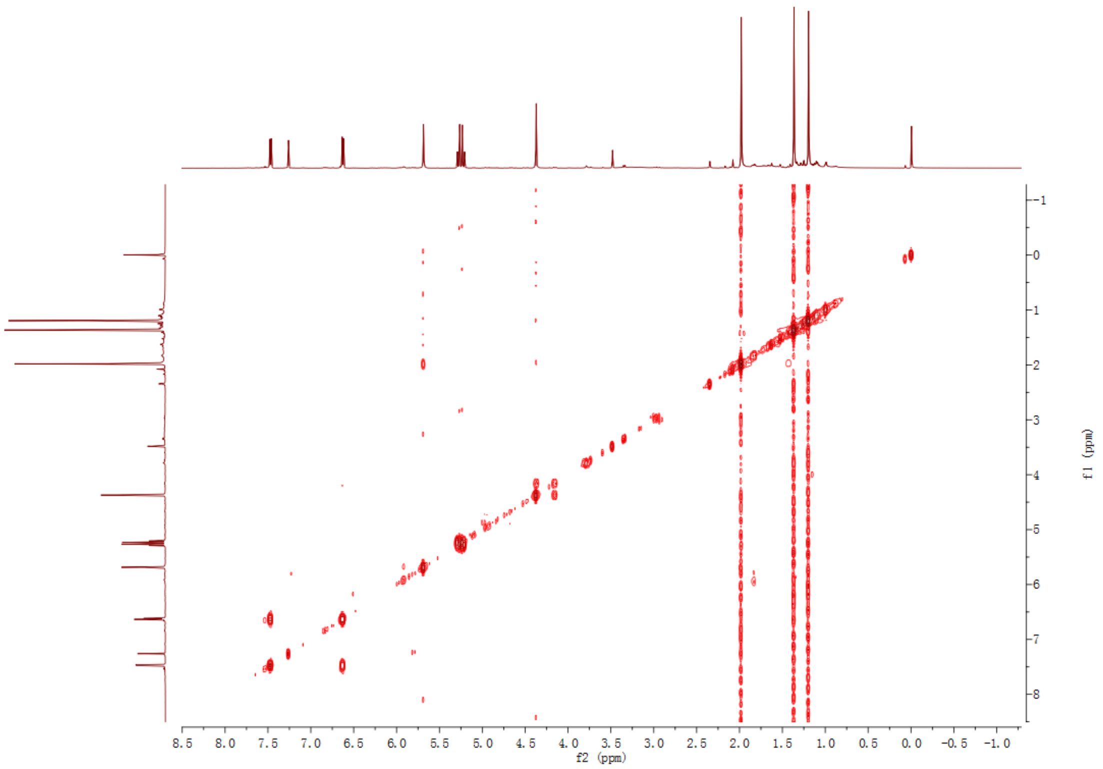

**Figure S24**. HSQC spectrum of **3** in $CDCl_3$

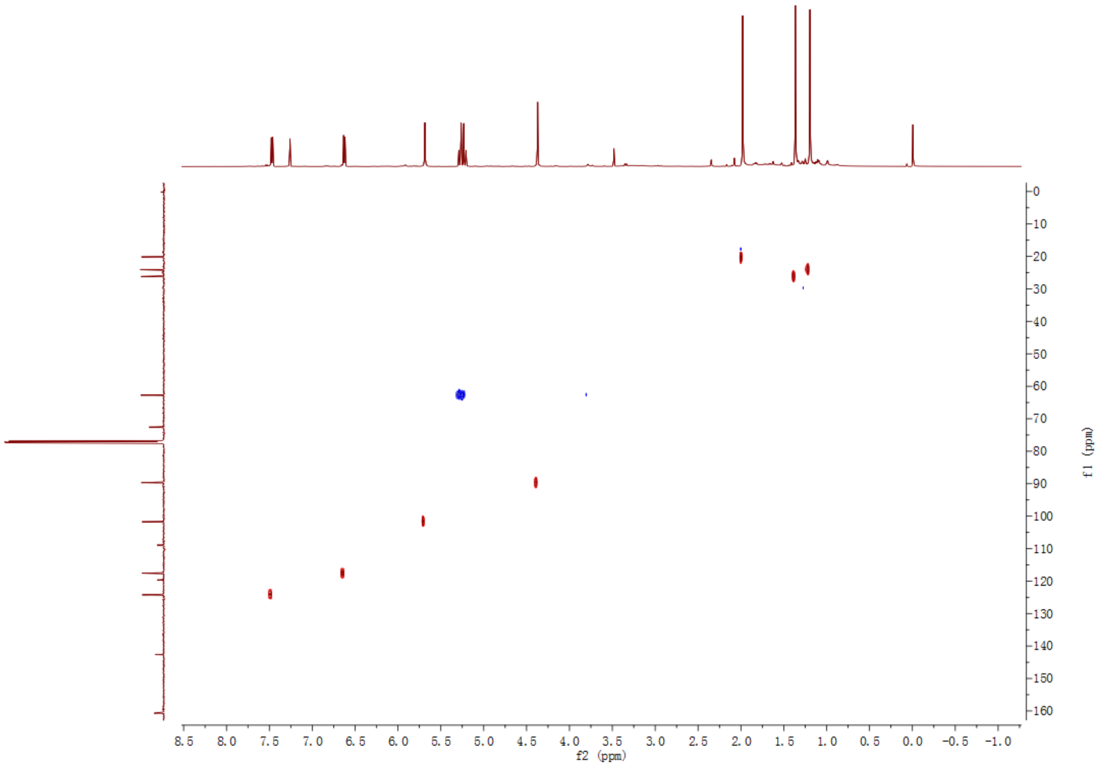

**Figure S25**. HMBC spectrum of **3** in $CDCl_3$

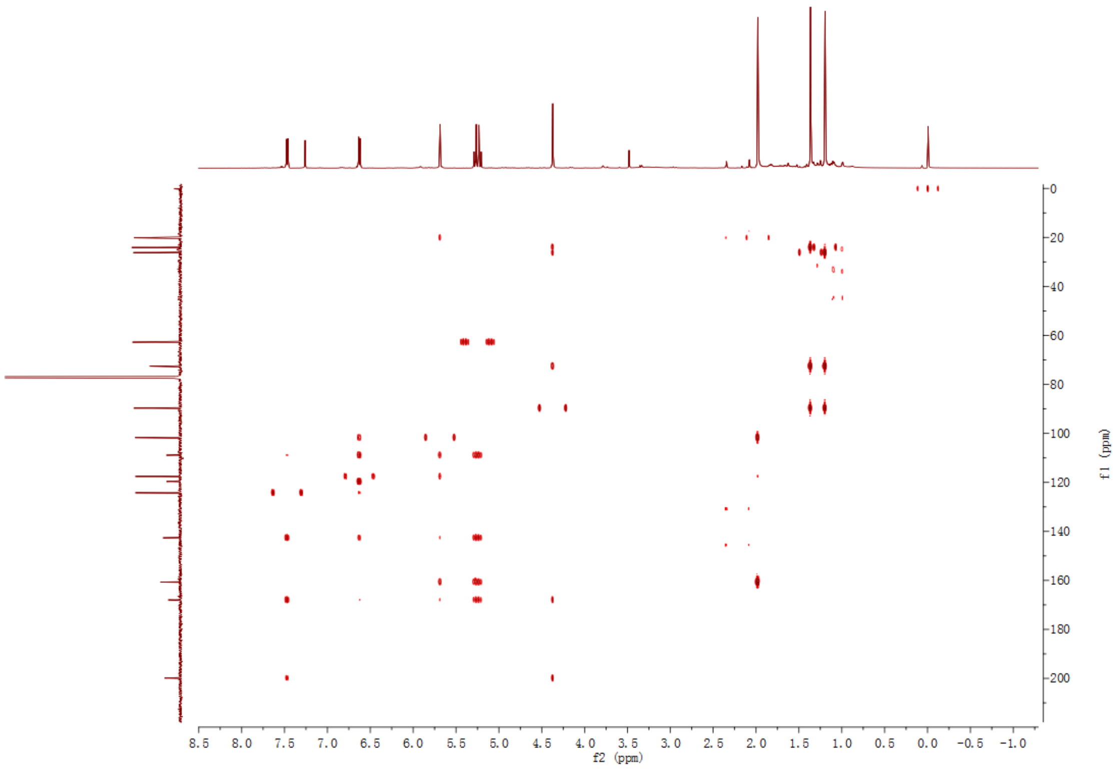


**Figure S26**. IR spectrum of **3**

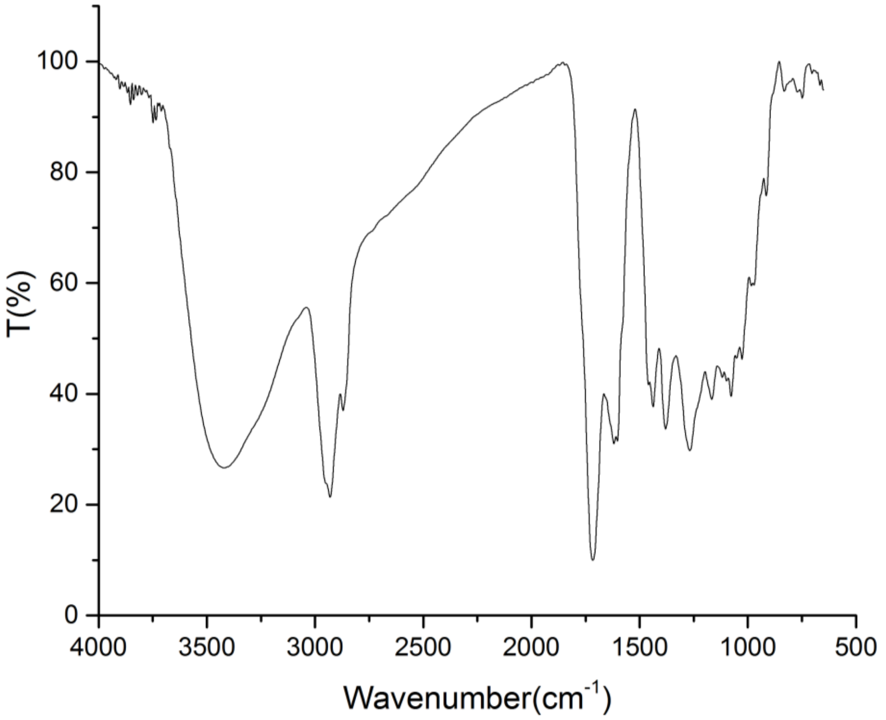

**Figure S27**. UV data of **3** in MeOH

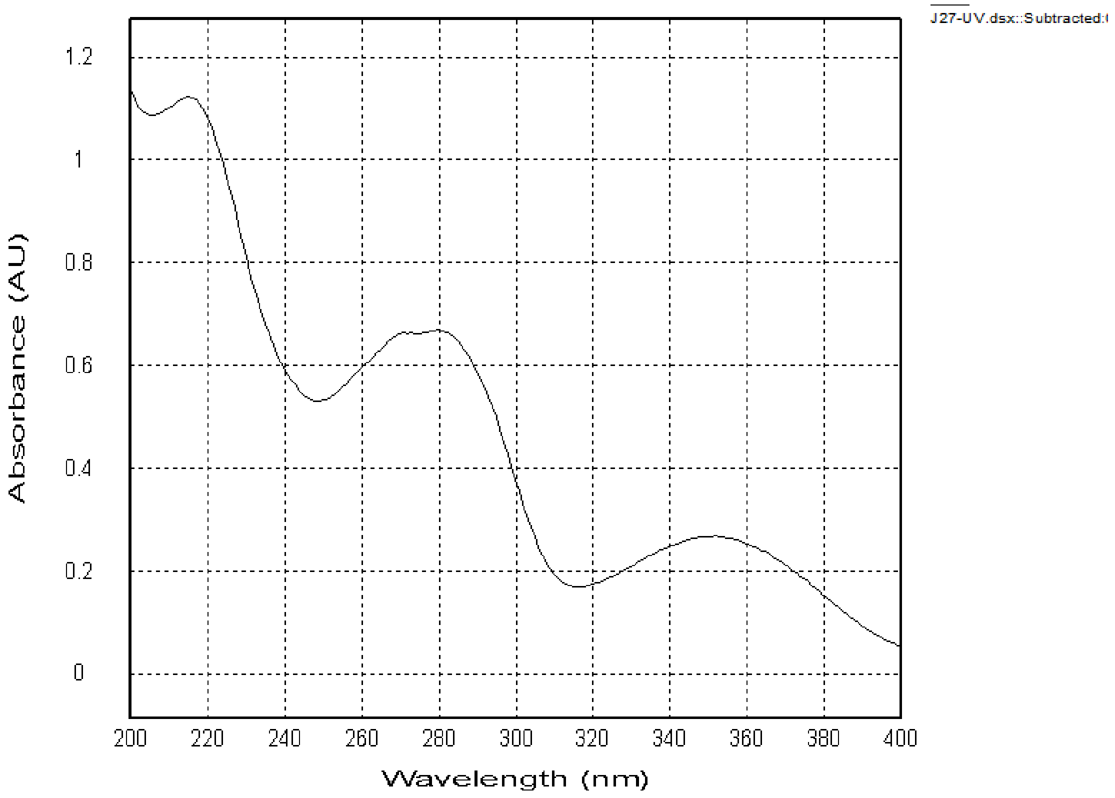


**Figure S28**. HRESIMS spectrum of **4**

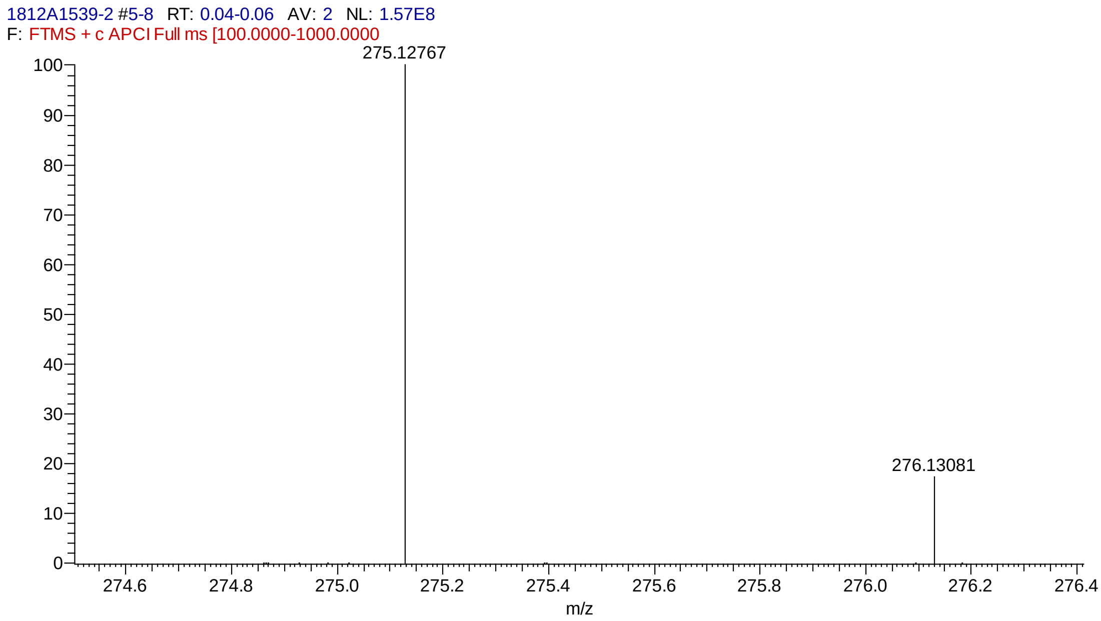


| m/z | Theo. Mass | Delta (mmu) | RDB equiv. | Composition |
|---|---|---|---|---|
| 275.12767 | 275.12779 | -0.12 | 7.5 | $C_{16}H_{19}O_4$ |

**Figure S29**. $^{1}H$ NMR spectrum of **4** in $CDCl_3$

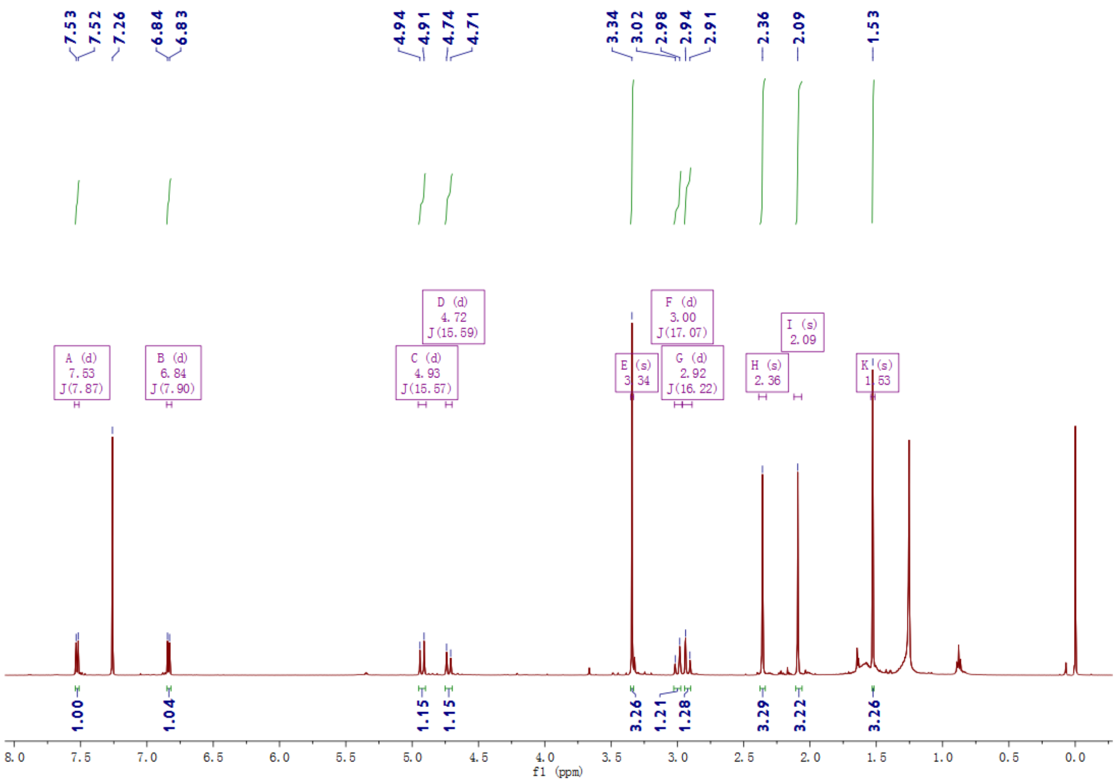

**Figure S30**. $^{13}C$ NMR spectrum of **4** in $CDCl_3$

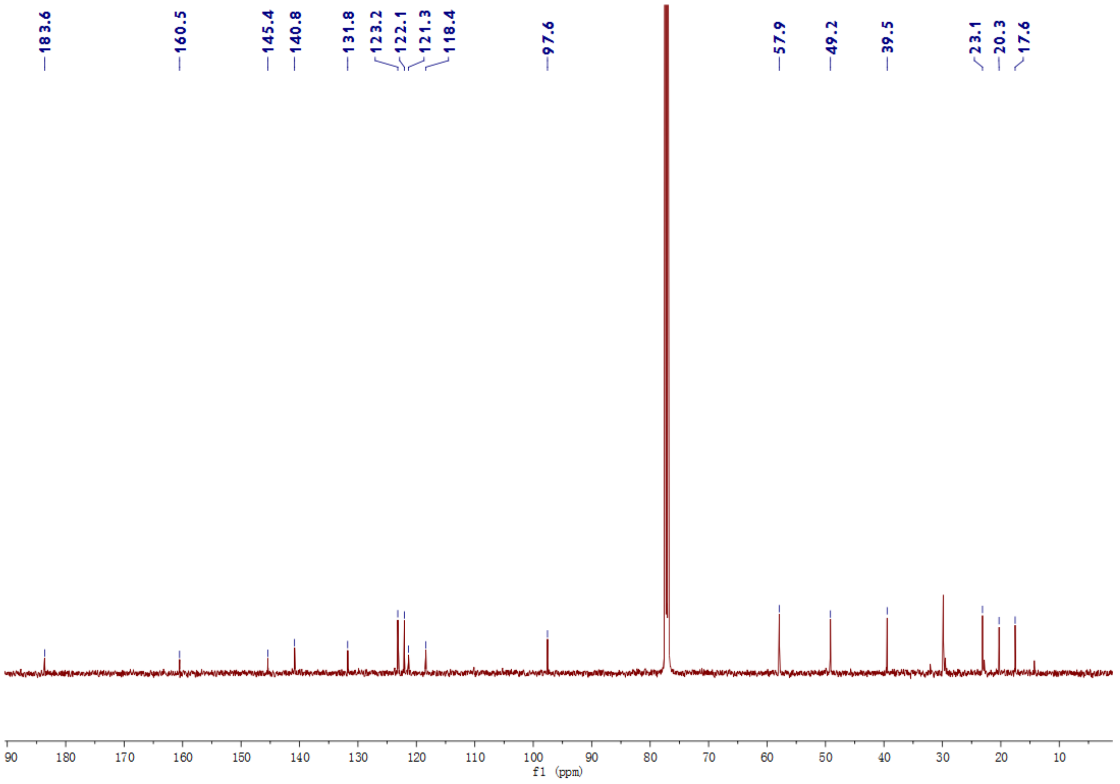

**Figure S31**. ${}^{1}H$-${}^{1}H$ COSY spectrum of **4** in $CDCl_3$

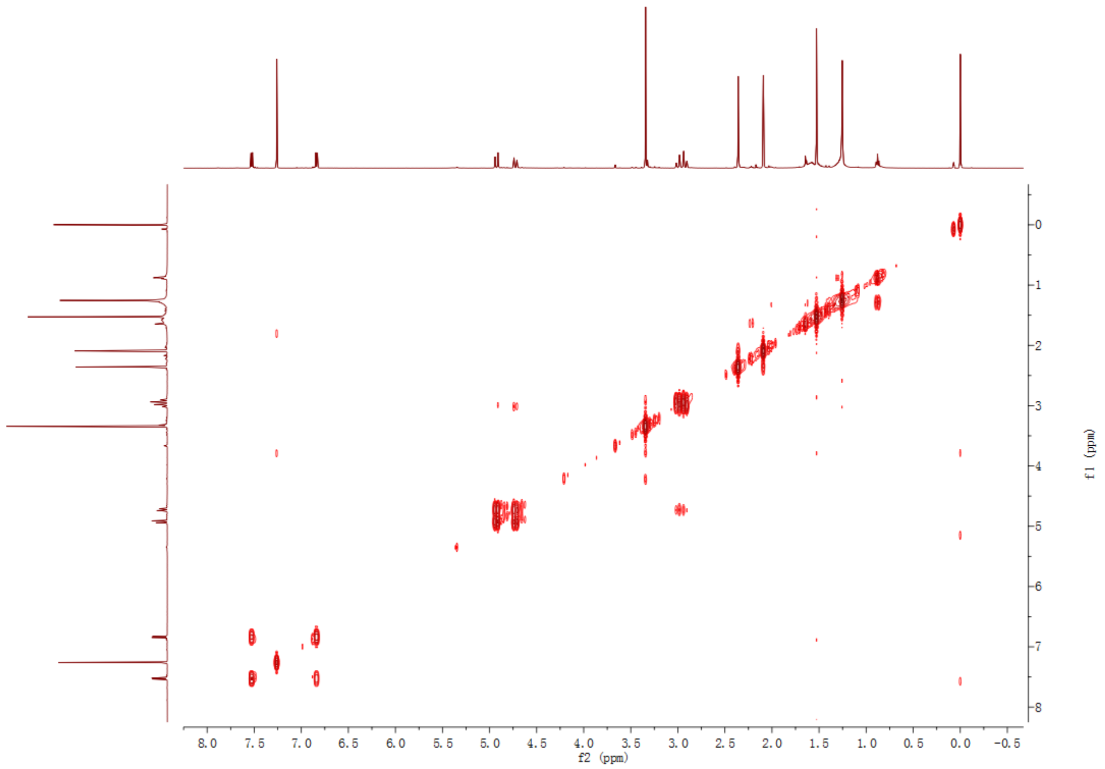


**Figure S32**. HSQC spectrum of **4** in $CDCl_3$

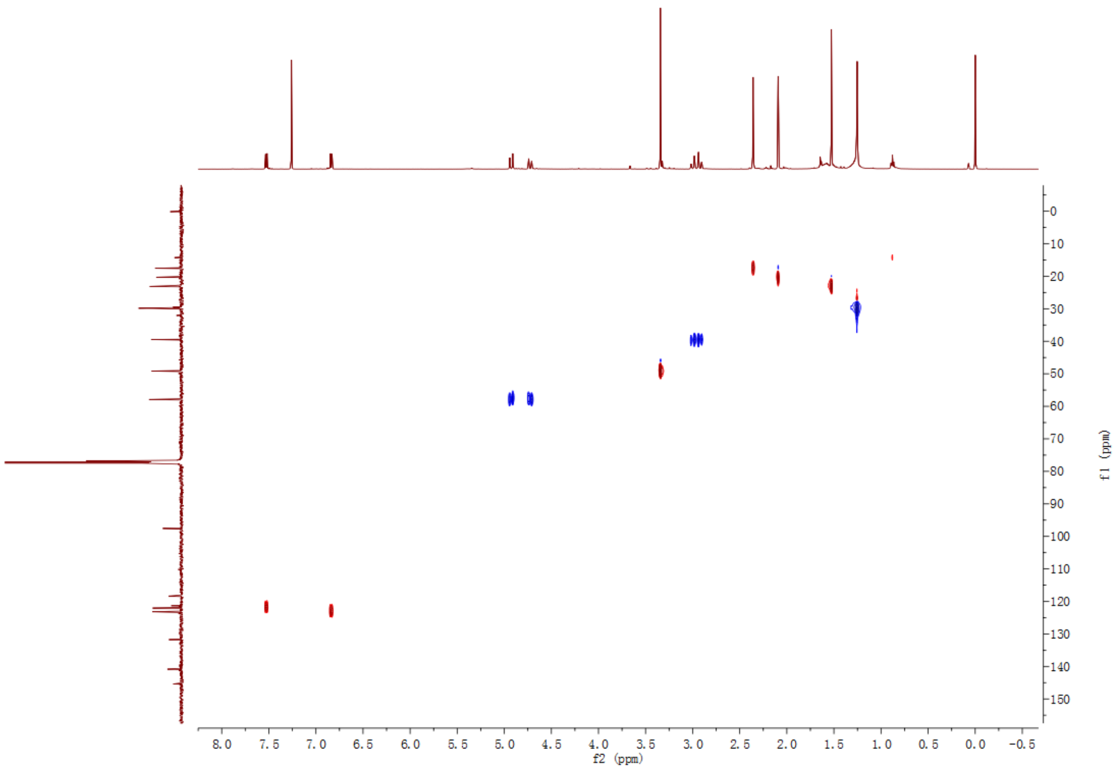

**Figure S33**. HMBC spectrum of **4** in $CDCl_3$

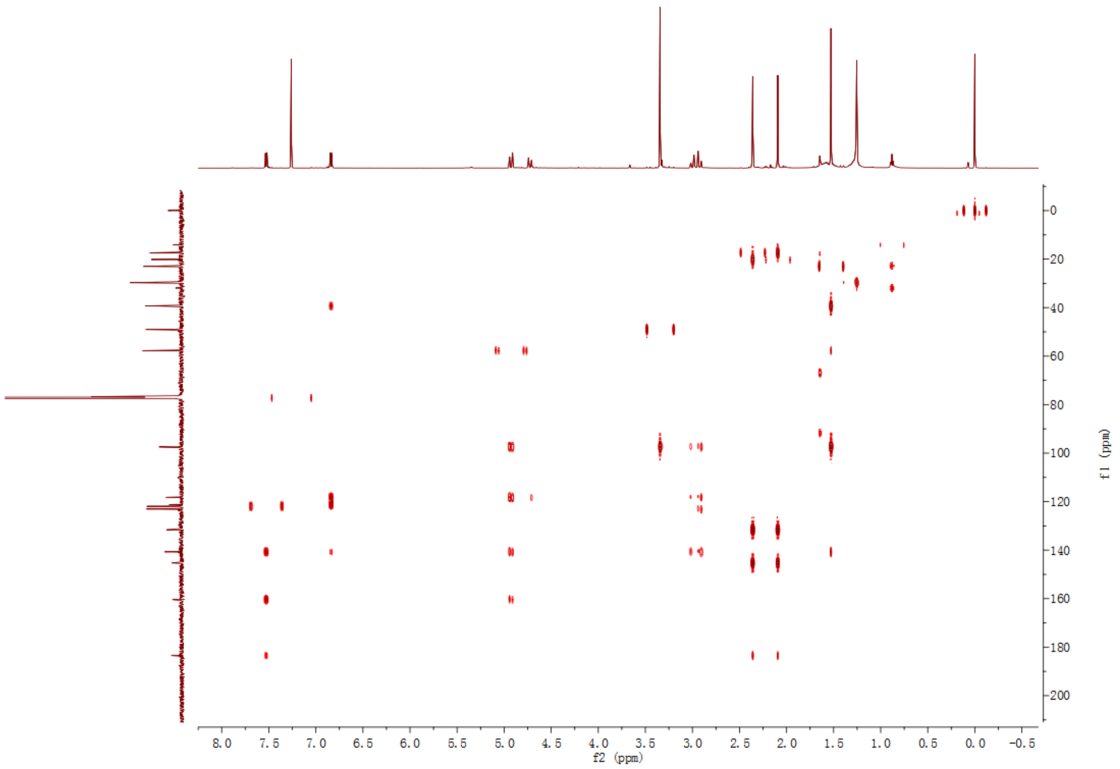


**Figure S34**. IR spectrum of **4**

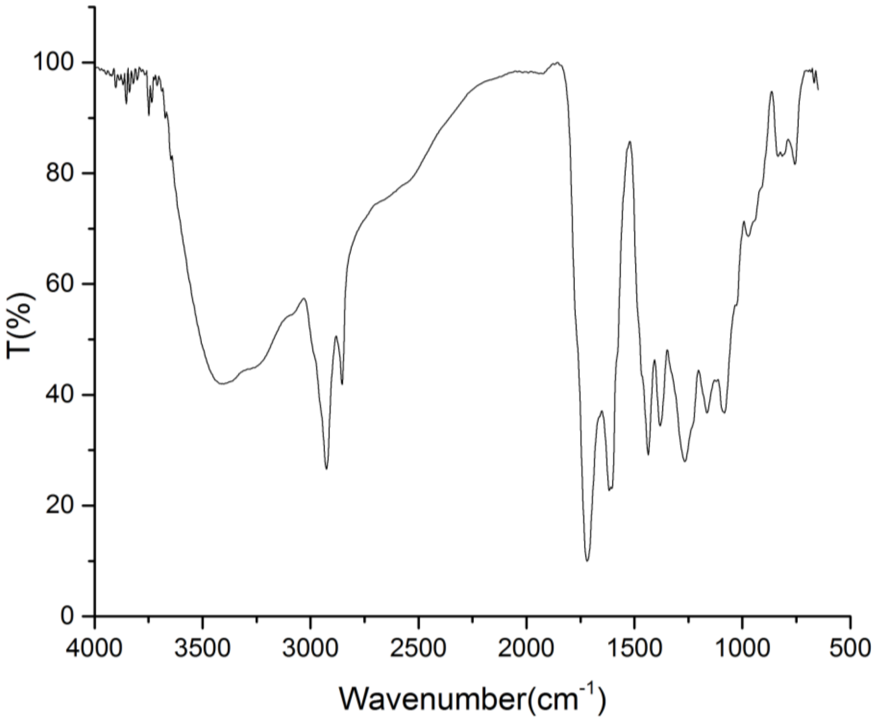

**Figure S35**. UV data of **4** in MeOH

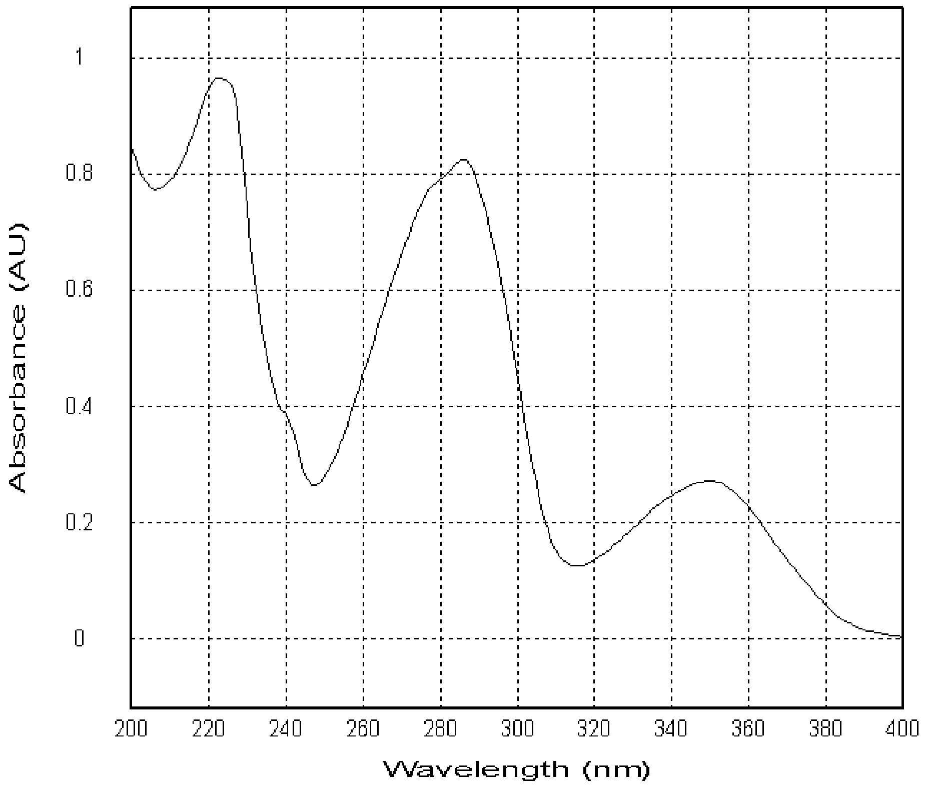


**Figure S36**. HRESIMS spectrum of **5**

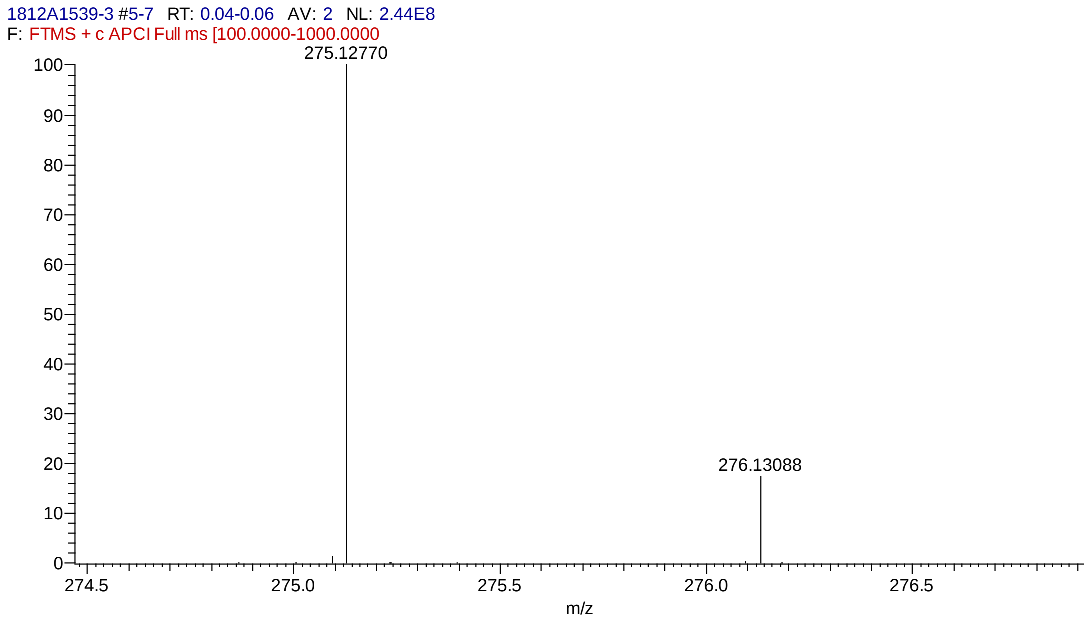


| m/z | Theo. Mass | Delta (mmu) | RDB equiv. | Composition |
|---|---|---|---|---|
| 275.12770 | 275.12779 | -0.09 | 7.5 | $C_{16}H_{19}O_4$ |

**Figure S37**. $^{1}$H NMR spectrum of **5** in $CDCl_3$

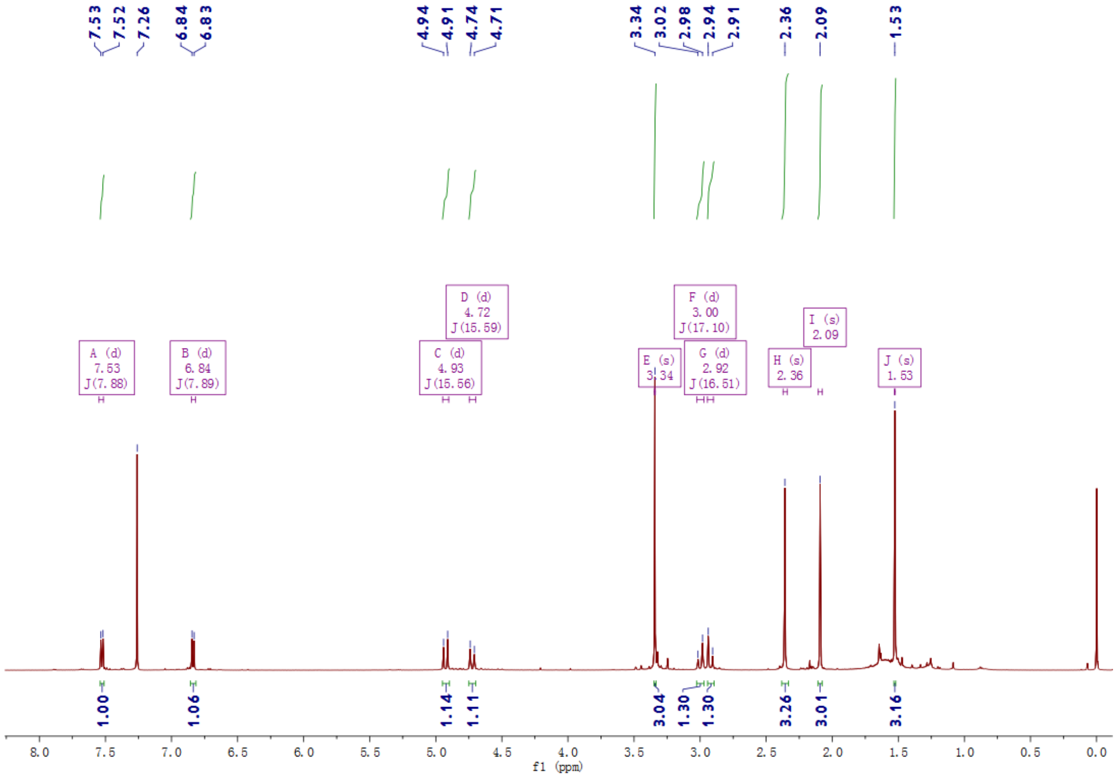

**Figure S38**. $^{13}$C NMR spectrum of **5** in $CDCl_3$

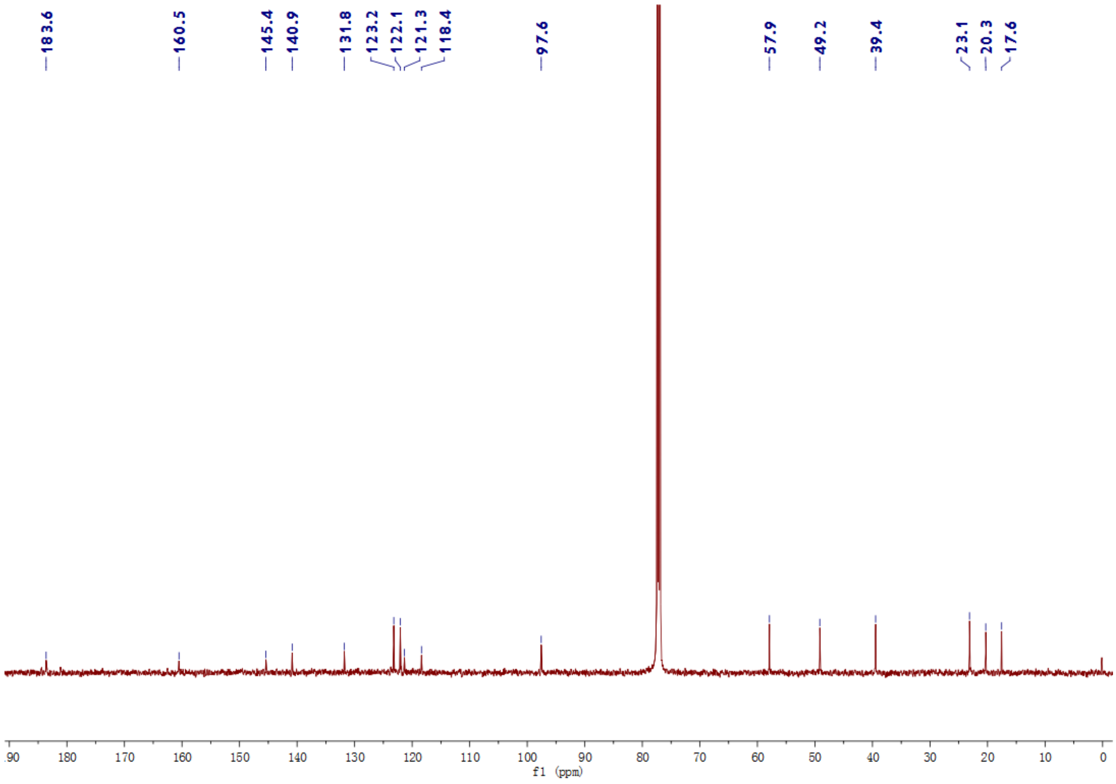

**Figure S39**. $^{1}H$-$^{1}H$ COSY spectrum of **5** in $CDCl_3$

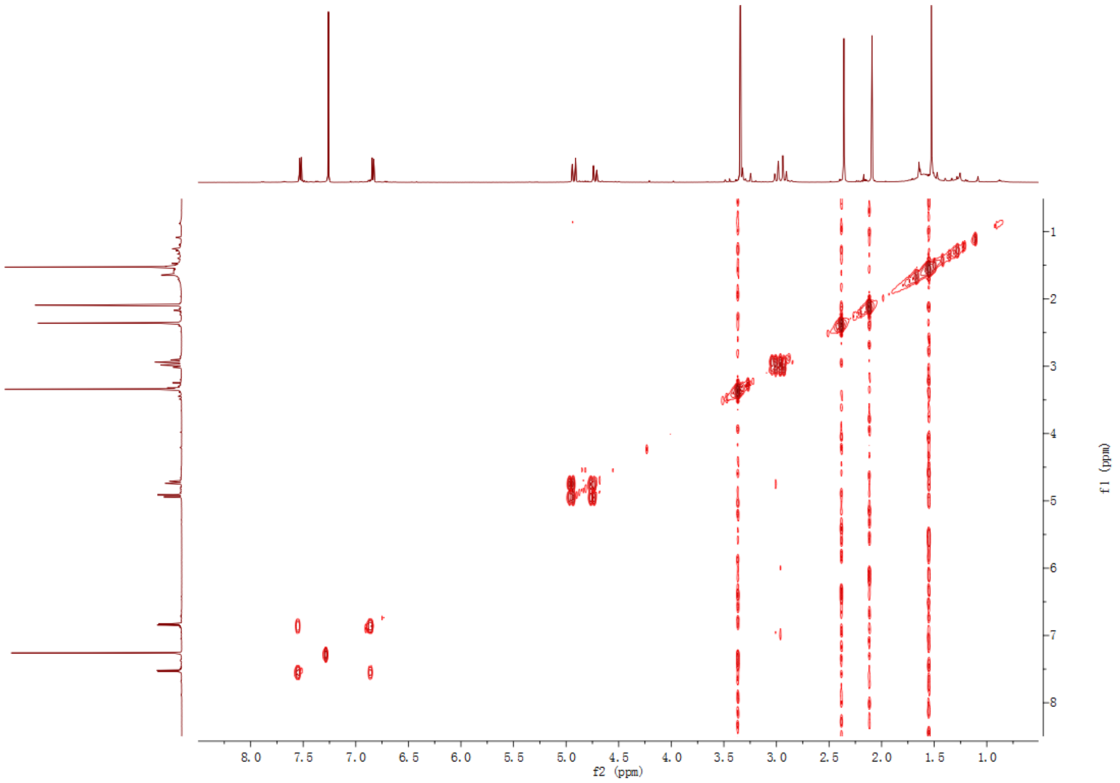


**Figure S40**. HSQC spectrum of **5** in $CDCl_3$

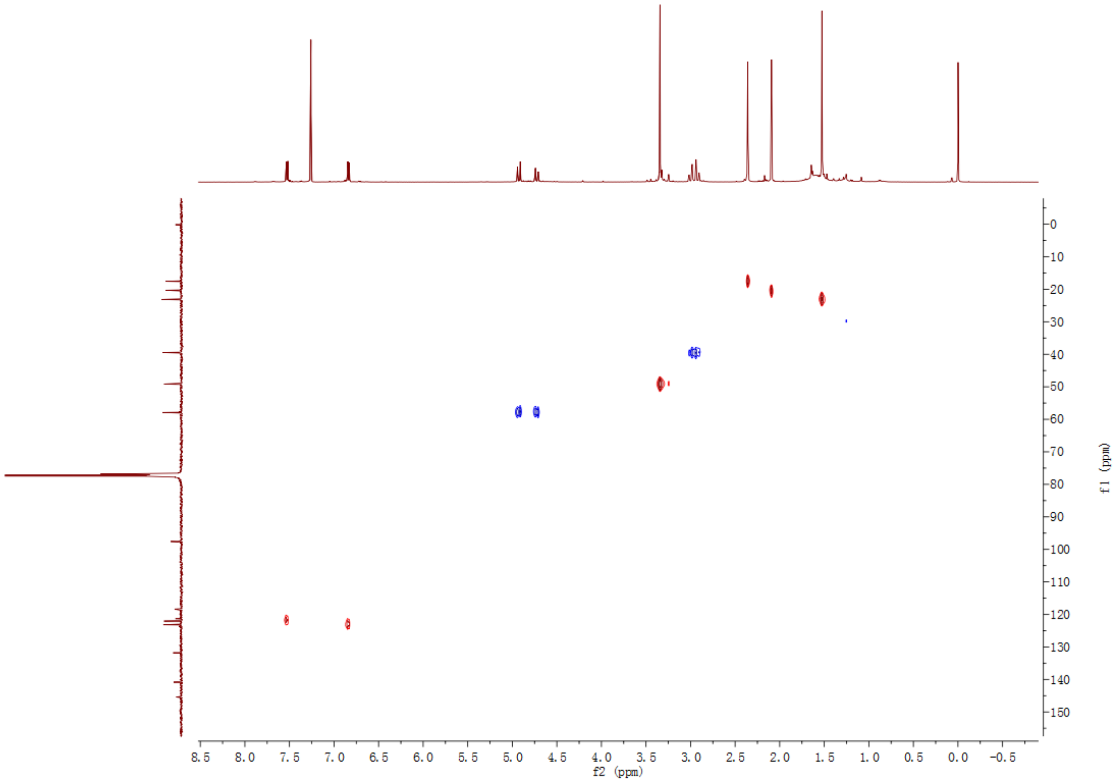

**Figure S41**. HMBC spectrum of **5** in $CDCl_3$

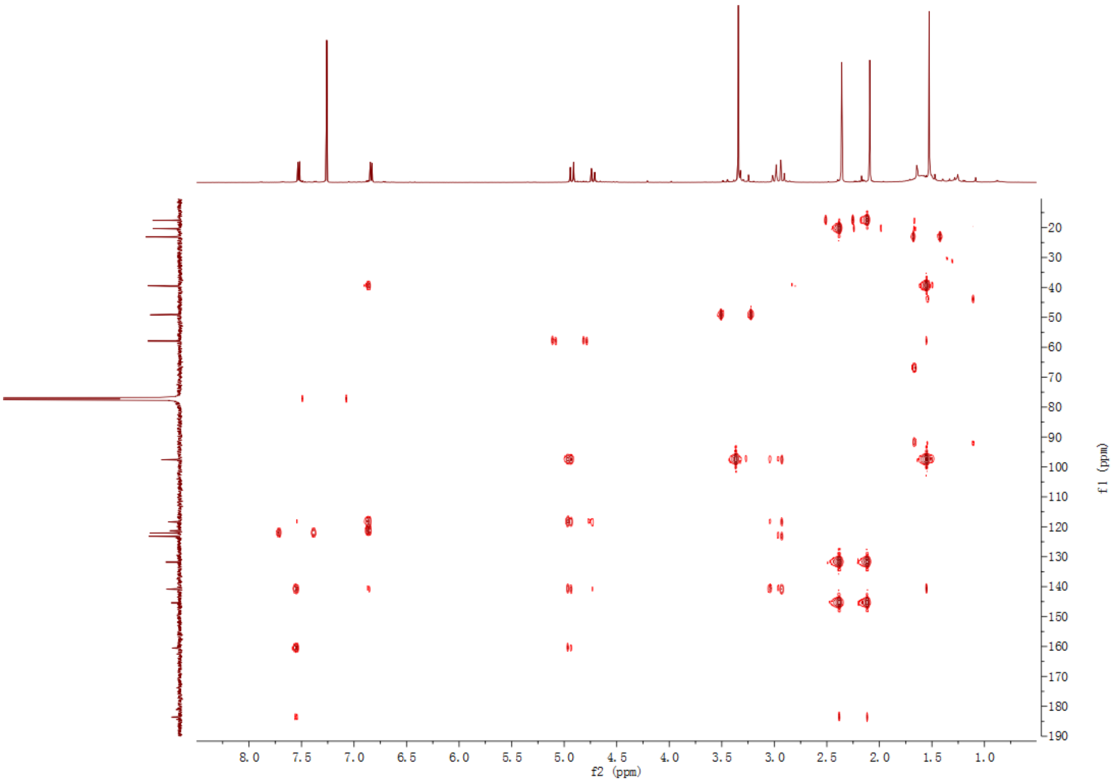


**Figure S42**. IR spectrum of **5**

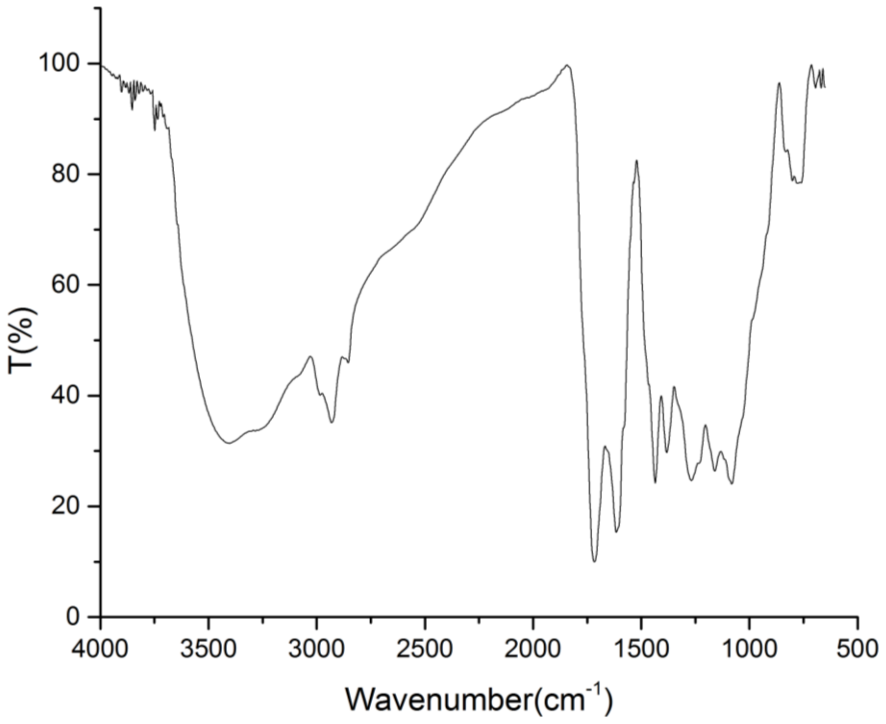

**Figure S43**. UV data of **5** in MeOH

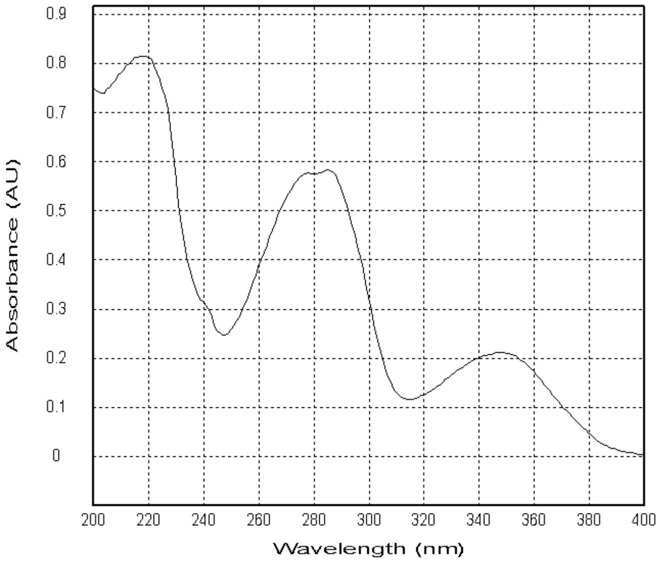


**Figure S44**. HRESIMS spectrum of **6**

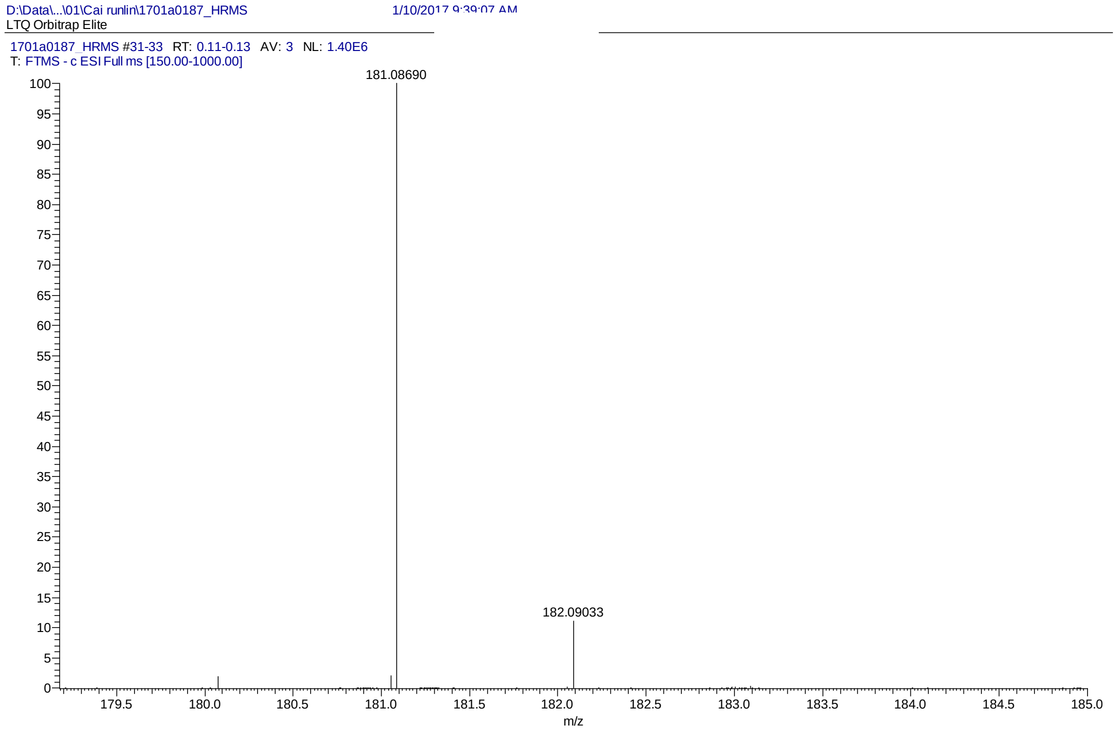


SPECTRUM - simulation :

| m/z | Theo. Mass | Delta (ppm) | RDB equiv. | Composition |
|---|---|---|---|---|
| 181.08690 | 181.08702 | -0.65 | 4.5 | $C_{10}H_{13}O_3$ |

**Figure S45**. $^1$H NMR spectrum of **6** in acetone-$d_6$

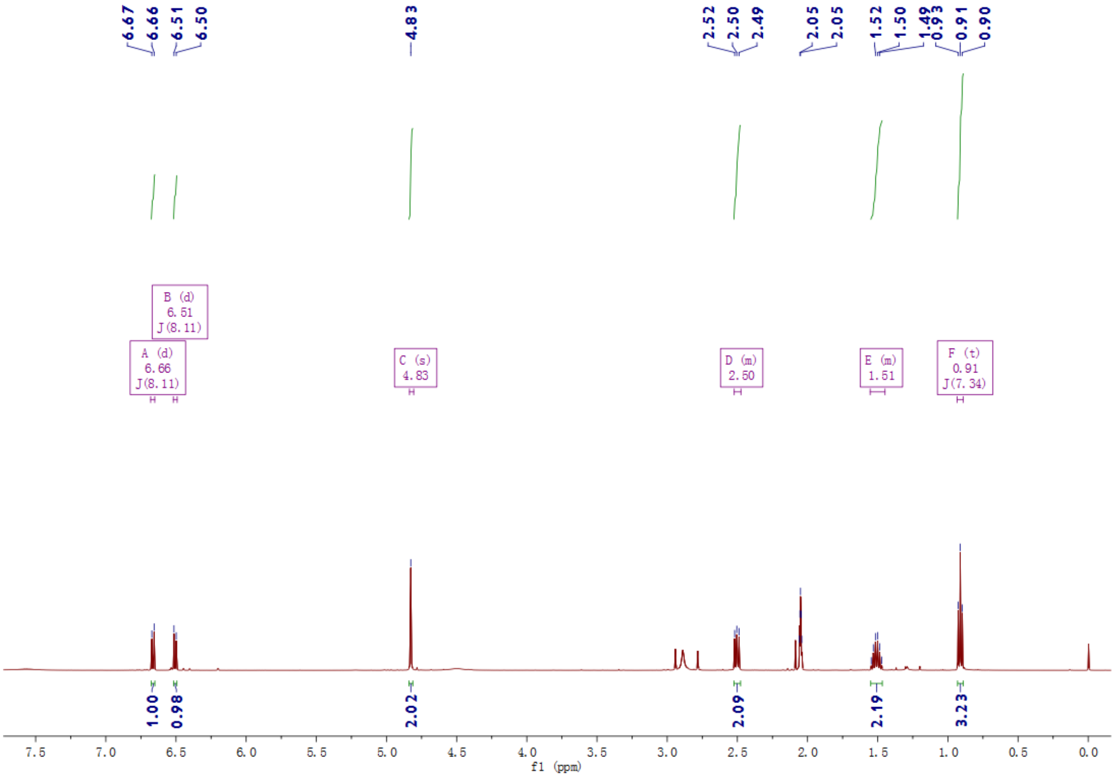


**Figure S46**. $^{13}$C NMR spectrum of **6** in acetone-$d_6$

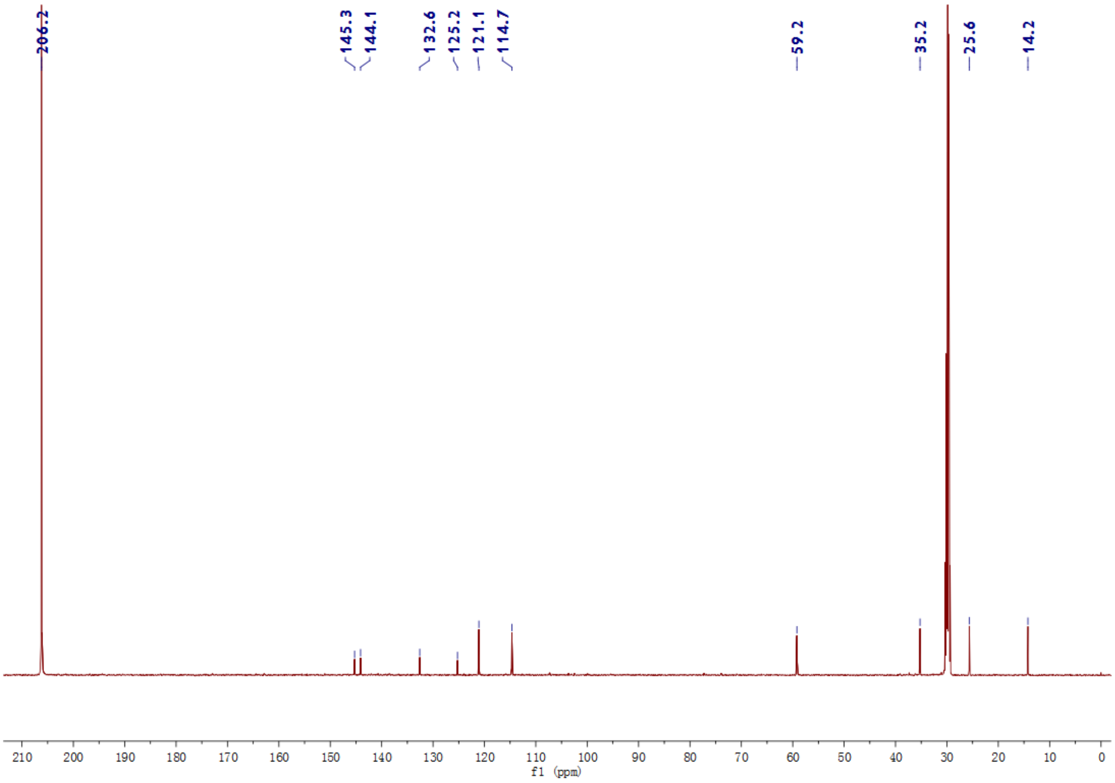

**Figure S47**. DEPT spectrum of **6** in acetone-$d_6$

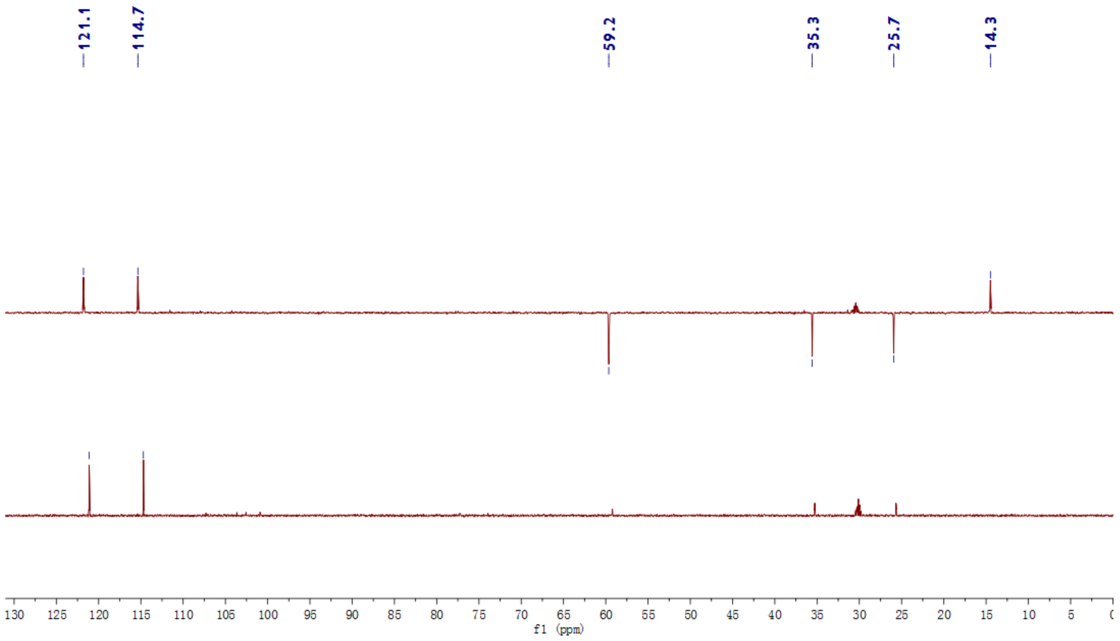


**Figure S48**. $^{1}$H-$^{1}$H COSY spectrum of **6** in acetone-$d_6$

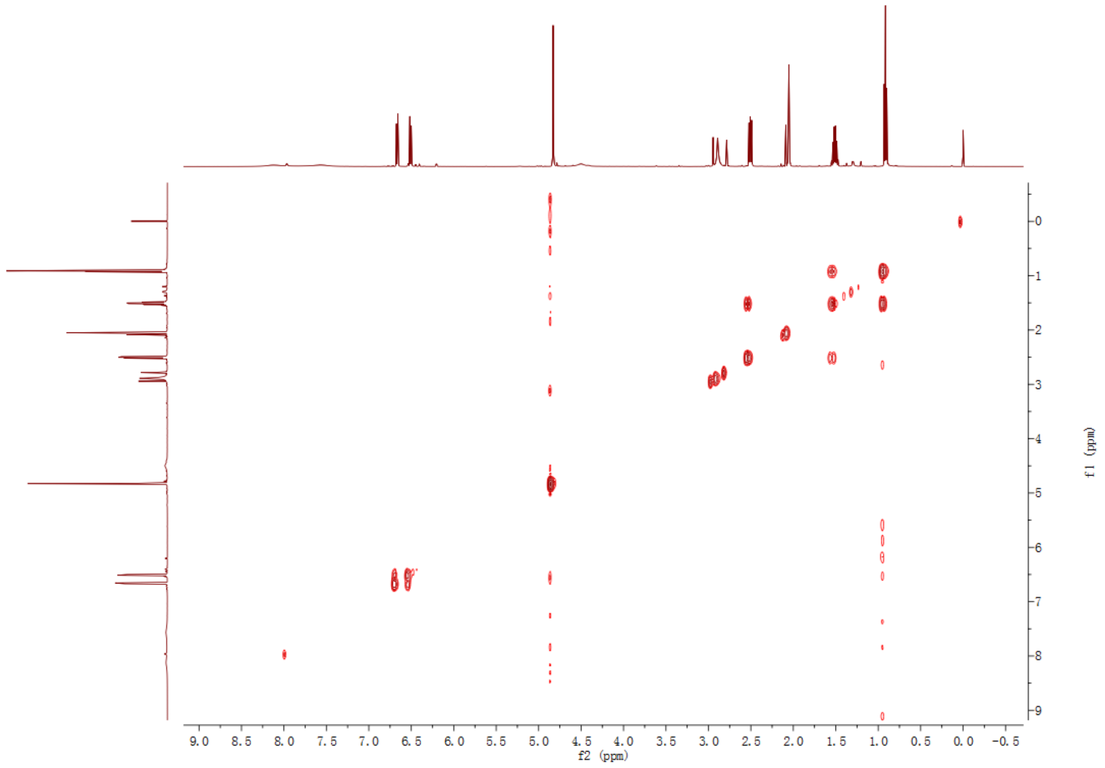

**Figure S49**. HSQC spectrum of **6** in acetone-$d_6$

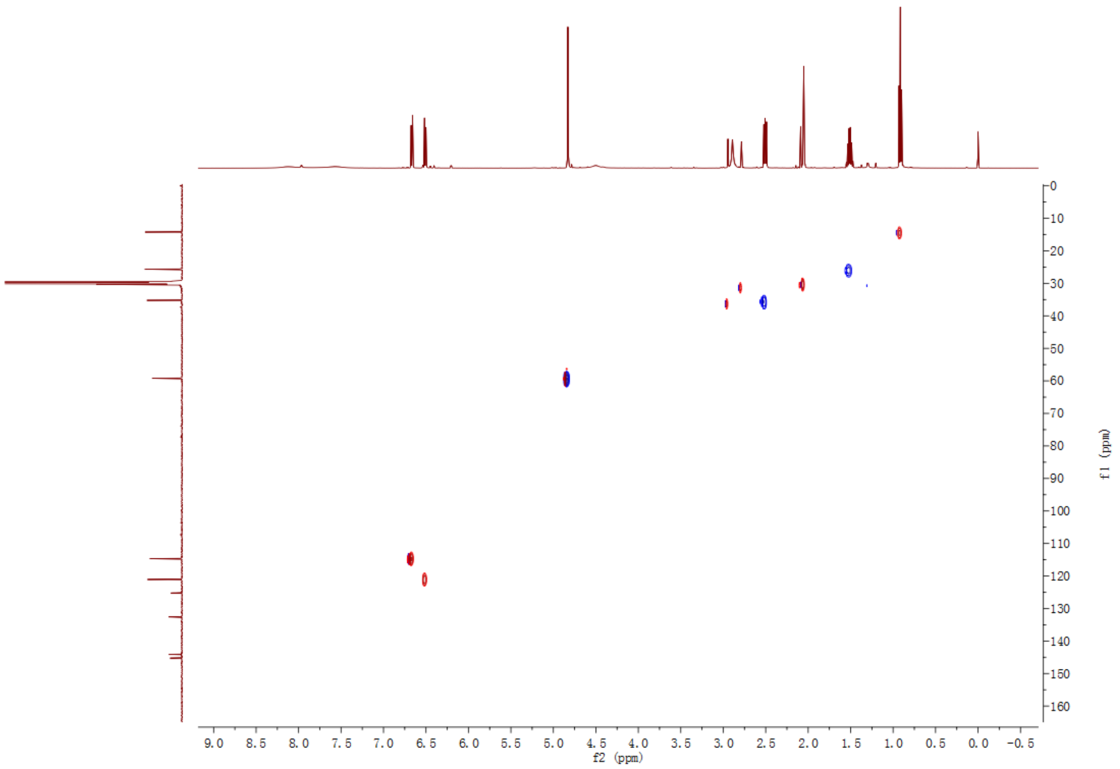


**Figure S50**. HMBC spectrum of **6** in acetone-$d_6$

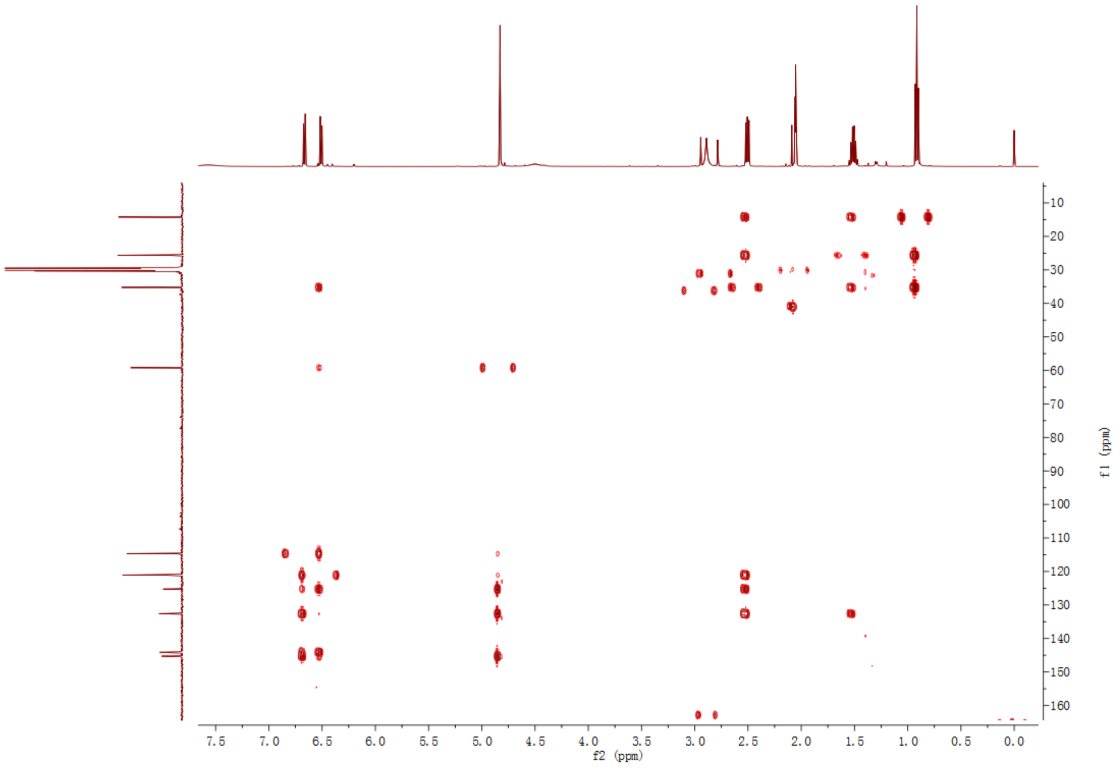

**Figure S51**. IR spectrum of **6**

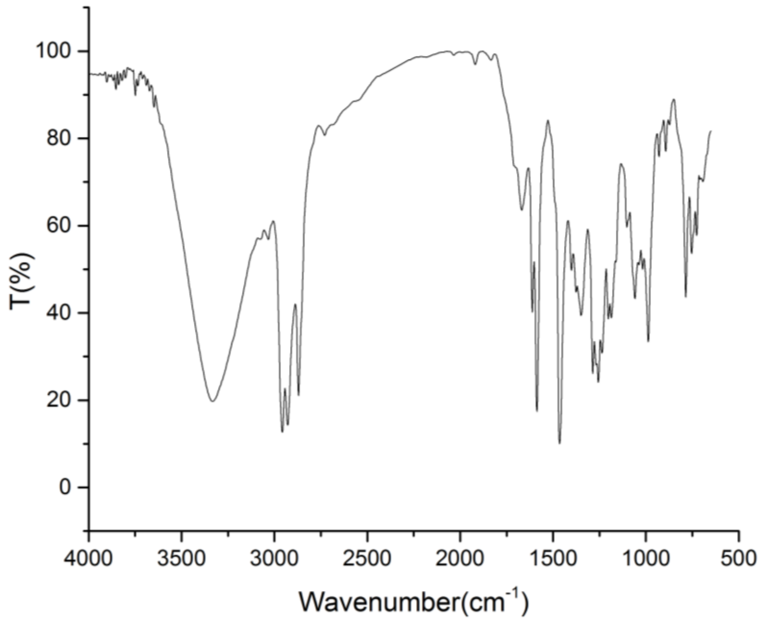


**Figure S52**. UV data of **6** in MeOH

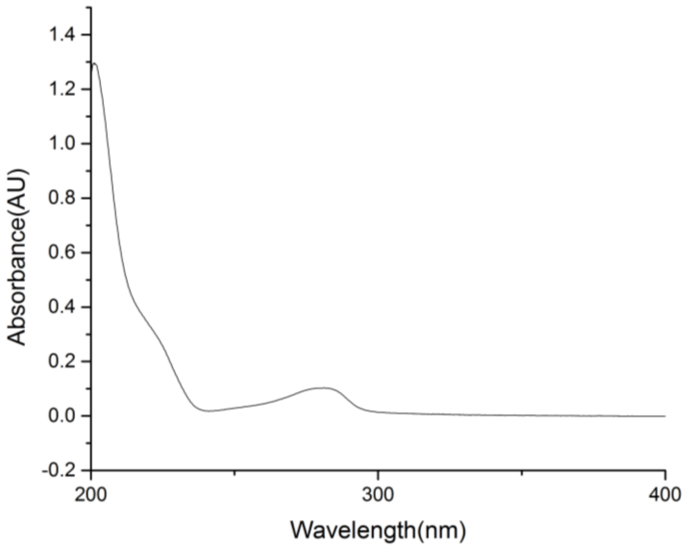

**Figure S53**. HRESIMS spectrum of **7**

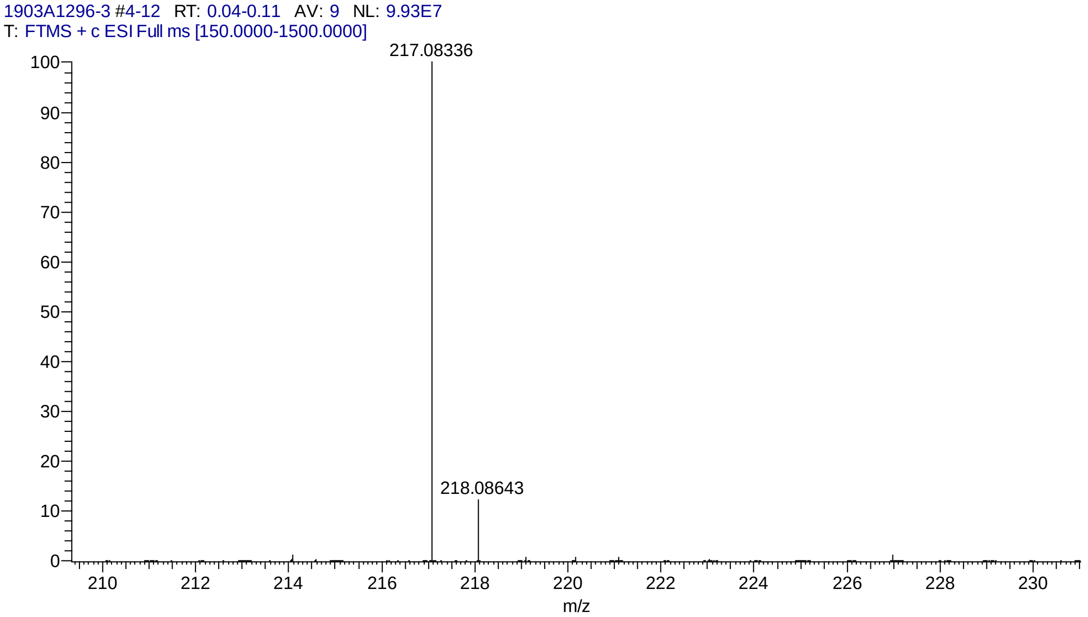


| m/z | Theo. Mass | Delta (mmu) | RDB equiv. | Composition |
|---|---|---|---|---|
| 217.08336 | 217.08352 | -0.16 | 4.5 | $C_{11}H_{14}O_3Na$ |

**Figure S54**. $^1$H NMR spectrum of **7** in methanol-$d_4$

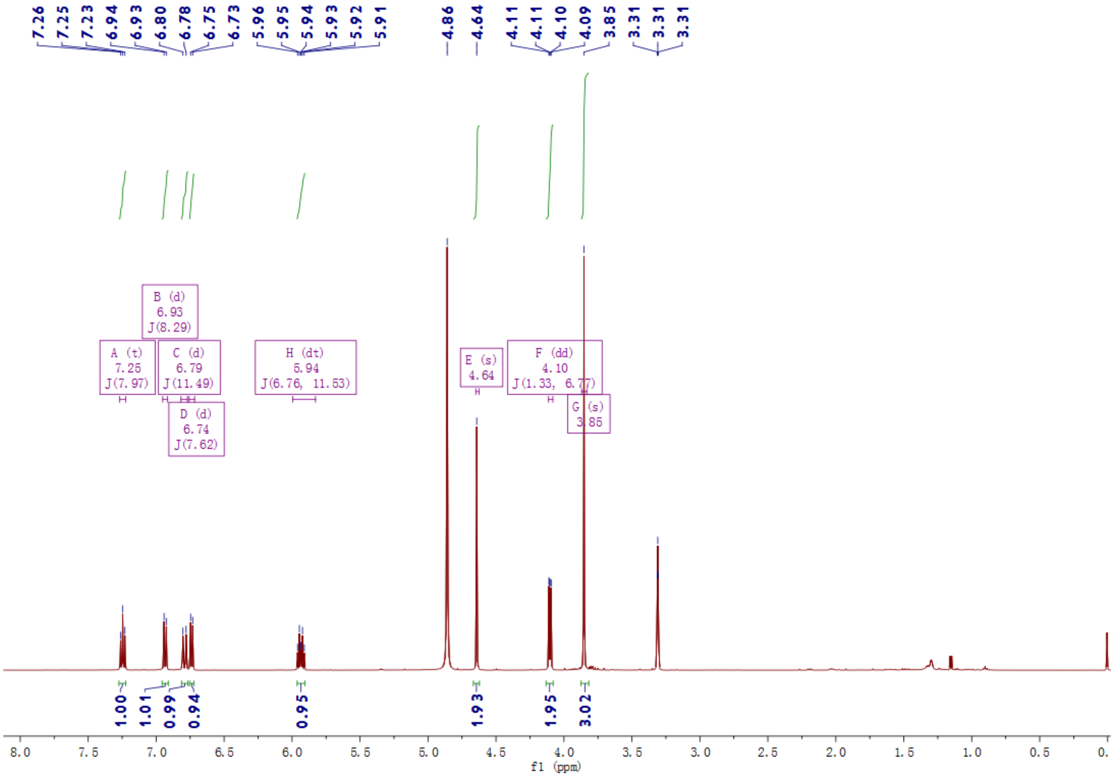

**Figure S55**. $^{13}$C NMR spectrum of **7** in methanol-$d_4$

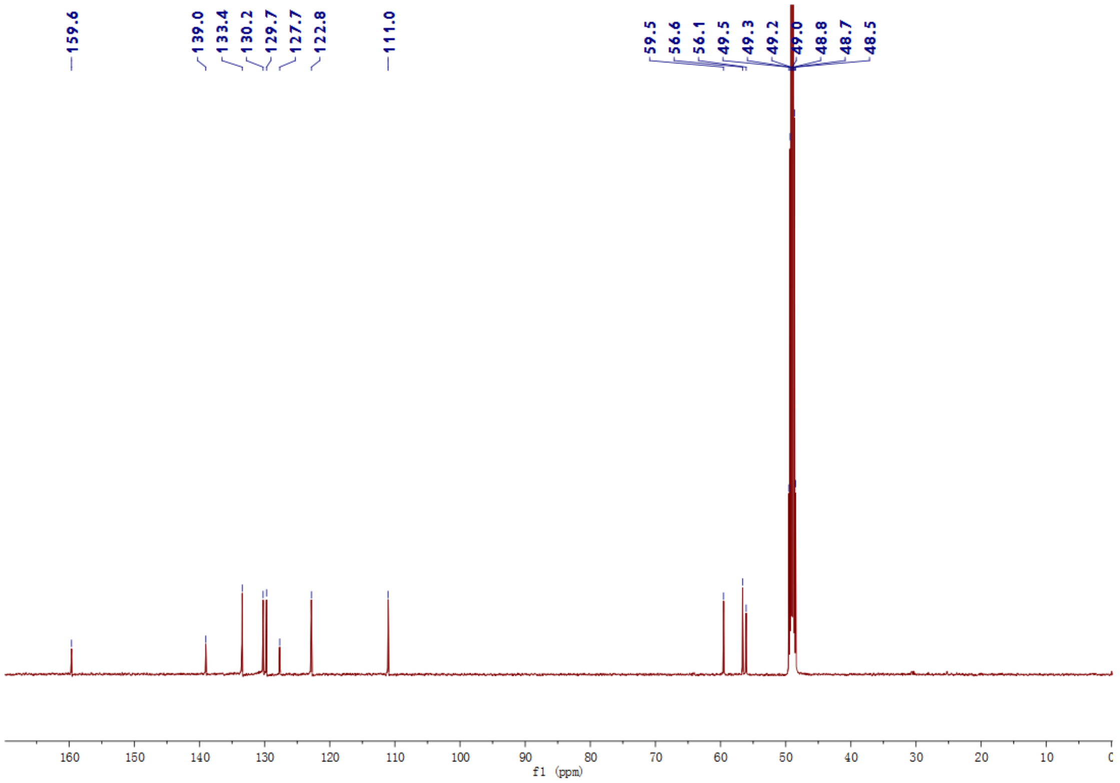


**Figure S56**. DEPT spectrum of **7** in methanol-$d_4$

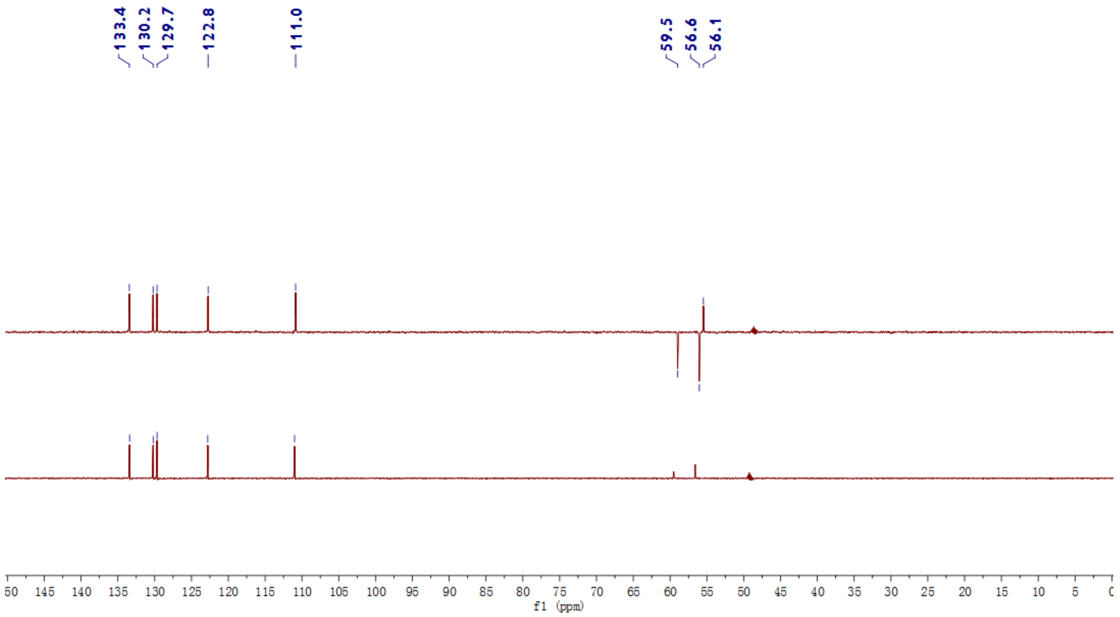

**Figure S57**. $^{1}H$-$^{1}H$ COSY spectrum of **7** in methanol-$d_4$

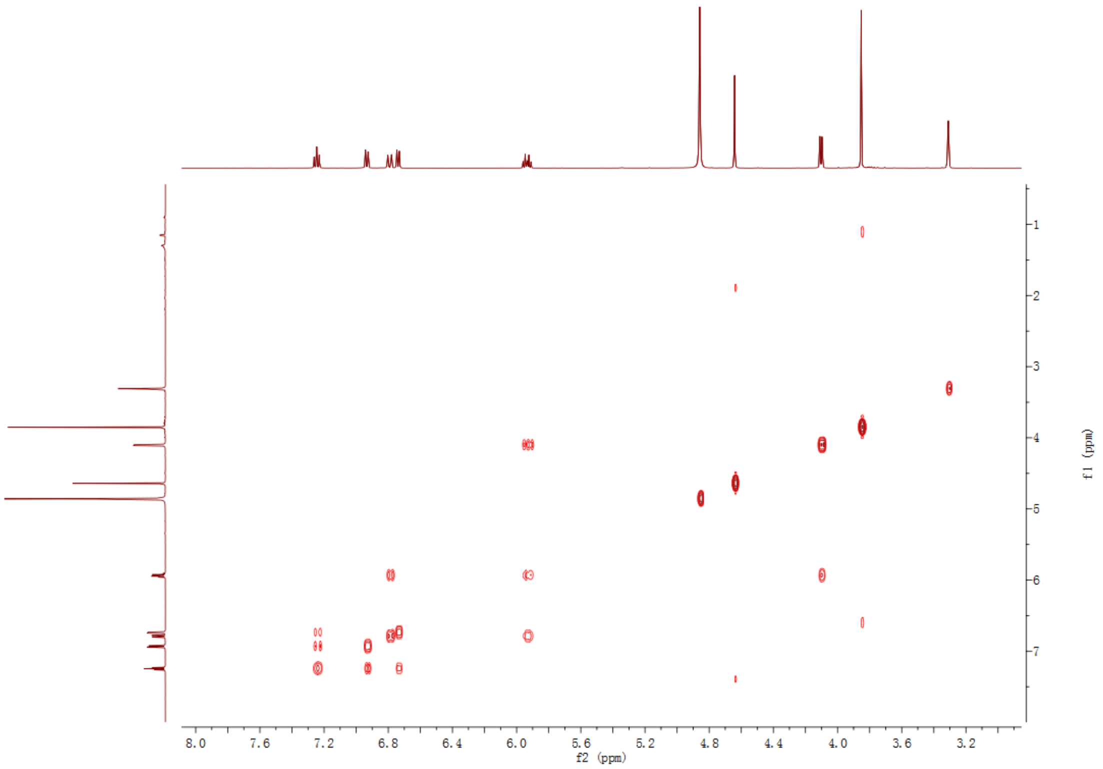


**Figure S58**. HSQC spectrum of **7** in methanol-$d_4$

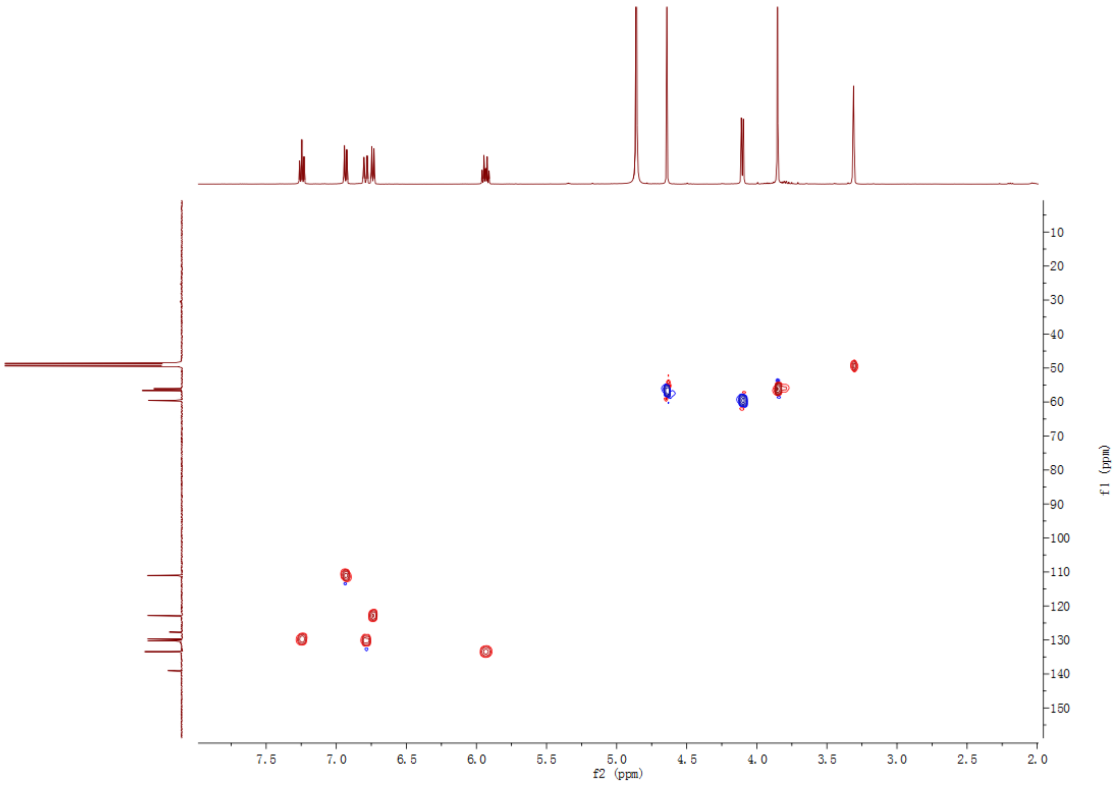

**Figure S59**. HMBC spectrum of **7** in methanol-$d_4$

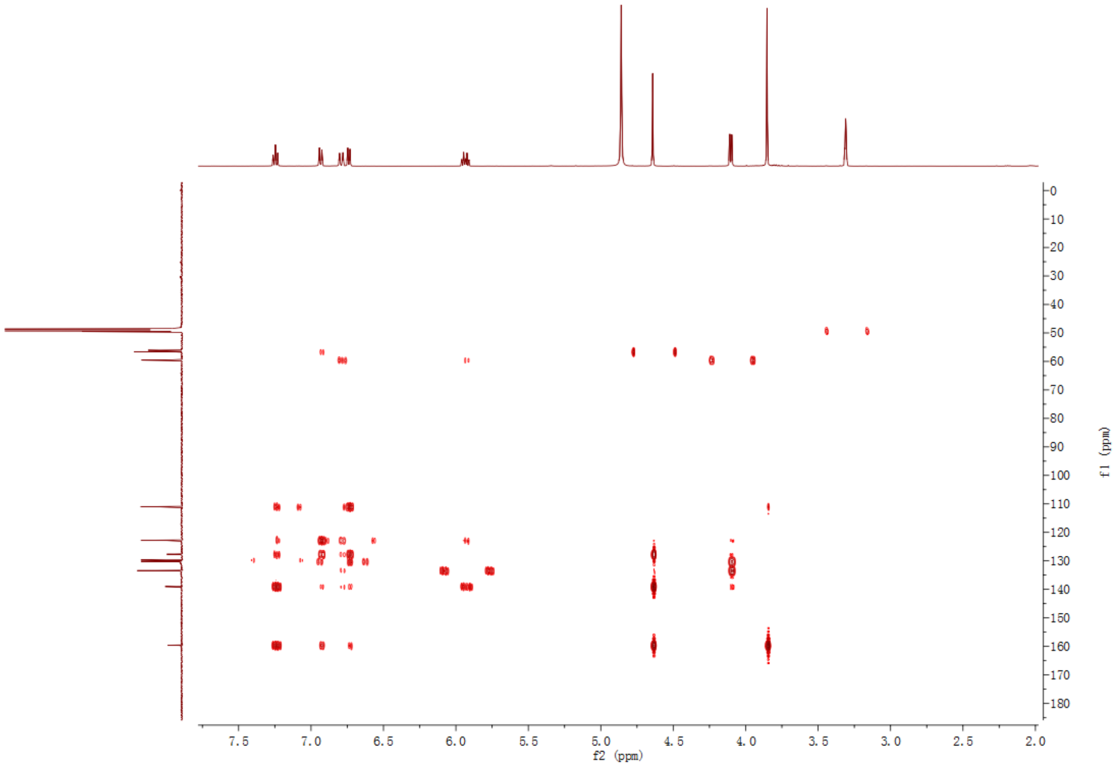


**Figure S60**. NOESY spectrum of 7 in methanol-$d_4$

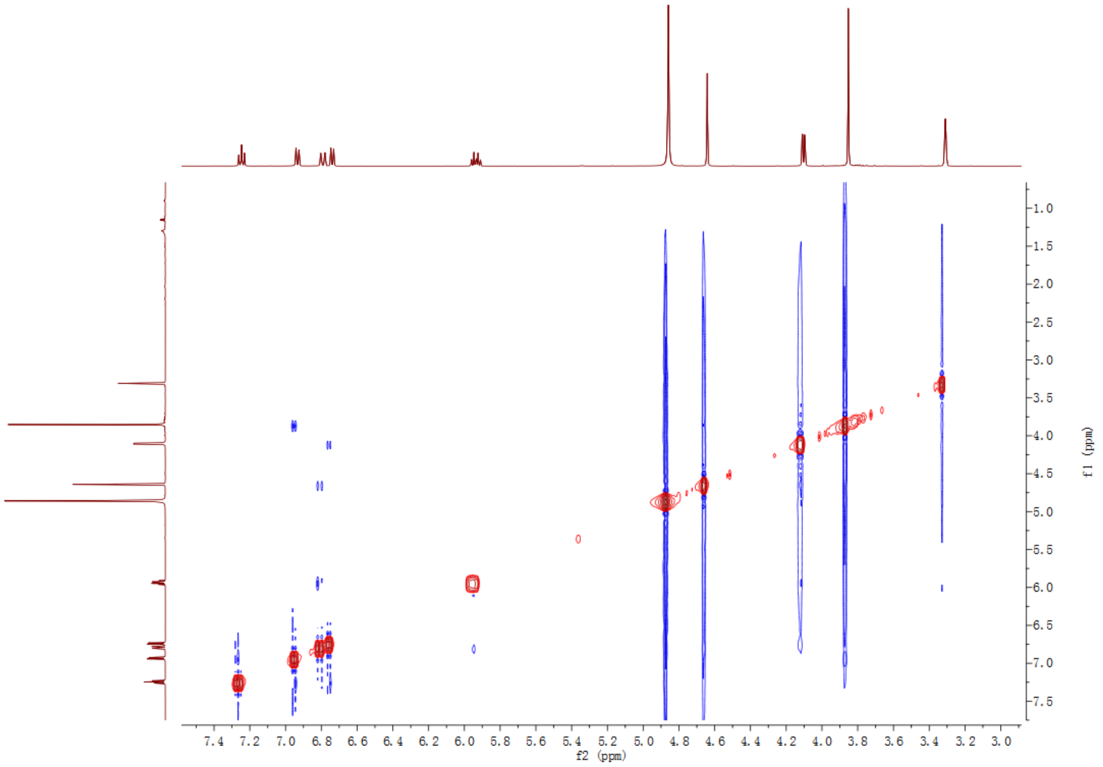

**Figure S61**. IR spectrum of **7**

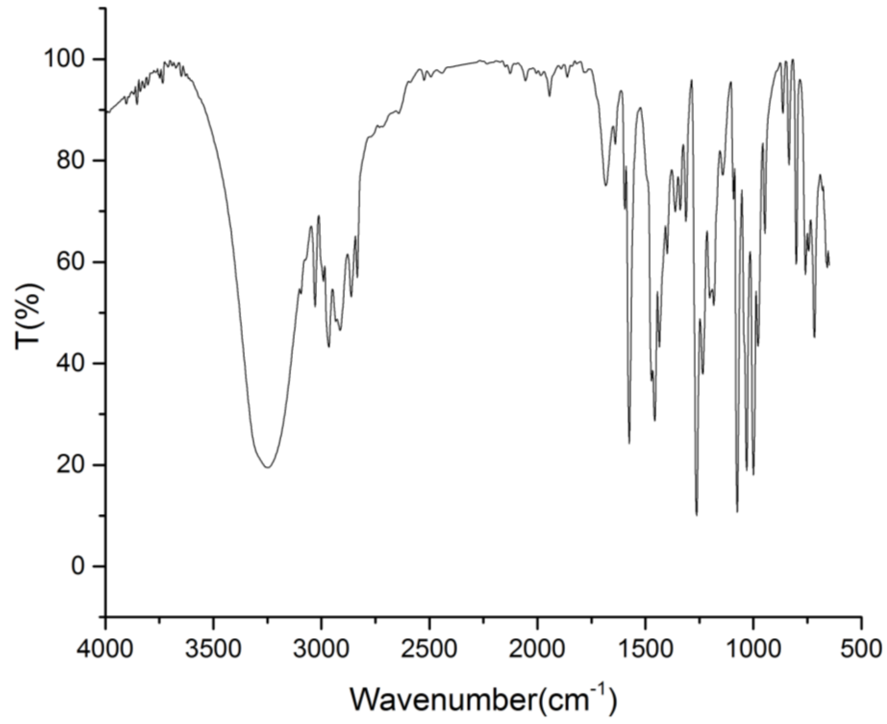


**Figure S62**. UV data of **7** in MeOH

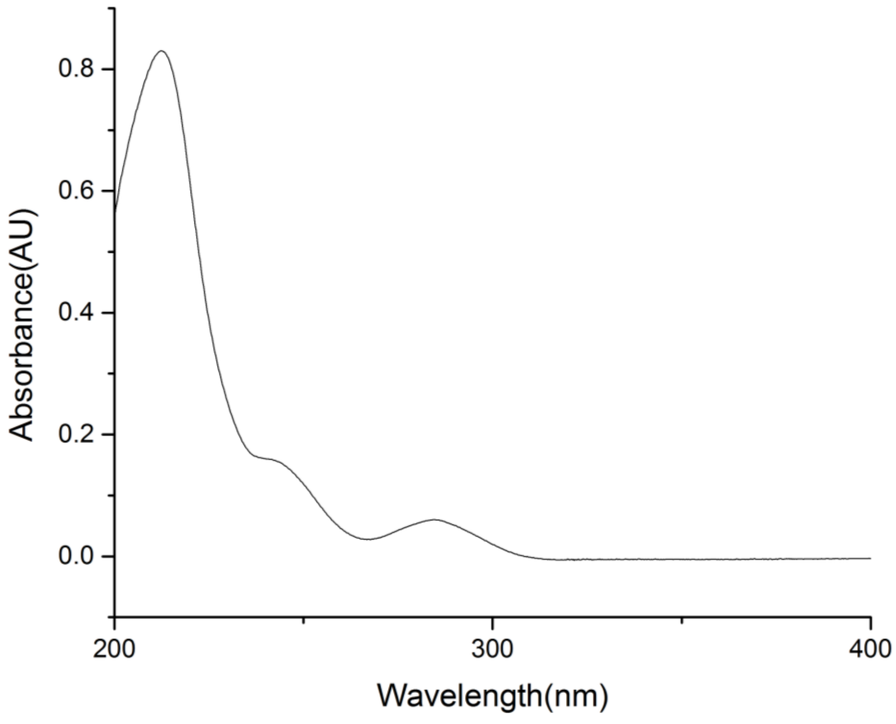

**Preparation of MTPA esters of 1 by the modified Mosher's method**

Compound **1** (4.1 mg) was reacted with (–)-$\alpha$-methoxy-$\alpha$-(trifluoromethyl) phenylacetyl chloride ((–)-MTPA-Cl, 20 $\mu$L) in anhydrous pyridine (400 $\mu$L) for 8 h at room temperature. Then the solvent from reaction mixture was removed under reduced pressure to furnish a residue, which was purified by preparative TLC (with $CH_2Cl_2$ eluant) to give (*S*)-MTPA ester (**1a**, 3.8 mg). Through a same procedure, (*R*)-MTPA ester (**1b**, 3.7 mg) was obtained from **1** (4.2 mg) using (+)-MTPA-Cl.

**(*S*)-MTPA ester for 1a:** $^1$H NMR ($CDCl_3$, 400 MHz) $\delta_H$ 7.04 (1H, d, *J* = 7.7 Hz, H-4), 6.60 (1H, d, *J* = 7.7 Hz, H-5), 5.81 (1H, dd, *J* = 5.1, 8.7 Hz, H-6), 5.63 (1H, d, *J* = 12.0 Hz, H-9a), 5.33 (1H, d, *J* = 12.0 Hz, H-9b), 4.60 (1H, t, *J* = 9.1 Hz, H-2), 3.22 (1H, dd, *J* = 8.9, 16.6 Hz, H-3a), 3.11 (1H, dd, *J* = 9.4, 16.6 Hz, H-3b), 1.64 (2H, m, H-7), 1.33 (3H, s, H-13), 1.19 (3H, s, H-12), 0.75 (3H, t, *J* = 7.3 Hz, H-10); ESIMS *m*/*z* 716.05 $[M+NH_4^+]^+$.

**(*R*)-MTPA ester for 1b:** $^1$H NMR ($CDCl_3$, 400 MHz) $\delta_H$ 7.14 (1H, d, *J* = 7.7 Hz, H-4), 6.85 (1H, d, *J* = 7.7 Hz, H-5), 6.00 (1H, dd, *J* = 4.9, 8.8 Hz, H-6), 5.66 (1H, d, *J* = 12.0 Hz, H-9a), 5.48 (1H, d, *J* = 12.0 Hz, H-9b), 4.62 (1H, t, *J* = 9.1 Hz, H-2), 3.22 (1H, dd, *J* = 8.3, 16.3 Hz, H-3a), 3.13 (1H, dd, *J* = 9.4, 16.3 Hz, H-3b), 1.55 (2H, m, H-7), 1.35 (3H, s, H-13), 1.19 (3H, s, H-12), 0.72 (3H, t, *J* = 7.3 Hz, H-10); ESIMS *m*/*z* 716.05 $[M+NH_4^+]^+$.

**Figure S63**. ESIMS spectrum of (*S*)-MTPA ester (**1a**)

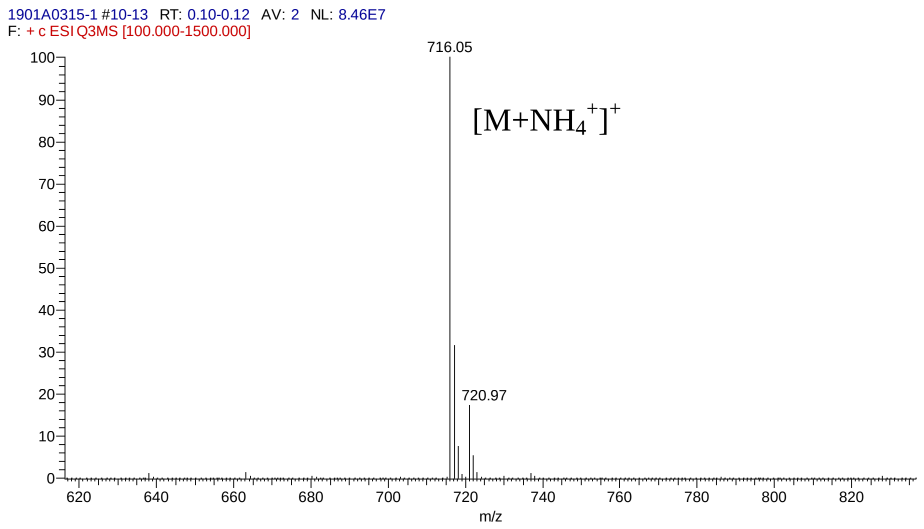

**Figure S64**. ESIMS spectrum of (*R*)-MTPA ester (**1b**)

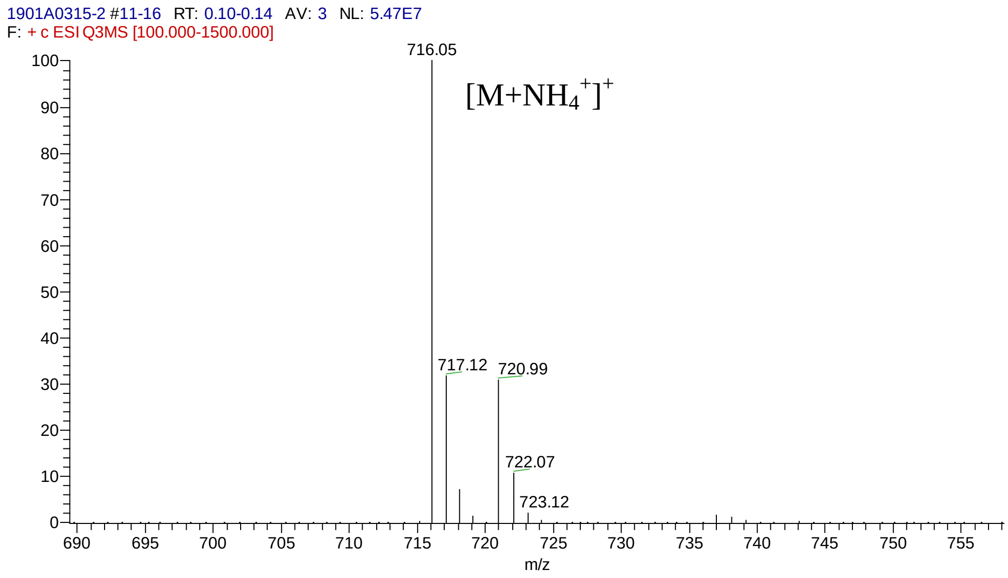


**Figure S65**. $^{1}$H NMR spectrum of (*S*)-MTPA ester (**1a**) in $CDCl_3$

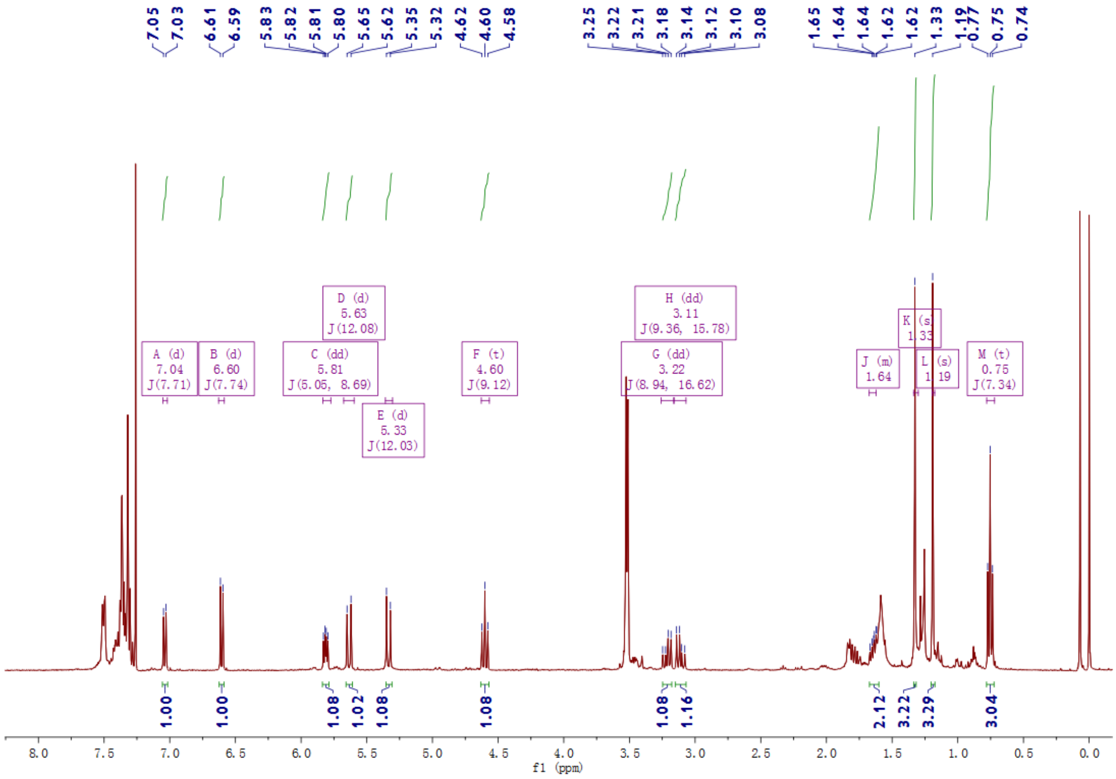

**Figure S66**. $^1$H NMR spectrum of (*R*)-MTPA ester (**1b**) in $CDCl_3$

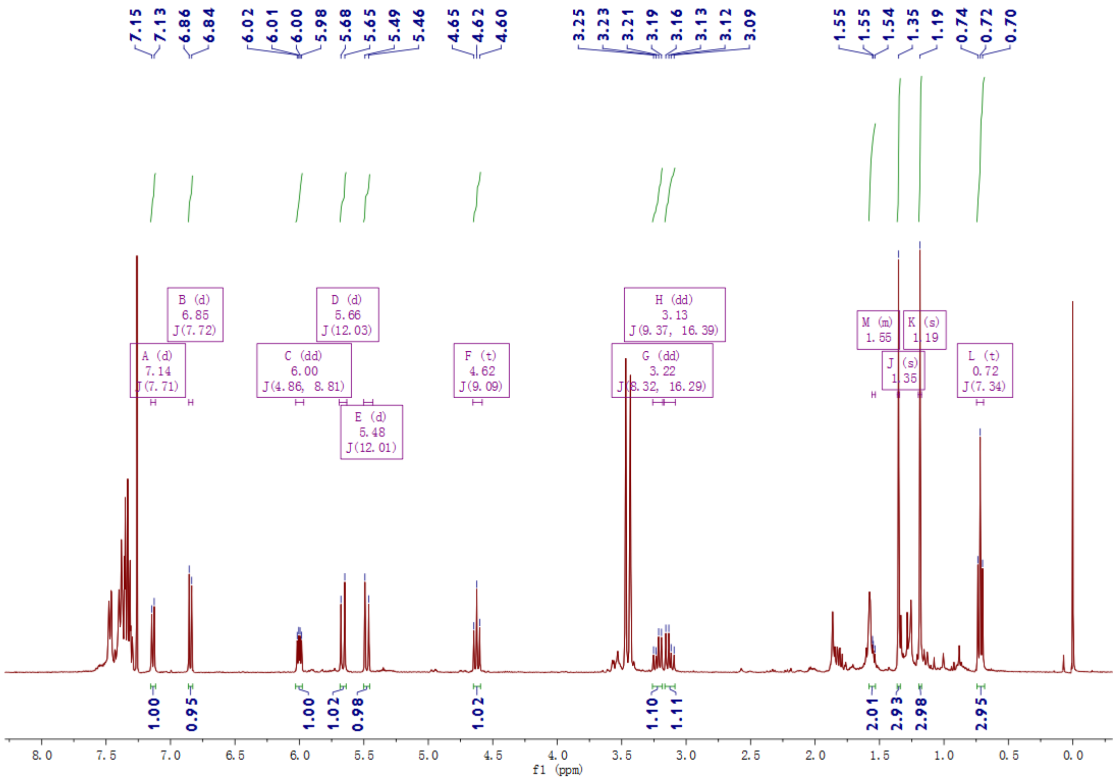

**The $^{1}$H and $^{13}$C NMR data and HRMS data of compounds 8-14.**

2-(Hydroxymethyl)-3-propylphenol (**8**): colorless oil; $^{1}$H NMR (400 MHz, $CDCl_3$): $\delta_H$ 7.11(1H, t, *J* = 7.9, H-2), 6.73 (1H, d, *J* = 7.9, H-1), 6.71 (1H, d, *J* =7.9, H-3), 4.90 (2H, s, H-10), 2.55 (2H, t, *J* = 7.7, H-7), 1.54 (2H, m, H-8), 0.94 (3H, t, *J* = 7.3, H-9); $^{13}$C NMR (101 MHz, $CDCl_3$): $\delta_C$ 156.6 (C-6), 141.1 (C-4), 128.9 (C-2), 123.0 (C-5), 121.8 (C-3), 114.5 (C-1), 60.0 (C-10), 35.4 (C-7), 24.9 (C-8), 14.1 (C-9); HRESIMS *m/z* 167.1076 $[M+H]^+$ (calcd for $C_{10}H_{15}O_2$, 167.1072).

Peniciphenol (**9**): colorless oil; $^{1}$H NMR (400 MHz, $CD_3OD$) $\delta_H$ 7.08 (1H, t, *J* = 7.8 Hz, H-2), 6.75 (1H, d, *J* = 7.8 Hz, H-1), 6.74 (1H, d, *J* = 11.6 Hz, H-7), 6.61 (1H, d, *J* = 7.8 Hz, H-3), 5.90 (1H, dt, *J* = 11.6, 6.7 Hz, H-8), 4.68 (2H, s, H-10), 4.12 (2H, dd, *J* = 6.7, 1.4 Hz, H-9); $^{13}$C NMR (101 MHz, $CD_3OD$) $\delta_C$ 156.3 (C-6), 136.3 (C-4), 131.9 (C-8), 130.0 (C-7), 128.5 (C-2), 123.6 (C-5), 120.9 (C-3), 115.3 (C-1), 59.1 (C-9), 58.6 (C-10); HRESIMS *m/z* 181.0852 $[M+H]^+$ (calcd for $C_{10}H_{13}O_3$, 181.0865).

Penicibenzoxepinol (**10**): colorless oil; $^{1}$H NMR (400 MHz, $CDCl_3$) $\delta_H$ 7.05 (1H, t, *J* = 7.8 Hz, H-2), 6.82 (1H, d, *J* = 7.8 Hz, H-1), 6.59 (1H, d, *J* = 7.8 Hz, H-3), 6.47 (1H, dt, *J* = 12.4, 2.1 Hz, H-7), 5.86 (1H, dt, *J* = 12.4, 3.0 Hz, H-8), 4.85 (2H, s, H-10), 4.56 (2H, t, *J* = 2.6 Hz, H-9); $^{13}$C NMR (101 MHz, $CDCl_3$) $\delta_C$ 153.4 (C-6), 138.1 (C-4), 133.2 (C-8), 128.7 (C-7), 127.7 (C-2), 126.3 (C-5), 122.3 (C-3), 113.8 (C-1), 72.8 (C-9), 65.0 (C-10); HRESIMS *m/z* 163.0749 $[M + H]^+$ (calcd for $C_{10}H_{11}O_2$, 163.0759).

(−)-Brassicadiol (**11**): colorless oil; $^{1}$H NMR (400 MHz, $CDCl_3$) $\delta_H$ 6.98 (1H, d, *J* = 7.5 Hz, H-4), 6.65 (1H, d, *J* = 7.5 Hz, H-5), 4.70 (1H, d, *J* = 12.0 Hz, H-9), 4.64 (1H, d, *J* = 12.0 Hz, H-9), 4.58 (1H, t, *J* = 9.1 Hz, H-2), 3.09 (2H, m, H-3), 2.60, (2H, m, H-6), 1.56 (2H, m, H-7), 1.32 (3H, s, H-13), 1.16 (3H, s, H-12), 0.95 (3H, t, *J* = 7.3 Hz, H-10); $^{13}$C NMR (101 MHz, $CDCl_3$) $\delta_C$ 158.8 (C-9b), 141.0 (C-5a), 124.7 (C-3a), 124.2 (C-4), 121.8 (C-5), 120.2 (C-9a), 89.8 (C-2), 71.8 (C-11), 57.1 (C-9), 34.7 (C-6), 30.6 (C-3), 26.4 (C-13), 25.3 (C-7), 24.1 (C-12), 14.2 (C-10); HRESIMS *m/z* 251.1627 $[M + H]^+$ (calcd for $C_{15}H_{23}O_3$, 251.1647).

(+)-Pseudodeflectusin (**12**): colorless oil; $^{1}$H NMR (400 MHz, $CDCl_3$) $\delta_H$ 7.61 (1H, d, *J* = 7.9 Hz, H-4), 6.88 (1H, d, *J* = 7.9 Hz, H-5), 6.28 (1H, s, H-9), 4.46 (1H, m,

H-7), 2.79 (1H, dd, $J$ = 17.3, 3.6 Hz, H-6), 2.71 (1H, $J$ = 17.3, 10.7 Hz, H-6), 2.36 (3H, s, H-13), 2.12 (3H, s, H-12), 1.40 (3H, d, $J$ = 6.2 Hz, H-10); $^{13}$C NMR (101 MHz, $CDCl_3$) $\delta_C$ 183.2 (C-3), 162.0 (C-9b), 145.2 (C-2), 143.5 (C-5a), 132.3 (C-11), 123.8 (C-4), 122.7 (C-5), 122.2 (C-3a), 119.5 (C-9a), 87.8 (C-9), 62.7 (C-7), 35.9 (C-6), 21.1 (C-10), 20.3 (C-12), 17.8 (C-13); HRESIMS $m/z$ 261.1136 $[M + H]^+$ (calcd for $C_{16}H_{21}O_4$, 261.1127).

(−)-Penicisochroman B (**13**): colorless oil; $^1$H NMR (400 MHz, $CDCl_3$) $\delta_H$ 7.50 (1H, d, $J$ = 7.9 Hz, H-4), 6.79 (1H, d, $J$ = 7.9 Hz, H-5), 5.67 (1H, s, H-9), 4.41 (1H, d, $J$ = 4.0 Hz, H-2), 4.25 (1H, m, H-7), 3.61 (3H, s, $OCH_3$-9), 2.74, (1H, dd, $J$ = 17.3, 3.8 Hz, H-6), 2.67 (1H, $J$ = 17.3, 10.5 Hz, H-6), 2.34 (1H, m, H-11), 1.39 (3H, d, $J$ = 6.3 Hz, H-10), 1.18 (3H, d, $J$ = 6.9 Hz, H-13), 0.87 (3H, d, $J$ = 6.9 Hz, H-12); $^{13}$C NMR (101 MHz, $CDCl_3$) $\delta_C$ 202.1 (C-3), 171.0 (C-9b), 145.5 (C-5a), 123.2 (C-4), 122.4 (C-5), 120.2 (C-3a), 120.0 (C-9a), 94.9 (C-9), 90.2 (C-2), 62.7 (C-7), 55.8, ($OCH_3$-9), 35.9 (C-6), 31.4 (C-11), 21.1 (C-10), 19.0 (C-12), 15.8 (C-13); HRESIMS $m/z$ 277.1438 $[M + H]^+$ (calcd for $C_{16}H_{21}O_4$, 277.1440).

Ustusorane A (**14**): colorless oil; $^1$H NMR (400 MHz, $CDCl_3$) $\delta_H$ 7.55 (1H, d, $J$ = 7.8 Hz, H-4), 6.95 (1H, d, $J$ = 7.8 Hz, H-5), 4.91 (1H, d, $J$ = 12.0 Hz, H-9), 4.72 (1H, dd, $J$ = 12.0, 2.2 Hz, H-9), 4.08 (1H, m, H-7), 2.95 (1H, dd, $J$ = 13.7, 9.2 Hz, H-6), 2.90 (1H, dt, $J$ = 13.7, 3.0 Hz, H-6), 2.35 (3H, s, H-13), 2.11 (3H, s, H-12), 1.37 (3H, d, $J$ = 6.4 Hz, H-10); $^{13}$C NMR (101 MHz, $CDCl_3$) $\delta_C$ 183.6 (C-3), 163.1 (C-9b), 147.8 (C-5a), 145.1 (C-2), 132.3 (C-11), 124.6 (C-5), 124.3 (C-9a), 123.6 (C-4), 121.8 (C-3a), 68.8 (C-7), 54.3 (C-8), 41.6, (C-6), 24.2 (C-10), 20.2 (C-12), 17.5 (C-13); HRESIMS $m/z$ 263.1298 $[M + H]^+$ (calcd for $C_{15}H_{19}O_4$, 263.1283).

**The rDNA sequencing data of *Aspergillus* sp. ZJ-68**

ATACACGGGGGGGAGGTTAGGCCTGCCCGGGGGCAGACCTGCACTCG
GTAATGATCCTTCCGTAGGGGAACCTGCGGAAGGATCATTACCGAGTGCAG
GTCTGCCCCCGGGCAGGCCTAACCTCCCACCCGTGAATACCTGACCAACGT
TGCTTCGGCGGTGCGCCCCCCCCGGGGGTAGCCGCCGGAGACCACACCGA
ACCTCCTGTCTTTAGTGTTGTCTGAGCTTGATAGCAAACCTATTAAAACTTT
CAACAATGGATCTCTTGGTTCCGGCATCGATGAAGAACGCAGCGAACTGCG
ATAAGTAATGTGAATTGCAGAATTCAGTGAATCATCGAGTCTTTGAACGCA
CATTGCGCCCCCTGGCATTCCGGGGGGCATGCCTGTCCGAGCGTCATTGCT
GCCCTTCAAGCCCGGCTTGTGTGTTGGGTCGTCGTCCCCCCCGGGGGACG
GGCCCGAAAGGCAGCGGCGGCACCGCGTCCGGTCCTCGAGCGTATGGGGC
TTTGTCACCCGCTCGATTAGGGCCGGCCGGGCGCCAGCCGGCGTCTCCAAC
CTTCTATTTTACCAGGTtGACCTCGGATCAGGTAGGGATACCCGCTGAACTT
AAGCATATCAATAAGGCGGGAGGAATCATCGTTACCCATA

**Figure S67**. Simple picture and microscopic picture of *Aspergillus* sp. ZJ-68

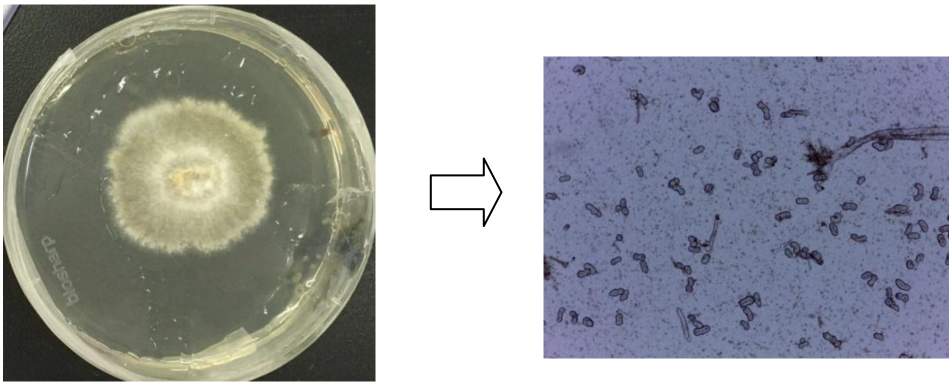

colonies of strain

hyphae and conidia of strain (10×10)

**Figure S68**. The chiral HPLC separation of (±)-penicisochroman A

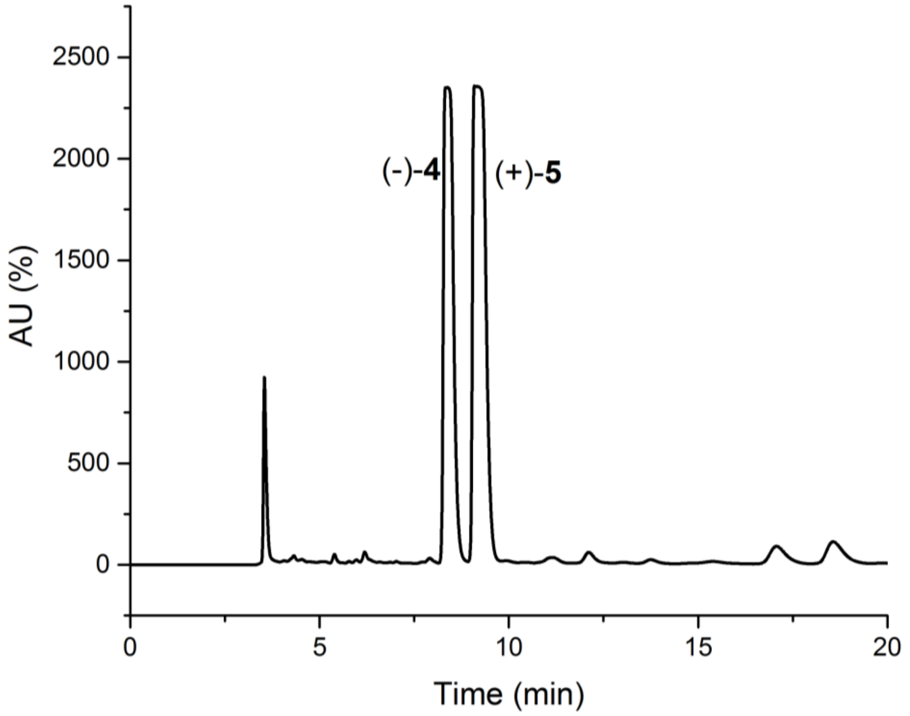

**ECD calculation details (Methods and Results)**

1. Methods

Conformational searches were carried out by means of the Spartan′14 software using Molecular Merck force field (MMFF). All density functional theory (DFT) and time-dependent (TD)-DFT calculations were performed with Gaussian 09 program. Conformers within a 10 kcal/mol energy window were generated and optimized by DFT calculations at the B3LYP/6-31+G (d, p) level. Conformers with a Bolzmann distribution over 3% were chose for ECD calculations by TD-DFT method at the B3LYP/6-311+G (d, p) level. The polarizable continuum model for MeOH was used. The calculated ECD curves were generated using the SpecDis 3.0 (University of Würzburg) and Origin Pro 8.5 (Origin Lab, Ltd.) from dipole-length rotational strengths by applying Gaussian band shapes with sigma = 0.30 eV.

2. Results

2.1. Gibbs free energies and Boltzmann-population of low-energy conformers of 2*R*,6*R*-**1**.

| Conformers of 2*R*,6*R*-**1** | In MeOH | |
|---|---|---|
| | Δ*G*[a] | P (%)[b] |
| **1a** | 0.00 | 42.6 |
| **1b** | 0.16 | 32.5 |
| **1c** | 0.83 | 10.4 |
| **1d** | 1.01 | 7.7 |
| **1e** | 1.08 | 6.8 |

[a]Δ*G*, B3LYP/ 6-31+G (d, p), in kcal/mol. [b]Boltzmann-population.

2.2. Gibbs free energies and Boltzmann-population of low-energy conformers of 2*S*-**3**.

| Conformers of 2*S* -**3** | In MeOH | |
|---|---|---|
| | Δ*G*[a] | P (%)[b] |
| **3a** | 0.00 | 66.7 |
| **3b** | 0.92 | 14.1 |
| **3c** | 1.22 | 8.5 |
| **3d** | 1.23 | 8.4 |

[a]Δ*G*, B3LYP/ 6-31+G (d, p), in kcal/mol. [b]Boltzmann-population.

2.3. Gibbs free energies and Boltzmann-population of low-energy conformers of 7*R*-**4**.

| Conformers of 7*R*-**4** | In MeOH | |
|---|---|---|
| | Δ*G*[a] | P (%)[b] |
| **4a** | 0.00 | 96.0 |
| **4b** | 2.25 | 2.1 |
| **4c** | 2.34 | 1.8 |

[a]Δ*G*, B3LYP/ 6-31+G (d, p), in kcal/mol. [b]Boltzmann-population.

2.4 Cartesian coordinates for the low-energy optimized conformers of 2*R*,6*R*-**1** at B3LYP/6-31+G (d, p) level of theory in MeOH.

| Conformer **1a** | | Standard Orientation (Ångstroms) | | | |
|---|---|---|---|---|---|
| Center Number | Atom | Type | X | Y | Z |
| 1 | 6 | 0 | -0.990000 | -1.120000 | 0.109000 |
| 2 | 6 | 0 | -2.264000 | -0.632000 | 0.302000 |
| 3 | 6 | 0 | -2.481000 | 0.729000 | 0.079000 |
| 4 | 6 | 0 | -1.434000 | 1.606000 | -0.322000 |
| 5 | 6 | 0 | -0.127000 | 1.083000 | -0.503000 |
| 6 | 6 | 0 | 0.043000 | -0.283000 | -0.283000 |
| 7 | 8 | 0 | 1.243000 | -0.947000 | -0.398000 |
| 8 | 6 | 0 | 0.871000 | -2.358000 | -0.418000 |
| 9 | 6 | 0 | -0.470000 | -2.490000 | 0.322000 |
| 10 | 6 | 0 | 2.021000 | -3.194000 | 0.191000 |
| 11 | 6 | 0 | 1.682000 | -4.686000 | 0.207000 |
| 12 | 6 | 0 | 3.331000 | -2.969000 | -0.572000 |
| 13 | 6 | 0 | -1.804000 | 3.070000 | -0.506000 |
| 14 | 6 | 0 | 1.054000 | 1.904000 | -0.950000 |
| 15 | 8 | 0 | 2.245000 | -2.791000 | 1.549000 |
| 16 | 8 | 0 | 0.949000 | 2.235000 | -2.328000 |
| 17 | 6 | 0 | -1.393000 | 3.977000 | 0.662000 |
| 18 | 6 | 0 | -2.107000 | 3.654000 | 1.966000 |
| 19 | 8 | 0 | -1.229000 | 3.560000 | -1.719000 |
| 20 | 1 | 0 | -3.074000 | -1.279000 | 0.622000 |
| 21 | 1 | 0 | -3.487000 | 1.117000 | 0.228000 |
| 22 | 1 | 0 | 0.739000 | -2.633000 | -1.475000 |
| 23 | 1 | 0 | -1.100000 | -3.266000 | -0.122000 |
| 24 | 1 | 0 | -0.349000 | -2.675000 | 1.394000 |
| 25 | 1 | 0 | 0.811000 | -4.892000 | 0.838000 |
| 26 | 1 | 0 | 2.508000 | -5.268000 | 0.633000 |

| | | | | | |
|---|---|---|---|---|---|
| 27 | 1 | 0 | 1.479000 | -5.063000 | -0.801000 |
| 28 | 1 | 0 | 3.649000 | -1.921000 | -0.521000 |
| 29 | 1 | 0 | 3.236000 | -3.252000 | -1.626000 |
| 30 | 1 | 0 | 4.145000 | -3.554000 | -0.129000 |
| 31 | 1 | 0 | -2.889000 | 3.147000 | -0.647000 |
| 32 | 1 | 0 | 1.999000 | 1.370000 | -0.809000 |
| 33 | 1 | 0 | 1.140000 | 2.825000 | -0.368000 |
| 34 | 1 | 0 | 2.342000 | -1.819000 | 1.532000 |
| 35 | 1 | 0 | 0.069000 | 2.661000 | -2.416000 |
| 36 | 1 | 0 | -1.609000 | 5.019000 | 0.393000 |
| 37 | 1 | 0 | -0.311000 | 3.941000 | 0.828000 |
| 38 | 1 | 0 | -1.842000 | 4.390000 | 2.732000 |
| 39 | 1 | 0 | -1.825000 | 2.667000 | 2.345000 |
| 40 | 1 | 0 | -3.194000 | 3.679000 | 1.836000 |
| 41 | 1 | 0 | -1.578000 | 4.459000 | -1.853000 |

| Conformer **1b** | | Standard Orientation (Ångstroms) | | | |
|---|---|---|---|---|---|
| Center Number | Atom | Type | X | Y | Z |
| 1 | 6 | 0 | -0.669000 | -1.079000 | 0.567000 |
| 2 | 6 | 0 | -1.722000 | -1.419000 | -0.254000 |
| 3 | 6 | 0 | -1.946000 | -0.642000 | -1.394000 |
| 4 | 6 | 0 | -1.126000 | 0.468000 | -1.719000 |
| 5 | 6 | 0 | -0.082000 | 0.833000 | -0.826000 |
| 6 | 6 | 0 | 0.126000 | 0.019000 | 0.284000 |
| 7 | 8 | 0 | 1.104000 | 0.224000 | 1.229000 |
| 8 | 6 | 0 | 1.124000 | -1.013000 | 2.004000 |
| 9 | 6 | 0 | -0.254000 | -1.680000 | 1.854000 |
| 10 | 6 | 0 | 1.520000 | -0.690000 | 3.465000 |
| 11 | 6 | 0 | 1.565000 | -1.955000 | 4.325000 |
| 12 | 6 | 0 | 2.874000 | 0.025000 | 3.524000 |
| 13 | 6 | 0 | -1.402000 | 1.296000 | -2.968000 |
| 14 | 6 | 0 | 0.789000 | 2.042000 | -1.035000 |
| 15 | 8 | 0 | 0.550000 | 0.190000 | 4.048000 |
| 16 | 8 | 0 | -0.030000 | 3.205000 | -1.140000 |
| 17 | 6 | 0 | -2.093000 | 0.607000 | -4.155000 |
| 18 | 6 | 0 | -1.256000 | -0.498000 | -4.782000 |
| 19 | 8 | 0 | -2.212000 | 2.408000 | -2.584000 |
| 20 | 1 | 0 | -2.374000 | -2.254000 | -0.019000 |
| 21 | 1 | 0 | -2.791000 | -0.907000 | -2.025000 |
| 22 | 1 | 0 | 1.895000 | -1.653000 | 1.550000 |

| | | | | | |
|---|---|---|---|---|---|
| 23 | 1 | 0 | -0.171000 | -2.770000 | 1.815000 |
| 24 | 1 | 0 | -0.956000 | -1.388000 | 2.641000 |
| 25 | 1 | 0 | 0.579000 | -2.429000 | 4.391000 |
| 26 | 1 | 0 | 1.852000 | -1.714000 | 5.355000 |
| 27 | 1 | 0 | 2.277000 | -2.685000 | 3.929000 |
| 28 | 1 | 0 | 2.843000 | 0.985000 | 2.997000 |
| 29 | 1 | 0 | 3.671000 | -0.587000 | 3.088000 |
| 30 | 1 | 0 | 3.145000 | 0.258000 | 4.561000 |
| 31 | 1 | 0 | -0.458000 | 1.698000 | -3.354000 |
| 32 | 1 | 0 | 1.382000 | 1.931000 | -1.948000 |
| 33 | 1 | 0 | 1.480000 | 2.199000 | -0.201000 |
| 34 | 1 | 0 | 0.417000 | 0.920000 | 3.412000 |
| 35 | 1 | 0 | 0.577000 | 3.965000 | -1.129000 |
| 36 | 1 | 0 | -2.298000 | 1.365000 | -4.923000 |
| 37 | 1 | 0 | -3.081000 | 0.224000 | -3.873000 |
| 38 | 1 | 0 | -1.769000 | -0.904000 | -5.659000 |
| 39 | 1 | 0 | -1.082000 | -1.322000 | -4.086000 |
| 40 | 1 | 0 | -0.284000 | -0.114000 | -5.109000 |
| 41 | 1 | 0 | -1.716000 | 2.843000 | -1.857000 |

| Conformer **1c** | | Standard Orientation (Ångstroms) | | | |
|---|---|---|---|---|---|
| Center Number | Atom | Type | X | Y | Z |
| 1 | 6 | 0 | -0.806000 | -0.731000 | 0.776000 |
| 2 | 6 | 0 | -1.934000 | 0.039000 | 0.964000 |
| 3 | 6 | 0 | -2.015000 | 1.264000 | 0.297000 |
| 4 | 6 | 0 | -0.980000 | 1.724000 | -0.556000 |
| 5 | 6 | 0 | 0.198000 | 0.938000 | -0.689000 |
| 6 | 6 | 0 | 0.230000 | -0.288000 | -0.030000 |
| 7 | 8 | 0 | 1.284000 | -1.169000 | -0.078000 |
| 8 | 6 | 0 | 0.763000 | -2.390000 | 0.531000 |
| 9 | 6 | 0 | -0.445000 | -2.022000 | 1.403000 |
| 10 | 6 | 0 | 1.914000 | -3.122000 | 1.262000 |
| 11 | 6 | 0 | 2.627000 | -2.250000 | 2.300000 |
| 12 | 6 | 0 | 1.430000 | -4.424000 | 1.903000 |
| 13 | 6 | 0 | -1.099000 | 3.068000 | -1.264000 |
| 14 | 6 | 0 | 1.386000 | 1.376000 | -1.502000 |
| 15 | 8 | 0 | 2.900000 | -3.486000 | 0.279000 |
| 16 | 8 | 0 | 1.837000 | 2.647000 | -1.039000 |
| 17 | 6 | 0 | -2.506000 | 3.599000 | -1.577000 |
| 18 | 6 | 0 | -3.269000 | 2.737000 | -2.573000 |
| 19 | 8 | 0 | -0.439000 | 4.045000 | -0.458000 |

| | | | | | |
|---|---|---|---|---|---|
| 20 | 1 | 0 | -2.735000 | -0.284000 | 1.621000 |
| 21 | 1 | 0 | -2.900000 | 1.872000 | 0.466000 |
| 22 | 1 | 0 | 0.430000 | -3.013000 | -0.313000 |
| 23 | 1 | 0 | -1.236000 | -2.773000 | 1.328000 |
| 24 | 1 | 0 | -0.186000 | -1.863000 | 2.453000 |
| 25 | 1 | 0 | 3.034000 | -1.340000 | 1.845000 |
| 26 | 1 | 0 | 3.485000 | -2.783000 | 2.727000 |
| 27 | 1 | 0 | 1.966000 | -1.961000 | 3.123000 |
| 28 | 1 | 0 | 0.957000 | -5.072000 | 1.156000 |
| 29 | 1 | 0 | 0.714000 | -4.244000 | 2.710000 |
| 30 | 1 | 0 | 2.273000 | -4.993000 | 2.313000 |
| 31 | 1 | 0 | -0.568000 | 3.022000 | -2.222000 |
| 32 | 1 | 0 | 1.117000 | 1.450000 | -2.561000 |
| 33 | 1 | 0 | 2.223000 | 0.677000 | -1.415000 |
| 34 | 1 | 0 | 3.112000 | -2.670000 | -0.212000 |
| 35 | 1 | 0 | 2.682000 | 2.816000 | -1.492000 |
| 36 | 1 | 0 | -2.409000 | 4.609000 | -1.997000 |
| 37 | 1 | 0 | -3.094000 | 3.734000 | -0.662000 |
| 38 | 1 | 0 | -4.233000 | 3.199000 | -2.806000 |
| 39 | 1 | 0 | -3.465000 | 1.736000 | -2.179000 |
| 40 | 1 | 0 | -2.710000 | 2.635000 | -3.508000 |
| 41 | 1 | 0 | 0.467000 | 3.691000 | -0.324000 |

| Conformer **1d** | | Standard Orientation (Ångstroms) | | | |
|---|---|---|---|---|---|
| Center Number | Atom | Type | X | Y | Z |
| 1 | 6 | 0 | -0.920000 | -0.779000 | 1.113000 |
| 2 | 6 | 0 | -2.208000 | -0.292000 | 1.082000 |
| 3 | 6 | 0 | -2.554000 | 0.573000 | 0.042000 |
| 4 | 6 | 0 | -1.621000 | 0.960000 | -0.959000 |
| 5 | 6 | 0 | -0.295000 | 0.462000 | -0.896000 |
| 6 | 6 | 0 | 0.006000 | -0.410000 | 0.149000 |
| 7 | 8 | 0 | 1.241000 | -0.977000 | 0.359000 |
| 8 | 6 | 0 | 0.987000 | -2.042000 | 1.325000 |
| 9 | 6 | 0 | -0.274000 | -1.655000 | 2.116000 |
| 10 | 6 | 0 | 2.248000 | -2.249000 | 2.197000 |
| 11 | 6 | 0 | 2.037000 | -3.354000 | 3.234000 |
| 12 | 6 | 0 | 3.469000 | -2.579000 | 1.330000 |
| 13 | 6 | 0 | -2.120000 | 1.945000 | -2.013000 |
| 14 | 6 | 0 | 0.764000 | 0.789000 | -1.908000 |
| 15 | 8 | 0 | 2.548000 | -1.042000 | 2.911000 |
| 16 | 8 | 0 | 0.458000 | 0.093000 | -3.114000 |

| | | | | | |
|---|---|---|---|---|---|
| 17 | 6 | 0 | -1.778000 | 3.393000 | -1.633000 |
| 18 | 6 | 0 | -2.402000 | 4.405000 | -2.585000 |
| 19 | 8 | 0 | -1.656000 | 1.671000 | -3.330000 |
| 20 | 1 | 0 | -2.934000 | -0.563000 | 1.842000 |
| 21 | 1 | 0 | -3.571000 | 0.963000 | 0.014000 |
| 22 | 1 | 0 | 0.792000 | -2.954000 | 0.742000 |
| 23 | 1 | 0 | -0.872000 | -2.534000 | 2.373000 |
| 24 | 1 | 0 | -0.050000 | -1.078000 | 3.019000 |
| 25 | 1 | 0 | 1.240000 | -3.093000 | 3.939000 |
| 26 | 1 | 0 | 2.939000 | -3.496000 | 3.840000 |
| 27 | 1 | 0 | 1.785000 | -4.308000 | 2.760000 |
| 28 | 1 | 0 | 3.702000 | -1.761000 | 0.640000 |
| 29 | 1 | 0 | 3.312000 | -3.493000 | 0.748000 |
| 30 | 1 | 0 | 4.362000 | -2.714000 | 1.952000 |
| 31 | 1 | 0 | -3.212000 | 1.851000 | -2.075000 |
| 32 | 1 | 0 | 1.762000 | 0.485000 | -1.577000 |
| 33 | 1 | 0 | 0.803000 | 1.863000 | -2.108000 |
| 34 | 1 | 0 | 2.549000 | -0.323000 | 2.249000 |
| 35 | 1 | 0 | 1.185000 | 0.290000 | -3.730000 |
| 36 | 1 | 0 | -0.693000 | 3.544000 | -1.643000 |
| 37 | 1 | 0 | -2.123000 | 3.607000 | -0.614000 |
| 38 | 1 | 0 | -2.163000 | 5.423000 | -2.261000 |
| 39 | 1 | 0 | -3.492000 | 4.304000 | -2.605000 |
| 40 | 1 | 0 | -2.025000 | 4.280000 | -3.604000 |
| 41 | 1 | 0 | -1.226000 | 0.791000 | -3.324000 |

| Conformer **1e** | | Standard Orientation (Ångstroms) | | | |
|---|---|---|---|---|---|
| Center Number | Atom | Type | X | Y | Z |
| 1 | 6 | 0 | -1.182000 | -0.608000 | 0.918000 |
| 2 | 6 | 0 | -2.426000 | -0.064000 | 0.701000 |
| 3 | 6 | 0 | -2.606000 | 0.712000 | -0.446000 |
| 4 | 6 | 0 | -1.551000 | 0.961000 | -1.370000 |
| 5 | 6 | 0 | -0.279000 | 0.376000 | -1.135000 |
| 6 | 6 | 0 | -0.144000 | -0.389000 | 0.025000 |
| 7 | 8 | 0 | 1.030000 | -0.987000 | 0.429000 |
| 8 | 6 | 0 | 0.615000 | -1.929000 | 1.465000 |
| 9 | 6 | 0 | -0.696000 | -1.402000 | 2.069000 |
| 10 | 6 | 0 | 1.765000 | -2.099000 | 2.486000 |
| 11 | 6 | 0 | 1.385000 | -3.076000 | 3.601000 |
| 12 | 6 | 0 | 3.049000 | -2.575000 | 1.797000 |
| 13 | 6 | 0 | -1.900000 | 1.856000 | -2.555000 |

| | | | | | |
|---|---|---|---|---|---|
| 14 | 6 | 0 | 0.929000 | 0.553000 | -2.013000 |
| 15 | 8 | 0 | 2.057000 | -0.837000 | 3.102000 |
| 16 | 8 | 0 | 0.604000 | 0.321000 | -3.380000 |
| 17 | 6 | 0 | -1.085000 | 3.149000 | -2.695000 |
| 18 | 6 | 0 | -1.160000 | 4.049000 | -1.471000 |
| 19 | 8 | 0 | -1.835000 | 1.122000 | -3.775000 |
| 20 | 1 | 0 | -3.243000 | -0.221000 | 1.397000 |
| 21 | 1 | 0 | -3.592000 | 1.142000 | -0.621000 |
| 22 | 1 | 0 | 0.431000 | -2.889000 | 0.960000 |
| 23 | 1 | 0 | -1.368000 | -2.219000 | 2.346000 |
| 24 | 1 | 0 | -0.535000 | -0.744000 | 2.929000 |
| 25 | 1 | 0 | 0.534000 | -2.705000 | 4.184000 |
| 26 | 1 | 0 | 2.210000 | -3.194000 | 4.313000 |
| 27 | 1 | 0 | 1.130000 | -4.062000 | 3.201000 |
| 28 | 1 | 0 | 3.399000 | -1.847000 | 1.057000 |
| 29 | 1 | 0 | 2.902000 | -3.537000 | 1.295000 |
| 30 | 1 | 0 | 3.863000 | -2.684000 | 2.523000 |
| 31 | 1 | 0 | -2.950000 | 2.161000 | -2.469000 |
| 32 | 1 | 0 | 1.722000 | -0.156000 | -1.755000 |
| 33 | 1 | 0 | 1.337000 | 1.562000 | -1.897000 |
| 34 | 1 | 0 | 2.165000 | -0.193000 | 2.377000 |
| 35 | 1 | 0 | 1.449000 | 0.328000 | -3.863000 |
| 36 | 1 | 0 | -1.465000 | 3.703000 | -3.563000 |
| 37 | 1 | 0 | -0.039000 | 2.942000 | -2.937000 |
| 38 | 1 | 0 | -0.635000 | 4.989000 | -1.663000 |
| 39 | 1 | 0 | -0.694000 | 3.580000 | -0.599000 |
| 40 | 1 | 0 | -2.199000 | 4.287000 | -1.222000 |
| 41 | 1 | 0 | -0.990000 | 0.623000 | -3.748000 |

2.5 Cartesian coordinates for the low-energy optimized conformers of 2*S*-**3** at B3LYP/6-31+G (d, p) level of theory in MeOH.

| Conformer **3a** | | Standard Orientation (Ångstroms) | | | |
|---|---|---|---|---|---|
| Center Number | Atom | Type | X | Y | Z |
| 1 | 6 | 0 | -0.319000 | -0.918000 | -1.375000 |
| 2 | 6 | 0 | -0.224000 | -2.294000 | -1.329000 |
| 3 | 6 | 0 | -0.255000 | -2.889000 | -0.066000 |
| 4 | 6 | 0 | -0.380000 | -2.098000 | 1.095000 |
| 5 | 6 | 0 | -0.475000 | -0.701000 | 1.007000 |
| 6 | 6 | 0 | -0.435000 | -0.125000 | -0.251000 |
| 7 | 8 | 0 | -0.474000 | 1.225000 | -0.488000 |

| | | | | | |
|---|---|---|---|---|---|
| 8 | 6 | 0 | -0.482000 | 1.379000 | -1.935000 |
| 9 | 6 | 0 | -0.289000 | -0.013000 | -2.493000 |
| 10 | 6 | 0 | 0.607000 | 2.359000 | -2.420000 |
| 11 | 6 | 0 | 0.379000 | 3.741000 | -1.794000 |
| 12 | 6 | 0 | 2.030000 | 1.893000 | -2.088000 |
| 13 | 8 | 0 | -0.113000 | -0.334000 | -3.654000 |
| 14 | 6 | 0 | -0.409000 | -2.697000 | 2.424000 |
| 15 | 6 | 0 | -0.720000 | -1.947000 | 3.494000 |
| 16 | 8 | 0 | -0.996000 | -0.596000 | 3.431000 |
| 17 | 6 | 0 | -0.533000 | 0.110000 | 2.268000 |
| 18 | 6 | 0 | -0.831000 | -2.519000 | 4.874000 |
| 19 | 8 | 0 | 0.513000 | 2.539000 | -3.840000 |
| 20 | 1 | 0 | -0.125000 | -2.883000 | -2.234000 |
| 21 | 1 | 0 | -0.182000 | -3.972000 | 0.013000 |
| 22 | 1 | 0 | -1.481000 | 1.721000 | -2.235000 |
| 23 | 1 | 0 | -0.620000 | 4.118000 | -2.042000 |
| 24 | 1 | 0 | 1.091000 | 4.472000 | -2.192000 |
| 25 | 1 | 0 | 0.480000 | 3.720000 | -0.704000 |
| 26 | 1 | 0 | 2.270000 | 0.951000 | -2.593000 |
| 27 | 1 | 0 | 2.175000 | 1.759000 | -1.011000 |
| 28 | 1 | 0 | 2.768000 | 2.621000 | -2.445000 |
| 29 | 1 | 0 | -0.215000 | -3.760000 | 2.517000 |
| 30 | 1 | 0 | -1.208000 | 0.962000 | 2.135000 |
| 31 | 1 | 0 | 0.467000 | 0.502000 | 2.491000 |
| 32 | 1 | 0 | -1.840000 | -2.361000 | 5.268000 |
| 33 | 1 | 0 | -0.627000 | -3.594000 | 4.893000 |
| 34 | 1 | 0 | -0.118000 | -2.028000 | 5.543000 |
| 35 | 1 | 0 | 0.573000 | 1.657000 | -4.264000 |

| Conformer **3b** | | Standard Orientation (Ångstroms) | | | |
|---|---|---|---|---|---|
| Center Number | Atom | Type | X | Y | Z |
| 1 | 6 | 0 | -0.357000 | -0.858000 | -1.284000 |
| 2 | 6 | 0 | -0.262000 | -2.235000 | -1.233000 |
| 3 | 6 | 0 | -0.282000 | -2.827000 | 0.031000 |
| 4 | 6 | 0 | -0.399000 | -2.033000 | 1.190000 |
| 5 | 6 | 0 | -0.498000 | -0.636000 | 1.099000 |
| 6 | 6 | 0 | -0.467000 | -0.064000 | -0.161000 |
| 7 | 8 | 0 | -0.514000 | 1.285000 | -0.402000 |
| 8 | 6 | 0 | -0.501000 | 1.439000 | -1.861000 |
| 9 | 6 | 0 | -0.345000 | 0.037000 | -2.410000 |
| 10 | 6 | 0 | 0.620000 | 2.420000 | -2.258000 |

| | | | | | |
|---|---|---|---|---|---|
| 11 | 6 | 0 | 2.015000 | 1.948000 | -1.834000 |
| 12 | 6 | 0 | 0.599000 | 2.736000 | -3.754000 |
| 13 | 8 | 0 | -0.235000 | -0.320000 | -3.568000 |
| 14 | 6 | 0 | -0.419000 | -2.628000 | 2.522000 |
| 15 | 6 | 0 | -0.724000 | -1.876000 | 3.591000 |
| 16 | 8 | 0 | -1.002000 | -0.526000 | 3.527000 |
| 17 | 6 | 0 | -0.550000 | 0.177000 | 2.358000 |
| 18 | 6 | 0 | -0.825000 | -2.444000 | 4.973000 |
| 19 | 8 | 0 | 0.379000 | 3.669000 | -1.579000 |
| 20 | 1 | 0 | -0.172000 | -2.827000 | -2.138000 |
| 21 | 1 | 0 | -0.208000 | -3.910000 | 0.112000 |
| 22 | 1 | 0 | -1.489000 | 1.828000 | -2.135000 |
| 23 | 1 | 0 | 2.073000 | 1.800000 | -0.750000 |
| 24 | 1 | 0 | 2.768000 | 2.707000 | -2.076000 |
| 25 | 1 | 0 | 2.299000 | 1.015000 | -2.329000 |
| 26 | 1 | 0 | -0.393000 | 3.082000 | -4.065000 |
| 27 | 1 | 0 | 0.874000 | 1.871000 | -4.365000 |
| 28 | 1 | 0 | 1.295000 | 3.550000 | -3.989000 |
| 29 | 1 | 0 | -0.221000 | -3.690000 | 2.616000 |
| 30 | 1 | 0 | -1.229000 | 1.027000 | 2.227000 |
| 31 | 1 | 0 | 0.449000 | 0.574000 | 2.573000 |
| 32 | 1 | 0 | -1.831000 | -2.287000 | 5.374000 |
| 33 | 1 | 0 | -0.618000 | -3.519000 | 4.994000 |
| 34 | 1 | 0 | -0.108000 | -1.950000 | 5.637000 |
| 35 | 1 | 0 | 0.277000 | 3.464000 | -0.631000 |

| Conformer **3c** | | Standard Orientation (Ångstroms) | | | |
|---|---|---|---|---|---|
| Center Number | Atom | Type | X | Y | Z |
| 1 | 6 | 0 | -0.313000 | -0.971000 | -1.313000 |
| 2 | 6 | 0 | -0.196000 | -2.346000 | -1.262000 |
| 3 | 6 | 0 | -0.177000 | -2.937000 | 0.003000 |
| 4 | 6 | 0 | -0.285000 | -2.143000 | 1.163000 |
| 5 | 6 | 0 | -0.413000 | -0.749000 | 1.072000 |
| 6 | 6 | 0 | -0.414000 | -0.178000 | -0.189000 |
| 7 | 8 | 0 | -0.503000 | 1.169000 | -0.430000 |
| 8 | 6 | 0 | -0.450000 | 1.330000 | -1.886000 |
| 9 | 6 | 0 | -0.360000 | -0.078000 | -2.439000 |
| 10 | 6 | 0 | 0.739000 | 2.243000 | -2.251000 |
| 11 | 6 | 0 | 0.928000 | 2.387000 | -3.762000 |
| 12 | 6 | 0 | 0.544000 | 3.636000 | -1.633000 |
| 13 | 8 | 0 | -0.372000 | -0.441000 | -3.601000 |

| | | | | | |
|---|---|---|---|---|---|
| 14 | 6 | 0 | -0.268000 | -2.737000 | 2.495000 |
| 15 | 6 | 0 | -0.568000 | -1.989000 | 3.570000 |
| 16 | 8 | 0 | -0.875000 | -0.646000 | 3.510000 |
| 17 | 6 | 0 | -0.461000 | 0.065000 | 2.331000 |
| 18 | 6 | 0 | -0.631000 | -2.558000 | 4.954000 |
| 19 | 8 | 0 | 1.960000 | 1.714000 | -1.710000 |
| 20 | 1 | 0 | -0.118000 | -2.936000 | -2.169000 |
| 21 | 1 | 0 | -0.083000 | -4.018000 | 0.083000 |
| 22 | 1 | 0 | -1.407000 | 1.764000 | -2.199000 |
| 23 | 1 | 0 | 1.250000 | 1.446000 | -4.219000 |
| 24 | 1 | 0 | 1.721000 | 3.110000 | -3.987000 |
| 25 | 1 | 0 | 0.009000 | 2.724000 | -4.252000 |
| 26 | 1 | 0 | 0.484000 | 3.584000 | -0.540000 |
| 27 | 1 | 0 | -0.366000 | 4.117000 | -2.008000 |
| 28 | 1 | 0 | 1.397000 | 4.286000 | -1.859000 |
| 29 | 1 | 0 | -0.047000 | -3.795000 | 2.586000 |
| 30 | 1 | 0 | -1.162000 | 0.898000 | 2.211000 |
| 31 | 1 | 0 | 0.532000 | 0.486000 | 2.527000 |
| 32 | 1 | 0 | -1.632000 | -2.422000 | 5.373000 |
| 33 | 1 | 0 | -0.401000 | -3.629000 | 4.971000 |
| 34 | 1 | 0 | 0.089000 | -2.050000 | 5.603000 |
| 35 | 1 | 0 | 1.852000 | 1.662000 | -0.742000 |

| Conformer **3d** | | Standard Orientation (Ångstroms) | | | |
|---|---|---|---|---|---|
| Center Number | Atom | Type | X | Y | Z |
| 1 | 6 | 0 | -0.197000 | -0.999000 | -1.334000 |
| 2 | 6 | 0 | -0.036000 | -2.369000 | -1.260000 |
| 3 | 6 | 0 | -0.118000 | -2.951000 | 0.007000 |
| 4 | 6 | 0 | -0.352000 | -2.153000 | 1.145000 |
| 5 | 6 | 0 | -0.508000 | -0.763000 | 1.031000 |
| 6 | 6 | 0 | -0.433000 | -0.204000 | -0.233000 |
| 7 | 8 | 0 | -0.587000 | 1.132000 | -0.500000 |
| 8 | 6 | 0 | -0.397000 | 1.287000 | -1.944000 |
| 9 | 6 | 0 | -0.191000 | -0.118000 | -2.471000 |
| 10 | 6 | 0 | 0.782000 | 2.250000 | -2.201000 |
| 11 | 6 | 0 | 1.105000 | 2.398000 | -3.689000 |
| 12 | 6 | 0 | 0.471000 | 3.634000 | -1.612000 |
| 13 | 8 | 0 | -0.089000 | -0.490000 | -3.626000 |
| 14 | 6 | 0 | -0.442000 | -2.737000 | 2.479000 |
| 15 | 6 | 0 | -0.465000 | -1.942000 | 3.562000 |

| | | | | | |
|---|---|---|---|---|---|
| 16 | 8 | 0 | -0.439000 | -0.564000 | 3.502000 |
| 17 | 6 | 0 | -0.829000 | 0.039000 | 2.256000 |
| 18 | 6 | 0 | -0.487000 | -2.483000 | 4.958000 |
| 19 | 8 | 0 | 1.968000 | 1.773000 | -1.546000 |
| 20 | 1 | 0 | 0.143000 | -2.962000 | -2.150000 |
| 21 | 1 | 0 | 0.004000 | -4.028000 | 0.105000 |
| 22 | 1 | 0 | -1.337000 | 1.677000 | -2.354000 |
| 23 | 1 | 0 | 1.504000 | 1.469000 | -4.108000 |
| 24 | 1 | 0 | 1.887000 | 3.150000 | -3.843000 |
| 25 | 1 | 0 | 0.223000 | 2.696000 | -4.264000 |
| 26 | 1 | 0 | 0.313000 | 3.583000 | -0.529000 |
| 27 | 1 | 0 | -0.419000 | 4.075000 | -2.073000 |
| 28 | 1 | 0 | 1.314000 | 4.319000 | -1.763000 |
| 29 | 1 | 0 | -0.447000 | -3.818000 | 2.570000 |
| 30 | 1 | 0 | -1.909000 | 0.222000 | 2.297000 |
| 31 | 1 | 0 | -0.324000 | 1.010000 | 2.212000 |
| 32 | 1 | 0 | -1.384000 | -2.137000 | 5.481000 |
| 33 | 1 | 0 | -0.488000 | -3.578000 | 4.976000 |
| 34 | 1 | 0 | 0.392000 | -2.138000 | 5.511000 |
| 35 | 1 | 0 | 1.775000 | 1.719000 | -0.591000 |

2.6 Cartesian coordinates for the low-energy optimized conformers of 7*R*-**4** at B3LYP/6-31+G (d, p) level of theory in MeOH.

| Conformer **4a** | | Standard Orientation (Ångstroms) | | | |
|---|---|---|---|---|---|
| Center Number | Atom | Type | X | Y | Z |
| 1 | 6 | 0 | 0.155000 | 2.123000 | 0.656000 |
| 2 | 6 | 0 | 0.334000 | 2.094000 | 2.024000 |
| 3 | 6 | 0 | 0.209000 | 0.855000 | 2.655000 |
| 4 | 6 | 0 | -0.084000 | -0.313000 | 1.911000 |
| 5 | 6 | 0 | -0.282000 | -0.251000 | 0.519000 |
| 6 | 6 | 0 | -0.143000 | 0.986000 | -0.086000 |
| 7 | 8 | 0 | -0.272000 | 1.242000 | -1.430000 |
| 8 | 6 | 0 | -0.054000 | 2.615000 | -1.599000 |
| 9 | 6 | 0 | 0.229000 | 3.244000 | -0.270000 |
| 10 | 6 | 0 | -0.036000 | 3.204000 | -2.815000 |
| 11 | 6 | 0 | -0.339000 | 2.425000 | -4.070000 |
| 12 | 6 | 0 | 0.213000 | 4.667000 | -3.065000 |
| 13 | 8 | 0 | 0.418000 | 4.400000 | 0.023000 |
| 14 | 6 | 0 | -0.249000 | -1.639000 | 2.616000 |

| | | | | | |
|---|---|---|---|---|---|
| 15 | 6 | 0 | -0.186000 | -2.846000 | 1.647000 |
| 16 | 8 | 0 | -1.018000 | -2.592000 | 0.510000 |
| 17 | 6 | 0 | -0.565000 | -1.488000 | -0.280000 |
| 18 | 6 | 0 | -0.771000 | -4.066000 | 2.375000 |
| 19 | 8 | 0 | 1.187000 | -3.070000 | 1.296000 |
| 20 | 6 | 0 | 1.427000 | -4.212000 | 0.486000 |
| 21 | 1 | 0 | 0.564000 | 2.997000 | 2.579000 |
| 22 | 1 | 0 | 0.342000 | 0.792000 | 3.734000 |
| 23 | 1 | 0 | -0.520000 | 1.362000 | -3.883000 |
| 24 | 1 | 0 | -1.233000 | 2.829000 | -4.558000 |
| 25 | 1 | 0 | 0.500000 | 2.497000 | -4.770000 |
| 26 | 1 | 0 | 0.435000 | 5.243000 | -2.167000 |
| 27 | 1 | 0 | 1.062000 | 4.791000 | -3.746000 |
| 28 | 1 | 0 | -0.668000 | 5.122000 | -3.531000 |
| 29 | 1 | 0 | 0.527000 | -1.741000 | 3.385000 |
| 30 | 1 | 0 | -1.229000 | -1.604000 | 3.109000 |
| 31 | 1 | 0 | 0.331000 | -1.786000 | -0.836000 |
| 32 | 1 | 0 | -1.357000 | -1.285000 | -1.008000 |
| 33 | 1 | 0 | -0.137000 | -4.371000 | 3.214000 |
| 34 | 1 | 0 | -1.782000 | -3.861000 | 2.744000 |
| 35 | 1 | 0 | -0.867000 | -4.924000 | 1.699000 |
| 36 | 1 | 0 | 2.452000 | -4.149000 | 0.111000 |
| 37 | 1 | 0 | 0.749000 | -4.251000 | -0.372000 |
| 38 | 1 | 0 | 1.344000 | -5.127000 | 1.078000 |

| Conformer **4b** | | Standard Orientation (Ångstroms) | | | |
|---|---|---|---|---|---|
| Center Number | Atom | Type | X | Y | Z |
| 1 | 6 | 0 | 0.057000 | 1.994000 | 0.455000 |
| 2 | 6 | 0 | 0.237000 | 1.971000 | 1.823000 |
| 3 | 6 | 0 | 0.097000 | 0.738000 | 2.464000 |
| 4 | 6 | 0 | -0.209000 | -0.430000 | 1.726000 |
| 5 | 6 | 0 | -0.409000 | -0.374000 | 0.332000 |
| 6 | 6 | 0 | -0.256000 | 0.857000 | -0.280000 |
| 7 | 8 | 0 | -0.386000 | 1.108000 | -1.626000 |
| 8 | 6 | 0 | -0.152000 | 2.477000 | -1.802000 |
| 9 | 6 | 0 | 0.143000 | 3.109000 | -0.478000 |
| 10 | 6 | 0 | -0.131000 | 3.061000 | -3.022000 |
| 11 | 6 | 0 | -0.446000 | 2.280000 | -4.272000 |
| 12 | 6 | 0 | 0.135000 | 4.520000 | -3.280000 |
| 13 | 8 | 0 | 0.352000 | 4.262000 | -0.191000 |
| 14 | 6 | 0 | -0.390000 | -1.754000 | 2.434000 |

| | | | | | |
|---|---|---|---|---|---|
| 15 | 6 | 0 | -0.254000 | -2.949000 | 1.460000 |
| 16 | 8 | 0 | -1.140000 | -2.714000 | 0.350000 |
| 17 | 6 | 0 | -0.717000 | -1.613000 | -0.460000 |
| 18 | 6 | 0 | -0.777000 | -4.226000 | 2.128000 |
| 19 | 8 | 0 | 1.068000 | -3.183000 | 0.960000 |
| 20 | 6 | 0 | 2.111000 | -3.270000 | 1.916000 |
| 21 | 1 | 0 | 0.475000 | 2.876000 | 2.372000 |
| 22 | 1 | 0 | 0.226000 | 0.683000 | 3.543000 |
| 23 | 1 | 0 | -0.639000 | 1.220000 | -4.079000 |
| 24 | 1 | 0 | -1.336000 | 2.691000 | -4.759000 |
| 25 | 1 | 0 | 0.392000 | 2.338000 | -4.974000 |
| 26 | 1 | 0 | 0.364000 | 5.098000 | -2.384000 |
| 27 | 1 | 0 | 0.986000 | 4.630000 | -3.961000 |
| 28 | 1 | 0 | -0.741000 | 4.982000 | -3.748000 |
| 29 | 1 | 0 | 0.321000 | -1.834000 | 3.263000 |
| 30 | 1 | 0 | -1.402000 | -1.740000 | 2.859000 |
| 31 | 1 | 0 | 0.158000 | -1.913000 | -1.048000 |
| 32 | 1 | 0 | -1.535000 | -1.410000 | -1.160000 |
| 33 | 1 | 0 | -0.248000 | -4.450000 | 3.060000 |
| 34 | 1 | 0 | -1.847000 | -4.149000 | 2.349000 |
| 35 | 1 | 0 | -0.645000 | -5.086000 | 1.462000 |
| 36 | 1 | 0 | 3.017000 | -3.594000 | 1.396000 |
| 37 | 1 | 0 | 1.891000 | -4.003000 | 2.696000 |
| 38 | 1 | 0 | 2.316000 | -2.291000 | 2.357000 |

| Conformer **4c** | | Standard Orientation (Ångstroms) | | | |
|---|---|---|---|---|---|
| Center Number | Atom | Type | X | Y | Z |
| 1 | 6 | 0 | 0.114000 | 2.127000 | 0.669000 |
| 2 | 6 | 0 | 0.322000 | 2.084000 | 2.033000 |
| 3 | 6 | 0 | 0.545000 | 0.830000 | 2.604000 |
| 4 | 6 | 0 | 0.549000 | -0.339000 | 1.806000 |
| 5 | 6 | 0 | 0.355000 | -0.264000 | 0.414000 |
| 6 | 6 | 0 | 0.127000 | 0.989000 | -0.127000 |
| 7 | 8 | 0 | -0.109000 | 1.260000 | -1.454000 |
| 8 | 6 | 0 | -0.282000 | 2.646000 | -1.554000 |
| 9 | 6 | 0 | -0.149000 | 3.267000 | -0.198000 |
| 10 | 6 | 0 | -0.458000 | 3.265000 | -2.734000 |
| 11 | 6 | 0 | -0.566000 | 2.492000 | -4.024000 |
| 12 | 6 | 0 | -0.627000 | 4.750000 | -2.911000 |
| 13 | 8 | 0 | -0.283000 | 4.421000 | 0.161000 |
| 14 | 6 | 0 | 0.825000 | -1.683000 | 2.433000 |

| | | | | | |
|---|---|---|---|---|---|
| 15 | 6 | 0 | 0.386000 | -2.869000 | 1.542000 |
| 16 | 8 | 0 | 0.871000 | -2.655000 | 0.213000 |
| 17 | 6 | 0 | 0.338000 | -1.500000 | -0.438000 |
| 18 | 6 | 0 | -1.138000 | -3.069000 | 1.588000 |
| 19 | 8 | 0 | 1.031000 | -4.022000 | 2.117000 |
| 20 | 6 | 0 | 0.936000 | -5.207000 | 1.340000 |
| 21 | 1 | 0 | 0.312000 | 2.990000 | 2.631000 |
| 22 | 1 | 0 | 0.721000 | 0.757000 | 3.676000 |
| 23 | 1 | 0 | -0.442000 | 1.413000 | -3.891000 |
| 24 | 1 | 0 | -1.549000 | 2.656000 | -4.480000 |
| 25 | 1 | 0 | 0.202000 | 2.826000 | -4.729000 |
| 26 | 1 | 0 | -0.546000 | 5.322000 | -1.987000 |
| 27 | 1 | 0 | 0.135000 | 5.134000 | -3.597000 |
| 28 | 1 | 0 | -1.612000 | 4.965000 | -3.339000 |
| 29 | 1 | 0 | 1.909000 | -1.743000 | 2.604000 |
| 30 | 1 | 0 | 0.346000 | -1.759000 | 3.417000 |
| 31 | 1 | 0 | 0.949000 | -1.339000 | -1.333000 |
| 32 | 1 | 0 | -0.684000 | -1.717000 | -0.769000 |
| 33 | 1 | 0 | -1.455000 | -3.448000 | 2.565000 |
| 34 | 1 | 0 | -1.688000 | -2.145000 | 1.385000 |
| 35 | 1 | 0 | -1.460000 | -3.791000 | 0.830000 |
| 36 | 1 | 0 | 1.500000 | -5.991000 | 1.853000 |
| 37 | 1 | 0 | 1.376000 | -5.069000 | 0.348000 |
| 38 | 1 | 0 | -0.101000 | -5.543000 | 1.254000 |
