# Supplementary material for: New Benzofuranoids and Phenylpropanoids from the Mangrove Endophytic Fungus, Aspergillus sp. ZJ-68
Source: Mar Drugs. 2019 Aug 18;17(8):478. doi: 10.3390/md17080478 (PMC6723808; doi:10.3390/md17080478)
Supplement: Supplementary file 1 [file marinedrugs-17-00478-s001.zip › Supporting Information-revision.pdf]

Supporting Information

New Benzofuranoids and Phenylpropanoids from the Mangrove Endophytic Fungus, *Aspergillus* sp. ZJ-68

Runlin Cai [1], Hongming Jiang [1], Zhenming Zang [1], Chunyuan Li [2,]* and Zhigang She [1, 3,]*

[1] School of Chemistry, Sun Yat-sen University, Guangzhou 510275, People's Republic of China; cairlin@mail2.sysu.edu.cn (R.C.); jianghm7@mail2.sysu.edu.cn (H.J.); zangzhm@mail2.sysu.edu.cn (Z.Z.); cesshzhg@mail.sysu.edu.cn (Z. S.)

[2] College of Materials and Energy, South China Agricultural University, Guangzhou 510642, People's Republic of China; chunyuanli@scau.edu.cn (C.L.)

[3] South China Sea Bio-Resource Exploitation and Utilization Collaborative Innovation Center, Guangzhou 510006, People's Republic of China

* Correspondence: chunyuanli@scau.edu.cn (C.L); cesshzhg@mail.sysu.edu.cn (Z. S.); Tel./Fax: +86-020-84113356 (Z. S.)

The List of Contents

No.	Content	page
**1**	Figure S1. HRESIMS spectrum of **1**	1
**2**	Figure S2. ^{1}H NMR spectrum of **1** in $CDCl_3$	1
**3**	Figure S3. ^{13}C NMR spectrum of **1** in $CDCl_3$	2
**4**	Figure S4. DEPT spectrum of **1** in $CDCl_3$	2
**5**	Figure S5. ^{1}H-^{1}H COSY spectrum of **1** in $CDCl_3$	3
**6**	Figure S6. HSQC spectrum of **1** in $CDCl_3$	3
**7**	Figure S7. HMBC spectrum of **1** in $CDCl_3$	4
**8**	Figure S8. IR spectrum of **1**	4
**9**	Figure S9. UV data of **1** in MeOH	5
**10**	Figure S10. HRESIMS spectrum of **2**	5
**11**	Figure S11. ^{1}H NMR spectrum of **2** in $CDCl_3$	6
**12**	Figure S12. ^{13}C NMR spectrum of **2** in $CDCl_3$	6
**13**	Figure S13. DEPT spectrum of **2** in $CDCl_3$	7
**14**	Figure S14. ^{1}H-^{1}H COSY spectrum of **2** in $CDCl_3$	7
**15**	Figure S15. HSQC spectrum of **2** in $CDCl_3$	8
**16**	Figure S16. HMBC spectrum of **2** in $CDCl_3$	8
**17**	Figure S17. IR spectrum of **2**	9
**18**	Figure S18. UV data of **2** in MeOH	9
**19**	Figure S19. HRESIMS spectrum of **3**	10
**20**	Figure S20. ^{1}H NMR spectrum of **3** in $CDCl_3$	10
**21**	Figure S21. ^{13}C NMR spectrum of **3** in $CDCl_3$	11
**22**	Figure S22. DEPT spectrum of **3** in $CDCl_3$	11
**23**	Figure S23. ^{1}H-^{1}H COSY spectrum of **3** in $CDCl_3$	12
**24**	Figure S24. HSQC spectrum of **3** in $CDCl_3$	12
**25**	Figure S25. HMBC spectrum of **3** in $CDCl_3$	13
**26**	Figure S26. IR spectrum of **3**	13
**27**	Figure S27. UV data of **3** in MeOH	14

**28**	Figure S28. HRESIMS spectrum of **4**	14
**29**	Figure S29. ^{1}H NMR spectrum of **4** in $CDCl_3$	15
**30**	Figure S30. ^{13}C NMR spectrum of **4** in $CDCl_3$	15
**31**	Figure S31. ^{1}H-^{1}H COSY spectrum of **4** in $CDCl_3$	16
**32**	Figure S32. HSQC spectrum of **4** in $CDCl_3$	16
**33**	Figure S33. HMBC spectrum of **4** in $CDCl_3$	17
**34**	Figure S34. IR spectrum of **4**	17
**35**	Figure S35. UV data of **4** in MeOH	18
**36**	Figure S36. HRESIMS spectrum of **5**	18
**37**	Figure S37. ^{1}H NMR spectrum of **5** in $CDCl_3$	19
**38**	Figure S38. ^{13}C NMR spectrum of **5** in $CDCl_3$	19
**39**	Figure S39. ^{1}H-^{1}H COSY spectrum of **5** in $CDCl_3$	20
**40**	Figure S40. HSQC spectrum of **5** in $CDCl_3$	20
**41**	Figure S41. HMBC spectrum of **5** in $CDCl_3$	21
**42**	Figure S42. IR spectrum of **5**	21
**43**	Figure S43. UV data of **5** in MeOH	22
**44**	Figure S44. HRESIMS spectrum of **6**	22
**45**	Figure S45. ^{1}H NMR spectrum of **6** in acetone-d_6	23
**46**	Figure S46. ^{13}C NMR spectrum of **6** in acetone-d_6	23
**47**	Figure S47. DEPT spectrum of **6** in acetone-d_6	24
**48**	Figure S48. ^{1}H-^{1}H COSY spectrum of **6** in acetone-d_6	24
**49**	Figure S49. HSQC spectrum of **6** in acetone-d_6	25
**50**	Figure S50. HMBC spectrum of **6** in acetone-d_6	25
**51**	Figure S51. IR spectrum of **6**	26
**52**	Figure S52. UV data of **6** in MeOH	26
**53**	Figure S53. HRESIMS spectrum of **7**	27
**54**	Figure S54. ^{1}H NMR spectrum of **7** in methanol-d_4	27
**55**	Figure S55. ^{13}C NMR spectrum of **7** in methanol-d_4	28
**56**	Figure S56. DEPT spectrum of **7** in methanol-d_4	28

**57**	Figure S57. ^{1}H-^{1}H COSY spectrum of **7** in methanol-d_4	29
**58**	Figure S58. HSQC spectrum of **7** in methanol-d_4	29
**59**	Figure S59. HMBC spectrum of **7** in methanol-d_4	30
**60**	Figure S60. NOESY spectrum of **7** in methanol-d_4	30
**61**	Figure S61. IR spectrum of **7**	31
**62**	Figure S62. UV data of **7** in MeOH	31
**63**	Preparation of MTPA esters of **1** by the modified Mosher's method	32
**64**	Figure S63. ESIMS spectrum of *(S)*-MTPA ester (**1a)**	32
**65**	Figure S64. ESIMS spectrum of *(R)*-MTPA ester (**1b)**	33
**66**	Figure S65. ^{1}H NMR spectrum of *(S)*-MTPA ester (**1a)** in $CDCl_3$	33
**68**	Figure S66. ^{1}H NMR spectrum of *(R)*-MTPA ester (**1b)** in $CDCl_3$	34
**69**	The ^{1}H and ^{13}C NMR data and HRMS data of compounds **8–14**	35
**70**	The rDNA sequencing data of *Aspergillus* sp. ZJ-68	37
**71**	Figure S67. Simple picture and microscopic picture of *Aspergillus* sp. ZJ-68	37
**72**	Figure S68. The chiral HPLC separation of (±)-penicisochroman A	38
**73**	ECD calculation details (Methods and Results)	39

Figure S1. HRESIMS spectrum of **1**

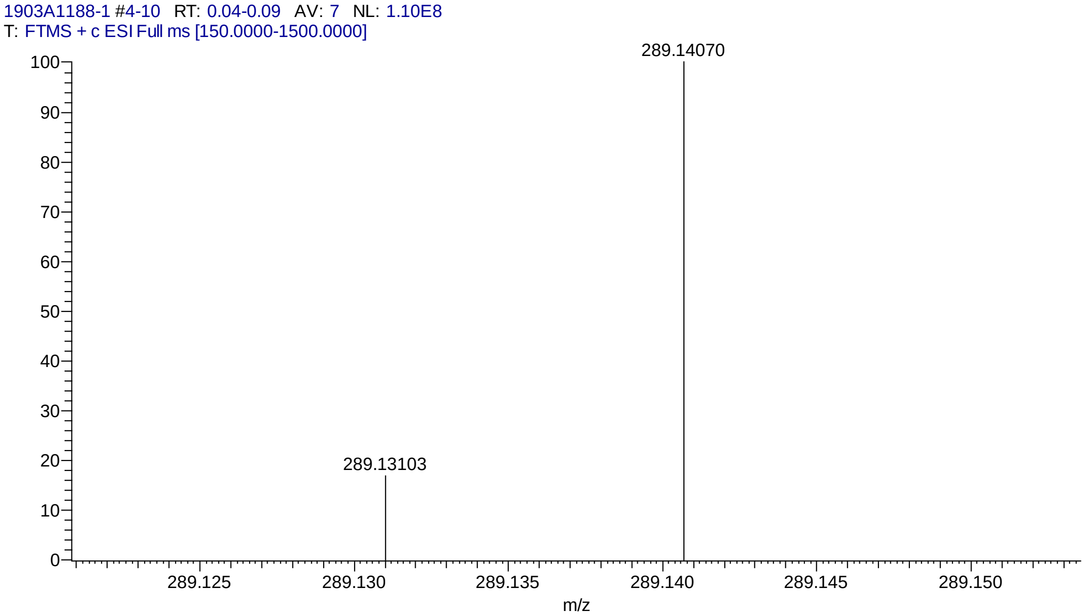

m/z	Theo. Mass	Delta (mmu)	RDB equiv.	Composition
289.14070	289.14103	-0.33	4.5	$C_{15}H_{22}O_4Na$

Figure S2. ^{1}H NMR spectrum of **1** in $CDCl_3$

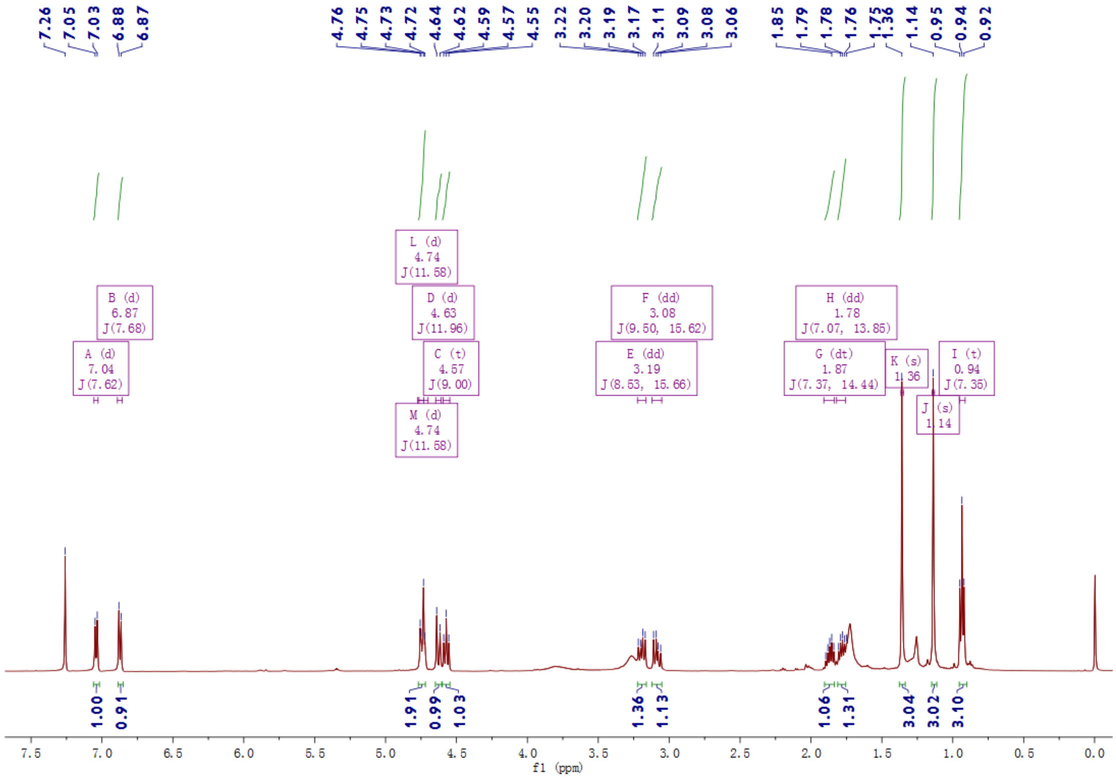

Figure S3. ^{13}C NMR spectrum of **1** in $CDCl_3$

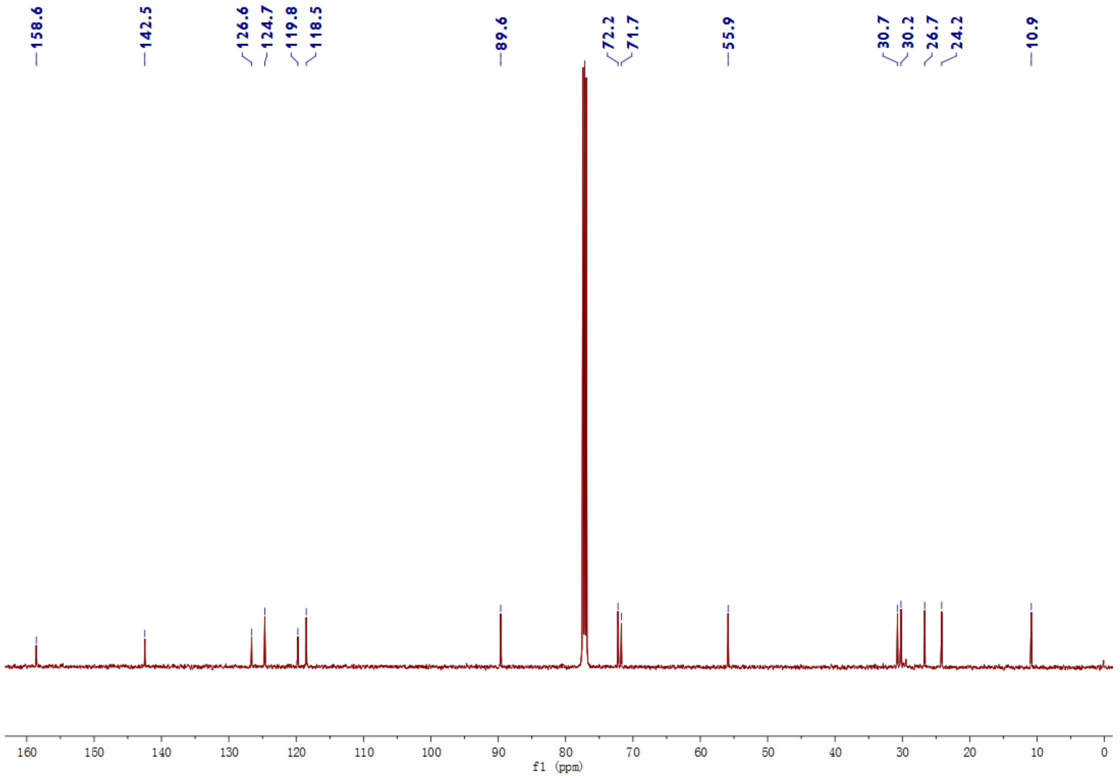

Figure S4. DEPT spectrum of **1** in $CDCl_3$

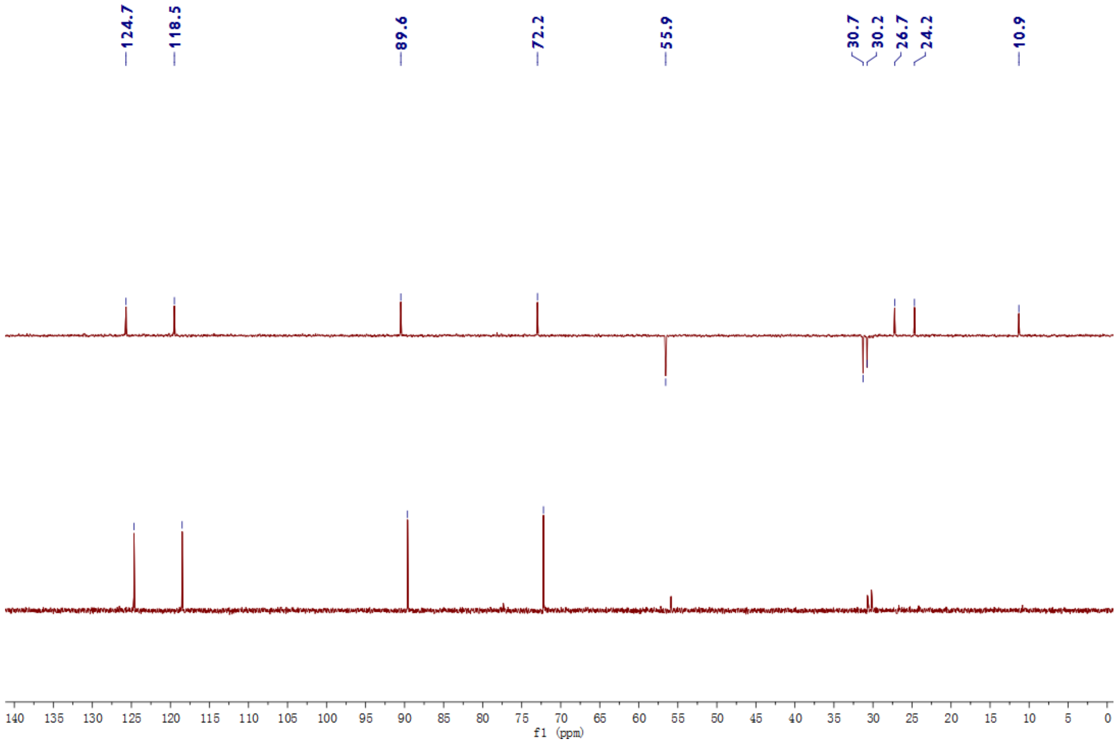

Figure S5. ^{1}H-^{1}H COSY spectrum of **1** in $CDCl_3$

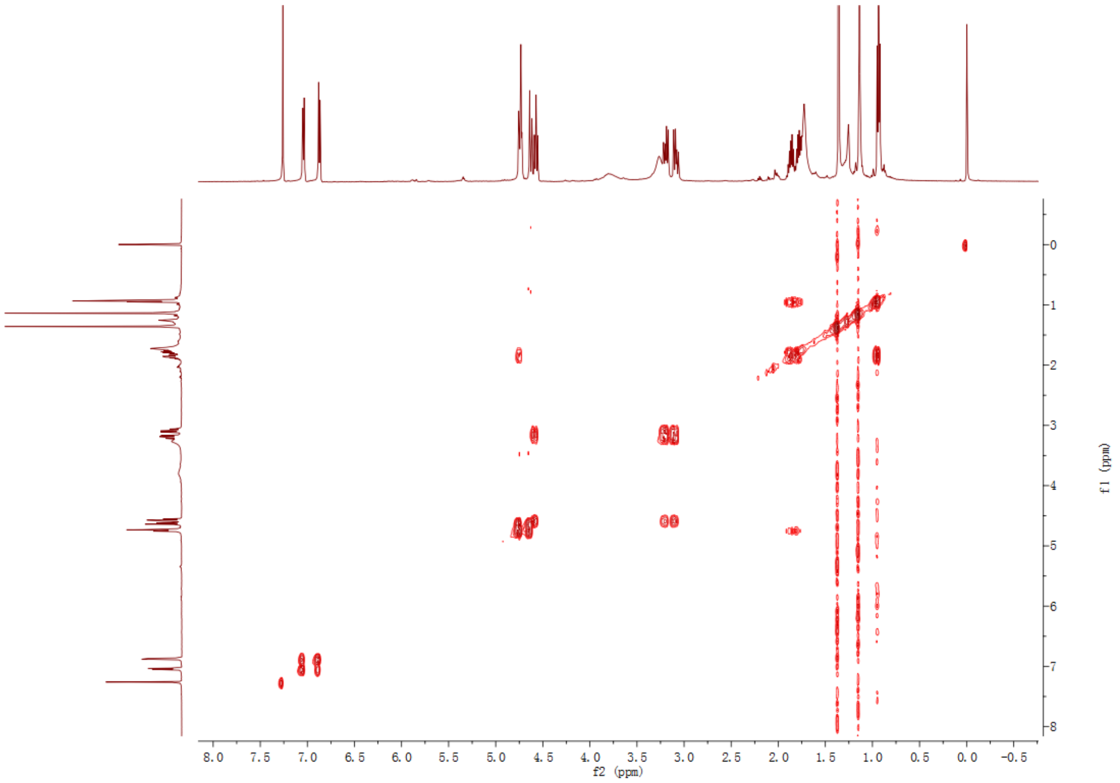

Figure S6. HSQC spectrum of **1** in $CDCl_3$

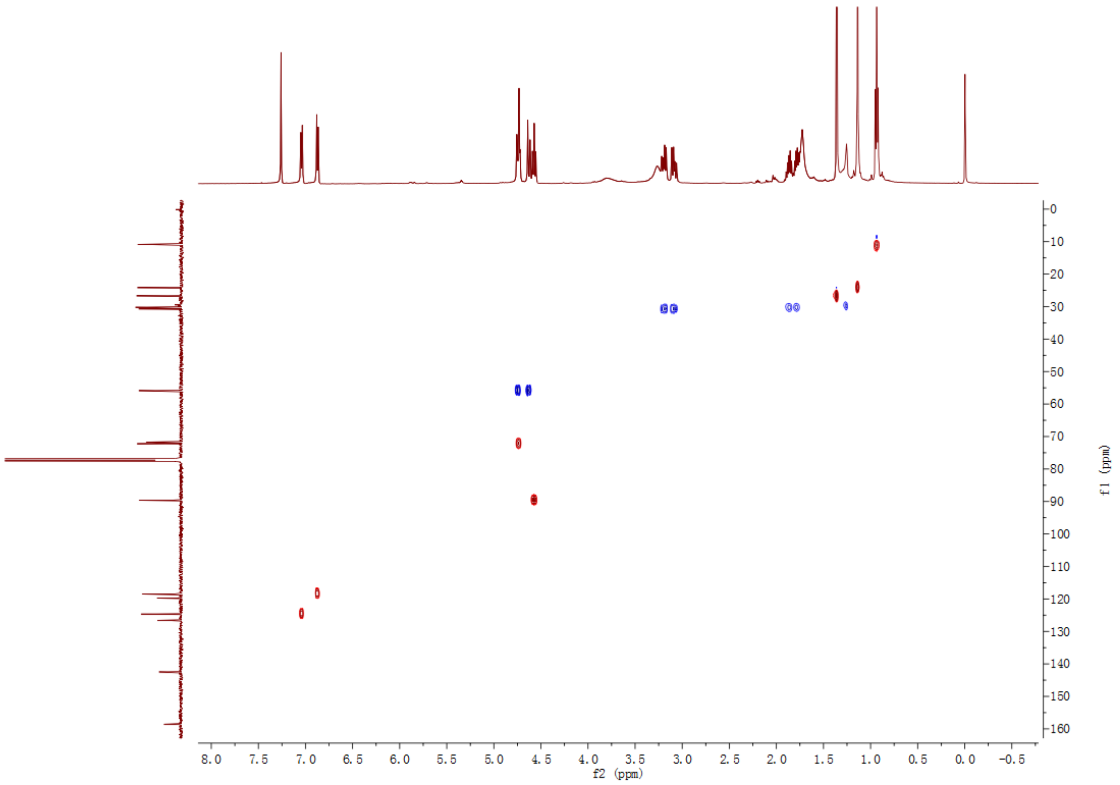

Figure S7. HMBC spectrum of **1** in $CDCl_3$

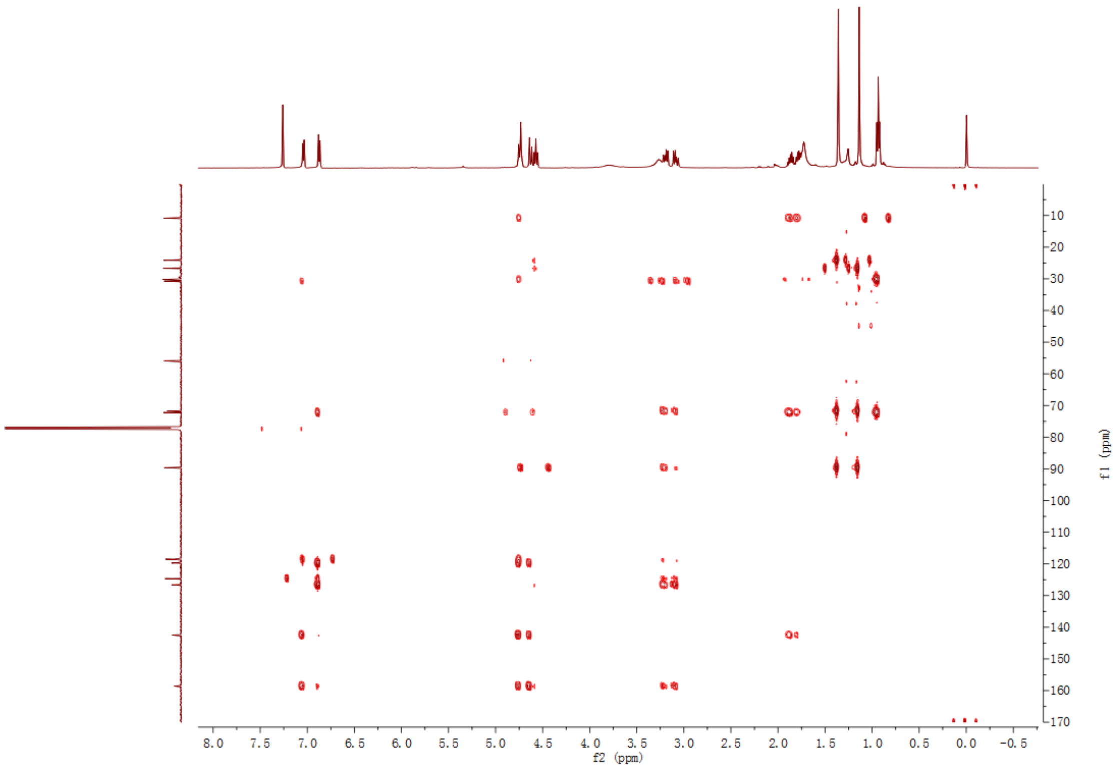

Figure S8. IR spectrum of **1**

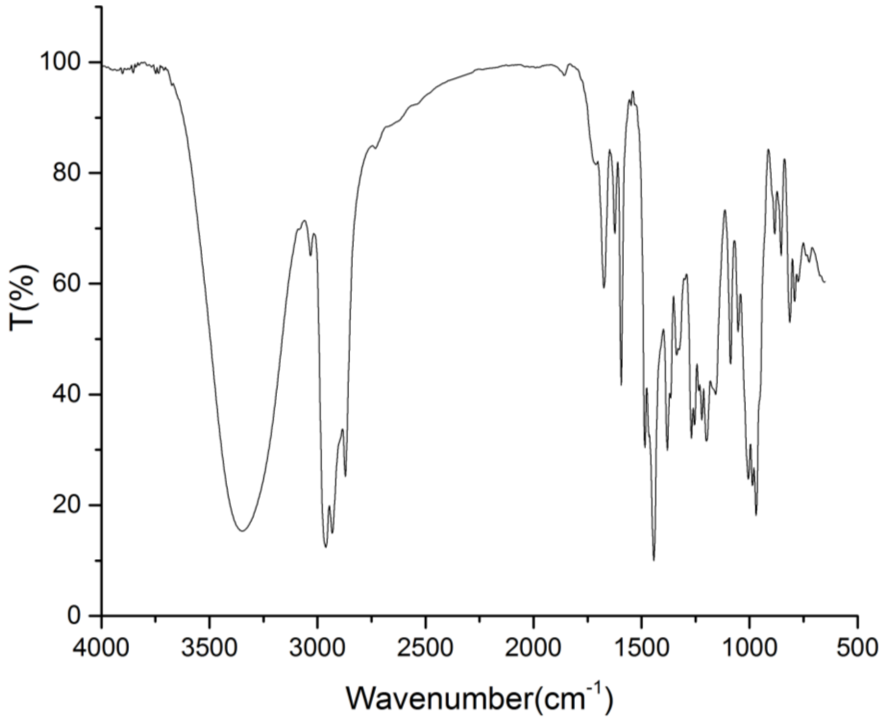

Figure S9. UV data of **1** in MeOH

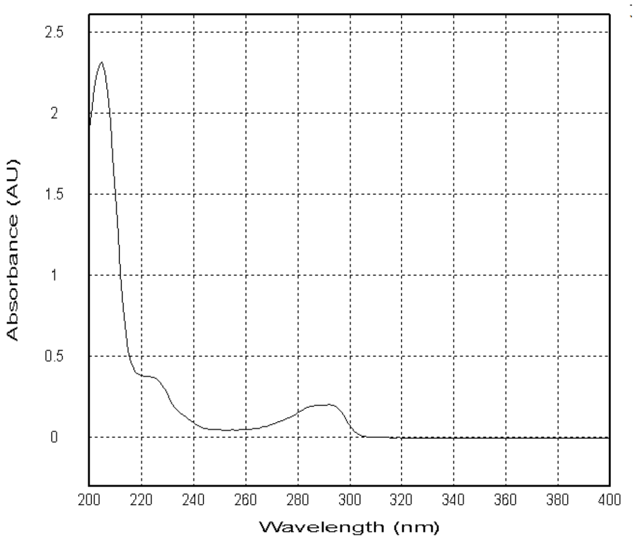

Figure S10. HRESIMS spectrum of **2**

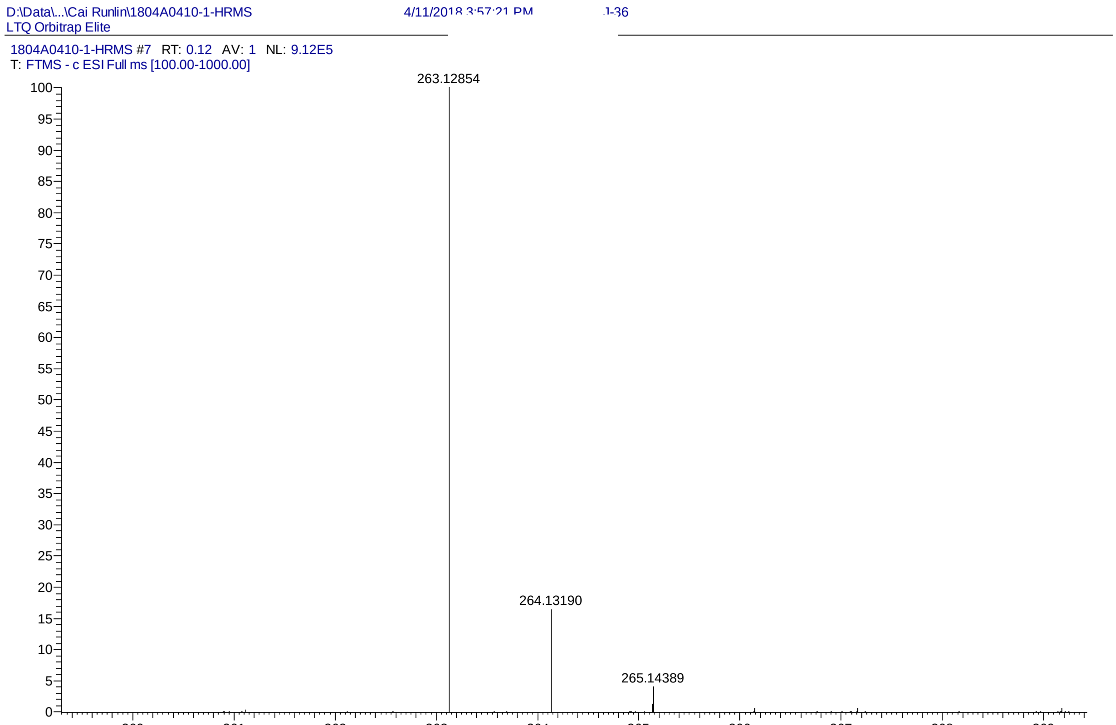

SPECTRUM - simulation :				
m/z	Theo. Mass	Delta (ppm)	RDB equiv.	Composition
263.12854	263.12888	-1.3	6.5	$C_{15} H_{19}O_4$

Figure S11. ^{1}H NMR spectrum of **2** in $CDCl_3$

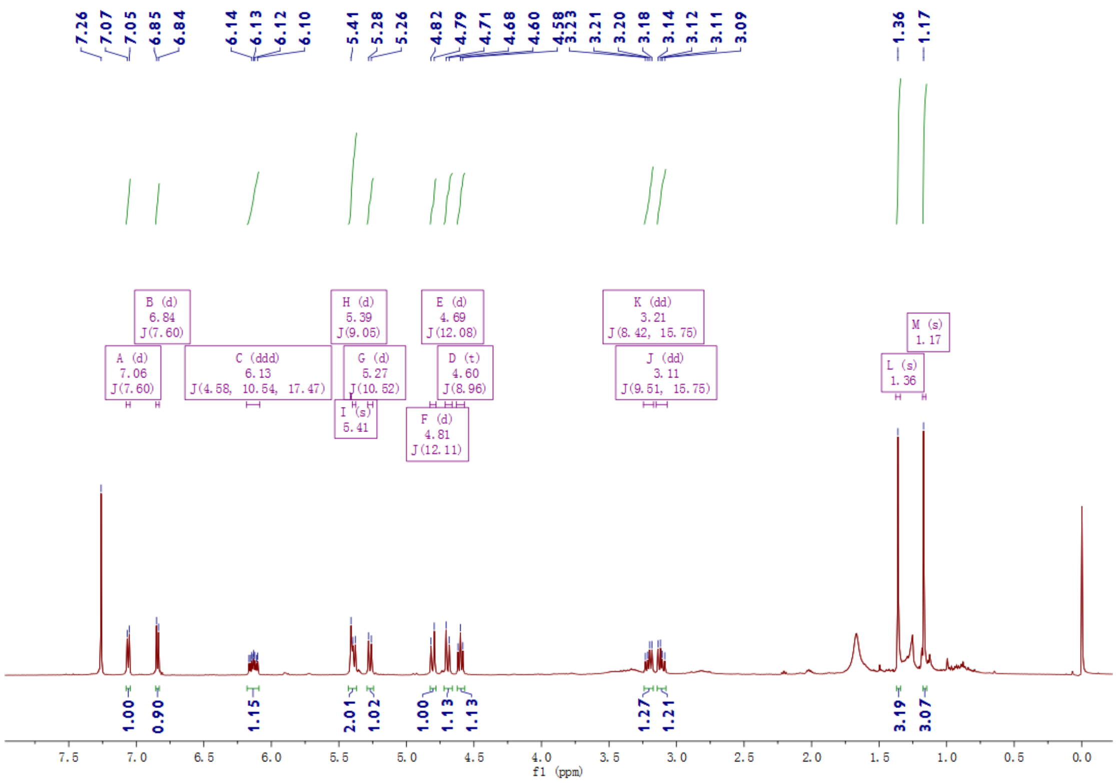

Figure S12. ^{13}C NMR spectrum of **2** in $CDCl_3$

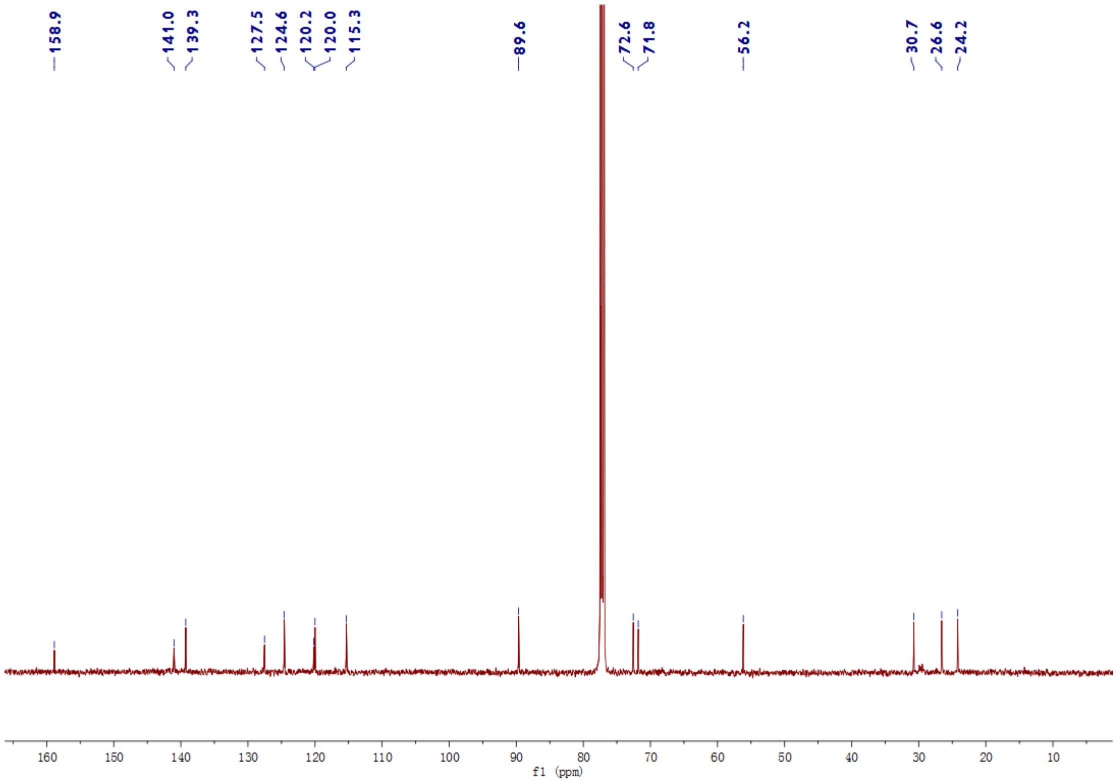

Figure S13. DEPT spectrum of **2** in $CDCl_3$

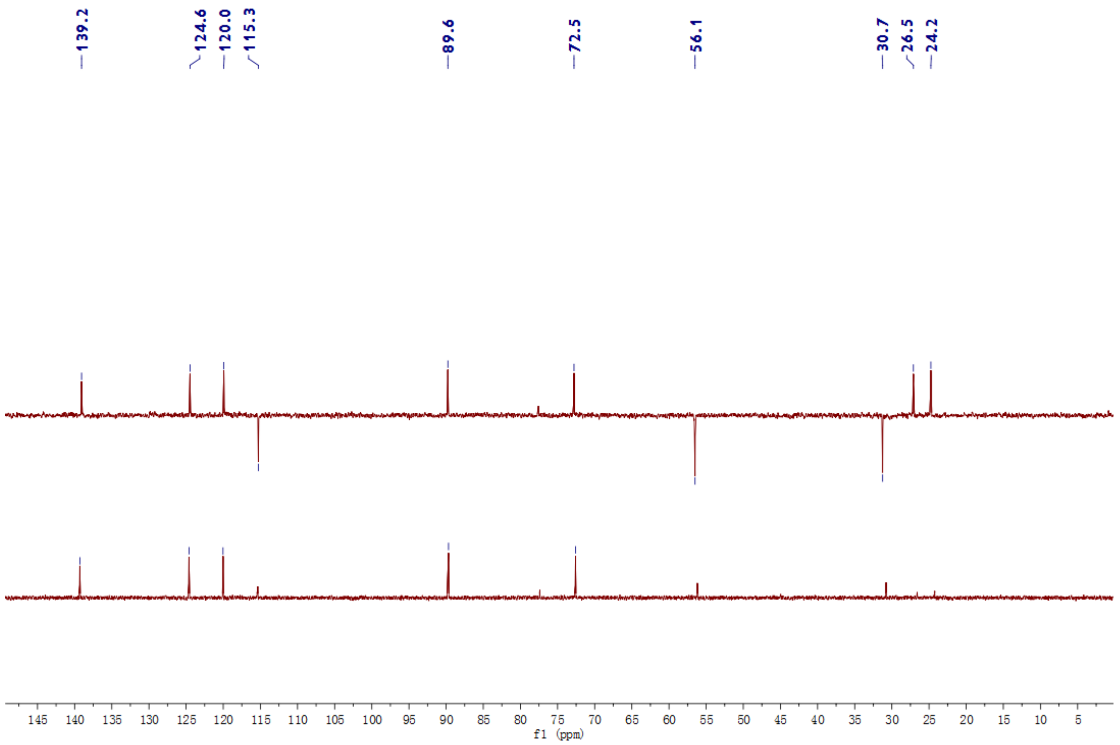

Figure S14. ^1H-^1H COSY spectrum of **2** in $CDCl_3$

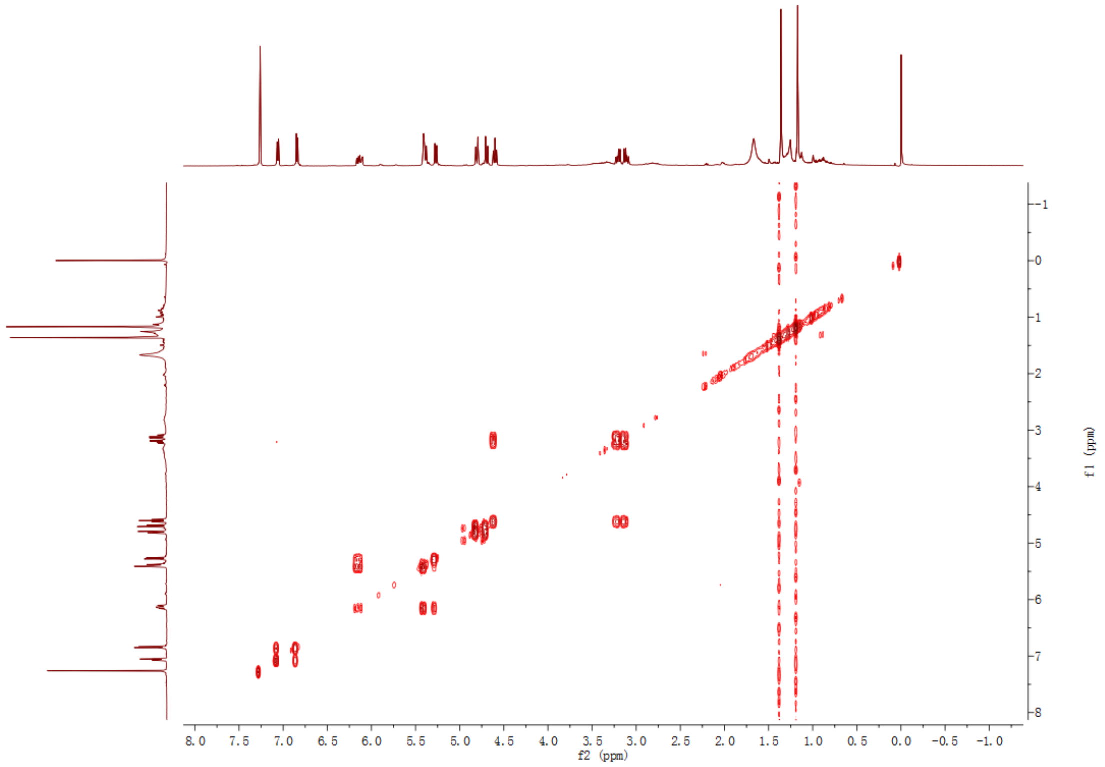

Figure S15. HSQC spectrum of **2** in $CDCl_3$

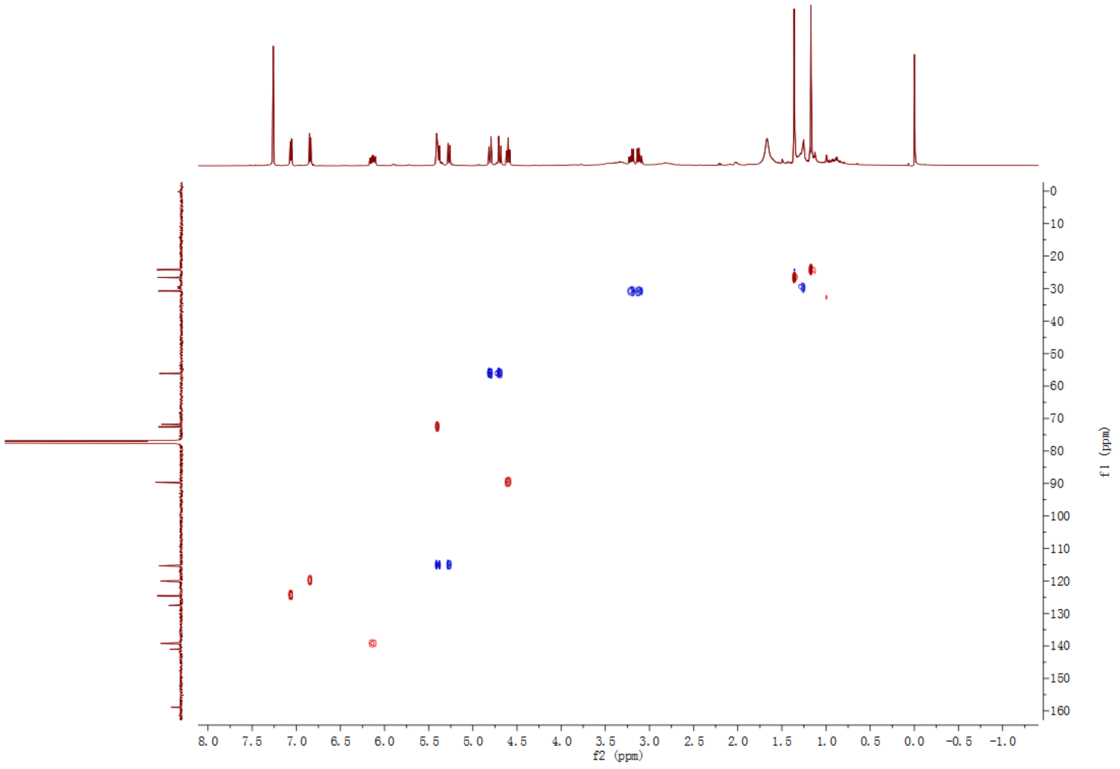

Figure S16. HMBC spectrum of **2** in $CDCl_3$

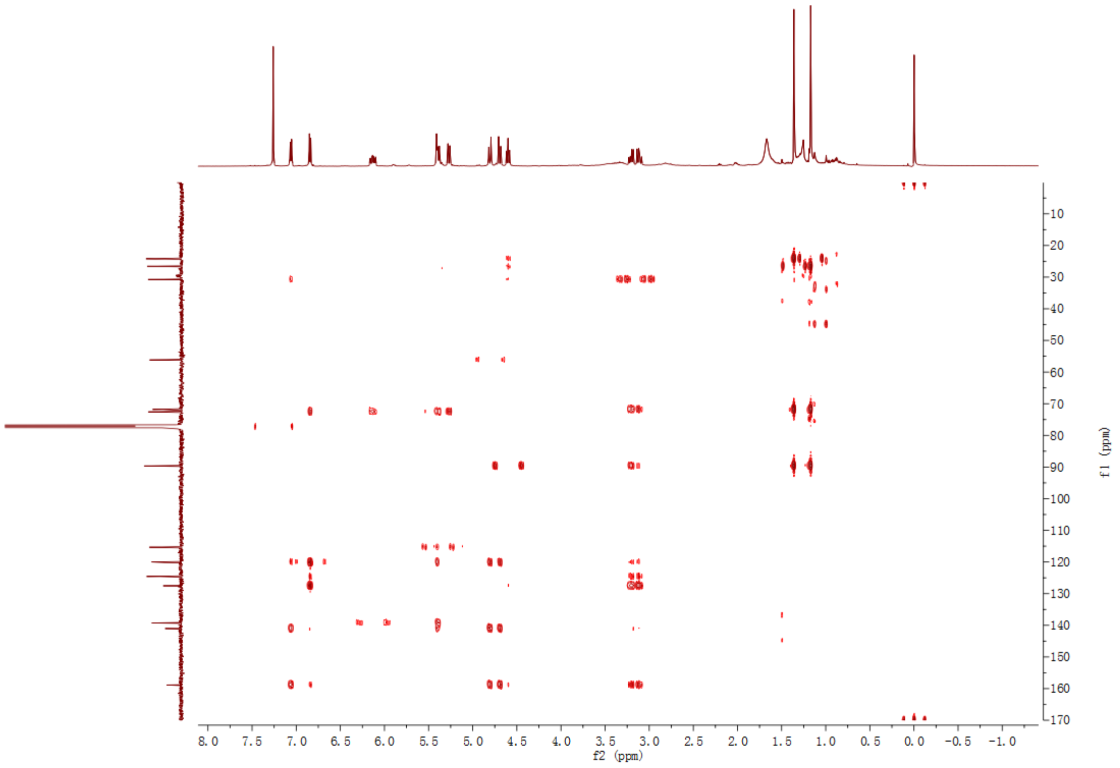

Figure S17. IR spectrum of **2**

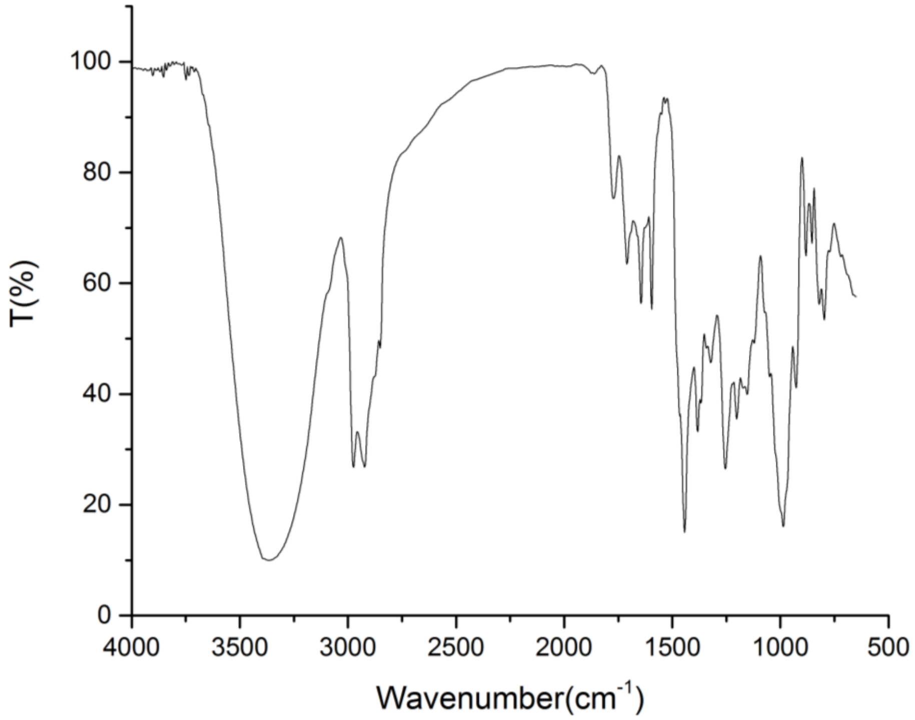

Figure S18. UV data of **2** in MeOH

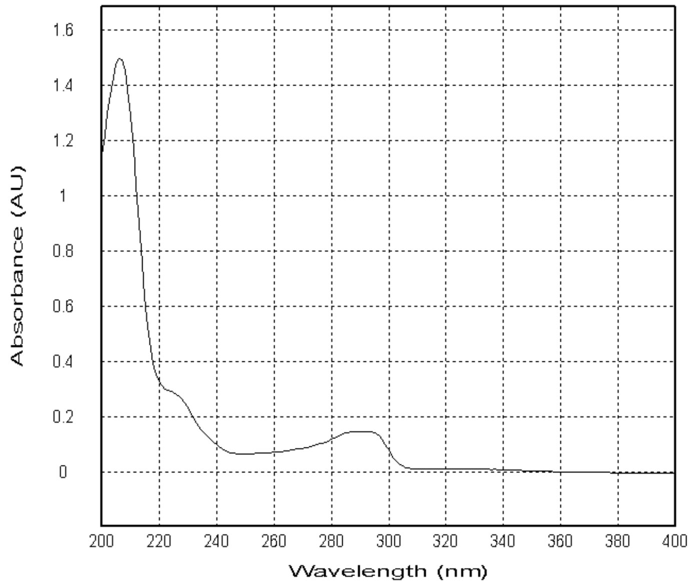

Figure S19. HRESIMS spectrum of **3**

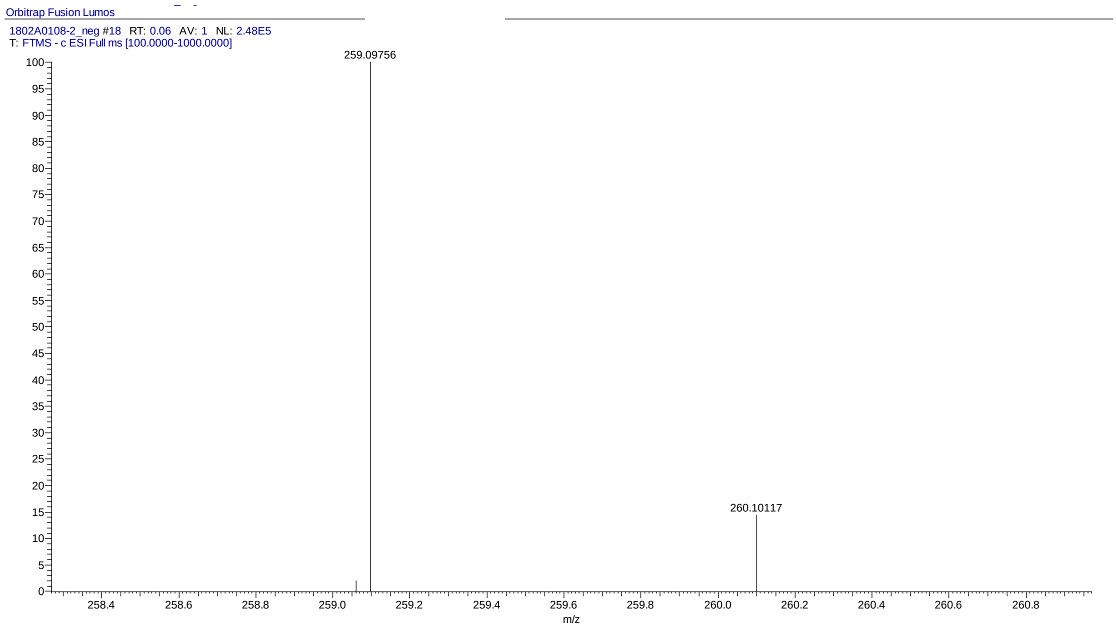

SPECTRUM - simulation :

m/z	Theo. Mass	Delta (ppm)	RDB equiv.	Composition
259.09756	259.09758	-0.09	8.5	$C_{15}H_{15}O_4$

Figure S20. ^{1}H NMR spectrum of **3** in $CDCl_3$

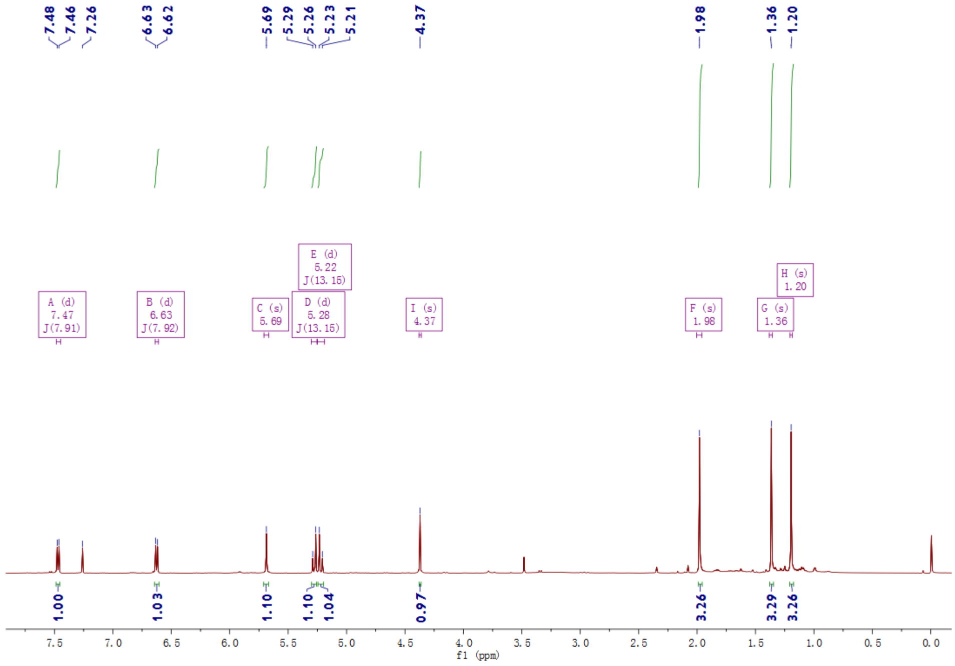

Figure S21. ^{13}C NMR spectrum of **3** in $CDCl_3$

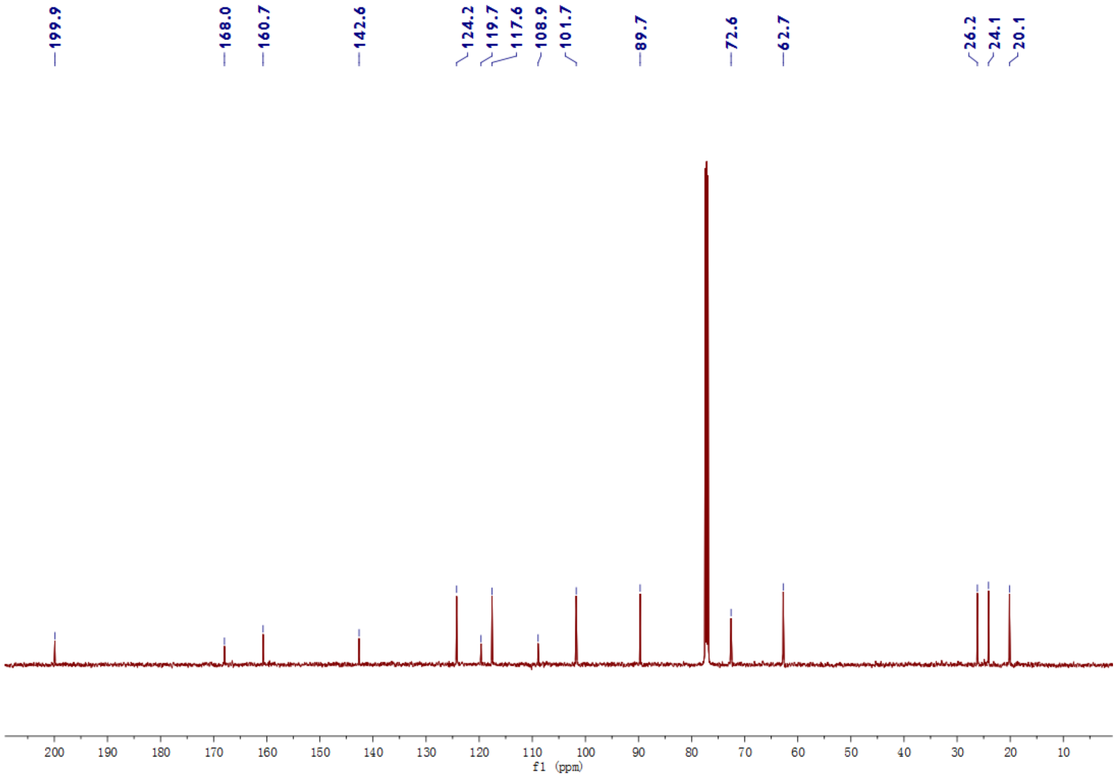

Figure S22. DEPT spectrum of **3** in $CDCl_3$

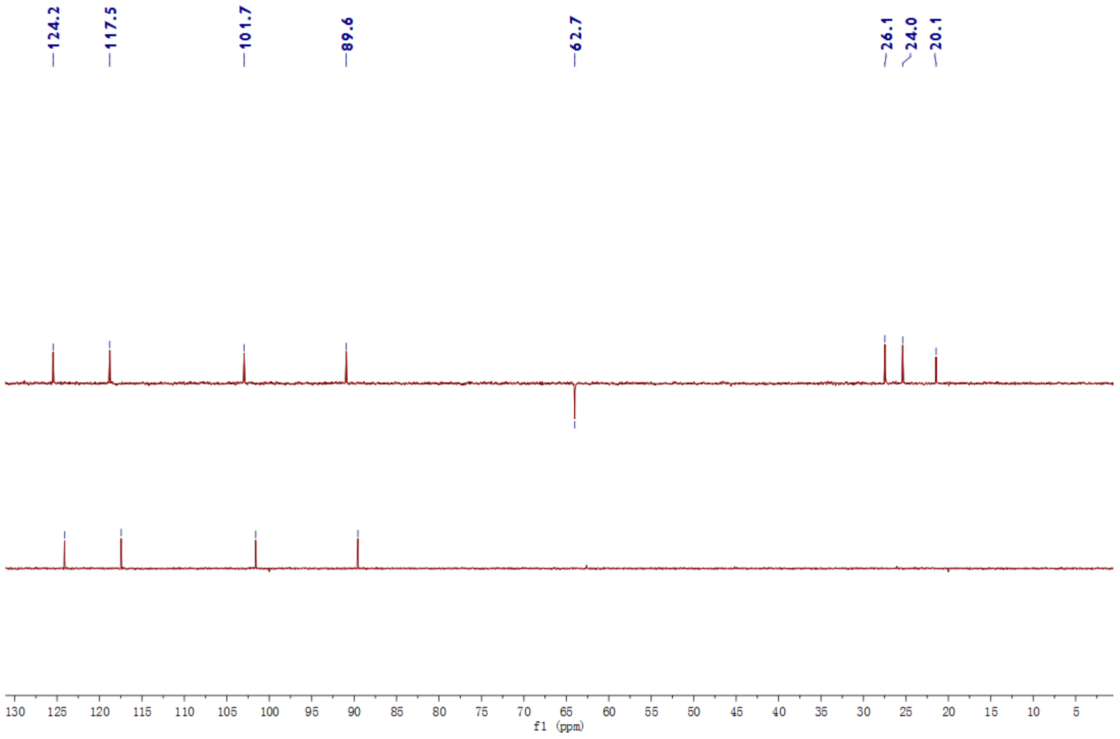

Figure S23. ^{1}H-^{1}H COSY spectrum of **3** in $CDCl_3$

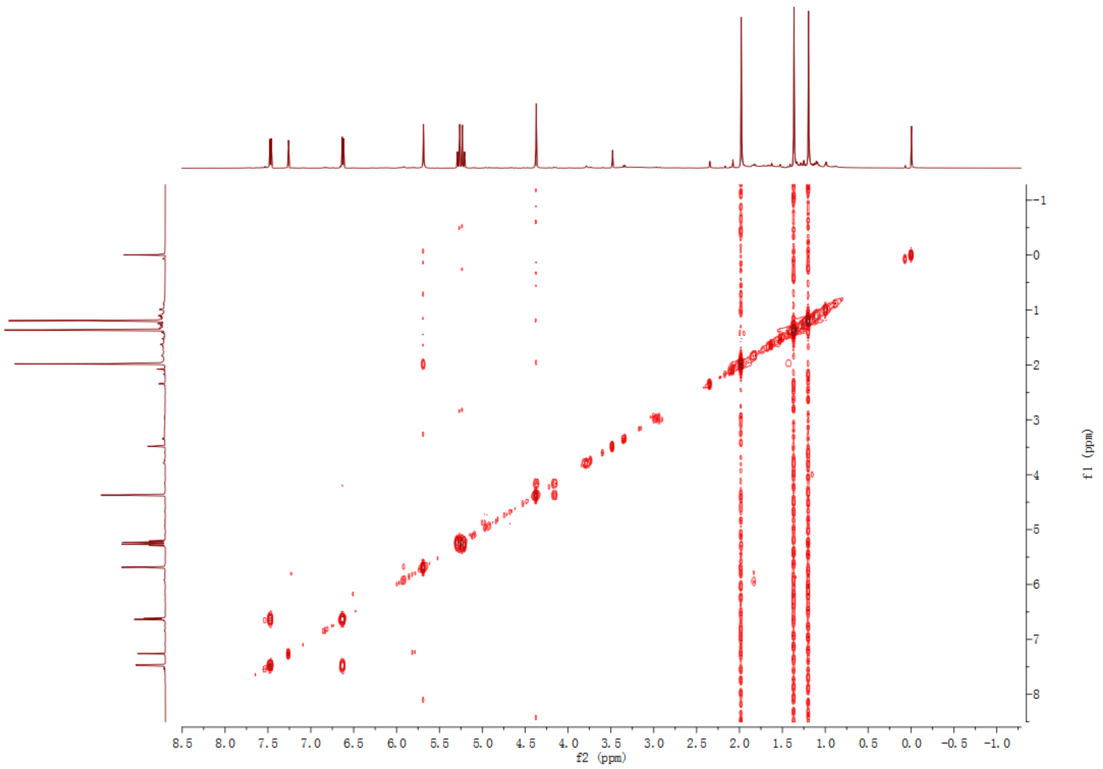

Figure S24. HSQC spectrum of **3** in $CDCl_3$

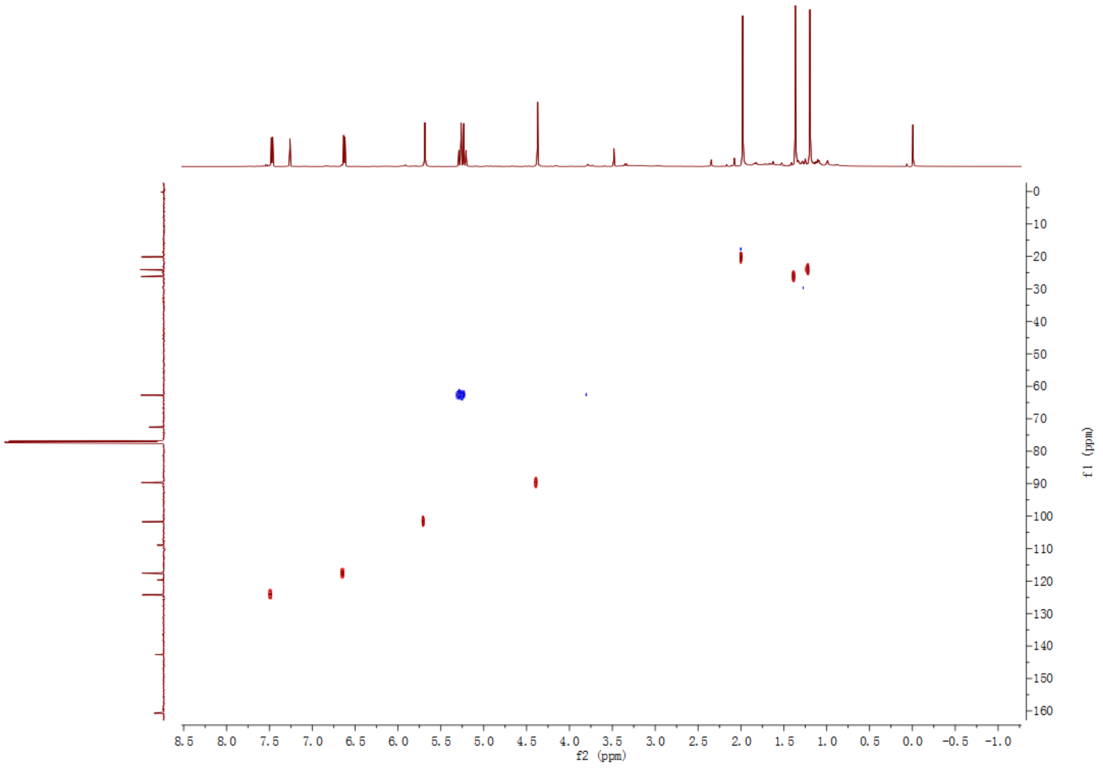

Figure S25. HMBC spectrum of **3** in $CDCl_3$

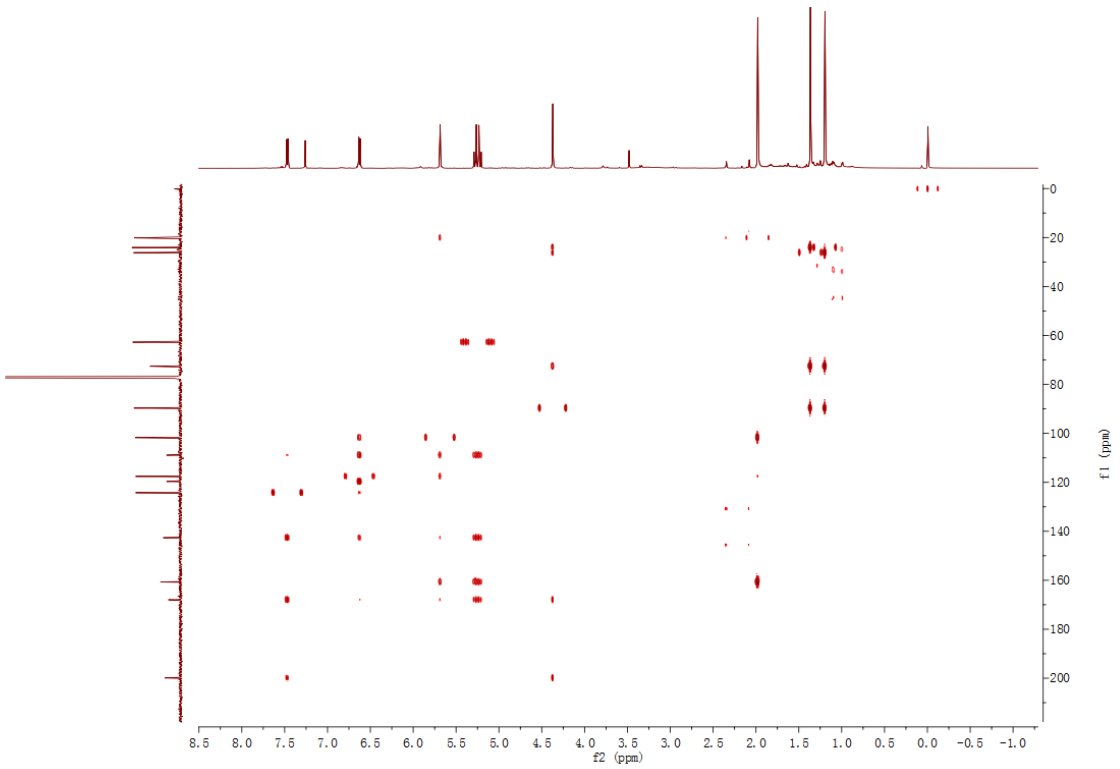

Figure S26. IR spectrum of **3**

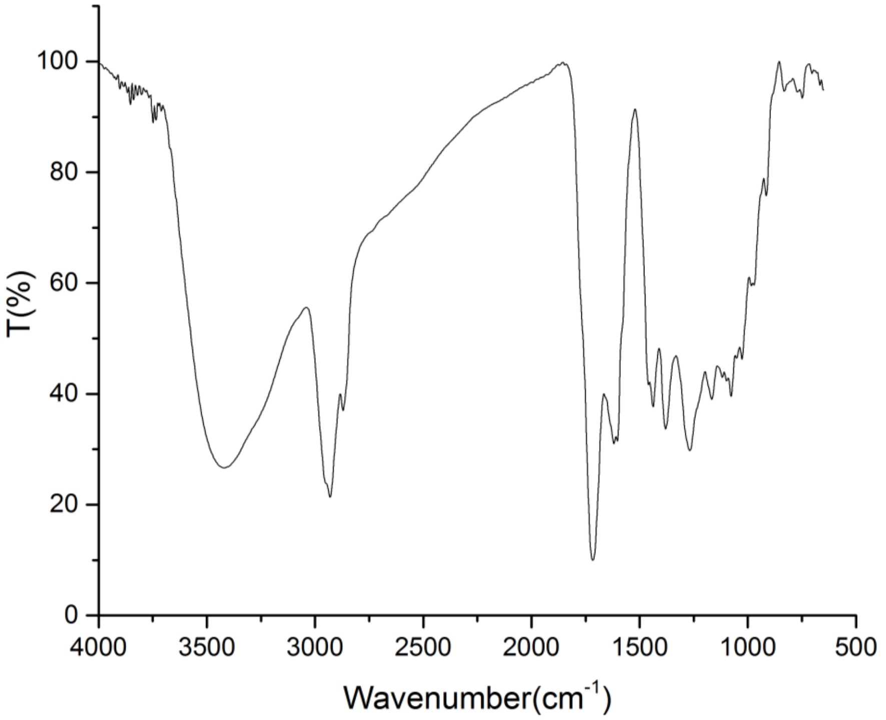

Figure S27. UV data of **3** in MeOH

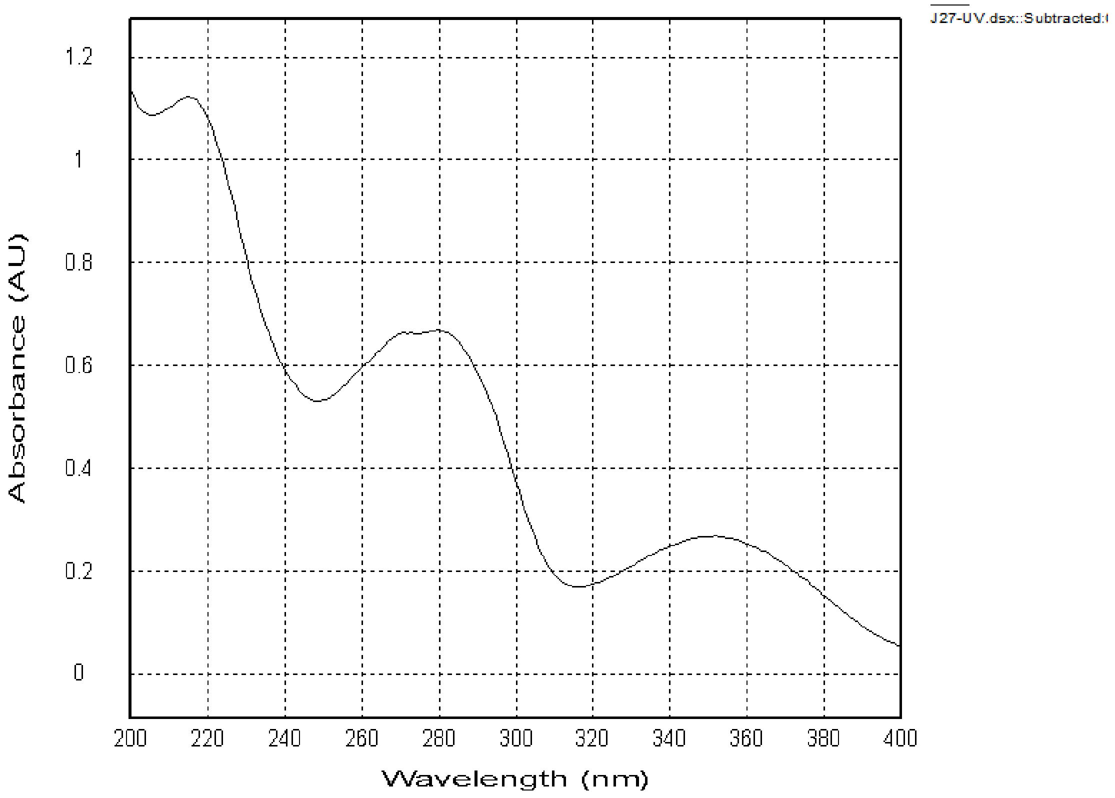

Figure S28. HRESIMS spectrum of **4**

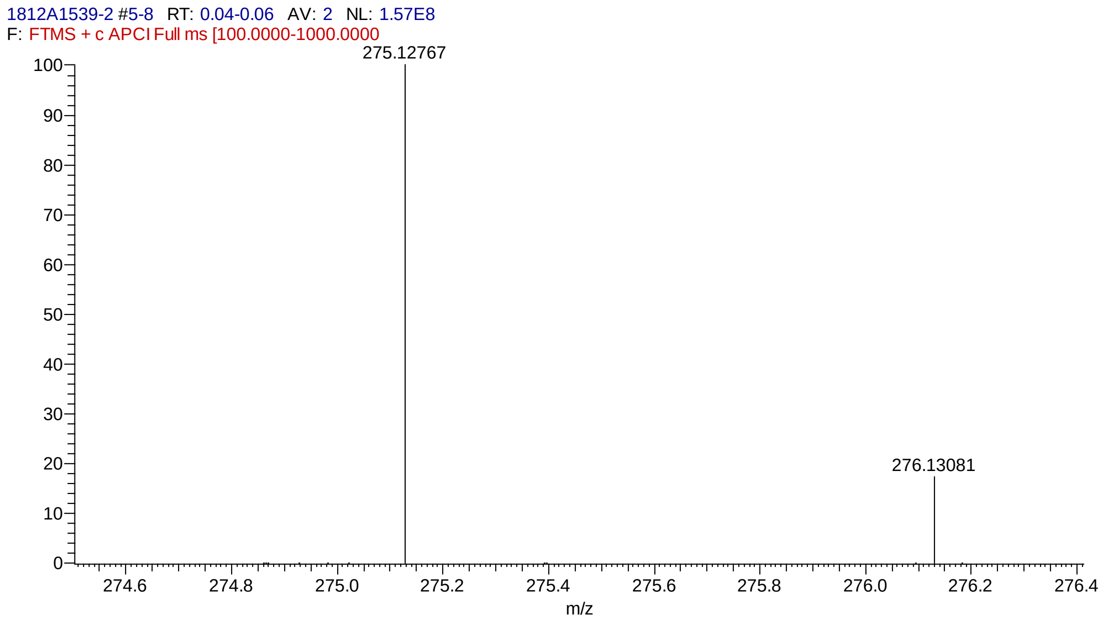

m/z	Theo. Mass	Delta (mmu)	RDB equiv.	Composition
275.12767	275.12779	-0.12	7.5	$C_{16}H_{19}O_4$

Figure S29. ^{1}H NMR spectrum of **4** in $CDCl_3$

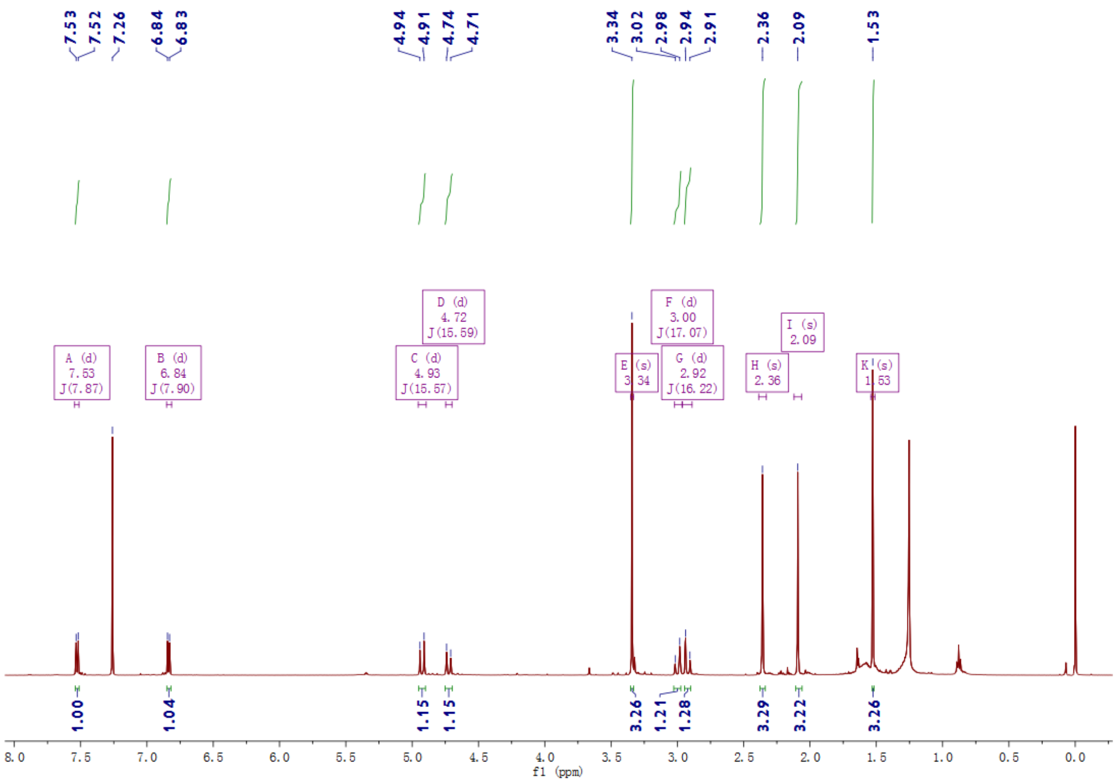

Figure S30. ^{13}C NMR spectrum of **4** in $CDCl_3$

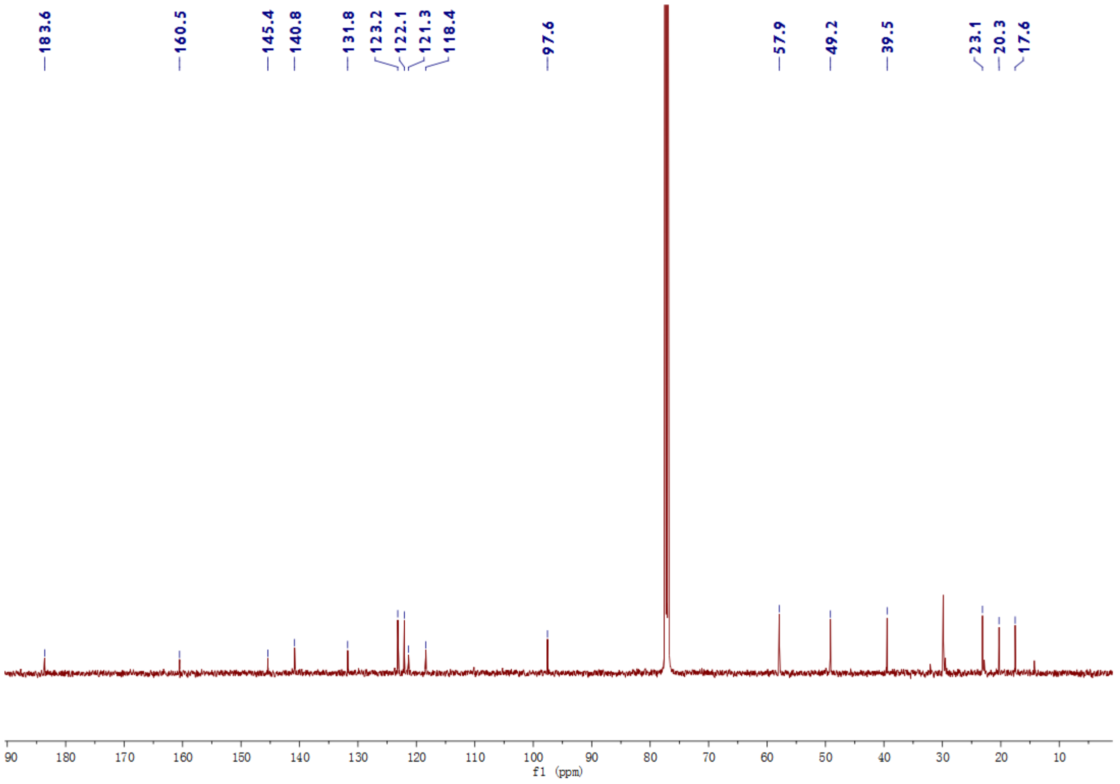

Figure S31. ^{1}H-^{1}H COSY spectrum of **4** in $CDCl_3$

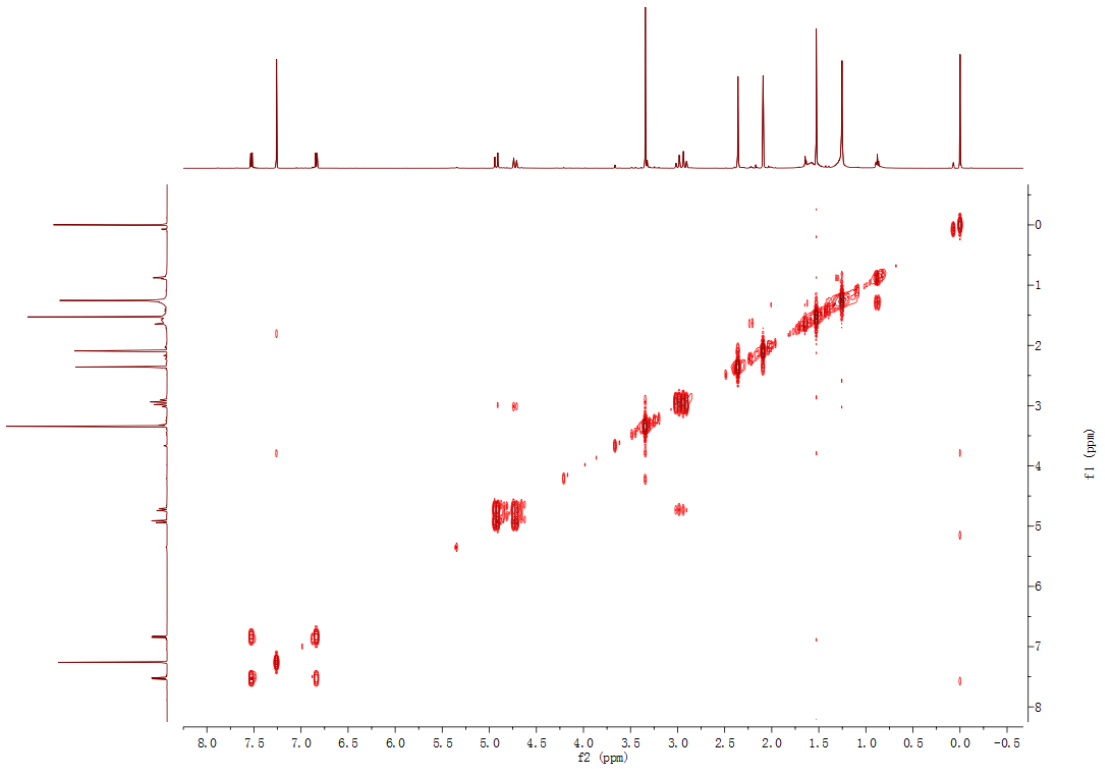

Figure S32. HSQC spectrum of **4** in $CDCl_3$

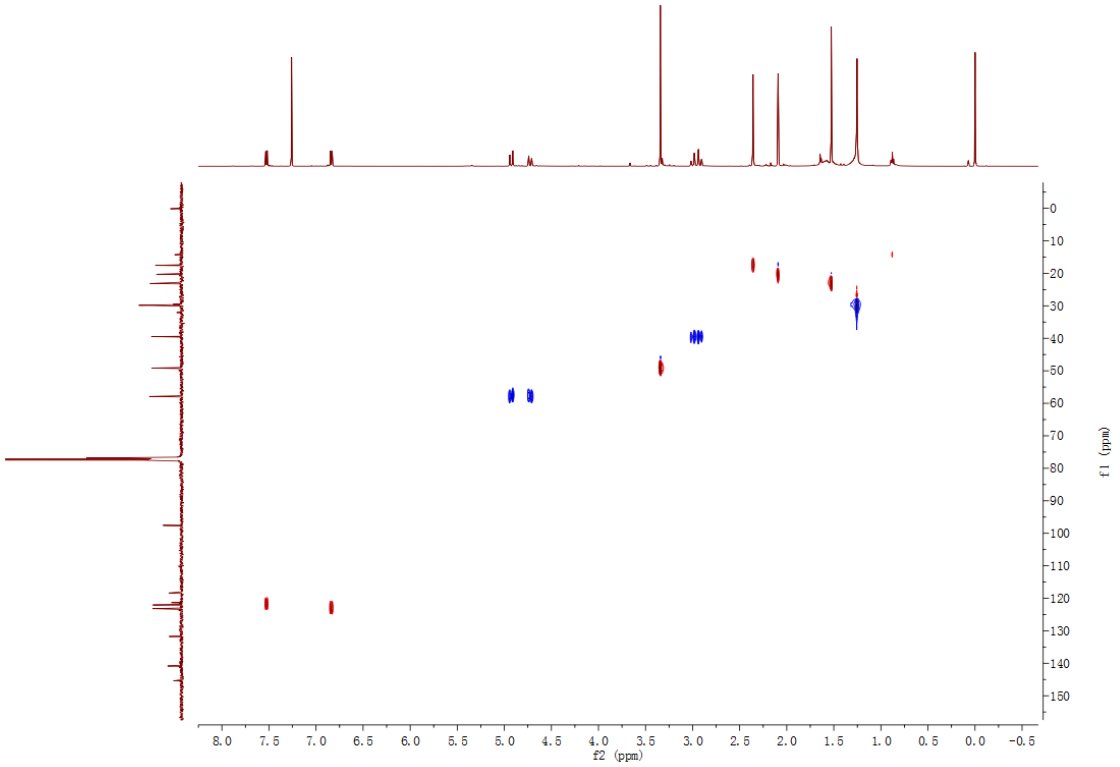

Figure S33. HMBC spectrum of **4** in $CDCl_3$

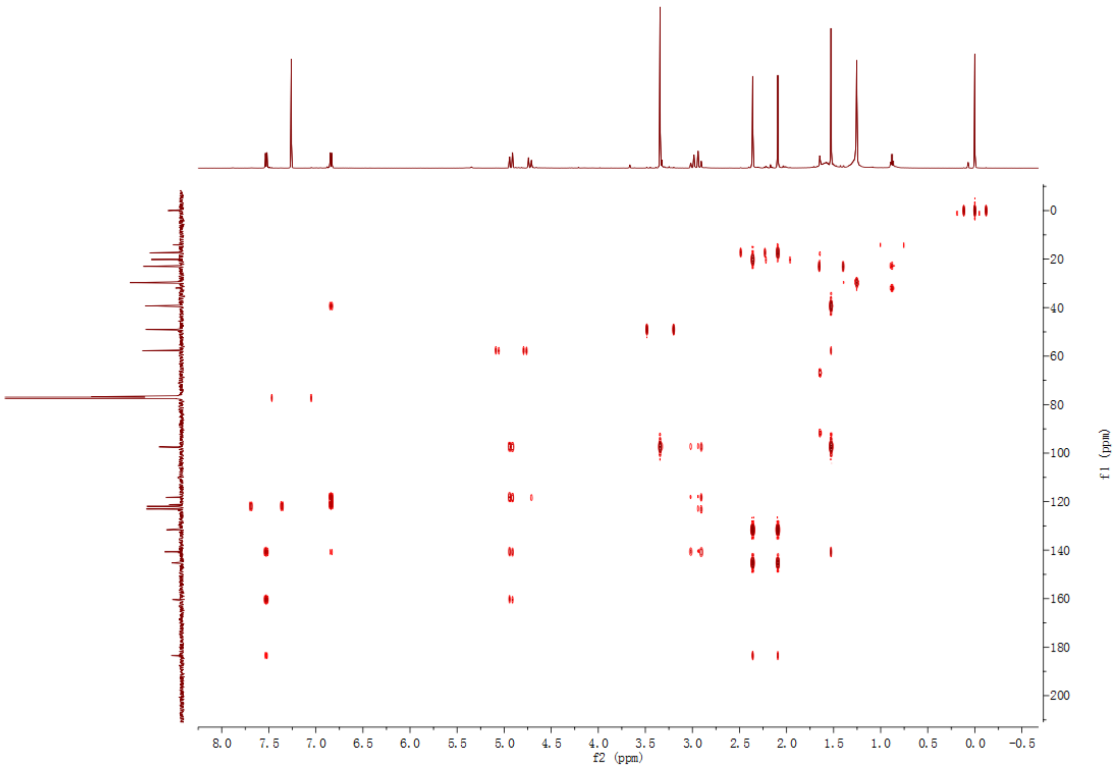

Figure S34. IR spectrum of **4**

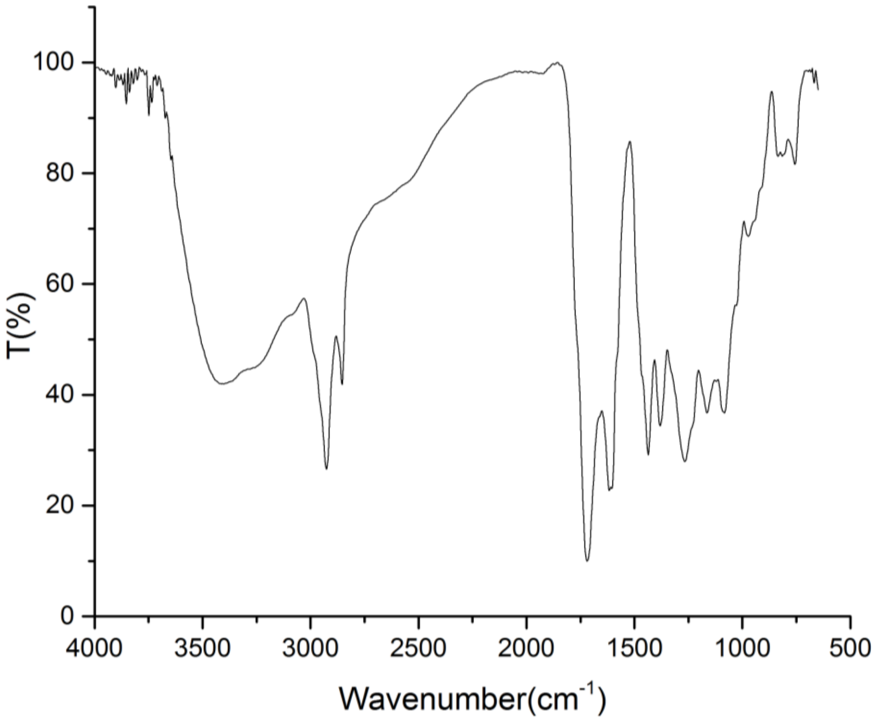

Figure S35. UV data of **4** in MeOH

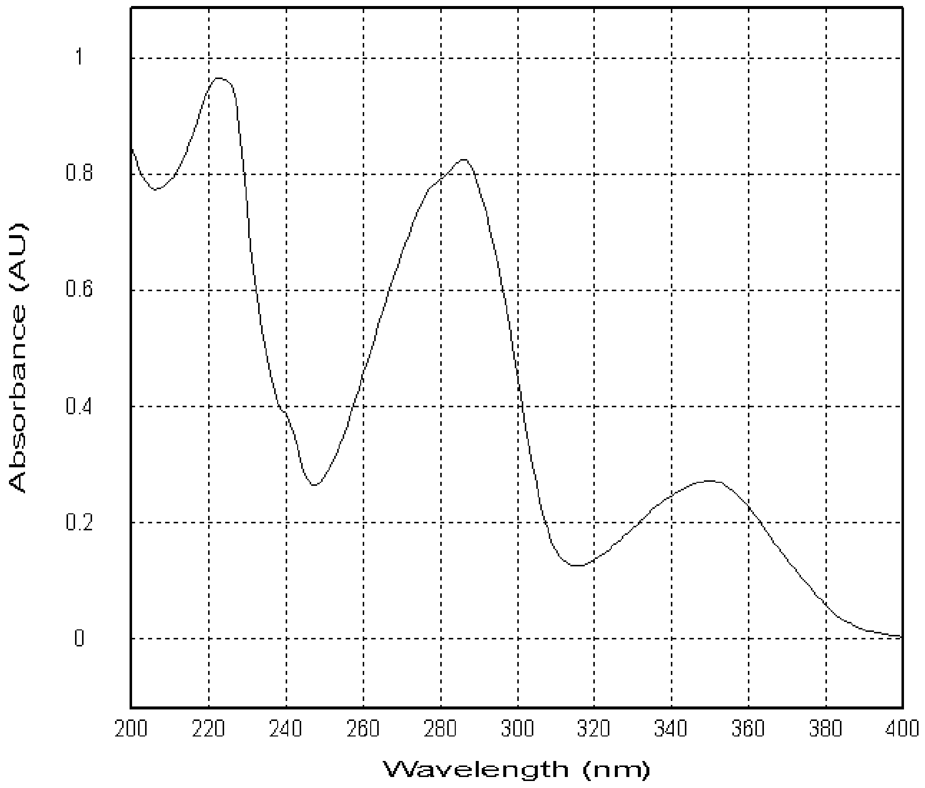

Figure S36. HRESIMS spectrum of **5**

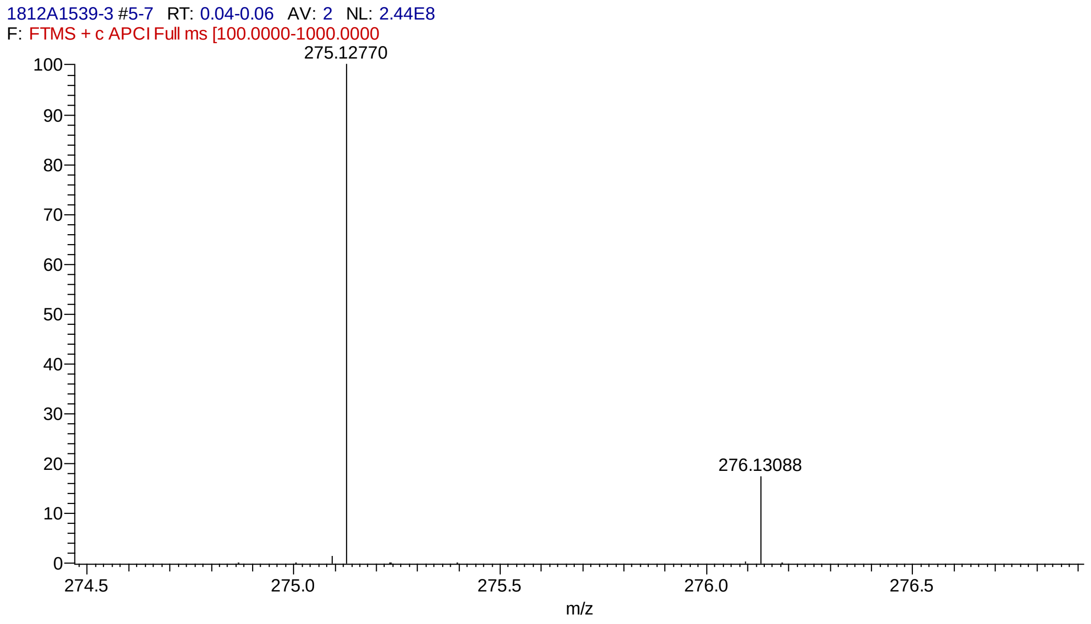

m/z	Theo. Mass	Delta (mmu)	RDB equiv.	Composition
275.12770	275.12779	-0.09	7.5	$C_{16}H_{19}O_4$

Figure S37. ^{1}H NMR spectrum of **5** in $CDCl_3$

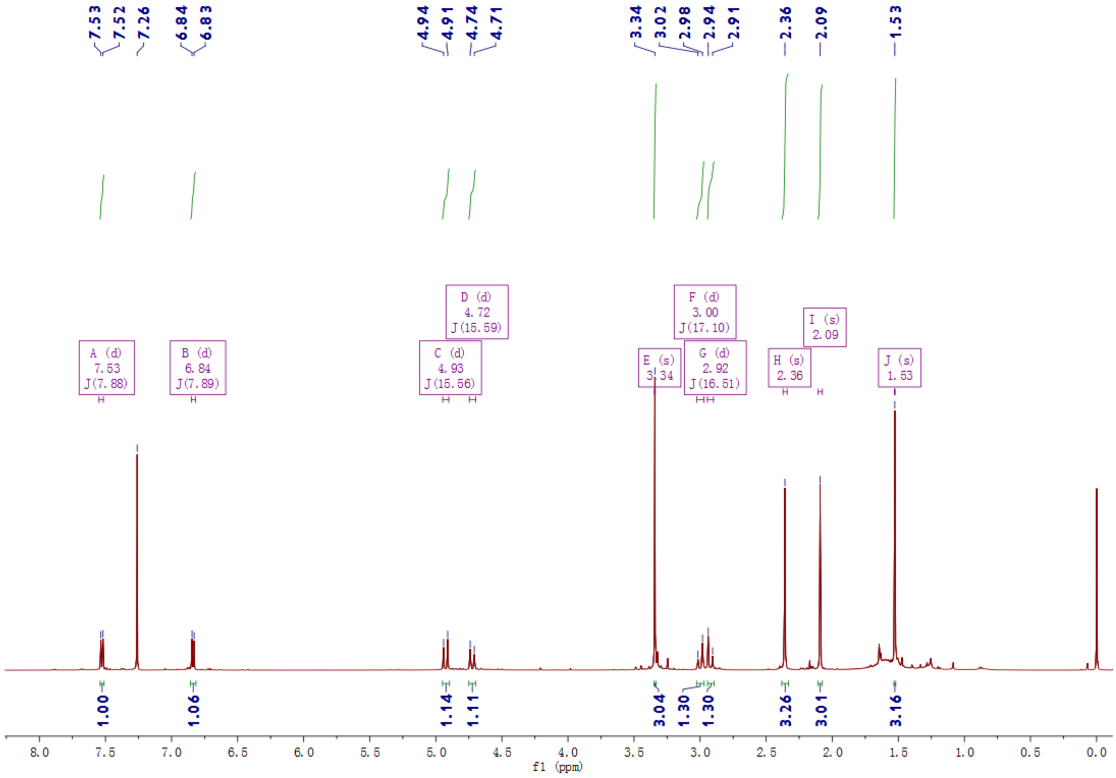

Figure S38. ^{13}C NMR spectrum of **5** in $CDCl_3$

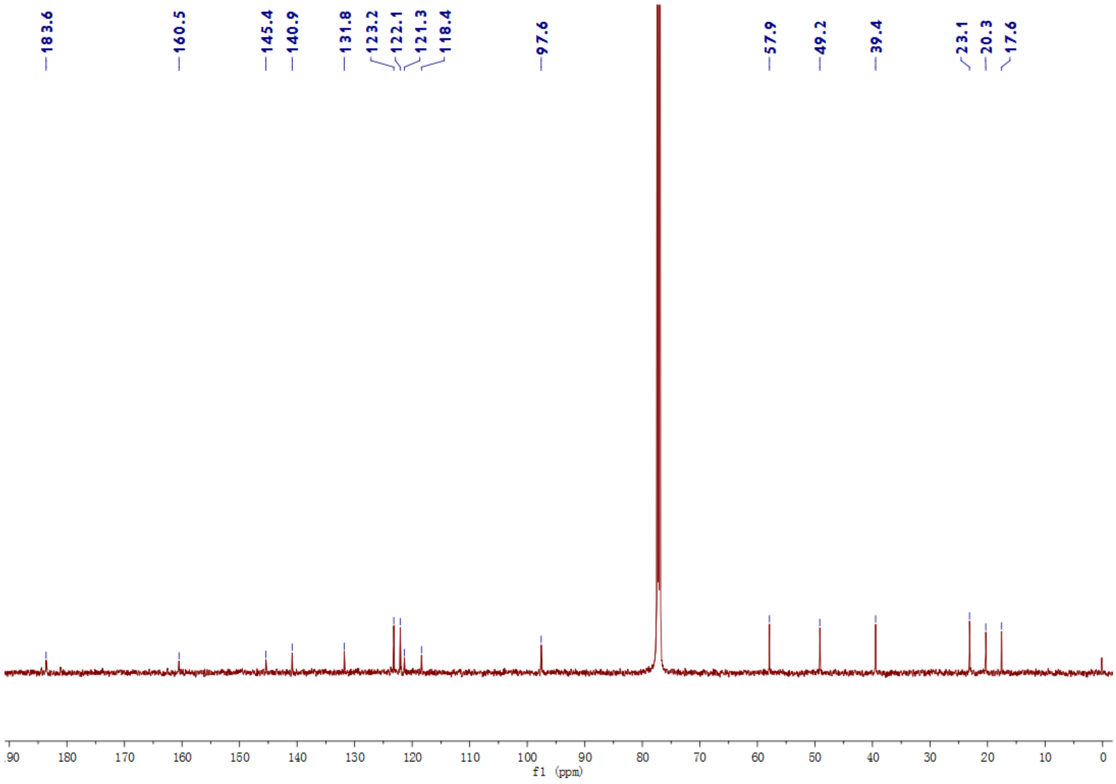

Figure S39. ^{1}H-^{1}H COSY spectrum of **5** in $CDCl_3$

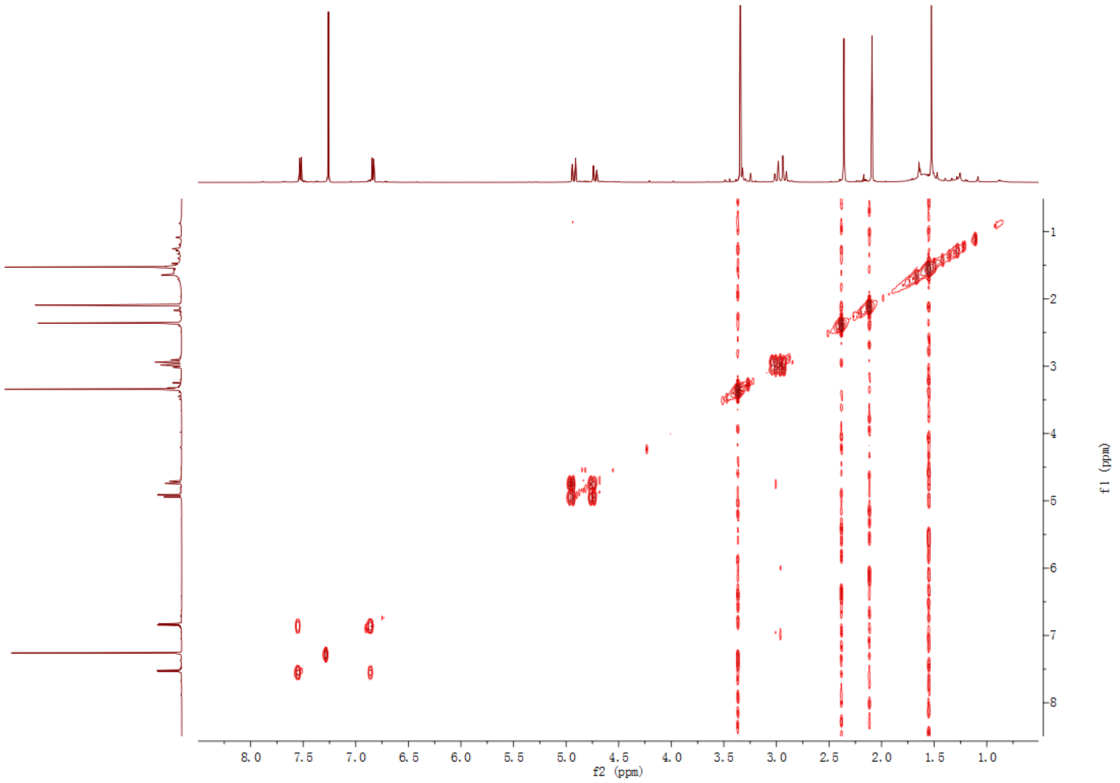

Figure S40. HSQC spectrum of **5** in $CDCl_3$

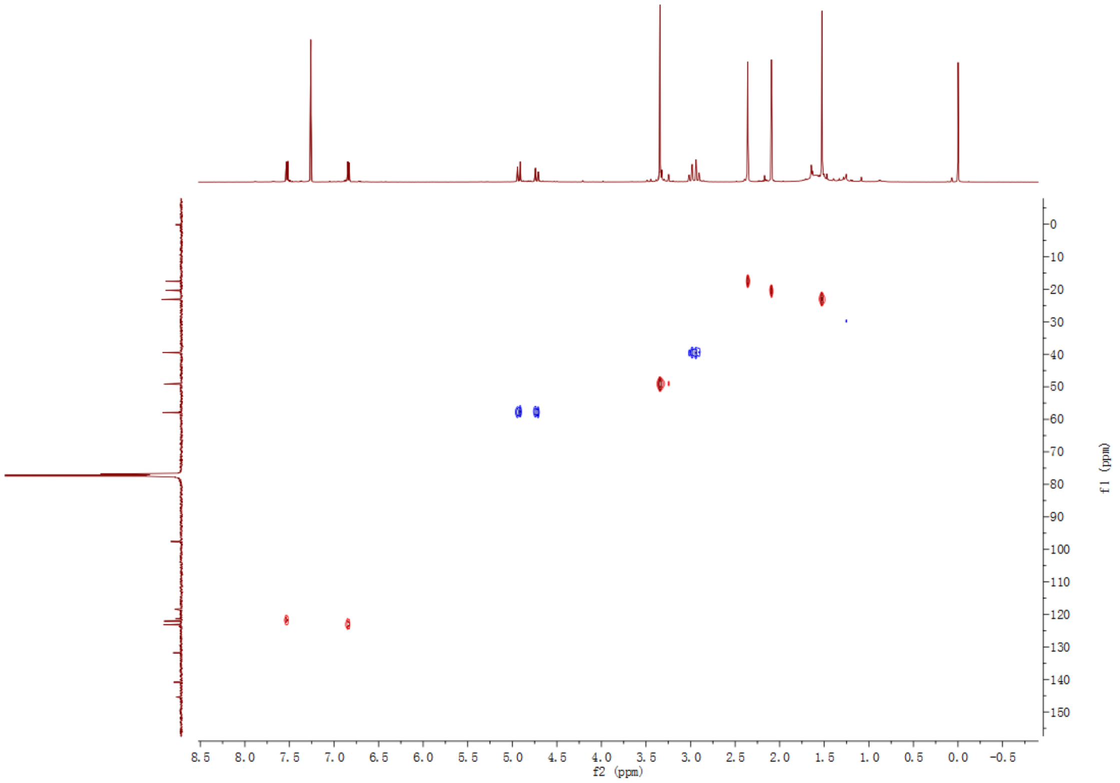

Figure S41. HMBC spectrum of **5** in $CDCl_3$

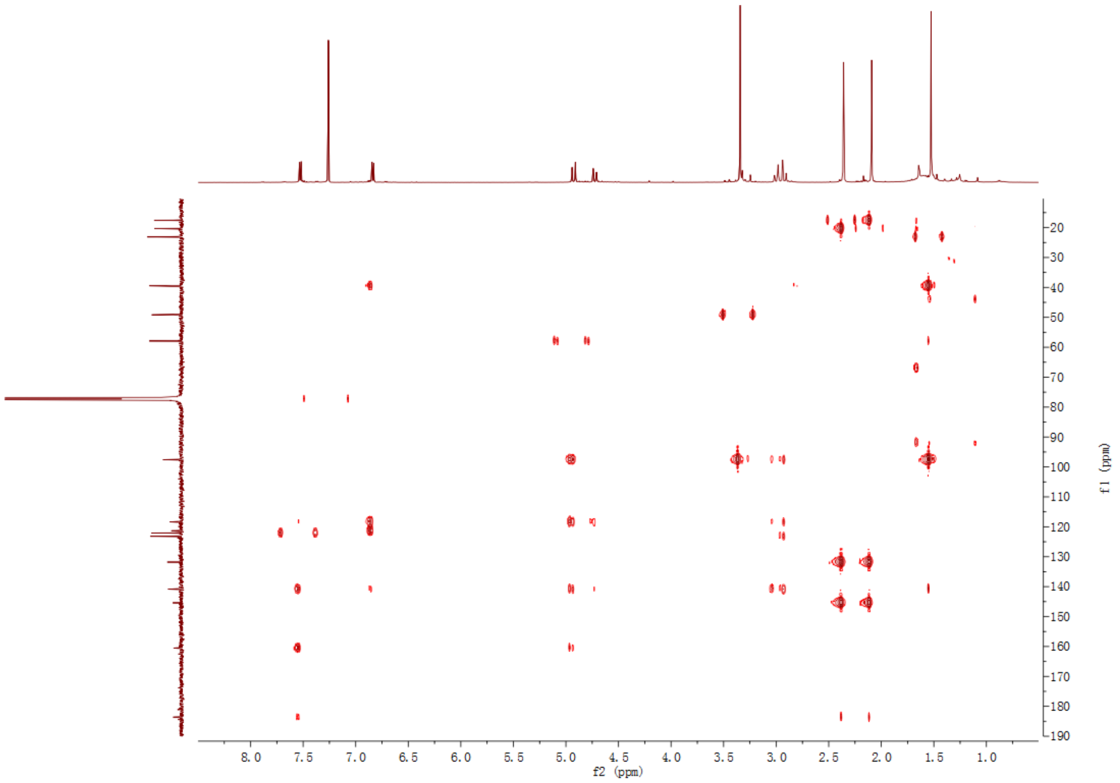

Figure S42. IR spectrum of **5**

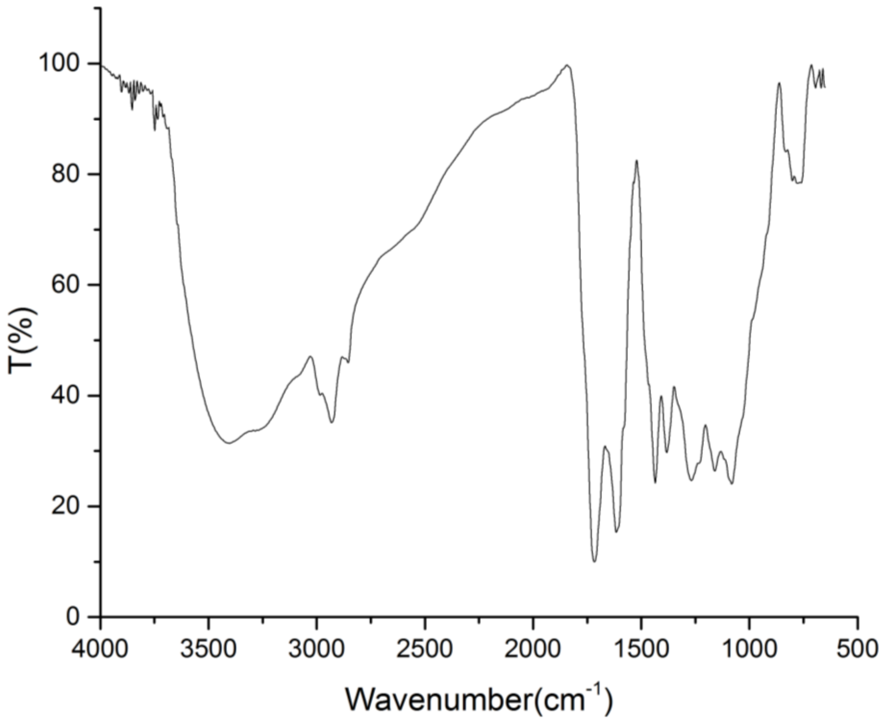

Figure S43. UV data of **5** in MeOH

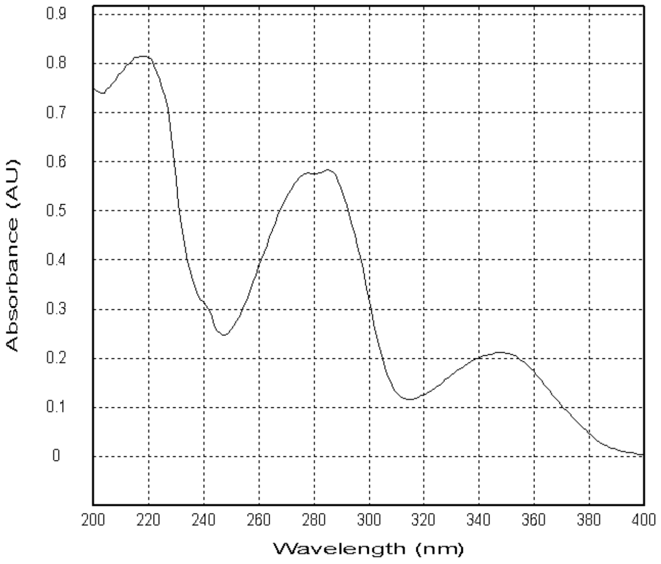

Figure S44. HRESIMS spectrum of **6**

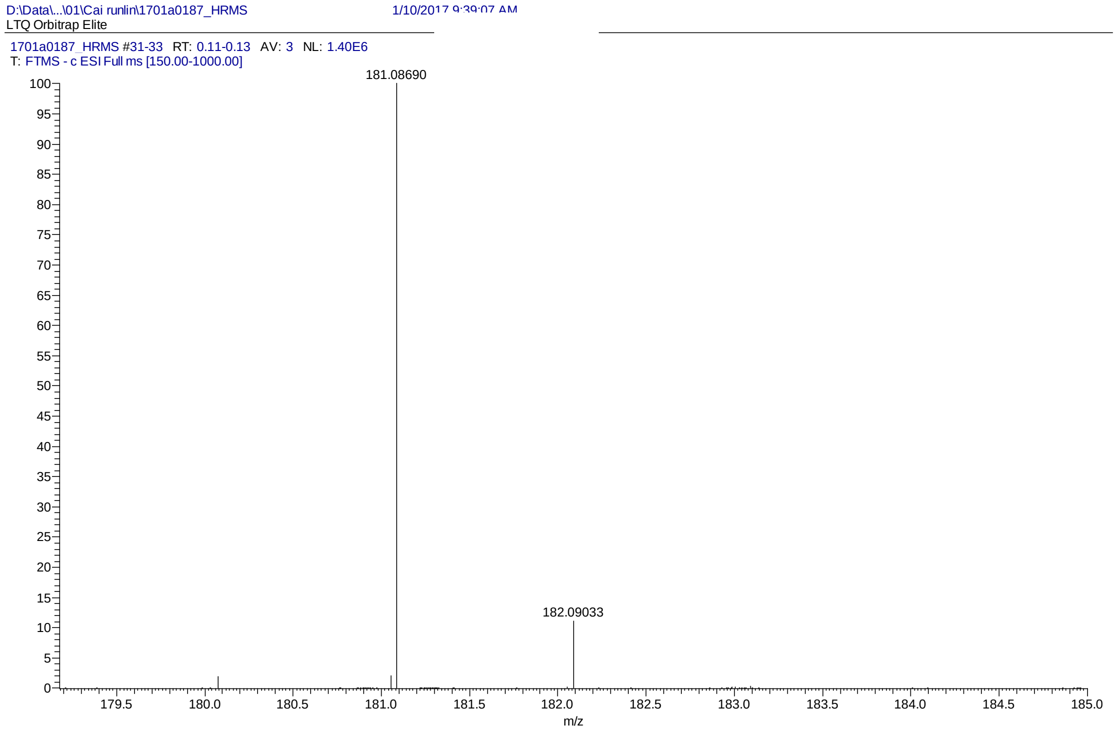

SPECTRUM - simulation :

m/z	Theo. Mass	Delta (ppm)	RDB equiv.	Composition
181.08690	181.08702	-0.65	4.5	$C_{10}H_{13}O_3$

Figure S45. ^{1}H NMR spectrum of **6** in acetone-d_6

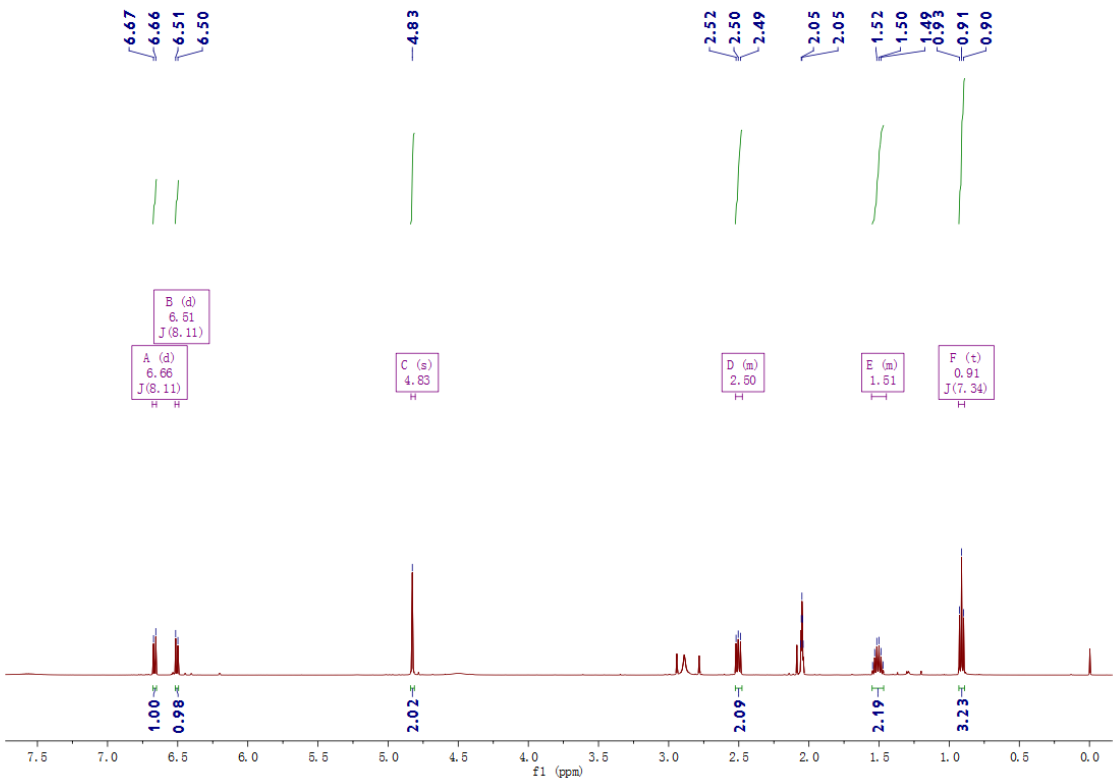

Figure S46. ^{13}C NMR spectrum of **6** in acetone-d_6

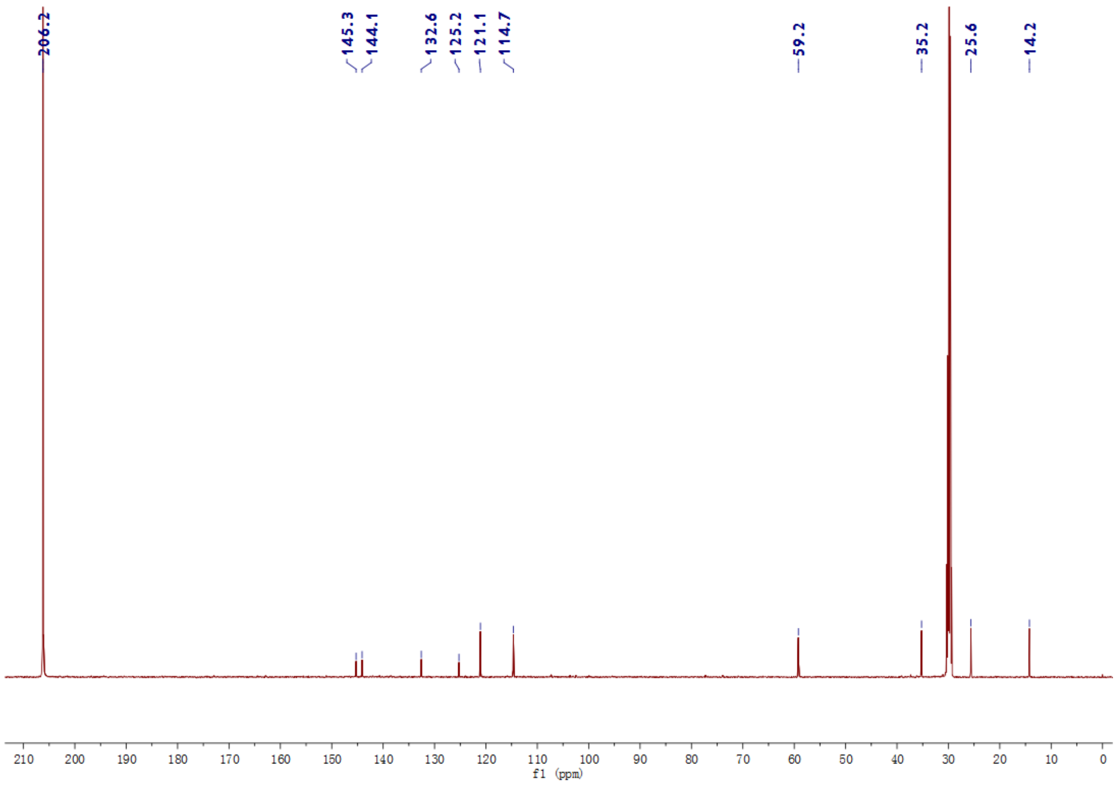

Figure S47. DEPT spectrum of **6** in acetone-d_6

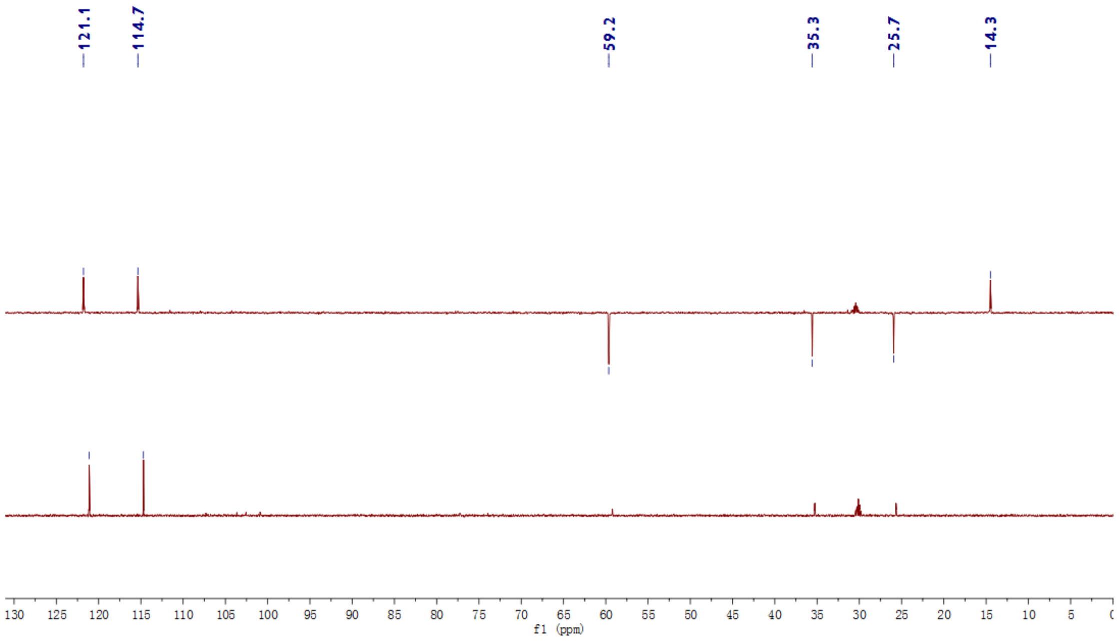

Figure S48. ^{1}H-^{1}H COSY spectrum of **6** in acetone-d_6

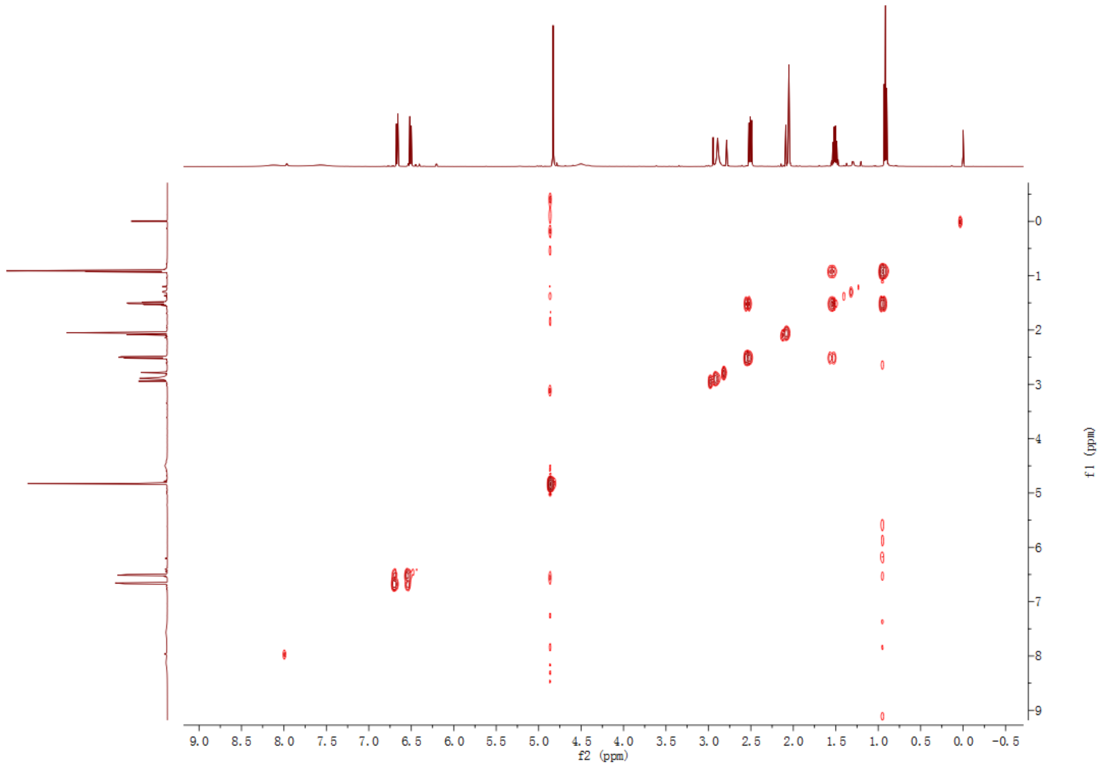

Figure S49. HSQC spectrum of **6** in acetone-d_6

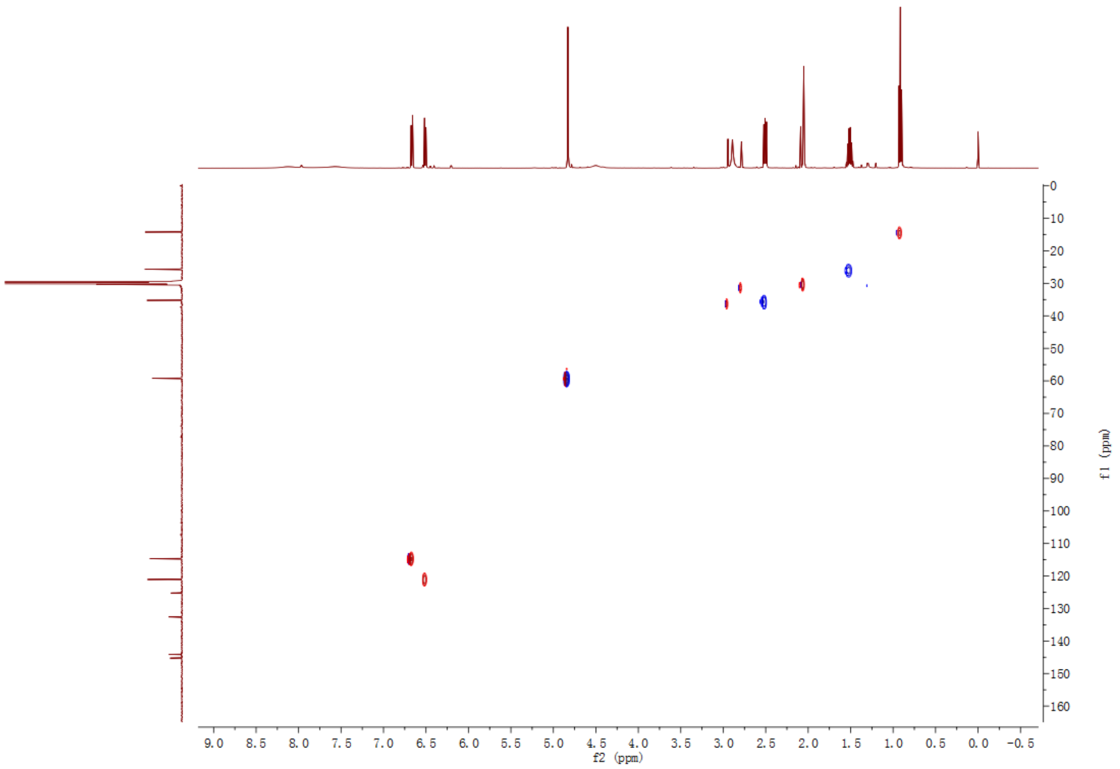

Figure S50. HMBC spectrum of **6** in acetone-d_6

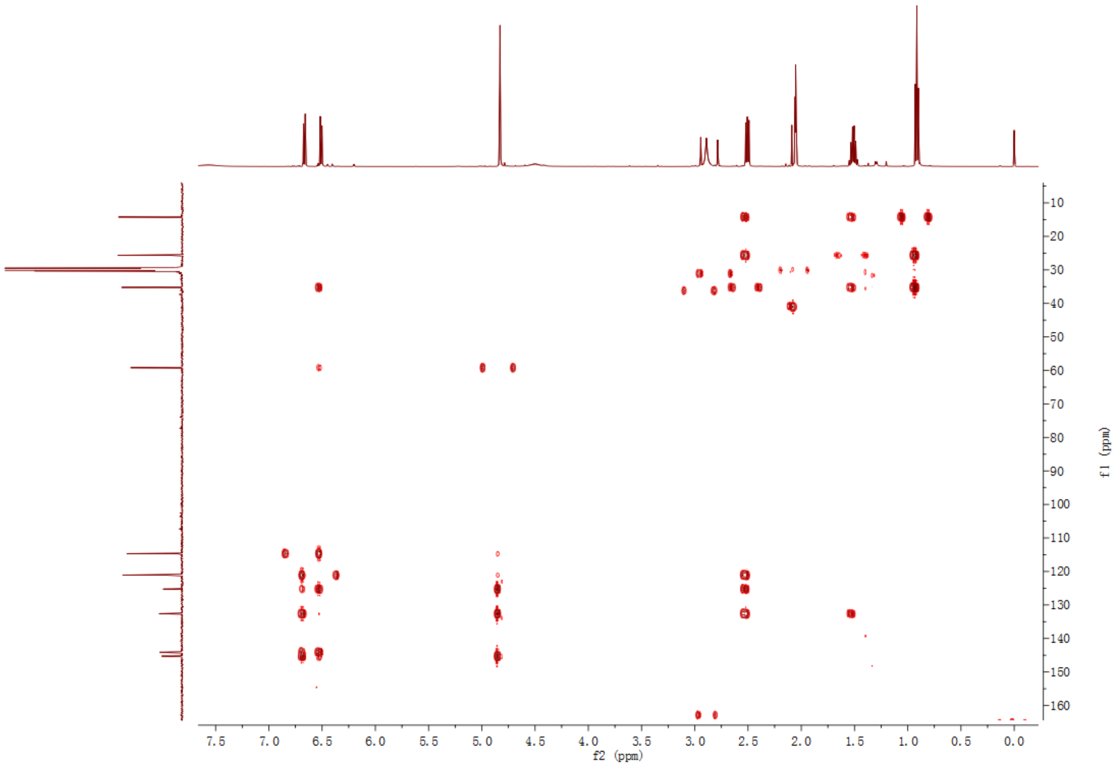

Figure S51. IR spectrum of **6**

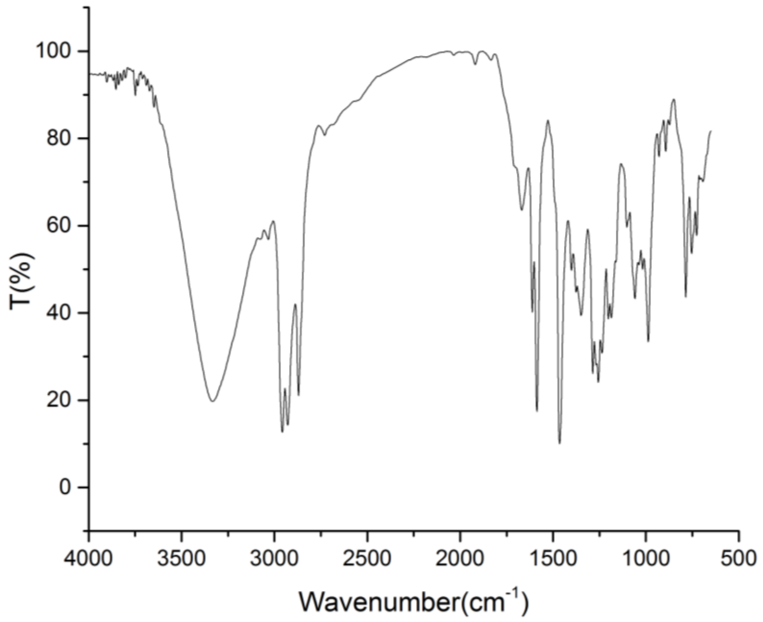

Figure S52. UV data of **6** in MeOH

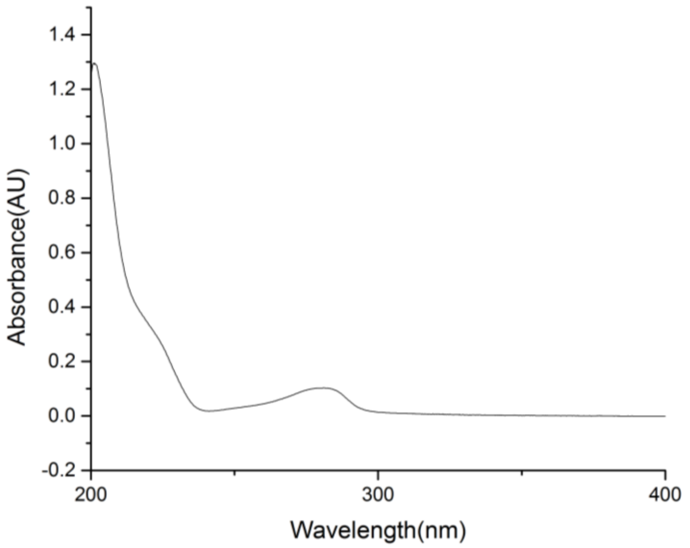

Figure S53. HRESIMS spectrum of **7**

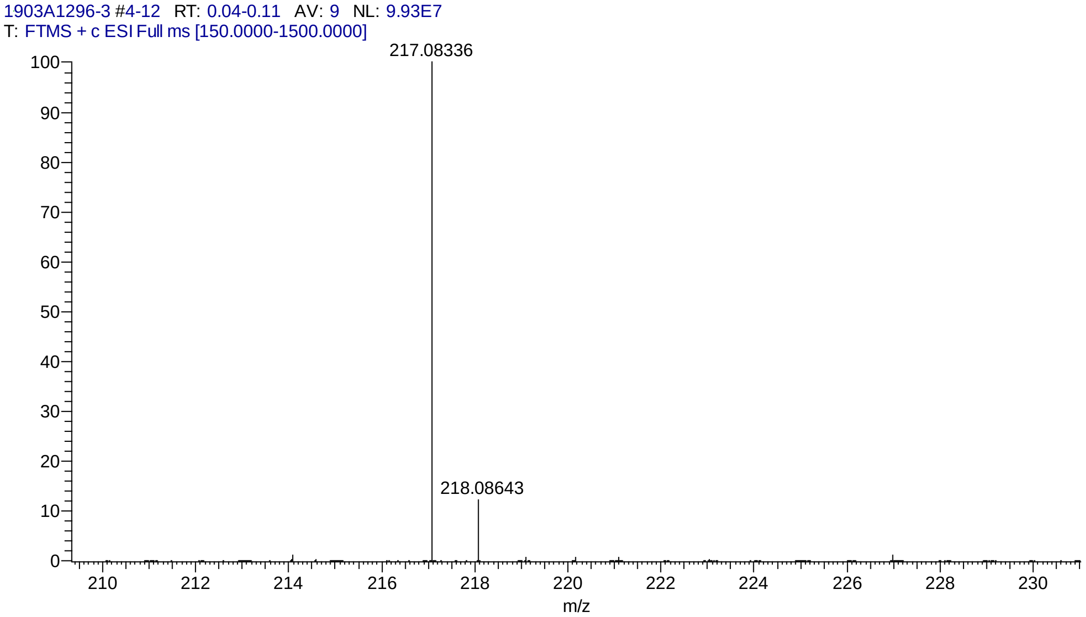

m/z	Theo. Mass	Delta (mmu)	RDB equiv.	Composition
217.08336	217.08352	-0.16	4.5	$C_{11}H_{14}O_3Na$

Figure S54. ^{1}H NMR spectrum of **7** in methanol-d_4

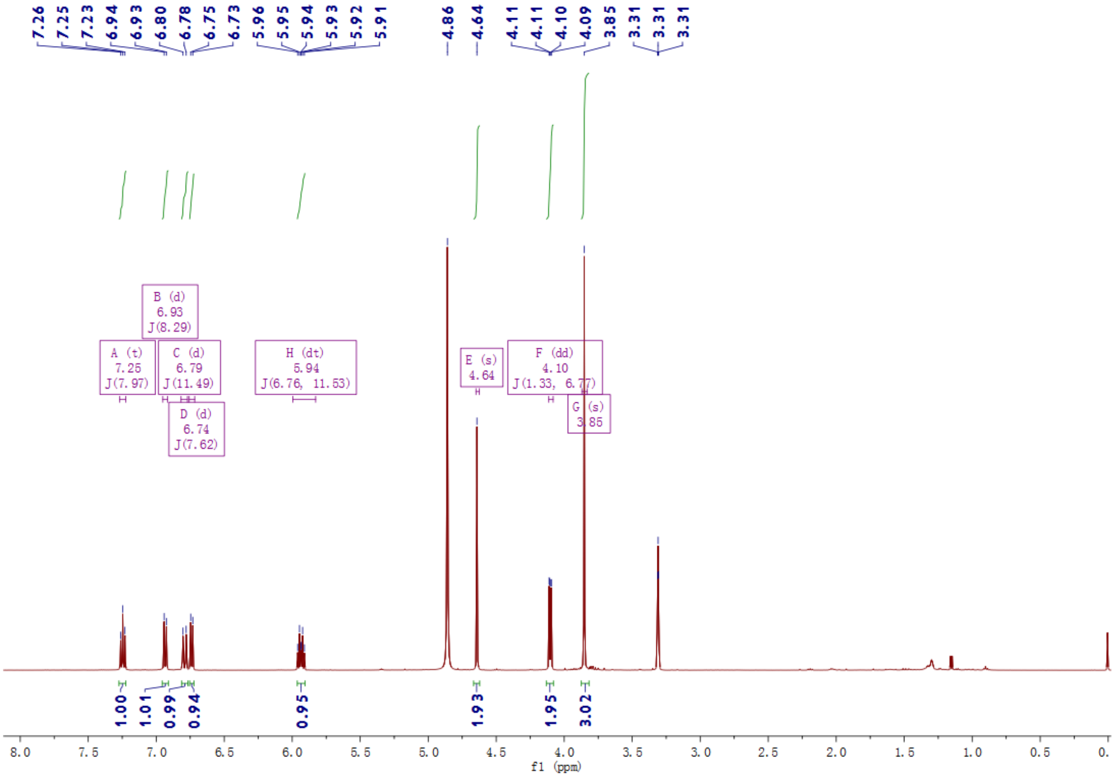

Figure S55. ^{13}C NMR spectrum of **7** in methanol-d_4

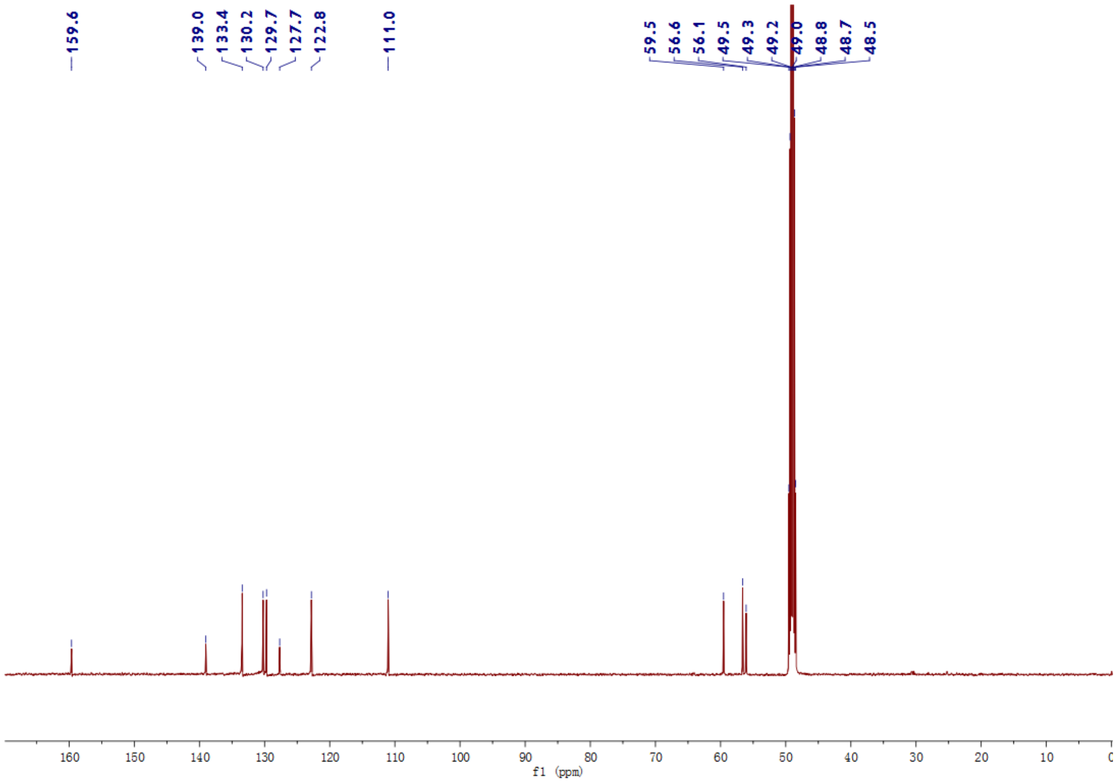

Figure S56. DEPT spectrum of **7** in methanol-d_4

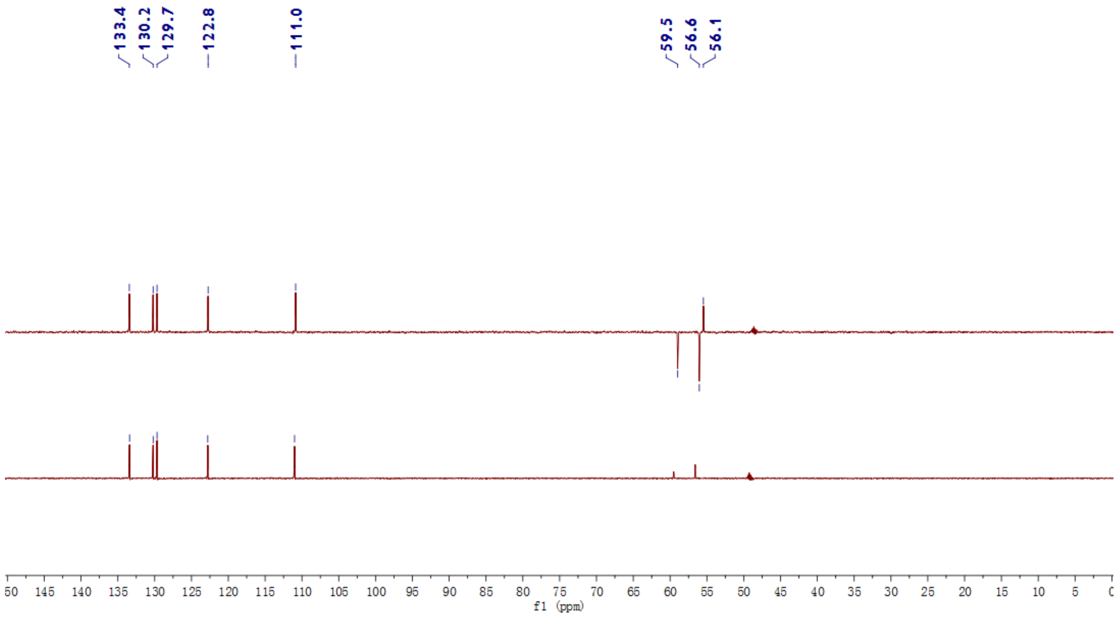

Figure S57. ^{1}H-^{1}H COSY spectrum of **7** in methanol-d_4

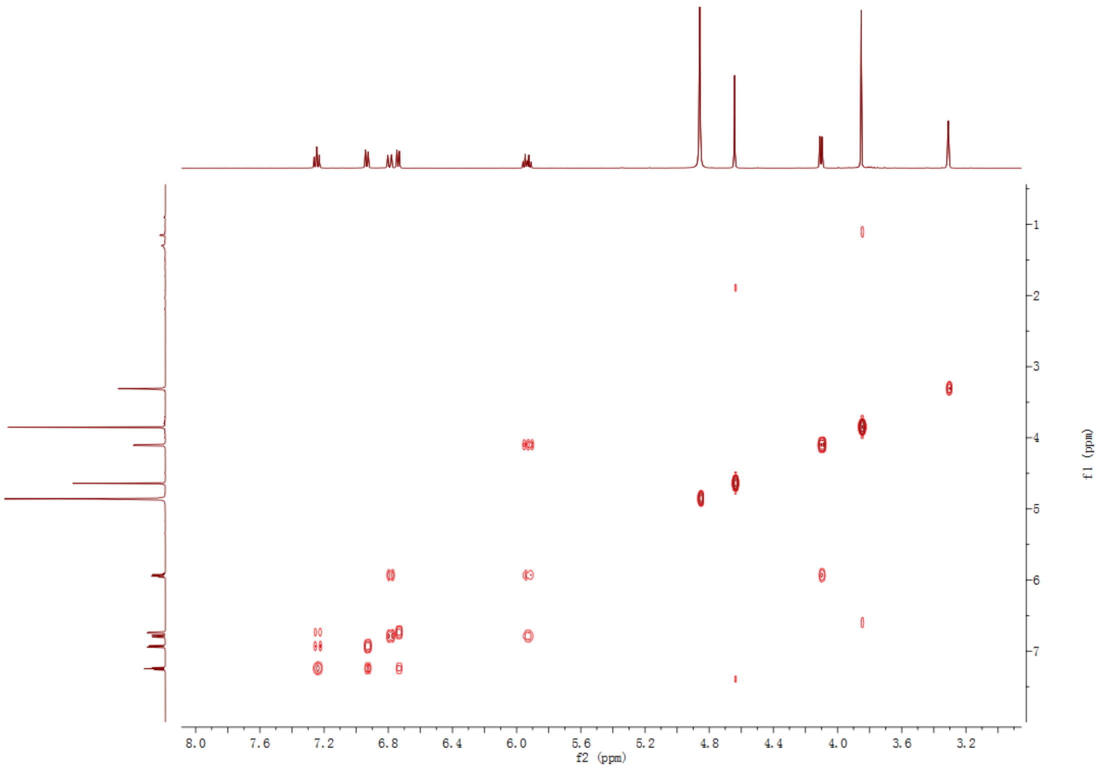

Figure S58. HSQC spectrum of **7** in methanol-d_4

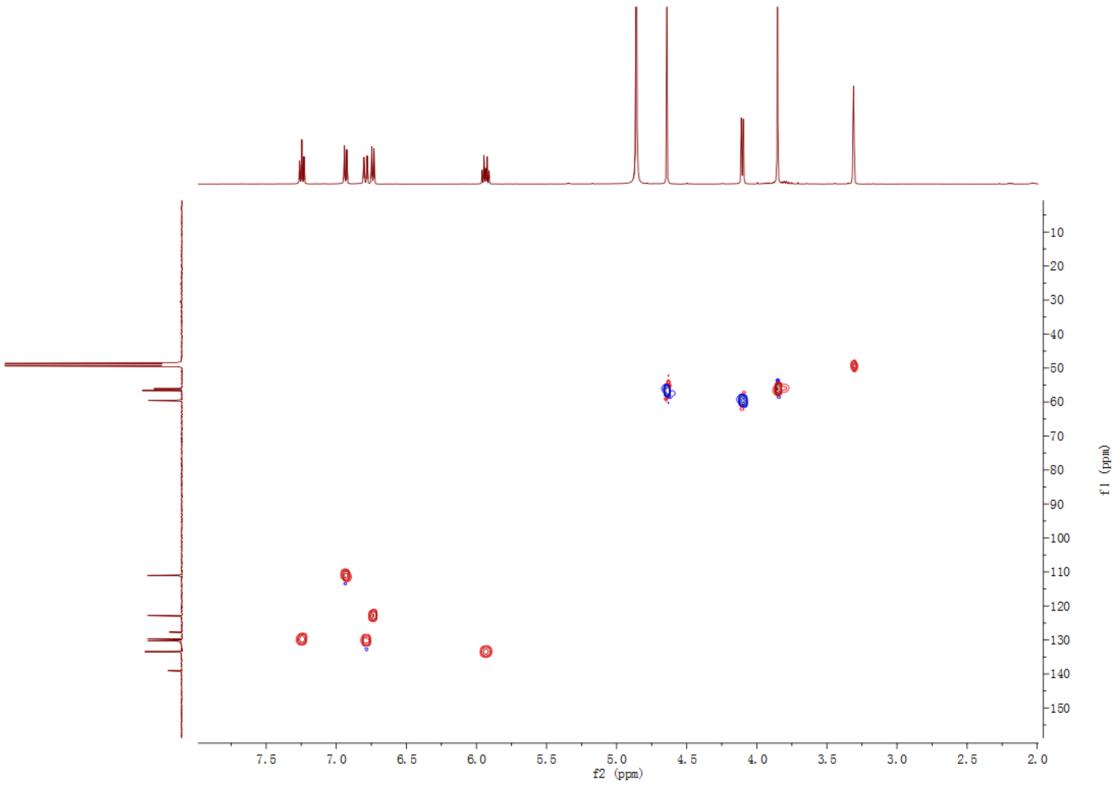

Figure S59. HMBC spectrum of **7** in methanol-d_4

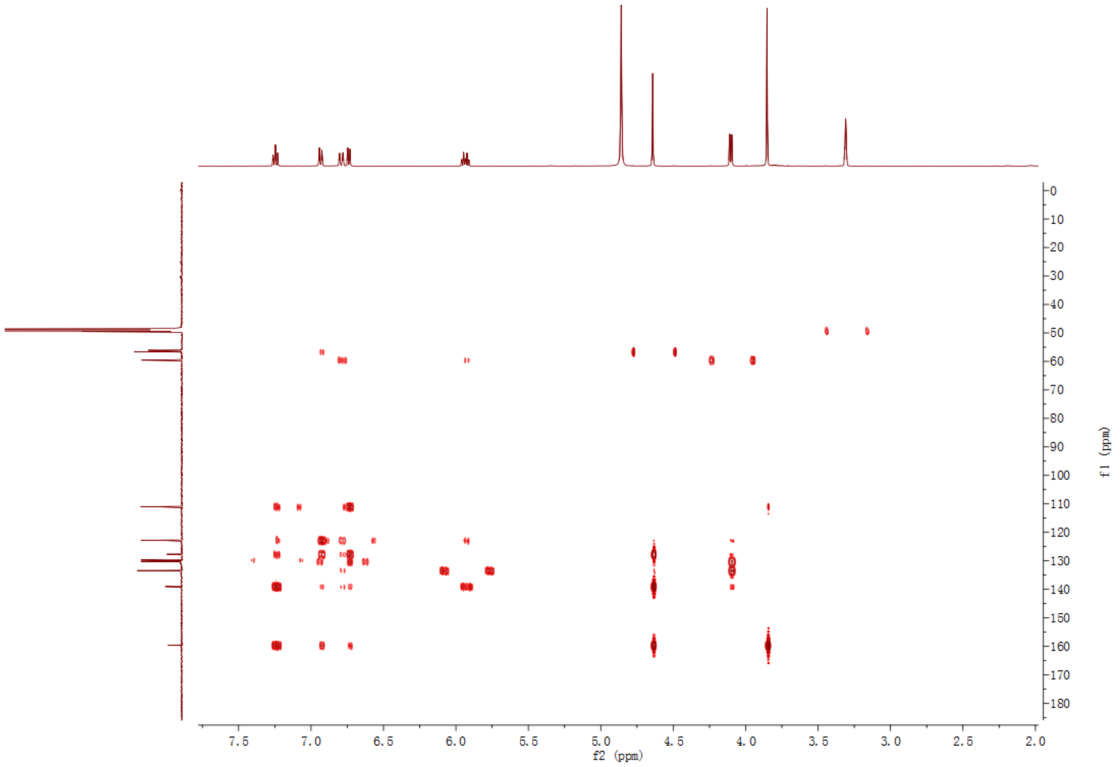

Figure S60. NOESY spectrum of 7 in methanol-d_4

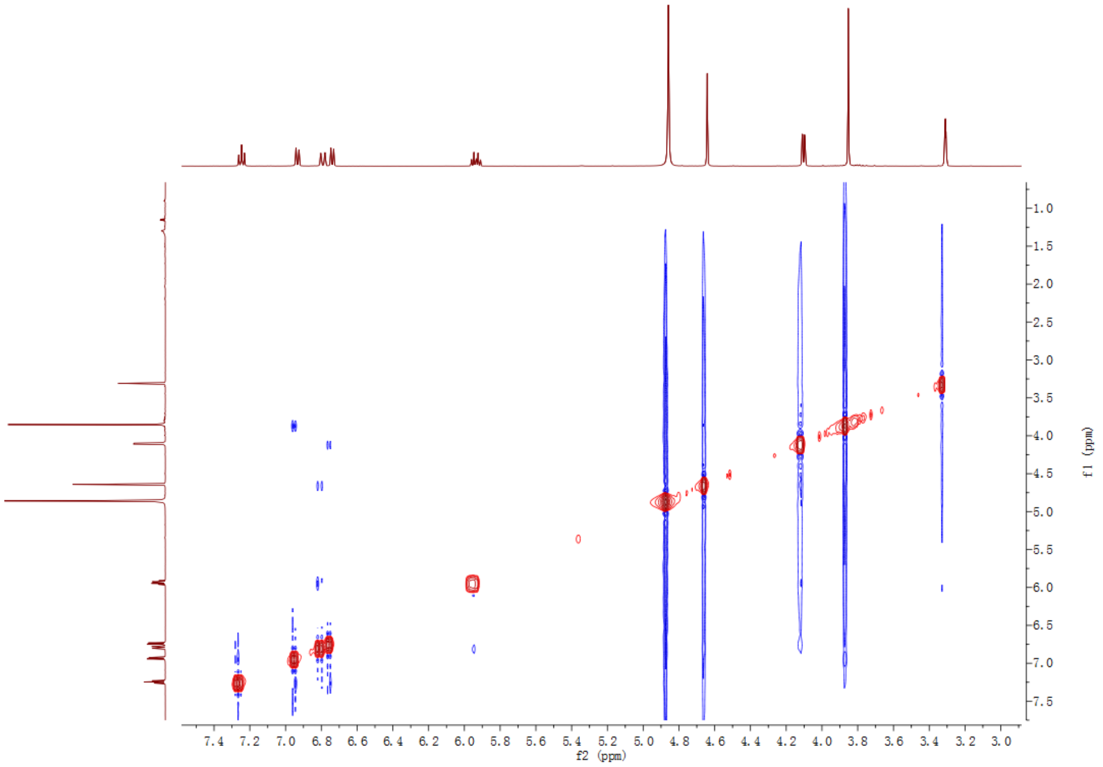

Figure S61. IR spectrum of **7**

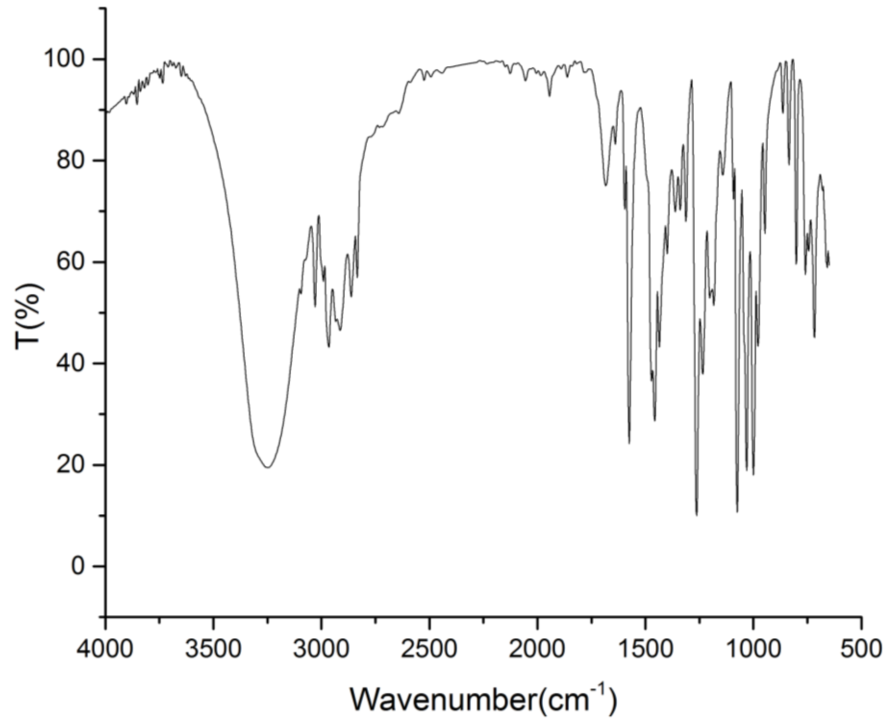

Figure S62. UV data of **7** in MeOH

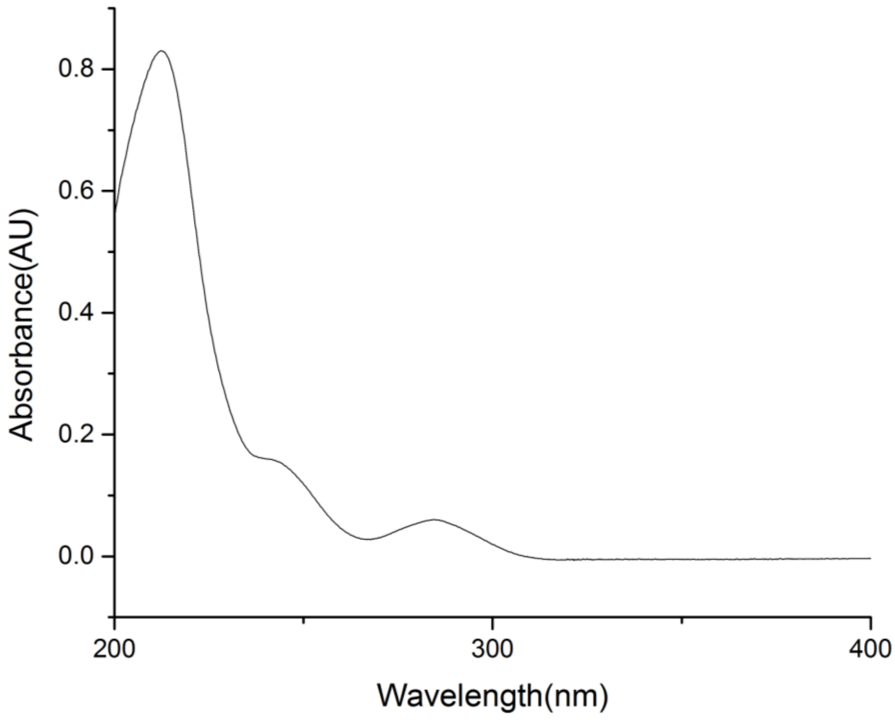

Preparation of MTPA esters of 1 by the modified Mosher's method

Compound **1** (4.1 mg) was reacted with (–)-α-methoxy-α-(trifluoromethyl) phenylacetyl chloride ((–)-MTPA-Cl, 20 μL) in anhydrous pyridine (400 μL) for 8 h at room temperature. Then the solvent from reaction mixture was removed under reduced pressure to furnish a residue, which was purified by preparative TLC (with CH_2Cl_2 eluant) to give (*S*)-MTPA ester (**1a**, 3.8 mg). Through a same procedure, (*R*)-MTPA ester (**1b**, 3.7 mg) was obtained from **1** (4.2 mg) using (+)-MTPA-Cl.

(*S*)-MTPA ester for 1a: ^{1}H NMR ($CDCl_3$, 400 MHz) δ_H 7.04 (1H, d, *J* = 7.7 Hz, H-4), 6.60 (1H, d, *J* = 7.7 Hz, H-5), 5.81 (1H, dd, *J* = 5.1, 8.7 Hz, H-6), 5.63 (1H, d, *J* = 12.0 Hz, H-9a), 5.33 (1H, d, *J* = 12.0 Hz, H-9b), 4.60 (1H, t, *J* = 9.1 Hz, H-2), 3.22 (1H, dd, *J* = 8.9, 16.6 Hz, H-3a), 3.11 (1H, dd, *J* = 9.4, 16.6 Hz, H-3b), 1.64 (2H, m, H-7), 1.33 (3H, s, H-13), 1.19 (3H, s, H-12), 0.75 (3H, t, *J* = 7.3 Hz, H-10); ESIMS *m*/*z* 716.05 $[M+NH_4^+]^+$.

(*R*)-MTPA ester for 1b: ^{1}H NMR ($CDCl_3$, 400 MHz) δ_H 7.14 (1H, d, *J* = 7.7 Hz, H-4), 6.85 (1H, d, *J* = 7.7 Hz, H-5), 6.00 (1H, dd, *J* = 4.9, 8.8 Hz, H-6), 5.66 (1H, d, *J* = 12.0 Hz, H-9a), 5.48 (1H, d, *J* = 12.0 Hz, H-9b), 4.62 (1H, t, *J* = 9.1 Hz, H-2), 3.22 (1H, dd, *J* = 8.3, 16.3 Hz, H-3a), 3.13 (1H, dd, *J* = 9.4, 16.3 Hz, H-3b), 1.55 (2H, m, H-7), 1.35 (3H, s, H-13), 1.19 (3H, s, H-12), 0.72 (3H, t, *J* = 7.3 Hz, H-10); ESIMS *m*/*z* 716.05 $[M+NH_4^+]^+$.

Figure S63. ESIMS spectrum of (*S*)-MTPA ester (**1a**)

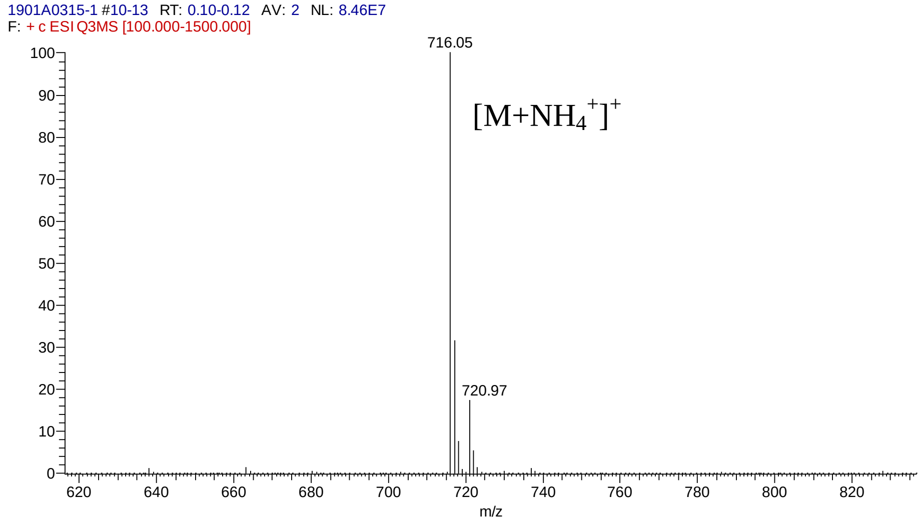

Figure S64. ESIMS spectrum of (*R*)-MTPA ester (**1b**)

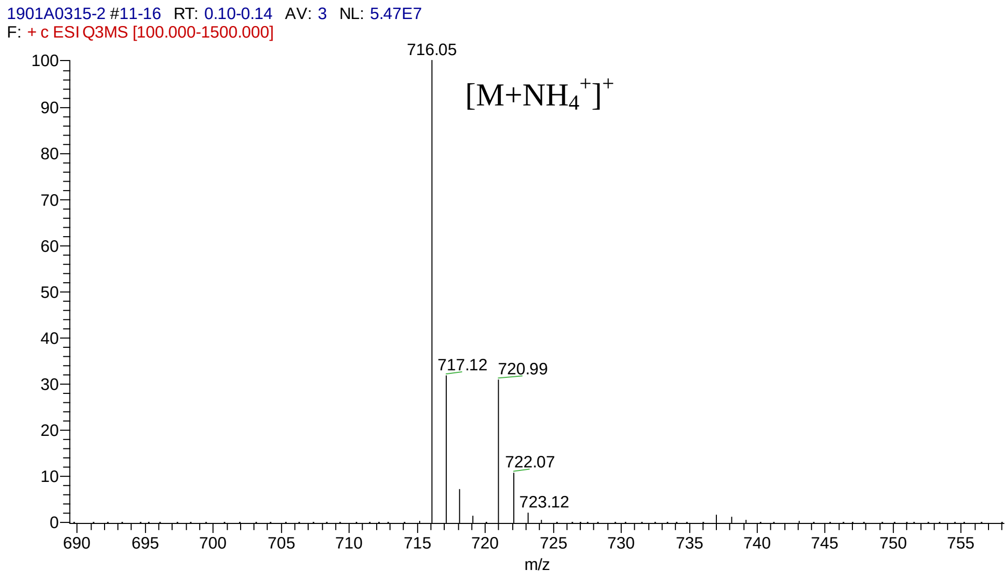

Figure S65. ^{1}H NMR spectrum of (*S*)-MTPA ester (**1a**) in $CDCl_3$

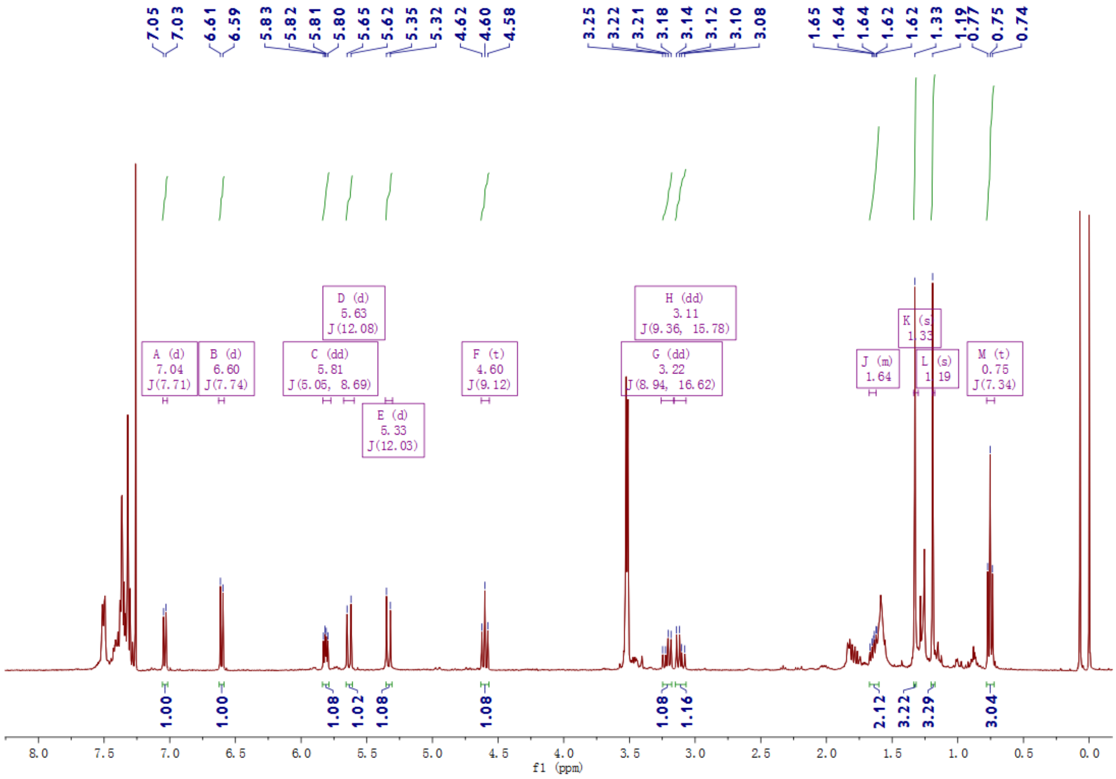

Figure S66. ^{1}H NMR spectrum of (*R*)-MTPA ester (**1b**) in $CDCl_3$

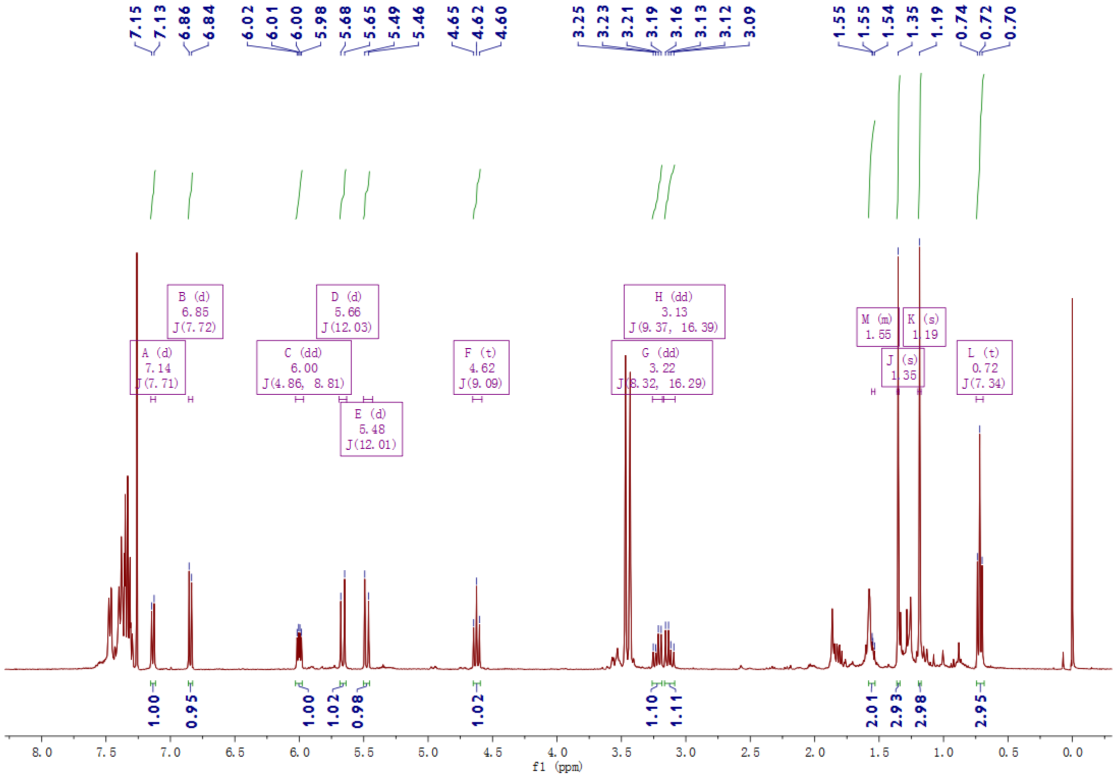

The ^{1}H and ^{13}C NMR data and HRMS data of compounds 8-14.

2-(Hydroxymethyl)-3-propylphenol (**8**): colorless oil; ^{1}H NMR (400 MHz, $CDCl_3$): δ_H 7.11(1H, t, J = 7.9, H-2), 6.73 (1H, d, J = 7.9, H-1), 6.71 (1H, d, J =7.9, H-3), 4.90 (2H, s, H-10), 2.55 (2H, t, J = 7.7, H-7), 1.54 (2H, m, H-8), 0.94 (3H, t, J = 7.3, H-9); ^{13}C NMR (101 MHz, $CDCl_3$): δ_C 156.6 (C-6), 141.1 (C-4), 128.9 (C-2), 123.0 (C-5), 121.8 (C-3), 114.5 (C-1), 60.0 (C-10), 35.4 (C-7), 24.9 (C-8), 14.1 (C-9); HRESIMS m/z 167.1076 $[M+H]^+$ (calcd for $C_{10}H_{15}O_2$, 167.1072).

Peniciphenol (**9**): colorless oil; ^{1}H NMR (400 MHz, CD_3OD) δ_H 7.08 (1H, t, J = 7.8 Hz, H-2), 6.75 (1H, d, J = 7.8 Hz, H-1), 6.74 (1H, d, J = 11.6 Hz, H-7), 6.61 (1H, d, J = 7.8 Hz, H-3), 5.90 (1H, dt, J = 11.6, 6.7 Hz, H-8), 4.68 (2H, s, H-10), 4.12 (2H, dd, J = 6.7, 1.4 Hz, H-9); ^{13}C NMR (101 MHz, CD_3OD) δ_C 156.3 (C-6), 136.3 (C-4), 131.9 (C-8), 130.0 (C-7), 128.5 (C-2), 123.6 (C-5), 120.9 (C-3), 115.3 (C-1), 59.1 (C-9), 58.6 (C-10); HRESIMS m/z 181.0852 $[M+H]^+$ (calcd for $C_{10}H_{13}O_3$, 181.0865).

Penicibenzoxepinol (**10**): colorless oil; ^{1}H NMR (400 MHz, $CDCl_3$) δ_H 7.05 (1H, t, J = 7.8 Hz, H-2), 6.82 (1H, d, J = 7.8 Hz, H-1), 6.59 (1H, d, J = 7.8 Hz, H-3), 6.47 (1H, dt, J = 12.4, 2.1 Hz, H-7), 5.86 (1H, dt, J = 12.4, 3.0 Hz, H-8), 4.85 (2H, s, H-10), 4.56 (2H, t, J = 2.6 Hz, H-9); ^{13}C NMR (101 MHz, $CDCl_3$) δ_C 153.4 (C-6), 138.1 (C-4), 133.2 (C-8), 128.7 (C-7), 127.7 (C-2), 126.3 (C-5), 122.3 (C-3), 113.8 (C-1), 72.8 (C-9), 65.0 (C-10); HRESIMS m/z 163.0749 $[M + H]^+$ (calcd for $C_{10}H_{11}O_2$, 163.0759).

(−)-Brassicadiol (**11**): colorless oil; ^{1}H NMR (400 MHz, $CDCl_3$) δ_H 6.98 (1H, d, J = 7.5 Hz, H-4), 6.65 (1H, d, J = 7.5 Hz, H-5), 4.70 (1H, d, J = 12.0 Hz, H-9), 4.64 (1H, d, J = 12.0 Hz, H-9), 4.58 (1H, t, J = 9.1 Hz, H-2), 3.09 (2H, m, H-3), 2.60, (2H, m, H-6), 1.56 (2H, m, H-7), 1.32 (3H, s, H-13), 1.16 (3H, s, H-12), 0.95 (3H, t, J = 7.3 Hz, H-10); ^{13}C NMR (101 MHz, $CDCl_3$) δ_C 158.8 (C-9b), 141.0 (C-5a), 124.7 (C-3a), 124.2 (C-4), 121.8 (C-5), 120.2 (C-9a), 89.8 (C-2), 71.8 (C-11), 57.1 (C-9), 34.7 (C-6), 30.6 (C-3), 26.4 (C-13), 25.3 (C-7), 24.1 (C-12), 14.2 (C-10); HRESIMS m/z 251.1627 $[M + H]^+$ (calcd for $C_{15}H_{23}O_3$, 251.1647).

(+)-Pseudodeflectusin (**12**): colorless oil; ^{1}H NMR (400 MHz, $CDCl_3$) δ_H 7.61 (1H, d, J = 7.9 Hz, H-4), 6.88 (1H, d, J = 7.9 Hz, H-5), 6.28 (1H, s, H-9), 4.46 (1H, m,

H-7), 2.79 (1H, dd, J = 17.3, 3.6 Hz, H-6), 2.71 (1H, J = 17.3, 10.7 Hz, H-6), 2.36 (3H, s, H-13), 2.12 (3H, s, H-12), 1.40 (3H, d, J = 6.2 Hz, H-10); ^{13}C NMR (101 MHz, $CDCl_3$) δ_C 183.2 (C-3), 162.0 (C-9b), 145.2 (C-2), 143.5 (C-5a), 132.3 (C-11), 123.8 (C-4), 122.7 (C-5), 122.2 (C-3a), 119.5 (C-9a), 87.8 (C-9), 62.7 (C-7), 35.9 (C-6), 21.1 (C-10), 20.3 (C-12), 17.8 (C-13); HRESIMS m/z 261.1136 $[M + H]^+$ (calcd for $C_{16}H_{21}O_4$, 261.1127).

(−)-Penicisochroman B (**13**): colorless oil; ^{1}H NMR (400 MHz, $CDCl_3$) δ_H 7.50 (1H, d, J = 7.9 Hz, H-4), 6.79 (1H, d, J = 7.9 Hz, H-5), 5.67 (1H, s, H-9), 4.41 (1H, d, J = 4.0 Hz, H-2), 4.25 (1H, m, H-7), 3.61 (3H, s, OCH_3-9), 2.74, (1H, dd, J = 17.3, 3.8 Hz, H-6), 2.67 (1H, J = 17.3, 10.5 Hz, H-6), 2.34 (1H, m, H-11), 1.39 (3H, d, J = 6.3 Hz, H-10), 1.18 (3H, d, J = 6.9 Hz, H-13), 0.87 (3H, d, J = 6.9 Hz, H-12); ^{13}C NMR (101 MHz, $CDCl_3$) δ_C 202.1 (C-3), 171.0 (C-9b), 145.5 (C-5a), 123.2 (C-4), 122.4 (C-5), 120.2 (C-3a), 120.0 (C-9a), 94.9 (C-9), 90.2 (C-2), 62.7 (C-7), 55.8, (OCH_3-9), 35.9 (C-6), 31.4 (C-11), 21.1 (C-10), 19.0 (C-12), 15.8 (C-13); HRESIMS m/z 277.1438 $[M + H]^+$ (calcd for $C_{16}H_{21}O_4$, 277.1440).

Ustusorane A (**14**): colorless oil; ^{1}H NMR (400 MHz, $CDCl_3$) δ_H 7.55 (1H, d, J = 7.8 Hz, H-4), 6.95 (1H, d, J = 7.8 Hz, H-5), 4.91 (1H, d, J = 12.0 Hz, H-9), 4.72 (1H, dd, J = 12.0, 2.2 Hz, H-9), 4.08 (1H, m, H-7), 2.95 (1H, dd, J = 13.7, 9.2 Hz, H-6), 2.90 (1H, dt, J = 13.7, 3.0 Hz, H-6), 2.35 (3H, s, H-13), 2.11 (3H, s, H-12), 1.37 (3H, d, J = 6.4 Hz, H-10); ^{13}C NMR (101 MHz, $CDCl_3$) δ_C 183.6 (C-3), 163.1 (C-9b), 147.8 (C-5a), 145.1 (C-2), 132.3 (C-11), 124.6 (C-5), 124.3 (C-9a), 123.6 (C-4), 121.8 (C-3a), 68.8 (C-7), 54.3 (C-8), 41.6, (C-6), 24.2 (C-10), 20.2 (C-12), 17.5 (C-13); HRESIMS m/z 263.1298 $[M + H]^+$ (calcd for $C_{15}H_{19}O_4$, 263.1283).

The rDNA sequencing data of *Aspergillus* sp. ZJ-68

ATACACGGGGGGGAGGTTAGGCCTGCCCGGGGGCAGACCTGCACTCG GTAATGATCCTTCCGTAGGGGAACCTGCGGAAGGATCATTACCGAGTGCAG GTCTGCCCCCGGGCAGGCCTAACCTCCCACCCGTGAATACCTGACCAACGT TGCTTCGGCGGTGCGCCCCCCCCGGGGGTAGCCGCCGGAGACCACACCGA ACCTCCTGTCTTTAGTGTTGTCTGAGCTTGATAGCAAACCTATTAAAACTTT CAACAATGGATCTCTTGGTTCCGGCATCGATGAAGAACGCAGCGAACTGCG ATAAGTAATGTGAATTGCAGAATTCAGTGAATCATCGAGTCTTTGAACGCA CATTGCGCCCCCTGGCATTCCGGGGGGCATGCCTGTCCGAGCGTCATTGCT GCCCTTCAAGCCCGGCTTGTGTGTTGGGTCGTCGTCCCCCCCGGGGGACG GGCCCGAAAGGCAGCGGCGGCACCGCGTCCGGTCCTCGAGCGTATGGGGC TTTGTCACCCGCTCGATTAGGGCCGGCCGGGCGCCAGCCGGCGTCTCCAAC CTTCTATTTTACCAGGTtGACCTCGGATCAGGTAGGGATACCCGCTGAACTT AAGCATATCAATAAGGCGGGAGGAATCATCGTTACCCATA

Figure S67. Simple picture and microscopic picture of *Aspergillus* sp. ZJ-68

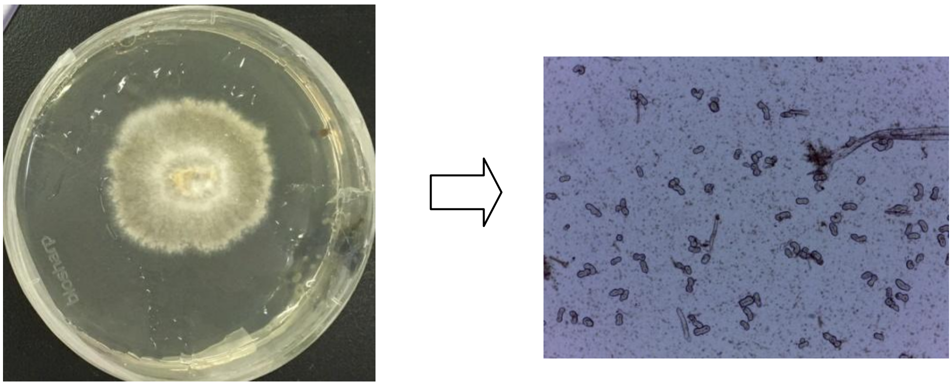

colonies of strain

hyphae and conidia of strain (10×10)

Figure S68. The chiral HPLC separation of (±)-penicisochroman A

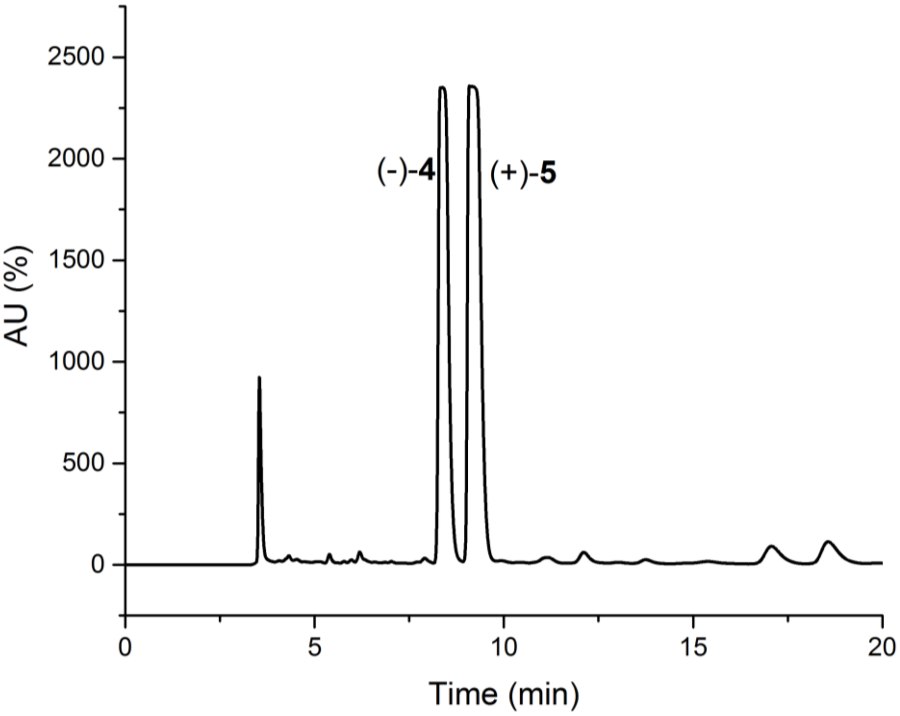

ECD calculation details (Methods and Results)

1. Methods

Conformational searches were carried out by means of the Spartan′14 software using Molecular Merck force field (MMFF). All density functional theory (DFT) and time-dependent (TD)-DFT calculations were performed with Gaussian 09 program. Conformers within a 10 kcal/mol energy window were generated and optimized by DFT calculations at the B3LYP/6-31+G (d, p) level. Conformers with a Bolzmann distribution over 3% were chose for ECD calculations by TD-DFT method at the B3LYP/6-311+G (d, p) level. The polarizable continuum model for MeOH was used. The calculated ECD curves were generated using the SpecDis 3.0 (University of Würzburg) and Origin Pro 8.5 (Origin Lab, Ltd.) from dipole-length rotational strengths by applying Gaussian band shapes with sigma = 0.30 eV.

2. Results

2.1. Gibbs free energies and Boltzmann-population of low-energy conformers of 2*R*,6*R*-**1**.

Conformers of 2*R*,6*R*-**1**	In MeOH	
	ΔG^a	P (%)[b]
**1a**	0.00	42.6
**1b**	0.16	32.5
**1c**	0.83	10.4
**1d**	1.01	7.7
**1e**	1.08	6.8

[a]Δ*G*, B3LYP/ 6-31+G (d, p), in kcal/mol. [b]Boltzmann-population.

2.2. Gibbs free energies and Boltzmann-population of low-energy conformers of 2*S*-**3**.

Conformers of 2*S* -**3**	In MeOH	
	ΔG^a	P (%)[b]
**3a**	0.00	66.7
**3b**	0.92	14.1
**3c**	1.22	8.5
**3d**	1.23	8.4

[a]Δ*G*, B3LYP/ 6-31+G (d, p), in kcal/mol. [b]Boltzmann-population.

2.3. Gibbs free energies and Boltzmann-population of low-energy conformers of 7*R*-**4**.

Conformers of 7*R*-**4**	In MeOH	
	ΔG^a	P (%)[b]
**4a**	0.00	96.0
**4b**	2.25	2.1
**4c**	2.34	1.8

[a]Δ*G*, B3LYP/ 6-31+G (d, p), in kcal/mol. [b]Boltzmann-population.

2.4 Cartesian coordinates for the low-energy optimized conformers of 2*R*,6*R*-**1** at B3LYP/6-31+G (d, p) level of theory in MeOH.

Conformer **1a**		Standard Orientation (Ångstroms)			
Center Number	Atom	Type	X	Y	Z
1	6	0	-0.990000	-1.120000	0.109000
2	6	0	-2.264000	-0.632000	0.302000
3	6	0	-2.481000	0.729000	0.079000
4	6	0	-1.434000	1.606000	-0.322000
5	6	0	-0.127000	1.083000	-0.503000
6	6	0	0.043000	-0.283000	-0.283000
7	8	0	1.243000	-0.947000	-0.398000
8	6	0	0.871000	-2.358000	-0.418000
9	6	0	-0.470000	-2.490000	0.322000
10	6	0	2.021000	-3.194000	0.191000
11	6	0	1.682000	-4.686000	0.207000
12	6	0	3.331000	-2.969000	-0.572000
13	6	0	-1.804000	3.070000	-0.506000
14	6	0	1.054000	1.904000	-0.950000
15	8	0	2.245000	-2.791000	1.549000
16	8	0	0.949000	2.235000	-2.328000
17	6	0	-1.393000	3.977000	0.662000
18	6	0	-2.107000	3.654000	1.966000
19	8	0	-1.229000	3.560000	-1.719000
20	1	0	-3.074000	-1.279000	0.622000
21	1	0	-3.487000	1.117000	0.228000
22	1	0	0.739000	-2.633000	-1.475000
23	1	0	-1.100000	-3.266000	-0.122000
24	1	0	-0.349000	-2.675000	1.394000
25	1	0	0.811000	-4.892000	0.838000
26	1	0	2.508000	-5.268000	0.633000

27	1	0	1.479000	-5.063000	-0.801000
28	1	0	3.649000	-1.921000	-0.521000
29	1	0	3.236000	-3.252000	-1.626000
30	1	0	4.145000	-3.554000	-0.129000
31	1	0	-2.889000	3.147000	-0.647000
32	1	0	1.999000	1.370000	-0.809000
33	1	0	1.140000	2.825000	-0.368000
34	1	0	2.342000	-1.819000	1.532000
35	1	0	0.069000	2.661000	-2.416000
36	1	0	-1.609000	5.019000	0.393000
37	1	0	-0.311000	3.941000	0.828000
38	1	0	-1.842000	4.390000	2.732000
39	1	0	-1.825000	2.667000	2.345000
40	1	0	-3.194000	3.679000	1.836000
41	1	0	-1.578000	4.459000	-1.853000

Conformer **1b**		Standard Orientation (Ångstroms)			
Center Number	Atom	Type	X	Y	Z
1	6	0	-0.669000	-1.079000	0.567000
2	6	0	-1.722000	-1.419000	-0.254000
3	6	0	-1.946000	-0.642000	-1.394000
4	6	0	-1.126000	0.468000	-1.719000
5	6	0	-0.082000	0.833000	-0.826000
6	6	0	0.126000	0.019000	0.284000
7	8	0	1.104000	0.224000	1.229000
8	6	0	1.124000	-1.013000	2.004000
9	6	0	-0.254000	-1.680000	1.854000
10	6	0	1.520000	-0.690000	3.465000
11	6	0	1.565000	-1.955000	4.325000
12	6	0	2.874000	0.025000	3.524000
13	6	0	-1.402000	1.296000	-2.968000
14	6	0	0.789000	2.042000	-1.035000
15	8	0	0.550000	0.190000	4.048000
16	8	0	-0.030000	3.205000	-1.140000
17	6	0	-2.093000	0.607000	-4.155000
18	6	0	-1.256000	-0.498000	-4.782000
19	8	0	-2.212000	2.408000	-2.584000
20	1	0	-2.374000	-2.254000	-0.019000
21	1	0	-2.791000	-0.907000	-2.025000
22	1	0	1.895000	-1.653000	1.550000

23	1	0	-0.171000	-2.770000	1.815000
24	1	0	-0.956000	-1.388000	2.641000
25	1	0	0.579000	-2.429000	4.391000
26	1	0	1.852000	-1.714000	5.355000
27	1	0	2.277000	-2.685000	3.929000
28	1	0	2.843000	0.985000	2.997000
29	1	0	3.671000	-0.587000	3.088000
30	1	0	3.145000	0.258000	4.561000
31	1	0	-0.458000	1.698000	-3.354000
32	1	0	1.382000	1.931000	-1.948000
33	1	0	1.480000	2.199000	-0.201000
34	1	0	0.417000	0.920000	3.412000
35	1	0	0.577000	3.965000	-1.129000
36	1	0	-2.298000	1.365000	-4.923000
37	1	0	-3.081000	0.224000	-3.873000
38	1	0	-1.769000	-0.904000	-5.659000
39	1	0	-1.082000	-1.322000	-4.086000
40	1	0	-0.284000	-0.114000	-5.109000
41	1	0	-1.716000	2.843000	-1.857000

Conformer **1c**		Standard Orientation (Ångstroms)			
Center Number	Atom	Type	X	Y	Z
1	6	0	-0.806000	-0.731000	0.776000
2	6	0	-1.934000	0.039000	0.964000
3	6	0	-2.015000	1.264000	0.297000
4	6	0	-0.980000	1.724000	-0.556000
5	6	0	0.198000	0.938000	-0.689000
6	6	0	0.230000	-0.288000	-0.030000
7	8	0	1.284000	-1.169000	-0.078000
8	6	0	0.763000	-2.390000	0.531000
9	6	0	-0.445000	-2.022000	1.403000
10	6	0	1.914000	-3.122000	1.262000
11	6	0	2.627000	-2.250000	2.300000
12	6	0	1.430000	-4.424000	1.903000
13	6	0	-1.099000	3.068000	-1.264000
14	6	0	1.386000	1.376000	-1.502000
15	8	0	2.900000	-3.486000	0.279000
16	8	0	1.837000	2.647000	-1.039000
17	6	0	-2.506000	3.599000	-1.577000
18	6	0	-3.269000	2.737000	-2.573000
19	8	0	-0.439000	4.045000	-0.458000

20	1	0	-2.735000	-0.284000	1.621000
21	1	0	-2.900000	1.872000	0.466000
22	1	0	0.430000	-3.013000	-0.313000
23	1	0	-1.236000	-2.773000	1.328000
24	1	0	-0.186000	-1.863000	2.453000
25	1	0	3.034000	-1.340000	1.845000
26	1	0	3.485000	-2.783000	2.727000
27	1	0	1.966000	-1.961000	3.123000
28	1	0	0.957000	-5.072000	1.156000
29	1	0	0.714000	-4.244000	2.710000
30	1	0	2.273000	-4.993000	2.313000
31	1	0	-0.568000	3.022000	-2.222000
32	1	0	1.117000	1.450000	-2.561000
33	1	0	2.223000	0.677000	-1.415000
34	1	0	3.112000	-2.670000	-0.212000
35	1	0	2.682000	2.816000	-1.492000
36	1	0	-2.409000	4.609000	-1.997000
37	1	0	-3.094000	3.734000	-0.662000
38	1	0	-4.233000	3.199000	-2.806000
39	1	0	-3.465000	1.736000	-2.179000
40	1	0	-2.710000	2.635000	-3.508000
41	1	0	0.467000	3.691000	-0.324000

Conformer **1d**		Standard Orientation (Ångstroms)			
Center Number	Atom	Type	X	Y	Z
1	6	0	-0.920000	-0.779000	1.113000
2	6	0	-2.208000	-0.292000	1.082000
3	6	0	-2.554000	0.573000	0.042000
4	6	0	-1.621000	0.960000	-0.959000
5	6	0	-0.295000	0.462000	-0.896000
6	6	0	0.006000	-0.410000	0.149000
7	8	0	1.241000	-0.977000	0.359000
8	6	0	0.987000	-2.042000	1.325000
9	6	0	-0.274000	-1.655000	2.116000
10	6	0	2.248000	-2.249000	2.197000
11	6	0	2.037000	-3.354000	3.234000
12	6	0	3.469000	-2.579000	1.330000
13	6	0	-2.120000	1.945000	-2.013000
14	6	0	0.764000	0.789000	-1.908000
15	8	0	2.548000	-1.042000	2.911000
16	8	0	0.458000	0.093000	-3.114000

17	6	0	-1.778000	3.393000	-1.633000
18	6	0	-2.402000	4.405000	-2.585000
19	8	0	-1.656000	1.671000	-3.330000
20	1	0	-2.934000	-0.563000	1.842000
21	1	0	-3.571000	0.963000	0.014000
22	1	0	0.792000	-2.954000	0.742000
23	1	0	-0.872000	-2.534000	2.373000
24	1	0	-0.050000	-1.078000	3.019000
25	1	0	1.240000	-3.093000	3.939000
26	1	0	2.939000	-3.496000	3.840000
27	1	0	1.785000	-4.308000	2.760000
28	1	0	3.702000	-1.761000	0.640000
29	1	0	3.312000	-3.493000	0.748000
30	1	0	4.362000	-2.714000	1.952000
31	1	0	-3.212000	1.851000	-2.075000
32	1	0	1.762000	0.485000	-1.577000
33	1	0	0.803000	1.863000	-2.108000
34	1	0	2.549000	-0.323000	2.249000
35	1	0	1.185000	0.290000	-3.730000
36	1	0	-0.693000	3.544000	-1.643000
37	1	0	-2.123000	3.607000	-0.614000
38	1	0	-2.163000	5.423000	-2.261000
39	1	0	-3.492000	4.304000	-2.605000
40	1	0	-2.025000	4.280000	-3.604000
41	1	0	-1.226000	0.791000	-3.324000

Conformer **1e**		Standard Orientation (Ångstroms)			
Center Number	Atom	Type	X	Y	Z
1	6	0	-1.182000	-0.608000	0.918000
2	6	0	-2.426000	-0.064000	0.701000
3	6	0	-2.606000	0.712000	-0.446000
4	6	0	-1.551000	0.961000	-1.370000
5	6	0	-0.279000	0.376000	-1.135000
6	6	0	-0.144000	-0.389000	0.025000
7	8	0	1.030000	-0.987000	0.429000
8	6	0	0.615000	-1.929000	1.465000
9	6	0	-0.696000	-1.402000	2.069000
10	6	0	1.765000	-2.099000	2.486000
11	6	0	1.385000	-3.076000	3.601000
12	6	0	3.049000	-2.575000	1.797000
13	6	0	-1.900000	1.856000	-2.555000

14	6	0	0.929000	0.553000	-2.013000
15	8	0	2.057000	-0.837000	3.102000
16	8	0	0.604000	0.321000	-3.380000
17	6	0	-1.085000	3.149000	-2.695000
18	6	0	-1.160000	4.049000	-1.471000
19	8	0	-1.835000	1.122000	-3.775000
20	1	0	-3.243000	-0.221000	1.397000
21	1	0	-3.592000	1.142000	-0.621000
22	1	0	0.431000	-2.889000	0.960000
23	1	0	-1.368000	-2.219000	2.346000
24	1	0	-0.535000	-0.744000	2.929000
25	1	0	0.534000	-2.705000	4.184000
26	1	0	2.210000	-3.194000	4.313000
27	1	0	1.130000	-4.062000	3.201000
28	1	0	3.399000	-1.847000	1.057000
29	1	0	2.902000	-3.537000	1.295000
30	1	0	3.863000	-2.684000	2.523000
31	1	0	-2.950000	2.161000	-2.469000
32	1	0	1.722000	-0.156000	-1.755000
33	1	0	1.337000	1.562000	-1.897000
34	1	0	2.165000	-0.193000	2.377000
35	1	0	1.449000	0.328000	-3.863000
36	1	0	-1.465000	3.703000	-3.563000
37	1	0	-0.039000	2.942000	-2.937000
38	1	0	-0.635000	4.989000	-1.663000
39	1	0	-0.694000	3.580000	-0.599000
40	1	0	-2.199000	4.287000	-1.222000
41	1	0	-0.990000	0.623000	-3.748000

2.5 Cartesian coordinates for the low-energy optimized conformers of 2*S*-**3** at B3LYP/6-31+G (d, p) level of theory in MeOH.

Conformer **3a**		Standard Orientation (Ångstroms)			
Center Number	Atom	Type	X	Y	Z
1	6	0	-0.319000	-0.918000	-1.375000
2	6	0	-0.224000	-2.294000	-1.329000
3	6	0	-0.255000	-2.889000	-0.066000
4	6	0	-0.380000	-2.098000	1.095000
5	6	0	-0.475000	-0.701000	1.007000
6	6	0	-0.435000	-0.125000	-0.251000
7	8	0	-0.474000	1.225000	-0.488000

8	6	0	-0.482000	1.379000	-1.935000
9	6	0	-0.289000	-0.013000	-2.493000
10	6	0	0.607000	2.359000	-2.420000
11	6	0	0.379000	3.741000	-1.794000
12	6	0	2.030000	1.893000	-2.088000
13	8	0	-0.113000	-0.334000	-3.654000
14	6	0	-0.409000	-2.697000	2.424000
15	6	0	-0.720000	-1.947000	3.494000
16	8	0	-0.996000	-0.596000	3.431000
17	6	0	-0.533000	0.110000	2.268000
18	6	0	-0.831000	-2.519000	4.874000
19	8	0	0.513000	2.539000	-3.840000
20	1	0	-0.125000	-2.883000	-2.234000
21	1	0	-0.182000	-3.972000	0.013000
22	1	0	-1.481000	1.721000	-2.235000
23	1	0	-0.620000	4.118000	-2.042000
24	1	0	1.091000	4.472000	-2.192000
25	1	0	0.480000	3.720000	-0.704000
26	1	0	2.270000	0.951000	-2.593000
27	1	0	2.175000	1.759000	-1.011000
28	1	0	2.768000	2.621000	-2.445000
29	1	0	-0.215000	-3.760000	2.517000
30	1	0	-1.208000	0.962000	2.135000
31	1	0	0.467000	0.502000	2.491000
32	1	0	-1.840000	-2.361000	5.268000
33	1	0	-0.627000	-3.594000	4.893000
34	1	0	-0.118000	-2.028000	5.543000
35	1	0	0.573000	1.657000	-4.264000

Conformer **3b**		Standard Orientation (Ångstroms)			
Center Number	Atom	Type	X	Y	Z
1	6	0	-0.357000	-0.858000	-1.284000
2	6	0	-0.262000	-2.235000	-1.233000
3	6	0	-0.282000	-2.827000	0.031000
4	6	0	-0.399000	-2.033000	1.190000
5	6	0	-0.498000	-0.636000	1.099000
6	6	0	-0.467000	-0.064000	-0.161000
7	8	0	-0.514000	1.285000	-0.402000
8	6	0	-0.501000	1.439000	-1.861000
9	6	0	-0.345000	0.037000	-2.410000
10	6	0	0.620000	2.420000	-2.258000

11	6	0	2.015000	1.948000	-1.834000
12	6	0	0.599000	2.736000	-3.754000
13	8	0	-0.235000	-0.320000	-3.568000
14	6	0	-0.419000	-2.628000	2.522000
15	6	0	-0.724000	-1.876000	3.591000
16	8	0	-1.002000	-0.526000	3.527000
17	6	0	-0.550000	0.177000	2.358000
18	6	0	-0.825000	-2.444000	4.973000
19	8	0	0.379000	3.669000	-1.579000
20	1	0	-0.172000	-2.827000	-2.138000
21	1	0	-0.208000	-3.910000	0.112000
22	1	0	-1.489000	1.828000	-2.135000
23	1	0	2.073000	1.800000	-0.750000
24	1	0	2.768000	2.707000	-2.076000
25	1	0	2.299000	1.015000	-2.329000
26	1	0	-0.393000	3.082000	-4.065000
27	1	0	0.874000	1.871000	-4.365000
28	1	0	1.295000	3.550000	-3.989000
29	1	0	-0.221000	-3.690000	2.616000
30	1	0	-1.229000	1.027000	2.227000
31	1	0	0.449000	0.574000	2.573000
32	1	0	-1.831000	-2.287000	5.374000
33	1	0	-0.618000	-3.519000	4.994000
34	1	0	-0.108000	-1.950000	5.637000
35	1	0	0.277000	3.464000	-0.631000

Conformer **3c**		Standard Orientation (Ångstroms)			
Center Number	Atom	Type	X	Y	Z
1	6	0	-0.313000	-0.971000	-1.313000
2	6	0	-0.196000	-2.346000	-1.262000
3	6	0	-0.177000	-2.937000	0.003000
4	6	0	-0.285000	-2.143000	1.163000
5	6	0	-0.413000	-0.749000	1.072000
6	6	0	-0.414000	-0.178000	-0.189000
7	8	0	-0.503000	1.169000	-0.430000
8	6	0	-0.450000	1.330000	-1.886000
9	6	0	-0.360000	-0.078000	-2.439000
10	6	0	0.739000	2.243000	-2.251000
11	6	0	0.928000	2.387000	-3.762000
12	6	0	0.544000	3.636000	-1.633000
13	8	0	-0.372000	-0.441000	-3.601000

14	6	0	-0.268000	-2.737000	2.495000
15	6	0	-0.568000	-1.989000	3.570000
16	8	0	-0.875000	-0.646000	3.510000
17	6	0	-0.461000	0.065000	2.331000
18	6	0	-0.631000	-2.558000	4.954000
19	8	0	1.960000	1.714000	-1.710000
20	1	0	-0.118000	-2.936000	-2.169000
21	1	0	-0.083000	-4.018000	0.083000
22	1	0	-1.407000	1.764000	-2.199000
23	1	0	1.250000	1.446000	-4.219000
24	1	0	1.721000	3.110000	-3.987000
25	1	0	0.009000	2.724000	-4.252000
26	1	0	0.484000	3.584000	-0.540000
27	1	0	-0.366000	4.117000	-2.008000
28	1	0	1.397000	4.286000	-1.859000
29	1	0	-0.047000	-3.795000	2.586000
30	1	0	-1.162000	0.898000	2.211000
31	1	0	0.532000	0.486000	2.527000
32	1	0	-1.632000	-2.422000	5.373000
33	1	0	-0.401000	-3.629000	4.971000
34	1	0	0.089000	-2.050000	5.603000
35	1	0	1.852000	1.662000	-0.742000

Conformer **3d**		Standard Orientation (Ångstroms)			
Center Number	Atom	Type	X	Y	Z
1	6	0	-0.197000	-0.999000	-1.334000
2	6	0	-0.036000	-2.369000	-1.260000
3	6	0	-0.118000	-2.951000	0.007000
4	6	0	-0.352000	-2.153000	1.145000
5	6	0	-0.508000	-0.763000	1.031000
6	6	0	-0.433000	-0.204000	-0.233000
7	8	0	-0.587000	1.132000	-0.500000
8	6	0	-0.397000	1.287000	-1.944000
9	6	0	-0.191000	-0.118000	-2.471000
10	6	0	0.782000	2.250000	-2.201000
11	6	0	1.105000	2.398000	-3.689000
12	6	0	0.471000	3.634000	-1.612000
13	8	0	-0.089000	-0.490000	-3.626000
14	6	0	-0.442000	-2.737000	2.479000
15	6	0	-0.465000	-1.942000	3.562000

16	8	0	-0.439000	-0.564000	3.502000
17	6	0	-0.829000	0.039000	2.256000
18	6	0	-0.487000	-2.483000	4.958000
19	8	0	1.968000	1.773000	-1.546000
20	1	0	0.143000	-2.962000	-2.150000
21	1	0	0.004000	-4.028000	0.105000
22	1	0	-1.337000	1.677000	-2.354000
23	1	0	1.504000	1.469000	-4.108000
24	1	0	1.887000	3.150000	-3.843000
25	1	0	0.223000	2.696000	-4.264000
26	1	0	0.313000	3.583000	-0.529000
27	1	0	-0.419000	4.075000	-2.073000
28	1	0	1.314000	4.319000	-1.763000
29	1	0	-0.447000	-3.818000	2.570000
30	1	0	-1.909000	0.222000	2.297000
31	1	0	-0.324000	1.010000	2.212000
32	1	0	-1.384000	-2.137000	5.481000
33	1	0	-0.488000	-3.578000	4.976000
34	1	0	0.392000	-2.138000	5.511000
35	1	0	1.775000	1.719000	-0.591000

2.6 Cartesian coordinates for the low-energy optimized conformers of 7*R*-**4** at B3LYP/6-31+G (d, p) level of theory in MeOH.

Conformer **4a**		Standard Orientation (Ångstroms)			
Center Number	Atom	Type	X	Y	Z
1	6	0	0.155000	2.123000	0.656000
2	6	0	0.334000	2.094000	2.024000
3	6	0	0.209000	0.855000	2.655000
4	6	0	-0.084000	-0.313000	1.911000
5	6	0	-0.282000	-0.251000	0.519000
6	6	0	-0.143000	0.986000	-0.086000
7	8	0	-0.272000	1.242000	-1.430000
8	6	0	-0.054000	2.615000	-1.599000
9	6	0	0.229000	3.244000	-0.270000
10	6	0	-0.036000	3.204000	-2.815000
11	6	0	-0.339000	2.425000	-4.070000
12	6	0	0.213000	4.667000	-3.065000
13	8	0	0.418000	4.400000	0.023000
14	6	0	-0.249000	-1.639000	2.616000

15	6	0	-0.186000	-2.846000	1.647000
16	8	0	-1.018000	-2.592000	0.510000
17	6	0	-0.565000	-1.488000	-0.280000
18	6	0	-0.771000	-4.066000	2.375000
19	8	0	1.187000	-3.070000	1.296000
20	6	0	1.427000	-4.212000	0.486000
21	1	0	0.564000	2.997000	2.579000
22	1	0	0.342000	0.792000	3.734000
23	1	0	-0.520000	1.362000	-3.883000
24	1	0	-1.233000	2.829000	-4.558000
25	1	0	0.500000	2.497000	-4.770000
26	1	0	0.435000	5.243000	-2.167000
27	1	0	1.062000	4.791000	-3.746000
28	1	0	-0.668000	5.122000	-3.531000
29	1	0	0.527000	-1.741000	3.385000
30	1	0	-1.229000	-1.604000	3.109000
31	1	0	0.331000	-1.786000	-0.836000
32	1	0	-1.357000	-1.285000	-1.008000
33	1	0	-0.137000	-4.371000	3.214000
34	1	0	-1.782000	-3.861000	2.744000
35	1	0	-0.867000	-4.924000	1.699000
36	1	0	2.452000	-4.149000	0.111000
37	1	0	0.749000	-4.251000	-0.372000
38	1	0	1.344000	-5.127000	1.078000

Conformer **4b**		Standard Orientation (Ångstroms)			
Center Number	Atom	Type	X	Y	Z
1	6	0	0.057000	1.994000	0.455000
2	6	0	0.237000	1.971000	1.823000
3	6	0	0.097000	0.738000	2.464000
4	6	0	-0.209000	-0.430000	1.726000
5	6	0	-0.409000	-0.374000	0.332000
6	6	0	-0.256000	0.857000	-0.280000
7	8	0	-0.386000	1.108000	-1.626000
8	6	0	-0.152000	2.477000	-1.802000
9	6	0	0.143000	3.109000	-0.478000
10	6	0	-0.131000	3.061000	-3.022000
11	6	0	-0.446000	2.280000	-4.272000
12	6	0	0.135000	4.520000	-3.280000
13	8	0	0.352000	4.262000	-0.191000
14	6	0	-0.390000	-1.754000	2.434000

15	6	0	-0.254000	-2.949000	1.460000
16	8	0	-1.140000	-2.714000	0.350000
17	6	0	-0.717000	-1.613000	-0.460000
18	6	0	-0.777000	-4.226000	2.128000
19	8	0	1.068000	-3.183000	0.960000
20	6	0	2.111000	-3.270000	1.916000
21	1	0	0.475000	2.876000	2.372000
22	1	0	0.226000	0.683000	3.543000
23	1	0	-0.639000	1.220000	-4.079000
24	1	0	-1.336000	2.691000	-4.759000
25	1	0	0.392000	2.338000	-4.974000
26	1	0	0.364000	5.098000	-2.384000
27	1	0	0.986000	4.630000	-3.961000
28	1	0	-0.741000	4.982000	-3.748000
29	1	0	0.321000	-1.834000	3.263000
30	1	0	-1.402000	-1.740000	2.859000
31	1	0	0.158000	-1.913000	-1.048000
32	1	0	-1.535000	-1.410000	-1.160000
33	1	0	-0.248000	-4.450000	3.060000
34	1	0	-1.847000	-4.149000	2.349000
35	1	0	-0.645000	-5.086000	1.462000
36	1	0	3.017000	-3.594000	1.396000
37	1	0	1.891000	-4.003000	2.696000
38	1	0	2.316000	-2.291000	2.357000

Conformer **4c**		Standard Orientation (Ångstroms)			
Center Number	Atom	Type	X	Y	Z
1	6	0	0.114000	2.127000	0.669000
2	6	0	0.322000	2.084000	2.033000
3	6	0	0.545000	0.830000	2.604000
4	6	0	0.549000	-0.339000	1.806000
5	6	0	0.355000	-0.264000	0.414000
6	6	0	0.127000	0.989000	-0.127000
7	8	0	-0.109000	1.260000	-1.454000
8	6	0	-0.282000	2.646000	-1.554000
9	6	0	-0.149000	3.267000	-0.198000
10	6	0	-0.458000	3.265000	-2.734000
11	6	0	-0.566000	2.492000	-4.024000
12	6	0	-0.627000	4.750000	-2.911000
13	8	0	-0.283000	4.421000	0.161000
14	6	0	0.825000	-1.683000	2.433000

15	6	0	0.386000	-2.869000	1.542000
16	8	0	0.871000	-2.655000	0.213000
17	6	0	0.338000	-1.500000	-0.438000
18	6	0	-1.138000	-3.069000	1.588000
19	8	0	1.031000	-4.022000	2.117000
20	6	0	0.936000	-5.207000	1.340000
21	1	0	0.312000	2.990000	2.631000
22	1	0	0.721000	0.757000	3.676000
23	1	0	-0.442000	1.413000	-3.891000
24	1	0	-1.549000	2.656000	-4.480000
25	1	0	0.202000	2.826000	-4.729000
26	1	0	-0.546000	5.322000	-1.987000
27	1	0	0.135000	5.134000	-3.597000
28	1	0	-1.612000	4.965000	-3.339000
29	1	0	1.909000	-1.743000	2.604000
30	1	0	0.346000	-1.759000	3.417000
31	1	0	0.949000	-1.339000	-1.333000
32	1	0	-0.684000	-1.717000	-0.769000
33	1	0	-1.455000	-3.448000	2.565000
34	1	0	-1.688000	-2.145000	1.385000
35	1	0	-1.460000	-3.791000	0.830000
36	1	0	1.500000	-5.991000	1.853000
37	1	0	1.376000	-5.069000	0.348000
38	1	0	-0.101000	-5.543000	1.254000
