# Supplementary figures and images for: New Benzofuranoids and Phenylpropanoids from the Mangrove Endophytic Fungus, Aspergillus sp. ZJ-68
Source: Mar Drugs. 2019 Aug 18;17(8):478. doi: 10.3390/md17080478 (PMC6723808; doi:10.3390/md17080478)

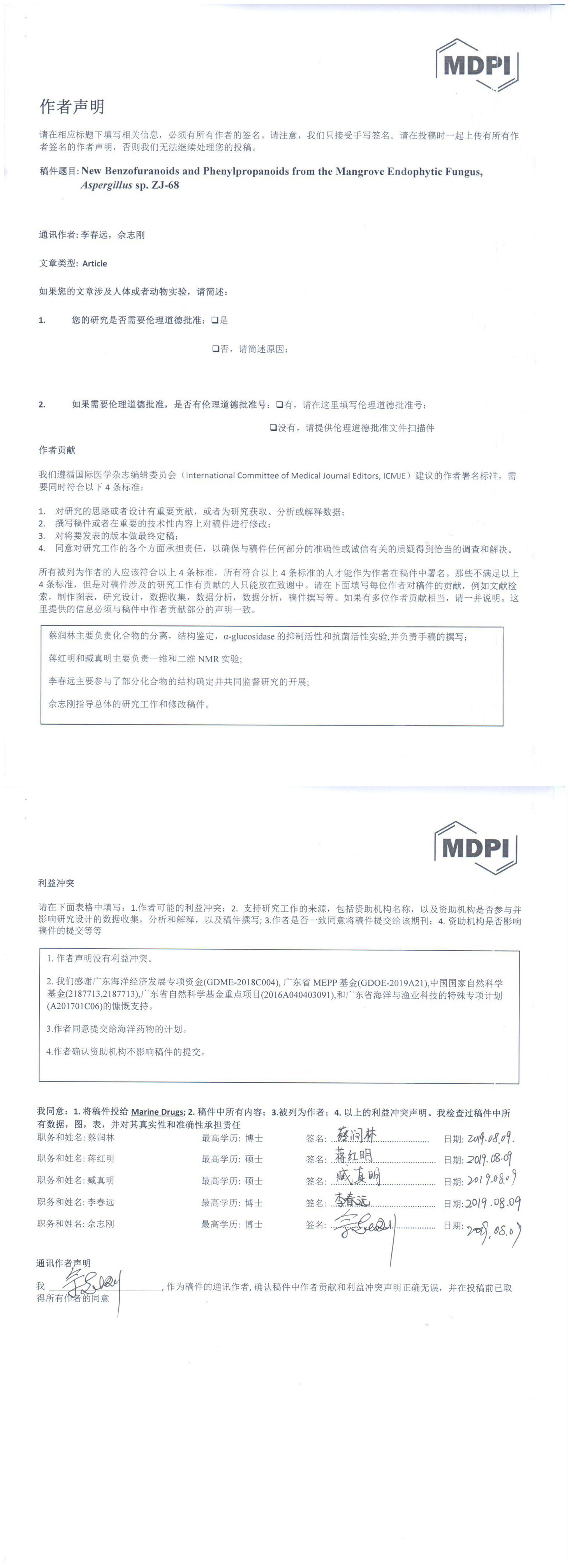

Supplement: Supplementary file 1 [file marinedrugs-17-00478-s001.zip › MDPI-author-signatures-╓╨╬─░μ▒╛╥╜╥⌐.jpg]

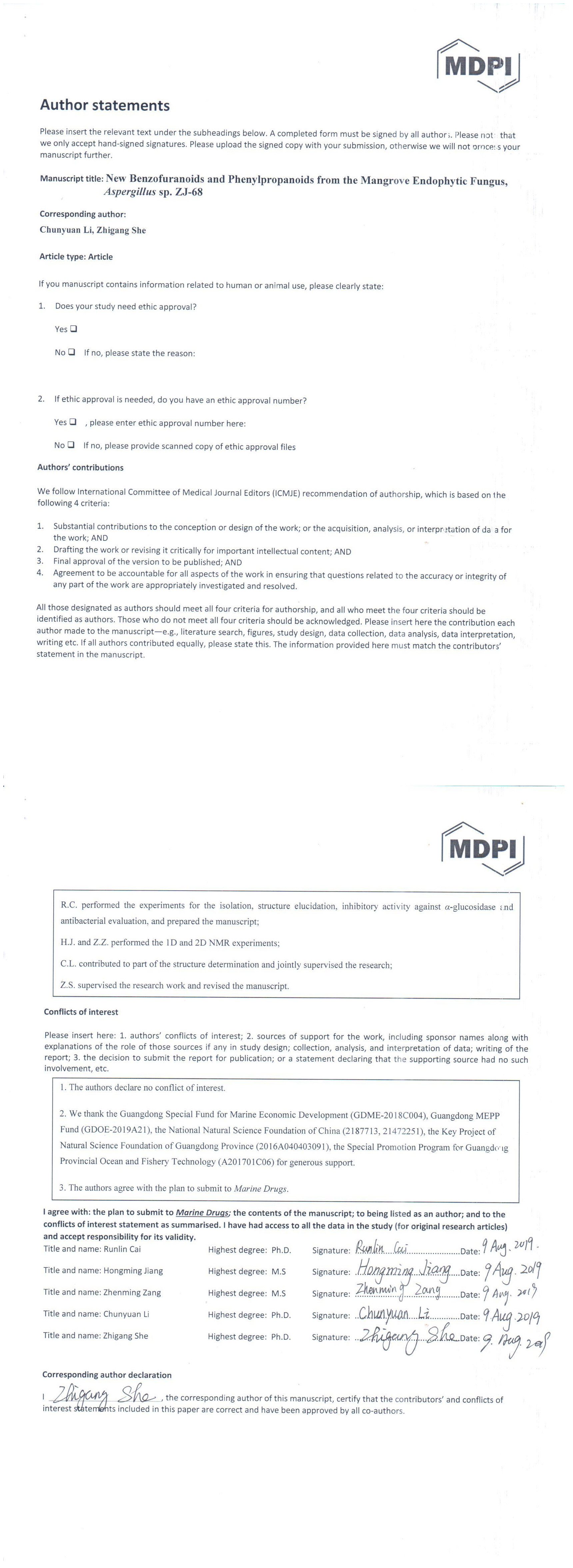

Supplement: Supplementary file 1 [file marinedrugs-17-00478-s001.zip › MDPI-author-signatures-╙ó╬─░μ▒╛╥╜╥⌐.jpg]
